# Supplementary material for: Process evaluation and exploration of telehealth in motor neuron disease in a UK specialist centre
Source: BMJ Open. 2019 Oct 22;9(10):e028526. doi: 10.1136/bmjopen-2018-028526 (PMC6830641; doi:10.1136/bmjopen-2018-028526)
Supplement: Supplementary data [file bmjopen-2018-028526supp001.pdf]

Telehealth in motor neurone disease to increase access to specialist multidisciplinary care: a pilot, feasibility study.

Hobson EV., Bradburn M., Baird WO, Cooper CL, Mawson S, Quinn A, Shaw PJ, Walsh T, McDermott CJ

Supplementary data files

TiM protocol V1.5..Page 2
TiM statistics analysis plan V1.3.......................Page 45
Shadow monitoring form ....Page 71

Hobson E, *et al. BMJ Open* 2019; 9:e028526. doi: 10.1136/bmjopen-2018-028526

TiM™

Telehealth in Motor Neurone Disease

Telehealth in Motor Neurone Disease: A single centre, randomised controlled feasibility and pilot study of the use of the TiM telehealth system to deliver highly specialised care in Motor Neurone Disease at a distance

The University Of Sheffield.

Sheffield Teaching Hospitals NHS
NHS Foundation Trust

SITraN
Sheffield Institute for Translational Neuroscience

D4D
Devices for Dignity

CLAHRC for South Yorkshire

2

General Information

Sponsor identifier: 17165
National Institute for Health Research Portfolio ID: 17022
Trial Identifier: ISRCTN26675465

Sponsor

Sheffield Teaching Hospitals NHS Foundation Trust
Tanya.turgoose@sth.nhs.uk
Sheffield Teaching Hospitals NHS Foundation Trust Research Department
11 Broomfield Road Sheffield S10 2SE
Tel: (0114) 2265942
Fax: (0114) 2265937
Email: Tanya.Turgoose@sth.nhs.uk

Principal investigator & Trial Manager

Dr Esther Hobson
National Institute for Health Research Doctoral Research Fellow
Neurology Specialty Registrar
Sheffield Institute of Translational Neuroscience University of Sheffield
385a Glossop Road
Sheffield
S10 2HQ
Tel: (0114) 2222261
Fax: (0114) 2222290
Email: esther.hobson@gmail.com

Chief investigator

Dr. Christopher McDermott
Reader and Honorary Consultant Neurologist
Sheffield Institute of Translational Neuroscience University of Sheffield
385a Glossop Road
Sheffield
S10 2HQ
Tel: (0114) 2222261
Fax: (0114) 2222290
Email: c.j.mcdermott@sheffield.ac.uk

This study is a non-commercial, portfolio study supported by the DeNDRoN (Dementia and Neurodegenerative Diseases Clinical Research Network). It is funded through a National Institute for Health Research Doctoral Research Fellowship grant.

Abbreviations

AE	Adverse event
ALS	Amyotrophic lateral sclerosis
ALSAQ-40	Amyotrophic Lateral Sclerosis Assessment Questionnaire – long form
ALS-FRS-R	Amyotrophic lateral sclerosis rating scale-revised
BiPAP	Bi-level positive pressure non-invasive ventilation
CBI	Carer burden inventory
CI	Chief Investigator
CLRN	Comprehensive Local Research Network
CONSORT	Consolidated standards of reporting trials
CRF	Clinical Research Facility, Sheffield Teaching Hospitals
CSS-MND	Clinical Saliva Scale for Motor Neurone disease
CTRU	Clinical trials research unit, University of Sheffield
EQ-5D-3L	EuroQol Group Health Questionnaire,
GCP	Good clinical practice
GP	General practitioner
HADS	Hospital Anxiety and Depression Scale
HTA	Health Technology Assessment
MCSI	Modified Caregiver Strain Index
MDT	Multidisciplinary team
MND	Motor neurone disease
NICE	National Institute for Health and Clinical Excellence
NHS	National Health Service
NIHR	National Institute for Heath Research
NIV	Non-invasive ventilation
PHQ	Patient Health Questionnaire
PI	Principal Investigator
SAE	Serious adverse event
ScHARR	School of Health and Related Research, University of Sheffield
SF-36 RAND	36-Item Short Form Survey from the RAND Medical Outcomes Study
SITraN	Sheffield Institute of Translational Neuroscience
STH	Sheffield Teaching Hospitals
SU	Sheffield University
Telecare	A system of sensors, alarms or communication in the home used to support safe living
Telehealth	Remote monitoring of patients physiology or patient reported measures, forwarded to a central service with the aim to diagnoses or monitor a medical condition
Telemedicine	Videoconferencing consultation
TMG	Trial management Group
TSC	Trial Steering Committee
TiM	Telehealth in Motor neurone disease
TM	Trial manager

Contents

1. Title and abstract
2. Lay summary
3. Background
4. Summary and hypothesis
5. Research objectives
    a. Objective of the pilot study
    b. Objectives of the full scale study
    c. Justification of the pilot study
6. Study Methodology
    a. Participant recruitment and selection
    b. Consent
    c. Randomisation
    d. Inclusion and exclusion criteria
    e. Withdrawal
    f. Compliance
    g. Sample size
    h. Blinding
7. Study treatment
8. Data collection
    a. Quantitative data collection
    b. Qualitative sub-study
    c. Shadow monitoring protocol
    d. Process evaluation
9. Analysis
    a. Feasibility and quanitative analysis
    b. Qualitative analysis
10. Data entry, security and confidentiality
11. Safety and safety assessments
12. Ethical considerations
13. Finance and indemnity
14. Dissemination and publication
15. References

Appendix 1 Drugs produced by Abbott Healthcare Ltd.
Appendix 2 CONSORT flow diagram
Appendix 3 Qualitative interview topic guides

This document describes a clinical study. The protocol is not intended for use as a guide to the treatment of other patients. Amendments may be necessary; these will be circulated to investigators in the study.

1. Telehealth in Motor Neurone Disease: The TiM ™ Study

Telehealth in Motor Neurone Disease: A single centre, randomised controlled pilot study of the use of the TiM ™ telehealth system to deliver highly specialised care in Motor Neurone Disease at a distance.

Abstract

Objectives

People with motor neurone disease benefit from the specialist care provided by multidisciplinary teams. As their disease progresses patients struggle to attend hospital and find it difficult to access the care they need. The aim of the TiM system is to improve access to this specialist care by using technology to monitor, educate and communicate with our patients and their carers.

This is a pilot study of the TiM ™ telehealth system. The pilot study is designed to assess the feasibility, acceptability and safety of the telehealth system in clinical practice and of conducting a full study of the system. It will also allow a process evaluation of the system to determine how the telehealth system could be effectively utilised within an NHS service.

Methods

This is a single-centre, randomized controlled mixed methods pilot study of the TiM ™ telehealth system. It will recruit 40 patients along with their primary informal carer. 20 will be assigned to use the TiM system for a minimum of 6 months (intervention) and 20 will be assigned usual care (control). Quantitative outcome data will be collected at baseline, three and six months, six monthly thereafter and at the end of the trial. Qualitative interviews with participants and staff and analysis of the system in use will enable a process evaluation of the system and the trial methodology. It will also assess the safety of the system in a clinical setting.

Results

Results of this pilot will determine whether a large, multi-centre full trial is appropriate and enable further development of the TiM system. It is proposed that the TiM system could be adopted into the care of patients with MND throughout the UK.

2. Lay summary

Motor neurone disease is a condition affecting approximately 5 000 people in the UK. It results in progressive weakness in muscles causing paralysis, disability, and eventually death after an average of only three to five years. To receive the expert care provided by motor neurone disease multidisciplinary teams most patients have to travel to regional centres, whilst community-based care is usually provided by non-specialist teams. Between clinic appointments and towards the end of their lives when patients are unable to travel to clinic and they may be unable to access the specialist assessment and care provided in these centres.

Telehealth has been shown to increase access to specialist care in patients with chronic disease, regardless of geography or the ability to travel. The overall purpose of this pilot study is to test the feasibility and acceptability of the TiM telehealth system. The TiM system is web-based system that enables weekly monitoring of patients and carers' health and wellbeing. It has been developed by the Sheffield Motor Neurone Disease Care Centre team in partnership with industry (Abbott Healthcare Products Ltd and Carematix) and other experts within the NHS and the University of Sheffield. The study will also determine whether it would be feasible to conduct a larger study of the system to examine the effectiveness of the TiM system.

Patients with motor neurone disease who are cared for by the Sheffield motor neurone disease clinic will be invited to take part in the trial. 40 patients will be recruited. Their primary informal carer (usually their spouse or close relative) will also be invited to participate in carer monitoring. All patients will continue their usual care but half will also be randomised to use the TiM telehealth system for a minimum of six months.

Information about the participants will be collected at the start, three and six months, and then every six months until the end of the trial by postal questionnaires and during routine appointments. Up to 20 participants in the control group will be interviewed at the start at the trial to explore their experiences on completing the postal questionnaires. Up to 20 participants who are using the TiM system will be interviewed at one and six months to understand the effect the trial and the system has on their lives in more depth. The clinical staff will also be interviewed at the end of the trial.

A pilot study is a small-scale study that is carried out to determine whether a larger study is practical. It will also enable the identification and resolution of any problems with either the telehealth system or the trial procedures.

3. Background

There are approximately 5 000 people in the UK suffering from motor neurone disease (MND) at any one time (1). MND is an incurable disease causing progressive weakness of muscles involving the limbs, speech and swallowing leading to progressive disability and eventual respiratory failure. The average life expectancy following diagnosis is two to three years but the course of MND can vary from only a few months to over 10 years. The distress and burden of the disease affects patients, their family and carers and the relenting progression of disability causes social, emotional and financial strain (2, 3).

There are 22 specialist multidisciplinary MND care centres in the UK. Expert clinicians and therapists offer interventions such as riluzole (which can improve survival by approximately two to three months) and gastrostomy feeding to promote good nutrition (4). Treatment of respiratory failure with non-invasive ventilation (NIV) improves both quality of life and life-expectancy by, on average, 11 months (5). Attendance at specialist MND clinics has also been reported to improve survival independent of these other interventions (6, 7).

The traditional model of care is to review patients at the MND centre at fixed regular intervals. This model is not responsive to patient or carer needs (which can change rapidly) and requires the patient and their family to undertake progressively more difficult journeys to clinic at a time predicted by the clinician at their last meeting. Given the burden associated with travelling to clinic, it is important that visits occur when they are most needed. Some patients whose needs have not changed may not benefit from a clinic appointment at the previously predicted interval whereas others may need more timely intervention.

The highly specialist services provided by the MND clinic are contrasted by the services most patients receive in their community (8). These community teams, who have limited experience in caring for patients with MND, are usually the first point of contact for patients between clinic visits. Lack of expertise in MND amongst community teams and limited access to specialist staff and equipment (particularly at the end stages of the disease) causes patients and their carers to experience significant difficulties (2, 3, 9-12). Research conducted in SITraN and by others, highlights the major impact that caring for someone with MND has on the physical and emotional well being of carers, as well as patients (3, 11, 13-16). Where access to specialist services and community support is limited, this impact is even more notable (3, 11, 13-16). This is particularly a problem in the later stages, when it is usually impossible to attend clinic, when arguably the most care is needed. It is therefore essential that the input from the specialist centre is still possible both between visits and when patients become unable to travel.

Telehealth to provide specialist care in MND

In the last few years, technology has developed sufficiently to allow high quality communication between patient and their care team at a potentially reasonable cost.

Hobson E, *et al. BMJ Open* 2019; 9:e028526. doi: 10.1136/bmjopen-2018-028526

Trials have shown that telehealth is an acceptable way to improve access to specialist expertise and facilitate self-management patients with long-term health conditions (17-21). In some cases this approach has been associated with a reduction in hospital admissions (17, 18, 20). In 2012, in response to research evidence and the need to provide cost-effective care to an expanding population of patients with chronic disease, the UK government created the “3millionlives” campaign (22). This project in aims foster NHS, academic and industry collaboration in order to provide telehealth services to up to three million people with chronic health and social care needs in the UK.

The problems faced in MND are unlike many common chronic diseases in which telehealth has been previously trialed. Care for MND requires holistic and multi-disciplinary expertise and the use of uncommon interventions such as non-invasive ventilation and gastrostomy feeding. To date, use of telehealth in MND is limited, although small studies do show promise in certain niche areas. Telehealth systems using telephone consultation have been developed with some success in Italy and Portugal, to remotely manage patients who require home ventilation. These systems were associated with a reduction in emergency healthcare usage; more efficient use of staff time and potential cost savings (23-28) In Holland and rural Scotland, MND services have used video-conferencing (29, 30). Both approaches have potential benefits but telehealth used in this way is labour intensive and costly and care is driven by the priorities identified by the clinician rather than those of the patient. No telehealth system has been developed to provide frequent, holistic and highly specialist care to patients with MND at all stages of their illness.

We propose that telehealth could enable people with MND to have better access to the specialist monitoring and care that they require. Patients with MND are able to accurately report their level of disability and appropriate questions can identify new symptoms or early signs of respiratory failure (31-33). These features would suggest that a system of remote, question-based monitoring could provide regular, accurate clinical information to enable the clinician to detect and better manage problems without the patient needing to attend hospital. Telehealth provides the opportunity to provide education and reassurance and support to enable patients to better manage their own care (a core requirement of the National Service Framework for Long-term Conditions (34)).

There are estimated to be 10 million people in the UK living with a neurological condition (1). Both the common diseases such as Parkinson’s disease and epilepsy and rarer conditions such as muscular dystrophy and MND require specialist, multidisciplinary support from specialist services. A successful telehealth system may therefore be able to improve the services provided to many patients in the UK and their families.

4. Summary and hypothesis

Summary

We will undertake a pilot study of the use of the TiM telehealth system to improve the care of patients and their carers living with motor neurone disease. Whilst telehealth services have been used successfully in other long-term conditions, no service of this kind exists for patients with motor neurone disease. A pilot randomised controlled trial will employ a mixed methods approach to explore the feasibility and acceptability of using the TiM system to improve access to specialist care in MND. The pilot study will also explore the feasibility of a full-scale trial.

Hypothesis

The TiM telehealth system will:

- Improve the quality of life of patients with MND
- Improved clinical outcomes for patients with MND
- Improve quality of life and other measures of well being for the primary informal carers of patients living with MND.
- Be acceptable to patients, carers and staff
- Lead to more cost effective utilisation of heath care resources

Hobson E, *et al*. *BMJ Open* 2019; 9:e028526. doi: 10.1136/bmjopen-2018-028526

5 Research objectives

5a. Objectives of the pilot study

- Determine the requirements of a full-scale study of the TiM system
  - Determine recruitment, retention and withdrawal rates.
  - Determine the most acceptable and appropriate outcome measure(s) that reflect the impact of the TiM system on patients and carers and health resources.
  - Provide an estimate of the resources required to conduct a full-scale study.
- Study the use of the TiM system in clinical practice
  - Assess the relationship between the benefits of the TiM system perceived by staff and participants with those captured by the outcome measures
  - Assessing participants' use and compliance with the TiM system
  - Health-care staff qualitative interviews and focus group
- Assess the safety of the TiM system using:
  - A shadow monitoring protocol
  - Health-care staff qualitative interviews and focus group
  - Analysis of technical and clinical adverse events

5b. Objectives of the full-scale study

Proposed primary end-point

- Patient quality of life (outcome measure(s) to be determined in the pilot trial)

Proposed secondary end-points

Patient outcomes

- Severity of pain
- Severity of oropharygeal secretions
- Incidence of depression and anxiety
- Time from diagnosis to death

Carer outcomes

- Quality of life
- Carer Burden
- Incidence of depression and anxiety

Health economic outcomes involving a cost utility analysis using costs of the system, costs of associated care requirements, EQ5D and patient survival

Safety of the TiM system

- Frequency of adverse events

5c. Justification of the pilot study

Since this type of telehealth has never been evaluated in those with MND a pilot study is necessary to determine how a full-scale evaluation of its clinical and cost-effectiveness could be conducted and to gain a better understanding of how the TiM system would work. This includes evaluating recruitment and retention, as well and resource requirements. By evaluating compliance and safety monitoring and using qualitative the study will also enable a better understanding of how the telehealth system is used by patients, carers and staff.

A number of the proposed benefits of telehealth such as improving quality of life, providing reassurance and support, prompting self-care and a more efficient use of resources (18, 35-40) may be difficult to quantify. The validated measures of quality of life most commonly used in research (EQ5D and SF-36) were not specifically designed for patients with MND or their carers. The ALSAQ-40 tool better encompasses dimensions of life that are particularly affected by MND such as social and emotional function but it is unclear whether the ALSAQ-40 would fully reflect the impact of telehealth (41).

Data from quantitative elements of a randomised controlled trial will not, in isolation determine which outcome measures best reflect the impact of the intervention. It would also not fully explain how the TiM system would be used in the real world and what factors would influence its adoption and success. Utilising mixed methods will allow the combination of quantitative data with more in-depth results from qualitative interviews that will explore participants' experiences in more depth. It will also allow explanation of outcomes that occurred (particularly those that were unexpected) and understand why (and in what context) aspects of the system were successful or unsuccessful which could lead to improvements in the TiM system.

Hobson E, *et al*. *BMJ Open* 2019; 9:e028526. doi: 10.1136/bmjopen-2018-028526

6. Study Methodology

We will conduct a randomised controlled pilot trial comparing the TiM telehealth service and standard care with standard care alone. The intervention and follow-up period will be a minimum of 6 months. Quantitative data will be collected at 0, 3 and 6 months then every six months until the patient finishes the trial. Qualitative data will be collected at baseline in the control arm and at 1 and 6 months for a selection of patients in the intervention arm.

6a. Participant recruitment and selection

Pre-screening will identify a list of potential patients who cared for by the Sheffield Teaching Hospitals MND care centre clinic as part of usual care using the MND care centre "ARC" clinical database. Each patient will be assigned a number. The Clinical Research Facility at the Royal Hallamshire Hospital, Sheffield Teaching Hospitals NHS trust will generate random numbers to identify the patients to invite. These patients will be sent a letter of invitation to participate. This will be accompanied by patient and carer information leaflets and a return slip to indicate their interest. Those who do not return the slip will be followed up by telephone or at clinic, if appropriate a minimum of once and a maximum of twice. A log will be kept in order to complete the CONSORT diagram (Appendix 2) (42).

Patients and their primary carer who express an interest will be invited to discuss the trial in a face-to-face meeting with the PI and also via telephone with their consultant neurologist (Dr. Christopher McDermott or Professor Dame Pamela Shaw, Sheffield Teaching Hospitals MND Care Centre). The Sheffield MND Care Centre sees approximately 120 new patients with MND per year. At any time there are approximately 300 patients attending clinic. We expect to be able to recruit a minimum of four patients per month.

Participants in the intervention arm will be invited to participate in qualitative interviews, conducted at month one and month six. Purposive sampling will be used to reflect the variation and predefined patient prognostic factors thereby capturing a range of experiences. Interviews will continue until data saturation is reached or 20 interviews have been conducted. All participants assigned to the control arm will be invited to be interviewed after completion of the baseline questionnaires to determine the feasibility and acceptability of these measures and their views on participating in the trial. The qualitative component will provide information not easily obtained from questionnaires that will facilitate understanding of the intervention from the perspective of all stakeholder groups.

Hobson E, *et al*. *BMJ Open* 2019; 9:e028526. doi: 10.1136/bmjopen-2018-028526

6b. Consent

Following indication of their interest to participant potential participants will be met at a mutually agreeable location, preferably the patients' home. They will have further opportunity to discuss the trial with the PI and decide whether they wish to participate. Willing participants will be asked to give informed written consent or use an appropriate witnessed alternative (which may include verbal consent or via a communication device) for screening and involvement in the trial. Carer consent will be obtained by full written consent.

In versions prior to V1.5 of the protocol both patient and carer consent were required. V1.5. has amended the inclusion criteria to allow a patient to participate with carer participation.

If one or both consent to the study a member of the study team will initiate the screening process. Participants will be screened and recruited by the PI according to the CONSORT principles and Good Clinical Practice (42, 43).

Those who decline participation will be invited to give their reasons in order to identify common factors; this may help recruitment strategies and identify potential problems for compliance. Basic anonymised details of these patients (age, gender, reason for exclusion) will be collected on all eligible patients in order to fulfill the CONSORT flow chart (Appendix 2) (42, 43).

6c. Randomisation

Once recruited, randomisation will be performed using the independent web-based system http://www.sealedenvelope.com using block randomisation. All patients and carers will be assigned an anonymous individual study code, and a recruitment log held by the research team in SITraN.

6b. Inclusion and exclusion criteria

Inclusion criteria:

- Patients aged 18 years or over who have attended the MND clinic at the Royal Hallamshire Hospital, Sheffield.
- Patients with amyotrophic lateral sclerosis diagnosed by a consultant neurologist with symptom onset within the last three years.
    - Or
- Patients with amyotrophic lateral sclerosis, primary muscular atrophy or progressive lateral sclerosis diagnosed by a consultant neurologist with a deterioration in their condition as evidenced by a deterioration in the ALS functional rating score (ALSFRS-R) by at least two points during the previous 18 months.
- Live within 120 minute drive from Sheffield

Exclusion criteria:

The main circumstances where patients or carers will be excluded are those in which individuals would be unable to use the telehealth system or give informed consent.

- Patients attend another MND care centre in the UK.
- Significant impairment in decision making capacity preventing informed consent by the subject due to a major mental disorder including fronto-temporal dementia.
- Patient unable to use the TiM system due to physical, intellectual or language difficulties and unwilling to permit carer to operate it on their behalf. Patients will be asked to complete two questions used within the TiM system, with, or without the help to their carer to verify their ability to use the system.
- ~~The patient has no eligible informal carer willing to participate in the trial~~ (V1.5)
- Insufficient mobile telephone reception in the patients' home to use the TiM system.
- Any other major impairment that may affect their ability to participate in the study

Carer inclusion criteria

- Age 18 years or older
- Person identified by the patient as the major provider of informal care (emotional and/or practical support) to the patient and provides more than one hour per week of unpaid care
- Carer willing to allow data they provide during the trial to be shared by the research team with their own doctor in the event of serious clinical need.

Carer exclusion criteria

- Significant decision making capacity preventing informed consent due to a major mental disorder.
- Carer unable to use the TiM system due to physical, intellectual or language difficulties. Carers will be asked to complete two questions used within the TiM system to verify their ability to use the system.

Hobson E, *et al*. *BMJ Open* 2019; 9:e028526. doi: 10.1136/bmjopen-2018-028526

- Inability to participate in the study due to other major physical or mental illness or language difficulties.
- Professional carers receiving direct payment for their services.

6e. Withdrawal

Participants will be followed up until the end of the study, death, or withdrawal. Those wishing to withdraw will be given the opportunity to speak to a member of the study team. Participants are free to withdraw from the intervention or study at any time. As a pilot study, importance of understanding reasons for withdrawal is recognised. This will be explained to the participants in the information leaflets. The importance of understanding reasons for withdrawal and the characteristics of these participants is recognised given the nature of the study. This will be explained to the participants in the information leaflets.

Withdrawal criteria

1. Patient request
2. Carer request
3. Patient loses capacity to continue to provide consent

If a Patient withdraws from the study arrangements will be made for the equipment to be collected or returned. Where appropriate participants will be invited to give the reasons for withdrawal.

Patients will also be given the option of:

1. Withdrawal from the intervention but remain within the study. Study data will only be collected at clinic visits at 3 and 6 months and six monthly until the end of the study.
2. Withdrawal from the study. Unless the participant objects, any data collected up to this point would be retained and used in study analysis. Participant agrees to allow contact to give safety and survival data.
3. Withdrawal from the study entirely. Unless the participant objects, any data collected up to this point would be retained and used in study analysis. If the participant does not wish to be contacted with regard to safety or survival data, no further contact with regard to this study will be made.

In the event that the patient dies or loses the capacity to provide consent they will be withdrawn from the trial but any data collected up to that point would be retained and used in study analysis. Carers would also be withdrawn at this point.

If the carer participant withdraws the patient can opt to continue to use the system or withdraw. If appropriate, carers will be invited to give reasons for withdrawal.

6f. Compliance

Steps have been taken to encourage compliance with the weekly schedule. Patients will receive regular messages on the system to invite them to complete a scheduled telehealth session. They will receive feedback on their compliance record and an

encouraging message at the start of each visit. Those who fail to complete the session within one day will be reminded by text and via the telehealth system. If after two weeks they do not enter data they will be contacted by the PI and offered more support and training. Compliance data will be analysed as part of the process evaluation.

Compliance with the patient reported outcome measures would be monitored by the CRF nurse. She will contact the participants to support data collection, identify and chase missing data and feedback to the PI. In the event of missing data the CRF nurse will telephone the patients/carer after two weeks. She will contact them a minimum of twice and a maximum of three times to chase the data. A contact log will be kept.

6g. Sample size

The study aims to recruit a total of 40 patients and their carers. 20 patients and their primary carer will be randomised to the intervention arm (a minimum of 6 months use of the TiM telehealth plus usual care) and 20 patents and their carer in the control arm (usual care).

Since the proposed trial is primarily an assessment of the acceptability of the intervention and the feasibility of a full trial, the proposed sample size is not based on standard statistical parameters such as a clinically relevant difference between groups. Instead, the sample size is justified on the grounds of quantifying patient variance (i.e. the standard deviation) in the proposed outcome measures (in particular quality of life measures) and on feasibility of the full trial, as follows:

- A sample size of 40 patients allows a standard deviation to be estimated to within a precision of ±20% of its true underlying value with 90% confidence. This estimate will be synthesised with standard deviations observed in other published studies (e.g. (41, 44-47) and on-going trials within SITraN (48, 49), to provide a robust estimate for use in the sample size calculation for the full trial.

- Given the rarity of MND, any definitive study will be infeasible if the required sample size is substantial. Assuming the upper limit for feasible UK study is around 200-300 patients in total, it follows that the full study would need powering to detect a standardised effect size of at least 0.4 SDs. This pilot trial will provide a preliminary assessment of whether the intervention might feasibly achieve this, and inform the choice of outcome measures for the proposed full study.

This sample size is also in keeping with the proposal of 12 evaluable patients per arm in a pilot study (after withdrawal or drop-out) (50).

6h. Blinding

The PI will not be blinded to the randomisation as they are responsible for training the participants to use the TiM system and any on-going technical or training requirements. The treating clinicians will not be blinded to the arm of the intervention as they are responsible for the clinical care of the patient and reviewing the data and the Shadow Monitoring System.

The PI will enter screening baseline data. Participant reported outcome measures after this will be collected by an independent research nurse from the Sheffield Teaching Hospitals Clinical Research Facility who will facilitate collection of these surveys by post or, if preferred by the patient, in person. They will enter the details into the study database.

Following the end of the trial and database lockdown the PI will analyse the two groups of data whilst remaining blinded to the allocation of the two groups. The STH CRF will hold the database code to identify the allocated groups.

The PI will conduct the qualitative interviews and collect the system use data and will not be blinded to these measures.

7. Study treatment

Standard clinical care – Intervention arm and Standard care arm

Usual clinical care will continue throughout for participants in both arms of the study. All participants will continue to be invited to the Sheffield MND Care Centre Clinic for routine review. They will be seen by their consultant neurologist and the MND multidisciplinary team, according to their routine two- to three-monthly schedule. All patients will have access to the MND telephone helpline provided by the Sheffield MND care team.

The TiM system - Intervention arm

Those in the intervention arm will use the TiM system, in addition to standard clinical care. All necessary hardware, software, data transfer and support costs for the TiM system will be met by Abbott Healthcare Products Ltd in collaboration with Carematix.

Patients will be provided with a TiM patient hub: a handheld, touch screen Samsung Galaxy tablet computer that communicates with the MND specialist nurse's TiM clinician system at the Sheffield MND Care Centre. Patients will be asked to use the system at least weekly. Each week the telehealth hub asks the patient a series of questions to detect common problems found in MND, such as worsening mobility, or swallow, symptoms of depression, anxiety, pain, saliva and spasms. Some of the questions closely match validated scoring scales (e.g. the ALS Revised Functional Rating Score (51) and the depression and anxiety short screen: PHQ-4) but others have been specifically designed by the clinical team to be used in the telehealth system. The TiM system also enquires about symptoms of respiratory insufficiency and infection, nutrition and social care. Patients using specialist equipment, such as non-invasive ventilation or gastrostomy tubes will additionally be asked to report problems related to the intervention. The TiM system can also weigh patients weekly and monitor patients' overnight oximetry using established telehealth monitors.

Hobson E, *et al. BMJ Open* 2019; 9:e028526. doi: 10.1136/bmjopen-2018-028526

Carers will also be asked to complete a weekly telehealth session in order to monitor their well being. The TiM system includes a carer strain screen and the PHQ-4 depression and anxiety screen. There is also the opportunity with the telehealth system for patients or carers to trigger an adhoc session if issues arise during the week about which they wish to inform the centre. Interspersed through the questions are educational messages and users have access to a bank of educational resources within the hub.

The patient and carer responses are transmitted (via an encrypted 3G mobile signal) to the Carematix server. The responses undergo immediate computational analysis, using pre-determined clinical algorithms, which assigns an alert level to each response. This limits the amount of nurse's time required to use the system. An automated acknowledgement is sent back to the user indicating whether to expect contact from the MND centre based on the results and the timescale for the response.

Each day the MND nurse will log into the TiM system and will be presented with the responses from all patients using the TiM system. They will be automatically alerted to any important changes. Urgent alerts include any new and severe symptom or any new problem that poses a major risk to the patient or carer (e.g. choking, falling, respiratory insufficiency). Routine alerts include any other deterioration in the patient's ALSFRS-R or any new symptom. An appropriately timely response will be made to each alert level. Patients will be reminded to seek urgent medical attention in an emergency.

The information on patient status may facilitate rescheduling of appointments according to patient need rather than the fixed intervals used at present (e.g. the appointment could be delayed if the patient is well, and the TiM system has activated no new alerts). As the feasibility and safety of the TiM system has not been previously evaluated, during the TiM trial patients will continue to attend routine clinic appointments and no patient will have their clinic delayed. The feasibility and safety of rescheduling appointments will be examined using a shadow monitoring protocol (detailed later).

Patients and carers will undergo a training session and a follow-up telephone call after two weeks. The TiM system has been designed to be user friendly and to encourage compliance with the weekly sessions. Face-to-face training with the hub system will be offered at the start of the intervention. Support will be available throughout the trial in the hub. Compliance will be monitored and should patients not complete the TiM system for three weeks in a row, contact will be made to offer more training or support.

The TiM system has been designed by the applicant in collaboration with her supervisors, the Sheffield MND team, Abbott Healthcare Products Ltd. and Carematix. Carematix have experience in delivering similar home telemonitoring systems in other diseases. In developing the system, expertise has also been sought from those developing telehealth services in other diseases in the University of Sheffield and NIHR CLARHC for South Yorkshire. These included the School of Health and Related Research (ScHARR) SMART consortium (Self Management supported by Assistive, Rehabilitation and Telecare technologies), NIHR CLAHRC SY Telehealth & Care Technologies (TaCT) for Long Term Conditions theme, and Devices for Dignity (52).

20

8. Data collection

8a. Quantitative data collection

Data collection will occur at baseline, three months and six months at the end of the study. Participant data will be completed using postal and telephone questionnaires to minimize patient burden and cost. The study will continue for a minimum of six months. Follow-up will continue until the last participant has used the system for six months. The maximum proposed follow-up will be 18 months.

Patient measures
Baseline measures:

- Age
- Gender
- Experience with technology (frequency of use of a computer, tablet or smart phone)
- Major health condition that could impact on the use of telehealth (including mood disorder, other symptomatic chronic disease)
- Medication

Outcome measures will be collected at 0, 3 and 6 months, then every six months until the end of the study and finally at the end of the study:

- Quality of life measures
    - ALSAQ-40 (an MND disease specific quality of life score (41))
    - SF-36-RAND
    - EQ-5D+D (EQ-5Q-3L with a dignity bolt-on)
- Clinical outcomes
    - ALSFRS-R (an MND disease specific functional rating score (51)
    - Pain score (modified Likert scale)
    - CSS-MND Saliva Severity Scale (designed for use with MND patients) plus global change scale
    - Hospital Anxiety and Depression Scale
- Health resource usage questionnaire
- Patient experience questionnaire

Hobson E, *et al. BMJ Open* 2019; 9:e028526. doi: 10.1136/bmjopen-2018-028526

Carer measures

Baseline measures:

- Age
- Gender
- Frequency of use of a computer, tablet or smart phone
- Major health that could impact on the use of telehealth
- Relationship to patient
- Number of hours spent per week providing care for patient

Outcome measures will be collected at 0, 3 and 6 months and at the end of the study:

- SF-36 RAND
- 12 item Zarit Burden Inventory (53)
- Hospital Anxiety and Depression Scale (54)
- Carer satisfaction questionnaire

Data will be collected to evaluate the conduct of the trial including:

- Participant compliance with the weekly telehealth session
- Rate of completion of outcome measures
- Rates of recruitment and withdrawal
- Participant actual and perceived time burden associated with the system
- Time spent by the MND nurse using TiM system and responding to alerts or queries generated by the system.

Hobson E, *et al. BMJ Open* 2019; 9:e028526. doi: 10.1136/bmjopen-2018-028526

8b. Qualitative sub-study

Intervention patient and carer interviews

Qualitative semi-structured interviews will be conducted with patients and carers in the intervention arm. Participants randomized into the intervention arm will be invited to take part in interviews. Baseline interviews will occur at one month after the intervention is started. A further interview will be conducted at 6 months. Six months is considered an appropriate timeframe for patients to become familiar with the intervention and its impact on quality of life.

Interviews will be conducted until data saturation is reached. The interviews will draw directly upon peoples' own experience and views, within the context of everyday lives to explore topics including

- Participants experience and expectations of technology
- Participants' expectations of telehealth services
- Barriers and aids to recruitment
- Compliance with the TiM system
- How the TiM system is used at home by patients and their carers
- The impact of using the TiM system on their lives and well-being
- The impact of education on their day-to-day lives
- The experiences of carers monitoring
- Whether the outcome measures used capture the changes in participants well being associated with using the TiM system.
- How the system would be used outside a trial

The early phase interview will explore participants' expectations of technology and the TiM system, the views on the system, their experiences of training and using the equipment. The later phase will explore further how the TiM system influenced their care and quality of life, mental well being as their condition changed. It will also identify barriers and facilitators to adoption of the TiM system.

The applicant will agree pre-defined topic schedules (see Appendix B) developed from the literature, expert consensus and discussion with the trial management group with supervision from Dr. Wendy Baird, an experienced qualitative researcher (School of Health and Related Research, Sheffield University). The PI will conduct interviews in the participants' home. The PI will conduct qualitative interviews until data saturation is reached (55). Interviews will be audio- recorded, transcribed verbatim and analysed with coding and retrieval of data supported by NVivo software.

Due to the nature of MND consideration will be given to participants' needs. The research team has experience in conducting qualitative interviews with patients and carers and these interviews will be conducted in a similar fashion. Often patients with MND prefer to be interviewed with their carer. This also aids communication where patients have speech difficulties and allows participants to support each other whilst discussing sensitive issues. Patients can use communication devices and all participants will be provided with a brief topic guide prior to the interview to facilitate participation for those with communication difficulties. Interviews will be limited to approximately one hour to reduce burden and fatigue. If participants prefer to be interviewed together carers will also be offered separate interviews where possible.

Field notes will also be collected by the PI during the face-to-face training using the TiM system to determine participants' early reactions to using the system and their needs for training.

Control group interviews

Following randomization, those patients and carers who are assigned the control arm will complete the baseline questionnaires. They will then have a short (15-20 minute) semi-structured interview with the PI. This will focus on their experiences and opinions of the baseline questionnaires. It will examine whether they were easy or difficult to complete, whether they were acceptable or caused distress to complete and which questions most reflected their condition and current quality of life.

The interviews, topic guides and analysis will be conducted in the same way as described in the previous section. Topic guides will not be provided before the interview but participants invited to submit any further comments to the research team either in writing or telephone following the interview. It is expected that patients and carers will be interviewed together. Interviews will continue until data saturation is reached or a maximum of 10 interviews conducted.

Staff interviews

At least five staff that care for the participants will undergo one-to-one semi structured interview by the PI during and at the end of the intervention. This will include the two responsible consultant clinicians (Dr Chris McDermott and Professor Pamela Shaw), at least one MND specialist nurse who has used the telehealth system and two members of the MND community team who have cared for participants. They will allow them to draw on their experiences of the TiM system in more depth. A staff information leaflet will be provided and written consent will be required prior to any interview.

Topics will include

- The day-to-day use of the TiM system
- The impact of the TiM system on clinical care of patients and carers
- The safety and accuracy of the system
- Barriers and aids to adoption of the TiM system.
- Views on amending the appointment schedule

These will be planned and conducted in the same manner as the participant interviews under the supervision of Dr Wendy Baird. An interview with the MND nurse and clinicians using the system will be scheduled early in the trial to capture any problems with training and set up of the system. At the end of the trial further interviews will be held with the MND team as described above.

Following the interviews a focus group with the clinical team will be held to draw together all the information gathered from the patient and staff interviews. It will be chaired by Dr Wendy Baird, independent qualitative researcher, transcribed and analysed by the PI under her supervision.

The qualitative findings will facilitate the exploration of any issues and challenges, which may arise from using TiM from the perspective of all stakeholder groups. The findings will enhance understanding of the feasibility of using TiM and assist with the interpretation of the clinical data from the perspective of patients and clinicians.

8c. Shadow monitoring protocol

In order to determine the safety of a remote monitoring system that may enable clinicians to make decisions regarding a patient's management the trial will also collect data on clinicians' opinion on the accuracy of the data displayed by the TiM system. This is referred to as the shadow monitoring protocol.

Prior to each patient's face-to-face visit (depending on their appointment schedule) the treating MND doctor will be asked to conduct a remote assessment of the patient by reviewing the TiM system clinical information. They will be asked to indicate, given the information provided by the TiM system, whether they would change their patient's appointment. The patient would attend the appointment as scheduled and after the appointment the clinician would be asked whether the appointment schedule time was correct. They would also be asked to indicate whether they felt that the information displayed on the TiM system was a safe and accurate reflection of the patient's condition and whether it influenced their clinic visit. Clinicians will also indicate whether the TiM system had affected the consultation. They will also report any adverse events identified. Should patients be unable to travel to clinic they will be offered a telephone consultation at the usual scheduled time. The same Shadow Monitoring questions and need to report adverse events will apply.

The results of this shadow monitoring will be triangulated with the qualitative sub-study and will influence the later interview topic guide.

8d Process evaluation

Data regarding the TiM system use by patients, carers and staff will be will be collected in order to understand how the system could be used in the NHS MND care process. It will also collect data regarding the extra time and resources required to manage the problems generated by the TiM system. It will be triangulated with data gained from the qualitative sub-study, adverse event log and shadow monitoring protocol.

9. Analysis

The PI will conduct analysis with regular supervision from the TMG.

9a. Feasibility and quantitative analysis

The feasibility of a full trial will be determined by analysis of

- Recruitment rates
- Retention rates
- Compliance rates
- Sample size calculations as detailed above

The safety, acceptability and feasibility of use of the TiM system

- Incidence of adverse events (clinical and related to the TiM system functionality)
- Information collected using the Shadow Monitoring process
- Qualitative data analysis

The PI and the CRF study nurse will be responsible for chasing missing data. The CRF nurse is responsible for chasing the questionnaire data and will telephone the patients a minimum of once and maximum of twice to chase unreturned questionnaires or clarify missing data within the questionnaire packs. They will report monthly to the PI. For the main outcome measures, (SF-36 and ALSAQ-40) protocols are provided for managing missing data if necessary. Participants who withdraw will be encouraged to continue to be followed up and reasons for withdrawal ascertained where possible. In the proposed larger, efficacy trial intention to treat analysis will be adopted.

Quantitative analysis will be undertaken in a similar manner for all endpoints. The change from baseline at each time point will be analysed using analysis of covariance in which the covariates are treatment group and the baseline value. For instance, the change in ALSFRS-r at six months will be analysed with treatment group and baseline ALSFRS-r as covariates. The mean (standard deviation) change in each group, the difference between groups and its associated 95% confidence interval will be reported. No formal hypothesis testing will be undertaken for this pilot study.

Hobson E, *et al*. *BMJ Open* 2019; 9:e028526. doi: 10.1136/bmjopen-2018-028526

9b. Qualitative analysis

Data from the interviews will be recorded, transcribed and undergo Framework analysis (56). Although Framework analysis was developed for applied policy it has proved useful in applied health research. Analysis will be ongoing and iterative involving concurrent data collection and analysis, with systematics efforts to check and refine developing categories of data. Themes and hypothesis identified in the early phases of data collection will inform the areas of investigation in later interviews. Regular meetings with supervisors will review the data analysis, explore respondents' underlying reasoning, discuss deviant cases and reach agreement on recurrent themes and findings. The PI's field notes and reflexive diary will also be reviewed and used to inform the analysis of qualitative data. Dr Wendy Baird, an independent, experienced qualitative research, will supervise this stage of the work.

Results from the qualitative analysis will be triangulated, for example, to explore the reasons why problems with the trial methodology or TiM system have occurred. Both themes and anonymous verbatim comments will be published to demonstrate the findings.

Hobson E, *et al. BMJ Open* 2019; 9:e028526. doi: 10.1136/bmjopen-2018-028526

10. Data entry, security and confidentiality

Clinical quantitative data input will be the responsibility of the PI (baseline) and CRF study nurses (months 3 and 6, and at the end of the study). Data quality will be the responsibility of CRF nurses and PI who will report back to the TMC and TSC. The qualitative data and system usage data will be the responsibility of the PI. Data (including audio-recordings) will be collected and retained in accordance with the Data Protection Act 1998 and Caldicott Principles. Anonymised study data will be entered onto a validated database system designed to an agreed specification between the PI and Sheffield CTRU and securely stored on the SU intranet. The PI and the CRF research nurses will have access to data on the database through the use of usernames and encrypted passwords. Study documents will be retained in a secure location during and after the study has finished.

All source documents will be retained for a period of at least 5 years following the end of the study, as per the CTRU SOP. Where study related information is documented in medical records those records will be retained for at least 5 years after the last patient last visit

The data provided through the TiM system will be collected using a secure web-app accessed by the participants by a unique username and password. It will be stored on a secure server that will be available through a web-portal hosted by Carematix to the clinical team using secure usernames and password.

For the purposes of the trial each participant will be given a unique TiM system code. This will allow all data to be relayed through the web-app without any associated patient identifiable features. This code will be held separately and stored securely on the STH intranet to allow individual identification by the MND care team. The clinician will display only the anonymous code. This will be accessed through a secure portal with usernames and passwords. No identifiable information will be stored on the patient hub or on the TiM server. The technology providers will have no access to patient identifiable information. Any technology problems will be dealt with by the research team and participants will have no contact with the technology providers.

The system has a full electronic audit trail and will be regularly backed up and will be held in a way that conforms to STH information governance procedures.

Access to source data

Monitoring and audit by the relevant health authorities will be permitted by the sponsor. These include the Research Ethics Committee and local R&D departments. The sponsor will be allowed to monitor and audit the study at each site and be allowed access to source data and documents for these purposes.

Hobson E, *et al. BMJ Open* 2019; 9:e028526. doi: 10.1136/bmjopen-2018-028526

11. Safety and safety assessments

We do not envisage any serious safety or adverse events associated with the intervention. The system does not give individual advice to a patient or recommend change in management without input from a clinician. The trial protocol requires patients to continue with their usual care including planned outpatient appointments and the Shadow Monitoring Protocol will evaluate whether the data provided by the TiM system is felt to accurately reflect the patients' clinical condition. The responsible clinician who is a consultant neurologist with specialist experience in MND and research will continue to review the patient on a regular basis (unless the patient is unable to attend clinic) and will have overall responsibility for their care throughout the trial. The specialist MND nurses using the TiM system have extensive experience in managing patients via the existing MND helpline.

The database will automatically alert the trial manager to any carer scoring 11 or more of the Hospital Anxiety and Depression score collected as part of the outcome measures. This will allow the trial manger to identify those carers who may require further support.

Adverse Event Reporting
All adverse events will be reported in accordance with the Sheffield CTRU Adverse Event and Serious Adverse Events SOP.

Participants will be monitored for adverse clinical events and efforts will be made to ascertain whether the TiM system influenced the event or could have predicted the event. These include unplanned admissions and deaths. Non-clinical events relating to the use of the telehealth hub will recorded e.g. failure to record or deliver information to and from the clinical interface.

In research other than CTIMPs an adverse event is defined as: is any unfavorable and unintended sign (including an abnormal laboratory finding), symptom, or disease having been absent at baseline, or, if present at baseline, appears to worsen AND is temporally associated with medical treatment or procedure, **REGARDLESS** of the attribution (i.e., relationship of event to medical treatment or procedure).

Serious Adverse Event (SAE)
In research other than CTIMPs, the National Research Ethics Service defines a Serious Adverse Event (SAE) is defined as an untoward occurrence that:
(a) results in death;
(b) is life-threatening*;
(c) requires hospitalization** or prolongation of existing hospitalization**;
(d) results in persistent or significant disability or incapacity;
(e) consists of a congenital anomaly or birth defect; or
(f) is otherwise considered medically significant by the investigator.

*"life-threatening" in the definition of "serious" refers to an event in which the patient was at risk of death at the time of the event; it does not refer to an event which hypothetically might have caused death if it were more severe. **Hospitalisation is defined as an inpatient admission, regardless of length of stay, even if the

hospitalisation is a precautionary measure for continued observation. Hospitalisations for a pre-existing condition, including elective procedures that have not worsened, do not constitute an SAE.

Adverse event exclusions
The only adverse event that will be excluded is:
1. Standard or expected disease progression.

Adverse event inclusions

All serious adverse events will be reported. These include deaths of participants and emergency admissions. We will attempt to determine whether the use of the TiM system contributed to the event, in particular whether there was any delay in seeking help due to the use of the system.

Assessment of Adverse Events
The following criteria will be used when assessing adverse events: Intensity (severity):
Mild - does not interfere with routine activities
Moderate - interferes with routine activities
Severe - impossible to perform routine activities

Relationship to the study treatment:
Unrelated - There is no evidence of any causal relationship. N.B. An alternative cause for the AE should be given
Unlikely - There is little evidence to suggest there is a causal relationship. There is another reasonable explanation for the event (e.g. the participant's clinical condition, other concomitant treatment).
Possible - There is some evidence to suggest a causal relationship. However, the influence of other factors may have contributed to the event (e.g. the participant's clinical condition, other concomitant treatments).
Probable - There is evidence to suggest a causal relationship and the influence of other factors is unlikely.
Definite - There is clear evidence to suggest a causal relationship and other possible contributing factors can be ruled out.
Not assessable - There is insufficient or contradictory information which cannot be supplemented or verified

Reporting procedures

All study participants will be encouraged to contact and inform their site research team if they experience any new medical problem or are admitted to hospital. Those that are not picked up through general contact will be identified at their routine 2-3 monthly outpatient appointments either in person or by telephone as part of the Shadow Monitoring Protocol. The patients' consultant neurologist (Dr. Chris McDermott or Professor Dame Pamela Shaw) will enquire about any adverse events since the previous visit and record these on the adverse event paper CRF and database. For any Serious Adverse Events an SAE paper CRF and database entry will be completed. The PI and consultant neurologist will assess the event and the CRF will be kept in the site file. Serious adverse events will be reported to the TSC, TMG and the sponsor if deemed by

Hobson E, *et al. BMJ Open* 2019; 9:e028526. doi: 10.1136/bmjopen-2018-028526

either to be related to the trial. Reports of related and unexpected SAEs will be submitted to the ethics committee within 15 days of the chief investigator becoming aware of the event. This will use the National Research Ethics Service Report of Serious Adverse Event form. Information will also be included in the routine progress reports to the sponsor and ethics committee. Routine safety data and all SAEs that the TMG or TSC deems to be related to the trial will also be reported to the technology provider in the same manner.

Any suspected adverse drug reaction would be assessed and reported to the MHRA as part of clinicians' routine pharmacovigilance responsibilities using the Yellow Card Scheme. The technology provider, Abbott Healthcare Products Ltd. manufactures a number of drugs (listed in Appendix A). Any suspected adverse drug reaction involving an Abbott Healthcare Products Ltd. drug would also be reported to the manufacturer in the same manner, within 24 hours of receipt by the CI or the next working day for reports received out of hours. Such reports should be sent to ukpharmacovigilance@abbott.com. On a monthly basis the PI will send a reconciliation list of all the reports sent to the Abbott Pharmacovigilance Department within that month to ensure that all the appropriate information has been exchanged. Should any discrepancies arise, both parties will immediately seek to resolve them.

Hobson E, *et al. BMJ Open* 2019; 9:e028526. doi: 10.1136/bmjopen-2018-028526

12. Ethical considerations

The study will be conducted in accordance to Good Clinical Practice Guidelines and subject to Research Ethics Committee favourable opinion. The study received a favorable approval from an independent panel representing the NIHR, which funds Dr. Esther Hobson's NIHR Doctoral Fellowship Award.

The study has approval from the Sheffield Teaching Hospitals NHS Foundation Trust's Research and Development department. It has also received favourable review from Dr. Mike Bradburn, study statistician at ScHARR, Dr. Cindy Cooper, director of CTRU and ScHARR, and Dr. Wendy Baird of the Yorkshire and Humber Research and Design service, Professor Alicia O'Cathain, Professor of Health Services Research, ScHARR and Professor Dame Pamela Shaw, SITraN. The application will be submitted through the IRAS central allocation system. The approval letter from the ethics committee and copy of approved patient information leaflet, consent forms, CRF's and questionnaires will be present in the site files before initiation of the study and patient recruitment.

It is recognized that patients with MND may be frail and nearing at the end stages of their lives. The research team has extensive experience in conducting clinical trials in this population. The study design has attempted to limit the burden imposed by the study by avoiding unnecessary study visits (by combining them with scheduled visits), collecting data in the participants' homes at their convenience and limiting the study procedures to the minimum necessary. The intervention has been designed in collaboration with patients and carers to maximize ease of use and minimize impact on participants' lives. It is appreciated that there are a number of questionnaires that require completion. Given one aim of the study is to determine the most appropriate outcome measures to evaluate efficacy of the TiM system there are more questions than would be used in a large scale trial. These have been reviewed by the Sheffield MND Research Advisory Group (the local PPI group) and the lay members of the TSC (David Stelmach) and TMG (Anne Quinn) to ensure acceptability. Participants will be supported by the CRF nurse to complete these at their convenience in a manner selected by the participant (either by post, telephone or in person).

There are other clinical studies ongoing in the Sheffield MND care centre. Involvement in other studies would not preclude patients from entering this study. Consideration of the burden involved in the study, potential impact on the outcome of the study and the patients' expressed priorities will be considered before patients are approached to be involved. If involvement in this study excludes patients from entering another clinical trial patients will be given the option to withdraw from this study.

The potential conflict of interest between the role of the clinical team in caring for patients and their role as researchers is recognized. The study design has considered the impact of this conflict on the participants choices and also any potential bias. Whilst PI is a doctor working within the MND team she is a specialty training registrar and overall responsibility for the patients' clinical care will remain with the consultant neurologist rather than the PI. Whilst she may have already cared for potential participants, following an invite to participate in the trial will no longer see these patients in their routine clinical appointments and her role will be as a researcher.

Hobson E, *et al. BMJ Open* 2019; 9:e028526. doi: 10.1136/bmjopen-2018-028526

The dual role of the PI as a doctor and researcher has been previously evaluated. The professional background of a doctor may actually aid the building of a research relationship, allow patients to be more open and comfortable with discussing their health with someone who they already trust (56). In order successfully identify any potential bias the purpose of the research and nature of the PI's role will be emphasized throughout the study, the PI will keep a reflexive diary and field notes and identify any potential bias. Where bias is most likely, i.e. in the collection of outcome measures steps have been taken to limit this: the quantitative outcome measures will be collected by an independent study nurse and the qualitative interview structure and topic guides have been planned with supervision from an independent researcher Dr. Wendy Baird. The PI will be supervised, as part of her PhD by independent academics: Dr. Cindy Cooper and Dr. Mike Bradburn (focusing mainly on the trial methodology and conduct, and quantative data analysis), Dr. Wendy Baird and Professor Sue Mawson (qualitative work and service evaluation). If, during the research, participants identify any medical problems, the PI has a duty of care and will make arrangements to deal with these problems. This might involve signposting them to appropriate services or liaising with the clinical team. A log of these activities will be kept and reviewed by the TMG.

When the participants have prior knowledge of the researcher they may feel a sense of duty and feel pressurized to participate (57). Ground rules, informed consent, confidentiality, freedom to stop and what to expect will be discussed with all participants. Participants will be approached by letter and they will be required to contact the study team allowing them to consider the trial in detail first. It will be explained to the patient (both verbally and in the information leaflets and consent forms) that participation is voluntary and will not affect their ongoing care. The information leaflet differentiates the research process and their usual care. It will be made clear, particularly in the interview phase that the PI's role is as a researcher and the aim of the study is to critically analyse service provision and that whilst comments, particular negative comments, will be passed back to the care team they will treated with confidence and respect.

Whilst the carer participant is not a patient of the Sheffield MND team the research team have a duty of care to the carer. There may be circumstances where the carer may disclose information that requires medical care, for example disclosing symptoms of depression or anxiety. At the start of the trial the carer participants' GP will be informed of the trial. In the event of a serious risk being identified the research team will discuss this in confidence with the carer and make arrangements to resolve the problem. This might include referral to his or her own GP or other health professional. Carer participants will be informed of these procedures in the Carer information leaflet and consent form. Confidentiality will be maintained in accordance with the General Medical Council's guidance on Confidentiality (58).

Upon publication of the qualitative interviews it may be possible to identify participants' comments although this will be avoided if possible. This is explained to participants in the Interview Information Leaflets and on the consent form.

Hobson E, *et al. BMJ Open* 2019; 9:e028526. doi: 10.1136/bmjopen-2018-028526

13. Finance and indemnity

The trial has been financed through an NIHR doctoral fellowship grant and details have been drawn up in a separate agreement.

This is an NHS sponsored study. If there is negligent harm during the clinical trial when the NHS body owes a duty of care to the person harmed, NHS indemnity will cover NHS staff, medical academic staff with honorary contracts and those conducting the trial.

The University of Sheffield has in place insurance against liabilities for which it may be legally liable and this cover includes any such liabilities arising out of this clinical trial.

14. Reporting and dissemination

Results of the study will be disseminated in peer reviewed scientific journals and clinical and academic conferences. Details of the study will also be made available on the SITraN and ScHARR websites, blogs and social media and through local MND groups. Summaries of the research will be updated periodically on the SITraN website to inform readers of the ongoing progress. Following publication contact with other UK MND care centres will be made to disseminate the findings and assess buy-in potential for a full study if this is appropriate.

15. References

1. The neurological alliance. Neuro numbers. A brief review of the numbers of people in the UK with a neurological condition.
2. Whitehead B, O'Brien MR, Jack BA, Mitchell D. Experiences of dying, death and bereavement in motor neurone disease: A qualitative study. Palliat Med. 2012;26(4):368-78.
3. O'Brien MR, Whitehead B, Jack BA, Mitchell D. The need for support services for family carers of people with motor neurone disease (MND): views of curent and former family caregivers a qualitative sutdy. Disabil Rehabil. 2012;34(3).
4. Miller RG, Mitchell JD, Moore DH. Riluzole for amyotrophic lateral sclerosis (ALS)/motor neuron disease (MND). Cochrane Database Syst Rev. 2012;3:CD001447.
5. Bourke SC, Tomlinson M, Williams TL, Bullock RE, Shaw PJ, Gibson GJ. Effects of non-invasive ventilation on survival and quality of life in patients with amyotrophic lateral sclerosis: a randomised controlled trial. The Lancet Neurology. 2006;5(2):140-7.
6. Aridegbe T, Kandler R, Walters SJ, Walsh T, Shaw PJ, McDermott CJ. The natural history of motor neuron disease: Assessing the impact of specialist care. Amyotroph Lateral Scler. 2012.
7. Chio A, Buffa C, Mutani R, Bora G, and the PARALS. Positive effects of tertiary centres for amyotrophic lateral sclerosis on outcome and use of hospital facilities. J Neurol Neurosurg Psychiatry. 2006;77(8):948-50.
8. Johnston B, Kidd L, Wengstrom Y, Kearney N. An evaluation of the use of Telehealth within palliative care settings across Scotland. Palliat Med. 2012;26(2):152-61.
9. Foley G, Timonen V, Hardiman O. Experience of Services as a Key Outcome in Amyotrophic Lateral Sclerosis (ALS) Care: The Case for a Better Understanding of Patient Experiences. Am J Hosp Palliat Care. 2012;29(5):362-7.
10. Motor Neurone Disease Association UK. MND statistics.
11. Baxter SK, Baird WO, Thompson S, Bianchi SM, Walters SJ, Lee E, et al. The initiation of non-invasive ventilation for patients with motor neuron disease: Patient and carer perceptions of obstacles and outcomes. Amyotroph Lateral Scler. 2012.
12. Foley G, Timonen V, Hardiman O. Patients' perceptions of services and preferences for care in amyotrophic lateral sclerosis: a review. Amyotroph Lateral Scler. 2012;13(1):11-24.
13. The use of non-invasive ventilation at end of life in patients with motor neurone disease. Submitted for publication. 2012.
14. Hecht MJ, Graesel E, Tigges S, Hillemacher T, Winterholler M, Hilz MJ, et al. Burden of care in amyotrophic lateral sclerosis. Palliat Med. 2003;17(4):327-33.
15. Peters M, Fitzpatrick R, Doll H, Playford ED, Jenkinson C. The impact of perceived lack of support provided by health and social care services to caregivers of people with motor neuron disease. Amyotroph Lateral Scler. 2012;13(2):223-8.
16. van Teijlingen ER, Friend E, Kamal AD. Service use and needs of people with motor neurone disease and their carers in Scotland. Health Soc Care Community. 2001;9(6):397-403.
17. McLean S, Chandler D, Nurmatov U, Liu J, Pagliari C, Car J, et al. Telehealthcare for asthma: a Cochrane review. CMAJ : Canadian Medical Association journal. 2011;183(11):E733-42.

18. McLean S, Nurmatov U, Liu JL, Pagliari C, Car J, Sheikh A. Telehealthcare for chronic obstructive pulmonary disease. Cochrane Database of Systematic Reviews (Online). 2011(7):CD007718.
19. Chua R, Craig J, Wootton R, Patterson V. Randomised controlled trial of telemedicine for new neurological outpatient referrals. 2001;71(1):63-6.
20. Steventon A, Bardsley M, Billings J, Dixon J, Doll H, Hirani S, et al. Effect of telehealth on use of secondary care and mortality: findings from the Whole System Demonstrator cluster randomised trial. Br Med J. 2012;344.
21. Corben S, Rosen R. Self-management for Long-term Conditions. Patients' perpective on the ways ahead. In: Conditions TKsFrML-t, editor. 2005.
22. 3millionlives Campaign. Available from: http://3millionlives.co.uk.
23. Vitacca M, Bazza A, Bianchi L, Gilè S, Assoni G, Porta R, et al. Tele-assistance in chronic respiratory failure: patients characterization and staff workload of 5-year activity. Telemedicine Journal and e-health : the official journal of the American Telemedicine Association. 2010;16(3):299-305.
24. Vitacca M, Comini L, Tentorio M, Assoni G, Trainini D, Fiorenza D, et al. A pilot trial of telemedicine-assisted, integrated care for patients with advanced amyotrophic lateral sclerosis and their caregivers. J Telemed Telecare. 2010;16(2):83-8.
25. Vitacca M, Bianchi L, Guerra A, Fracchia C, Spanevello A, Balbi B, et al. Tele-assistance in chronic respiratory failure patients: a randomised clinical trial. The European Respiratory Journal. 2009;33(2):411-8.
26. Vitacca M, Comini L, Assoni G, Fiorenza D, Gilè S, Bernocchi P, et al. Tele-assistance in patients with amyotrophic lateral sclerosis: long term activity and costs. Disability and rehabilitation Assistive technology. 2012.
27. Pinto A, Almeida JP, Pinto S, Pereira J, Oliveira AG, de Carvalho M. Home telemonitoring of non-invasive ventilation decreases healthcare utilisation in a prospective controlled trial of patients with amyotrophic lateral sclerosis. J Neurol Neurosurg Psychiatry. 2010;81(11):1238-42.
28. de Almeida JPL, Pinto AC, Pinto S, Ohana B, de Carvalho M. Economic cost of home-telemonitoring care for BiPAP-assisted ALS individuals. Amyotrophic lateral sclerosis : official publication of the World Federation of Neurology Research Group on Motor Neuron Diseases. 2012;13:533-7.
29. Sweeney V. Pilot scheme allowed Alan to be nursed at home. The Inverness Courier. 2009 24 Feb, 2009.
30. Nijeweme-d'Hollosy WO, Janssen EPF, Huis in 't Veld RMHA, Spoelstra J, Vollenbroek-Hutten MMR, Hermens HJ. Tele-treatment of patients with amyotrophic lateral sclerosis (ALS). J Telemed Telecare. 2006;12 Suppl 1:31-4.
31. Montes J, Levy G, Albert S, Kaufmann P, Buchsbaum R, Gordon PH, et al. Development and evaluation of a self-administered version of the ALSFRS-R. Neurology. 2006;67(7):1294-6.
32. Maier A, Holm T, Wicks P, Steinfurth L, Linke P, Munch C, et al. Online assessment of ALS functional rating scale compares well to in-clinic evaluation: a prospective trial. Amyotroph Lateral Scler. 2012;13(2):210-6.
33. Rafiq MK, Proctor AR, McDermott CJ, Shaw PJ. Respiratory management of motor neurone disease: a review of current practice and new developments. Pract Neurol. 2012;12(3):166-76.
34. Department of Health U. Supporting People with Long Term Conditions: An NHS and Social Care Model to supprt local innovation and integration. 2005.

35. McCall K, Keen J, Farrer K, Maguire R, McCann L, Johnston B, et al. Perceptions of the use of a remote monitoring system in patients receiving palliative care at home. Int J Palliat Nurs. 2008;14(9):426-31.
36. McCann L, Maguire R, Miller M, Kearney N. Patients's perceptions and experiences of using a mobile phone-based advanced symptom management system (ASyMS ©) to monitor and manage chemotherapy related toxicity. Eur J Cancer Care (Engl). 2009;18(2):156-64.
37. Kofoed S, Breen J, Gough K, Aranda S. Benefits of remote real-time side-effect monitoring systems for patients receiving cancer treatment. Oncology Reviews. 2012;6(7):1-13.
38. Bower P, Kennedy A, Reeves D, Rogers A, Blakeman T, Chew-Graham C, et al. A cluster randomised controlled trial of the clinical and cost-effectiveness of a 'whole systems' model of self-management support for the management of long- term conditions in primary care: trial protocol. Implement Sci. 2012;7:7.
39. Palmer R, Enderby P, Cooper C, Latimer N, Julious S, Paterson G, et al. Computer therapy compared with usual care for people with long-standing aphasia poststroke: a pilot randomized controlled trial. Stroke. 2012;43(7):1904-11.
40. Domingo M, Lupon J, Gonzalez B, Crespo E, Lopez R, Ramos A, et al. [Noninvasive remote telemonitoring for ambulatory patients with heart failure: effect on number of hospitalizations, days in hospital, and quality of life. CARME (CAtalan Remote Management Evaluation) study]. Rev Esp Cardiol. 2011;64(4):277-85.
41. Jenkinson C, Fitzpatrick R, Brennan C, Swash M. Evidence for the validity and reliability of the ALS assessment questionnaire: the ALSAQ-40. Amyotrophic lateral sclerosis and other motor neuron disorders : official publication of the World Federation of Neurology, Research Group on Motor Neuron Diseases. 1999;1(1):33-40.
42. Moher D, Hopewell S, Schulz KF, Montori V, Gotzsche PC, Devereaux PJ, et al. CONSORT 2010 explanation and elaboration: updated guidelines for reporting parallel group randomised trials. BMJ. 2010;340:c869.
43. Schulz KF, Altman DG, Moher D. CONSORT 2010 statement: updated guidelines for reporting parallel group randomised trials. BMJ. 2010;340:c332.
44. Jenkinson C, Peto V, Jones G, Fitzpatrick R. Interpreting change scores on the Amyotrophic Lateral Sclerosis Assessment Questionnaire (ALSAQ-40). Clin Rehabil. 2003;17(4):380-5.
45. Jenkinson C, Fitzpatrick R, Swash M, Jones G. Comparison of the 40-item Amyotrophic Lateral Sclerosis Assessment Questionnaire (ALSAQ-40) with a short-form five-item version (ALSAQ-5) in a longitudinal survey. Clin Rehabil. 2007;21(3):266-72.
46. Epton J, Harris R, Jenkinson C. Quality of life in amyotrophic lateral sclerosis/motor neuron disease: a structured review. Amyotroph Lateral Scler. 2009;10(1):15-26.
47. Jenkinson C, Hobart J, Chandola T, Fitzpatrick R, Peto V, Swash M. Use of the short form health survey (SF-36) in patients with amyotrophic lateral sclerosis: tests of data quality, score reliability, response rate and scaling assumptions. J Neurol. 2002;249(2):178-83.
48. McDermott CJ, Maguire C, Cooper CL, Ackroyd R, Baird WO, Baudouin S, et al. Protocol for diaphragm pacing in patients with respiratory muscle weakness due to motor neurone disease (DiPALS): a randomised controlled trial. BMC Neurol. 2012;12:74.

49. Evaluation of the impact of a CoughAssist® mechanical in-exsufflator (MI-E) device on morbidity, quality of life and survival in patients with motor neurone disease (MND) using non-invasive ventilation (NIV) [Internet].
50. Julious S. Sample size of 12 per group rule of thumb for a pilot study. Pharm Stat. 2005;4(4):287-91.
51. Cedarbaum JM, Stambler N, Malta E, Fuller C, Hilt D, Thurmond B, et al. The ALSFRS-R: a revised ALS functional rating scale that incorporates assessments of respiratory function. BDNF ALS Study Group (Phase III). J Neurol Sci. 1999;169(1-2):13-21.
52. NIHR CLAHRC South Yorkshire. Telehealth & Care Technologies (TaCT) for Long Term Conditions 2012. Available from: http://clahrc-sy.nihr.ac.uk/theme-tact-introduction.html.
53. O'Rourke N, Tuokko HA. Psychometric properties of an abridged version of The Zarit Burden Interview within a representative Canadian caregiver sample. Gerontologist. 2003;43(1):121-7.
54. Zigmond AS, Snaith RP. The hospital anxiety and depression scale. Acta Psychiatr Scand. 1983;67(6):361-70.
55. Francis JJ, Johnston M, Robertson C, Glidewell L, Entwistle V, Eccles MP, et al. What is an adequate sample size? Operationalising data saturation for theory-based interview studies. Psychology & health. 2010;25(10):1229-45.
56. Hoddinott P, Pill R. Qualitative research interviewing by general practitioners. A personal view of the opportunities and pitfalls. Fam Pract. 1997;14(4):307-12.
57. Richards HM, Schwartz LJ. Ethics of qualitative research: are there special issues for health services research? Fam Pract. 2002;19(2):135-9.
58. (UK) GMC. Confidentiality. 2009.

Hobson E, *et al. BMJ Open* 2019; 9:e028526. doi: 10.1136/bmjopen-2018-028526

Appendix 1: Drugs produced by Abbott Healthcare Ltd.

Fenofibrate
Pancreatin
Moxonidine
Estradiol/dydrogesterone
Mebeverine
Betahistine
Fluvoxamine maleate
Lactulose
Estradiol, oral applications
Influenza virus vaccine
Eprosartan mesylate
Ibuprofen
Flurbiprofen
Propafenone
Clarithromycin
Verapamil

Hobson E, *et al. BMJ Open* 2019; 9:e028526. doi: 10.1136/bmjopen-2018-028526

Appendix 2: CONSORT flow diagram

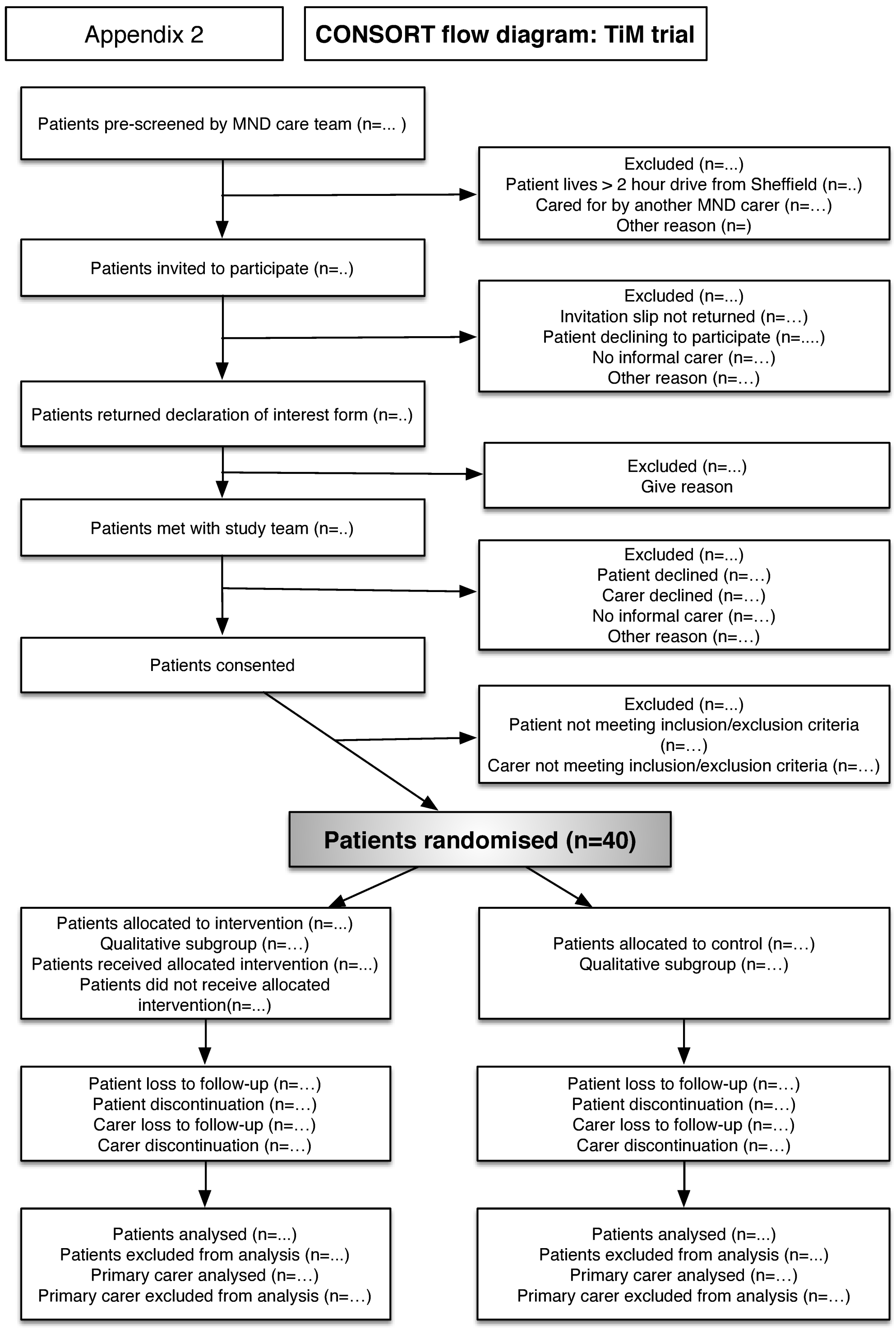

Hobson E, *et al. BMJ Open* 2019; 9:e028526. doi: 10.1136/bmjopen-2018-028526

Appendix 3 Qualitative interview topic guides

Baseline interview (control group)

"I'd just like to reiterate that everything you say in the interview is confidential to me and the research team. If anything you say is used in a publication then it will be anonymous. I'll be recording the interview to make sure I've don't miss anything important. It will take about 15 minutes but we can stop if you wish. If you have any questions I can answer them now or at the end of the interview. There aren't any right or wrong answers - I'm simply interested in your experience and your views. I'd like to know how you found filling in the questionnaire booklet. The questions are designed to understand more about your condition and experiences of MND."

For patients:

- Some are asking quite personal questions. How do you feel about that?
- Do you think the questionnaires asked questions about your life with MND?
- Did you find any of the questionnaires confusing?
- Did you find any of the questionnaires upsetting?
- What would you think about filling in these questionnaires again?
- If so, how would you fill them in?
- If you needed help, how would you fill in your questionnaire?

For carers

- Did you find any of the questionnaires confusing?
- Did you find any of the questionnaires upsetting?
- Some are asking quite personal questions. How do you feel about that?
- What would you think about filling in these questionnaires again?

Hobson E, *et al. BMJ Open* 2019; 9:e028526. doi: 10.1136/bmjopen-2018-028526

Intervention group

Interviews will be conducted at 1 and 6 months. Early interview results will guide later interviews. Participants will be provided with the topic guide questions prior to the interview in order for them to communicate their answers easily. The main focus of the interviews is on:

- Participants experience and expectations of technology
- Participants' expectations of telehealth services
- Barriers and aids to recruitment and compliance with the TiM system
- How the TiM system is used at home by patients and their carers
- The impact of using the TiM system on their lives and well-being
- The impact of education on their day-to-day lives
- The experiences of carers monitoring
- Whether the outcome measures used capture the changes in participants well being associated with using the TiM system.
- How the system would be used outside a trial

1-month interview (intervention group)

"I'd just like to reiterate that everything you say in the interview is confidential to me and the research team. If anything you say is used in a publication then it will be anonymous. I'll be recording the interview to make sure I've don't miss anything important. It will take about an hour but we can stop at any time if you wish. If you have any questions I can answer them now or at the end of the interview. There aren't any right or wrong answers. I'd like to here about your experiences of starting using the TiM system and of MND care. You remember you received some questions in the post, we'll be going over those subjects again today"

Previous experiences in MND care

- Can you tell me a little about how you came to get the diagnosis of MND?
- What have your experiences been since then?
- Can you tell me about your last MND hospital clinic visit?
- Have you used the MND helpline?
- How do you manage if you have a question or problem?
- What would you say you are most worried about?
- How do you think your MND team have helped you?
- How do you think your care could be better?
- What problems do you think have been most troublesome?
- How much do you know about MND?

For carers

- How do you get the support you need as a carer?
- How do you find the help the MND team gives?
- How do you think your care could be better?
- What problems do you think have been most troublesome?
- How much do you know about MND?

Expectations of the TiM system

- Before the start of the study what technology did you use?
- What did you expect the TiM system would be like?
- Is there anything you would have hoped it would have?
- Is there anything that worried you about it?

Experiences of training and starting to use the TiM system

- What did you think when you first saw it?
- How did you find the training?
- Do you remember what it was like using it for the first time?

Barriers and facilitators to using the TiM system

- Is there anything things you like about it?
- Is there anything you don't like?
- Has it worked every time as you expected?
- Do you think you will continue to use it regularly? Why?
- What have you told your friends about it?

For the carer

- How have you found using the TiM system?
- What was it like using it for the first time?
- How have you found the questions?

Hobson E, *et al. BMJ Open* 2019; 9:e028526. doi: 10.1136/bmjopen-2018-028526

6-month interview (intervention group)

"I'd just like to reiterate that everything you say in the interview is confidential to me and the research team. If anything you say is used in a publication then it will be anonymous. I'll be recording the interview to make sure I've don't miss anything important. It will take about an hour but we can stop at any time if you wish. If you have any questions I can answer them now or at the end of the interview. There aren't any right or wrong answers. I'd like to here about your experiences of using the TiM system and how it has affected your life and your MND care. You remember you received some questions in the post, we'll be going over those subjects again today"

For patients:

- How have you found using the TiM system?
- How often do you use it?
- Is it easy to use?
- Have there been any problems with it?
- Has the MND nurse contacted you about your answers?
- Have you talked about your answers during your clinic visits?
- How has it changed your MND care?
- Have you used the education section or the problem list?
- Would you like to use it as part of your routine care?
- How would you improve it?

For carers

- How have you found answering the questions?
- Has the MND nurse contacted you about your own well being?
- Would you like to use it as part of your routine care?

Hobson E, *et al. BMJ Open* 2019; 9:e028526. doi: 10.1136/bmjopen-2018-028526

Telehealth in Motor Neurone Disease

Telehealth in Motor Neurone Disease (TiM): A mixed methods, randomised controlled, pilot study of the use of the TiM telehealth system to deliver highly specialised care in Motor Neurone Disease, at a distance

Statistical Analysis Plan Version 1.3 17/8/16

Authored by

_________________________/__/____

Dr Esther Hobson *Date*

TiM trial manager & co-investigator

SITraN, University of Sheffield

Approved by

_________________________/__/____

Dr Christopher McDermott *Date*

TiM Chief Investigator

SITraN, University of Sheffield

_________________________/__/____

Mr Michael Bradburn *Date*

TiM Trial Statistician

CTRU, University of Sheffield

Contents

1 Introduction, Study Design & Objectives 5

1.1 Study Background 5
1.2 Primary Objectives 5
1.2.1 Feasibility Outcomes 5
1.2.2 Clinical Outcomes 6
2 Sample Size Estimation 7
3 Randomisation & Blinding 8
4 Interim Analysis & Study Monitoring. 8
5 Data Sources, Evaluability & Study Populations 8
5.1 Data Sources 8
5.2 Data Collection 9
5.3 Protocol non compliances 9
5.4 Study Population 9
5.5 Analysis Populations 9
6 Statistical Analysis 10
6.1 General considerations 10
6.2 Recruitment and attrition rates 10
6.2.1 Eligibility 10
6.2.2 Participant Attrition 11
6.3 Status of participants and completion of outcome measures 11
6.4 Baseline Characteristics 11
6.5 TiM Treatment adherence 12
6.6 Clinical outcomes 13
6.6.1 Patient outcomes 13
6.6.2 Carer clinical outcomes 16
6.6.3 Health economic outcomes 16
6.6.4 Patient experiences 16
6.6.5 Safety 17
6.7 Estimation of primary outcome and sample size for a main trial 17
6.8 Economic Evaluation Analysis 18
6.9 TiM process evaluation 18
7 Detailed Statistical Methods & Calculations 19
7.1 Missing Spurious & Unused Data 19
8 Implementation of the Analysis Plan 19
9 Modifications to the Original Protocol Analysis Statement 19
10 Appendix 20
11 20
10.2 Example Tables and Figures 22
12 References 26

List of Abbreviations

AE	Adverse event
ALS	Amyotrophic lateral sclerosis
ALSAQ-40	Amyotrophic Lateral Sclerosis Assessment Questionnaire – long form
ALS-FRS-R	Amyotrophic lateral sclerosis rating scale-revised
CI	Confidence Interval
CONSORT	Consolidated standards of reporting trials
CRF	Case Report Form
CSS-MND	Clinical Saliva Scale for Motor Neurone disease
CTRU	Clinical trials research unit, University of Sheffield
EQ-5D-3L	EuroQol Group Health Questionnaire
EQ-5D+D	EQ-5D questionnaire with dignity bolt-on
HADS	Hospital Anxiety and Depression Scale
ICH	International Conference on Harmonisation of Technical Requirements for Registration of Pharmaceuticals for human use
ITT	Intention To Treat
QoL	Quality of life
MND	Motor neurone disease
NIV	Non-invasive ventilation
SAE	Serious adverse event
SAP	Statistical analysis plan
SD	Standard deviation
SF-36 RAND	36-Item Short Form Survey from the RAND Medical Outcomes Study
SITraN	Sheffield Institute of Translational Neuroscience
SOP	Standard operating procedure
Telehealth	Remote monitoring of patients physiology or patient reported measures, forwarded to a central service with the aim to diagnoses or monitor a medical condition
TMG	Trial management Group
TSC	Trial Steering Committee
TiM	Telehealth in Motor neurone disease
TM	Trial manager (EH)
ZBI	Zarit Burden Index

1. Introduction, Study Design & Objectives

This Statistical Analysis Plan (SAP) is written in conjunction with the ICH E9 (International Conference on Harmonisation of Technical Requirements for Registration of Pharmaceuticals for human use; ICH Harmonised Tripartite Guideline: Statistical Principles for Clinical Trials E9), applicable standard operating procedures (SOPs) from the Sheffield Clinical Trials Research Unit (CTRU) and trial documents (Protocol, case report form (CRF) and Data Validation Specifications). This SAP will guide the trial manager (TM) and Trial Statistician during the statistical analysis of all quantitative outcomes in order to answer the objectives of the study.

1.1 Study Background

This is a single-centre, pilot, mixed methods, randomised controlled trial to explore the feasibility and acceptability of using the TiM (Telehealth in Motor Neurone Disease (MND)) system in clinical practice and explore the feasibility of a larger, multicentre trial. This plans refers to the TiM trial protocol V1.5 April 2015.

All analyses will be performed in a validated statistical software package such as GraphPad prism.

1.2 Primary Objectives

As this is a pilot study, no formal primary clinical outcome will be defined. Instead, the trial will assess the feasibility and requirements of a full-scale study of the TiM as defined by the as successful recruitment of 40 eligible patients and their primary carer; and the feasibility, acceptability, safety and use of the TiM system within a health service. The specific objectives and outcomes of this study are separated into two groups: feasibility and clinical outcomes.

1.2.1 Feasibility Outcomes

Feasibility of a full-scale study

- To make a decision on the primary outcome for the main trial. The mechanism for choosing this outcome will be informed by statistical considerations which are detailed in section 6.7.
- Number of potentially eligible patients among the pool of patients under the care of the Sheffield MND care centre
- Number/characteristics of eligible patients approached for the study:
- List of reasons for declining/refused consent;
- Participant attrition rate
- List of reasons for attrition
- Number of missing values/incomplete cases (see **Error! Reference source not found.**)

- Treatment receipt/adherence;
- Patient, carer and clinician views on intervention/research protocol (using qualitative methods).

Feasibility/safety of TiM system:

- Treatment receipt/participant and staff adherence
- Participant and clinician acceptability of the intervention (using qualitative methods and the TiM system experience questionnaire and Shadow monitoring protocol)
- Patient, carer and clinician views on intervention (using qualitative methods);
- Incidence of TiM system technical problems;
- Incidence of adverse events related to intervention.

Participant and clinician views will be investigated using qualitative interviews (described in the protocol). Participant and clinician acceptability will be reported based on TiM system experience questionnaire and Shadow monitoring questionnaire.

1.2.2 Clinical Outcomes

The following clinical outcomes will be reported using self-completed questionnaires baseline, 3, 6, 12 and 18 months.

Patient outcomes

- Quality of life (QoL) measures
  - ALSAQ-40 (an MND disease specific quality of life score)
  - SF-36-RAND
  - EQ-5D+D (EQ-5Q-3L with a dignity bolt-on)
- Clinical outcomes
  - ALS-FRS-R (an MND disease specific functional rating score
  - Pain score (modified Likert scale)
  - CSS-MND Saliva Severity Scale (designed for use with MND patients) plus global change scale
  - Hospital Anxiety and Depression Scale (HADS)
- Health resource usage questionnaire
- Patient experience questionnaire
- TiM experience questionnaire

Carer outcomes

- SF-36 RAND
- 12 item Zarit Burden Inventory (ZBI) (53)
- Hospital Anxiety and Depression Scale (54)
- Carer satisfaction questionnaire

Hobson E, *et al. BMJ Open* 2019; 9:e028526. doi: 10.1136/bmjopen-2018-028526

The following safety outcomes will be assessed at every clinical visit:

- Incidence of adverse events (AEs)
- Clinician satisfaction

2 Sample Size Estimation

The study aims to recruit a total of 40 patients and their carers. 20 patients and their primary carer will be randomised to the intervention arm (a minimum of 6 months use of the TiM telehealth plus usual care) and 20 patents and their carer in the control arm (usual care).

Since the proposed trial is primarily an assessment of the acceptability of the intervention and the feasibility of a full trial, the proposed sample size is not based on standard statistical parameters such as a clinically relevant difference between groups. Instead, the sample size is justified on the grounds of quantifying patient variance (i.e. the standard deviation) in the proposed outcome measures (in particular quality of life measures) and on feasibility of the full trial, as follows:

- A sample size of 40 patients allows a standard deviation to be estimated to within a precision of ±20% of its true underlying value with 90% confidence. This estimate will be synthesised by combining baselines measurements of quality of life measurement standard deviations with those observed in other published studies and on-going trials within SITraN, to provide a robust estimate for use in the sample size calculation for the full trial.

- Given the rarity of MND, any definitive study will be infeasible if the required sample size is substantial. Assuming the upper limit for feasible UK study is around 200-300 patients in total, it follows that the full study would need powering to detect a standardised effect size of at least 0.4 SDs. This pilot trial will provide a preliminary assessment of whether the intervention might feasibly achieve this, and inform the choice of outcome measures for the proposed full study.

This sample size is also in keeping with the proposal of 12 evaluable patients per arm in a pilot study (after withdrawal or drop-out) (1).

Hobson E, *et al*. *BMJ Open* 2019; 9:e028526. doi: 10.1136/bmjopen-2018-028526

3 Randomisation & Blinding

Randomisation is conducted according to the protocol.

The patient, clinicians, TM and trial team are not blinded to the outcomes. Data entry for follow-up clinical outcomes was performed by an independent research nurse, not involved in the study. Blinding of this nurse was impractical given additional measures were collected for those in the intervention group. The TM will undertake the analysis under the supervision of the independent trial statistician. Blinding during analysis was impractical given the small number of participants who had with unique and characteristics which are likely to be identifiable to the TM. This will be reported as a limitation

4 Interim Analysis & Study Monitoring.

This is a pilot study with no planned interim analysis or early stopping. Two committees have been set up to govern the conduct of the study:

- Trial Steering Committee (TSC)
- Trial Management Group (TMG)

Decisions to stop the trial early on grounds of safety will be made by the Trial Steering Committee or funding body. There will not be a Data Monitoring and Ethics Committee for this study as it is considered low risk. No interim analysis is planned.

The TM will receive notifications of all carers whose Hospital Anxiety and Depression subscores exceed 11. These events will be recorded as AEs, reported to the TMG and TSG during the study and reported in the analysis.

5 Data Sources, Evaluability & Study Populations

5.1 Data Sources

Data used in this study will come from data entered onto CRFs and questionnaires and from data entered directly on the CTRU database (PROSPECT). The data will be stored on the database with the exception of the randomisation list which is held on www.sealedenvelope.com and allocation verified by the data management team. Electronic data will be extracted from the system during the trial for the purpose of checking (validating) and trial progress reports. Access to PROSPECT is controlled by usernames and encrypted passwords, and a privilege management feature will be used to ensure that users have access to only the minimum amount of data required to complete their tasks. This will be used to restrict access to personal identifiable data.

Hobson E, *et al. BMJ Open* 2019; 9:e028526. doi: 10.1136/bmjopen-2018-028526

5.2 Data Collection

Data will be collected from the participants and their carers at:

- Consent and Screening, eligibility and baseline
- Month 3, 6, 12 and 18
- Each clinic visit (Shadow monitoring protocol)
- End of study (participant status alive/dead and date of death).

Due to the pilot nature of the study there are no predefined protocol non compliances other than misrandomisation or randomisation in error. Intervention adherence will be assess as an outcome (see section 6.5).

5.3 Protocol non compliances

Due to the pilot nature of the study there are no predefined protocol non compliances other than misrandomisation or randomisation in error. Intervention adherence will be assess as an outcome (see section 6.5).

5.4 Study Population

Described in the protocol.

5.5 Analysis Populations

The intention to treat population (ITT) includes all patients for whom consent is obtained and who are randomised to treatment. This is the primary analysis set and endpoints will be summarised for the intention to treat population unless stated otherwise.

Hobson E, *et al. BMJ Open* 2019; 9:e028526. doi: 10.1136/bmjopen-2018-028526

6 Statistical Analysis

6.1 General considerations

As the trial is a pilot parallel group randomised controlled trial, data will be reported and presented according to the proposed modifications for reporting pilot trials as well as the Consolidated standards of reporting trials (CONSORT) statement (2,3). The analysis will be performed on an ITT basis. The final analysis will be performed after data lock by the TM under the supervision of the study statistician who will also be responsible for quality checking the results.

Each planned follow-up timepoint will use a time window to ensure that responses have been collected within a reasonable time frame. The time windows allow a slippage of four weeks at 3 months and six weeks thereafter, as outlined below:

3 months: within 61-91 days following randomisation

6 months: within 140-224 days following randomisation

12 months: within 323-407days following randomisation

18 months: within 506-590 days following randomisation

6.2 Recruitment and attrition rates

Relevant summaries related to recruitment, consent and patient throughput will be reported and presented in a CONSORT flow diagram (see appendix, Figure 1).

The following will be reported:
The number of (potential) participants;

- Potentially eligible as identified by the study team at participating centres,
- Approached for the study,
- Not randomised (with reasons),
- Randomised,
  - allocated to treatment
  - allocated to control
- Withdrawn and lost to follow up (with reasons),
- Discontinuing TiM intervention,
  - reasons for discontinuation
- Included and excluded from analysis,
  - Reasons for exclusion.

6.2.1 Eligibility

Described in the study protocol

Hobson E, *et al. BMJ Open* 2019; 9:e028526. doi: 10.1136/bmjopen-2018-028526

6.2.2 Participant Attrition

The rate of attrition will be reported (defined as the proportion of the consented and randomised participants who withdrew or were lost to follow up). The reasons for attrition, where provided, will be reported as number and percentage in each category.

6.3 Status of participants and completion of outcome measures

We will report the status of patients and carers at each time point.

At each time-point we will report the number of patients and carers:

- Returning the postal questionnaire booklet
- Completing each questionnaire

We will report these by treatment group and overall.

For the patient and carer questionnaires the response rate at each time point (measured as the total number of questionnaires completed as a fraction of total number of patients alive) will be reported. An example table is given in section 0 (Table 1).

6.4 Baseline Characteristics

The baseline demographics and clinical characteristics of the participants will be reported. For the continuous variables, (e.g. age) either mean and standard deviation will be presented or median and inter quartile range (IQR) depending on the distribution of the data. The number of observations used in each calculation will be presented alongside the summaries. For the categorical variables, the number and percentage of participants in each of the categories and the total number of observations will be presented.

All baseline summaries will be presented and reported for each treatment group and in total. An example of the table of baseline summaries is given in section 0 (

Table 2). No statistical significance testing will be done to test baseline imbalances between the intervention arms but any noteworthy differences will be descriptively reported.

Hobson E, et al. BMJ Open 2019; 9:e028526. doi: 10.1136/bmjopen-2018-028526

The following summaries will be presented:

Demographics	Age, gender, technology use
MND Characteristics	Age of onset, disease duration, classification of MND (e.g. ALS, PMA, PLS), clinical stage of MND, use of non-invasive ventilation (NIV)/gastrostomy, riluzole use
Carer demographics	Age, gender, relationship to patient, technology use
Patient reported outcomes	ALS-FRS-R (including upper limb function), ALSAQ40, RAND36 and subscores, CSS-MND, HADS, pain score, EQ-5D+D, patient experience, health resource use (number and type of clinical encounters and hospital admissions in last 3 months, carer requirements)
Carer reported outcomes	RAND36, ZBI, HADS, carer experience

6.5 TiM Treatment adherence

Intervention adherence will be reported as the number of TiM sessions attended within between recruitment and the end of March 2016 and the mean and SD of percent adherence. We will also report adherence at 1, 3, 6, 9, 12, 15 and 18 months.

We will also report

- **The number and percentage of participants that completed 50% and 75% of expected sessions.**
- **A description of the adherence of each patient and carer using the TiM over the course of the trial.**

Any reasons for poor adherence will be reported where available although it was not possible to identify reasons for all missed sessions.

Cumulative session attendance will be displayed for each participant using a spaghetti plot to illustrate intervention adherence.

The number and percentage of participants that withdrew from the TiM intervention will be reported, alongside listings of:

Hobson E, *et al. BMJ Open* 2019; 9:e028526. doi: 10.1136/bmjopen-2018-028526

- **Reasons for withdrawing from intervention, where provided**
- **Number of TiM sessions (and %) before withdrawing from intervention**

6.6 Clinical outcomes

Descriptive statistics will be presented for the clinical outcomes; significance testing will not be undertaken. Continuous outcome measures will be presented as mean differences between groups and their associated 95% confidence intervals (CI). For categorical outcomes, the number and percentages falling into different categories and potential differences between groups in terms of the percentages in each category will be presented, together with their confidence intervals. Clinical outcomes will be presented for the ITT set with available 6 month and 12 month outcome data.

6.6.1 Patient outcomes

The following outcomes measured at 3, 6, 12, 18 months will be presented by group and overall.

The following patient –reported quality of life outcomes will be reported.

ALSAQ-40	Individual scores of five sub-scales and a summary aggregate score:<br>▪ physical mobility<br>▪ activities of daily living and independence<br>▪ eating and drinking<br>▪ communication<br>▪ emotional reactions
RAND-36	A summary of the eight sub-scales and two aggregated scales:<br>▪ Physical Functioning<br>▪ Role Limitations due to Physical Problems<br>▪ General Health Perceptions<br>▪ Vitality<br>▪ Social Functioning<br>▪ Role Limitations due to Emotional Problems<br>▪ General Mental Health<br>▪ Health Transition<br>▪ Aggregate physical health<br>▪ Aggregate mental health
EQ-5D+D	Health utility (as derived from the five questions)<br>Thermometer health scale<br>Health utility plus dignity (as derived five questions plus

	dignity bolt-on)

In each case the within-group results will be summarised as mean (SD), and the difference between the two as the mean difference together with its CI. Forest plots of confidence intervals of different widths (e.g. 95%, 90%, 80%) with respect to the treatment difference in the overall ALSAQ40 score and RAND36 (mental and physical domain) will be used to illustrate the strength of preliminary evidence (see Figure 2) (Lee, 2014).

In each case, the summaries will be presented by treatment group and time point (see Table 3 and

ll be presented for the clinical outcomes; significance testing will not be undertaken.
significance testing will not be undertaken. Continuous outcome measures will be
ignificance testing will not be undertaken. Continuous outcome measures will be
presented as mean differences between groups and their associated 95% confidence
erences between groups and their associated 95% confidence intervals (CI). For
5% confidence intervals (CI). For categorical outcomes, the number and percentages
% confidence intervals (CI). For categorical outcomes, the number and percentages
ntages falling into different categories and potential differences between groups in
potential differences between groups in terms of the percentages in each category will
potential differences between groups in terms of the percentages in each category will
ome data.
ll be presented by group and overall.
tcomes will be reported.
ary aggregate score:
obility
g
-36
le 3 and
e number of patients alive and the number of patients completing the outcome
score mean and SD
ure will be scored as described below and compared, where possible and relevant to

ompared, where possible and relevant to population values.
be scored as described in Ware et al (4). In the case of partially completed
9D4-BDB0-
C034</uuid><priority>0</priority><publications></publications></citation>(4). In
publications></citation>(4). In the case of partially completed questionnaires, scores
questionnaires, scores will be calculated for domains in which at least 50% of the
ons have been answered. Taking physical functioning as example, if at least five of the
ns have been answered. Taking physical functioning as example, if at least five of the
lity (Q1-10), activities of daily living/independence (Q11-20), eating and drinking,
ity (Q1-10), activities of daily living/independence (Q11-20), eating and drinking, (Q21-
(Q21-23) communication (Q24-30), emotional functioning (Q31-40). Each question is
unctioning (Q31-40). Each question is scored 0 (never) to 4 (always/cannot do at all).
nctioning (Q31-40). Each question is scored 0 (never) to 4 (always/cannot do at all).

5 +Q6 +Q7 +Q8 + Q9 + Q10)/40) x 100.
l).
urology, Neurosurgery & Psychiatry</title><type>-100</type><subtype>-
0</subtype><uuid>0303282F-D994-4EC9-B986-
bundle><authors><author><firstName>C</firstName><lastName>Jenkinson</lastNa
or><firstName>C</firstName><lastName>Jenkinson</lastName></author><author><f
stName>Jenkinson</lastName></author><author><firstName>J</firstName><middle
tName>Jenkinson</lastName></author><author><firstName>J</firstName><middleN

The following other clinical outcomes will be presented:

Hospital anxiety and depression score	The Anxiety and Depression subscores
Pain (Likert scale)	Current and average weekly score
ALS-FRS-R	Total score
CSS MND	Total score<br>% patients reporting a clinically significant improvement or worsening (according to Global change CSS-MND self-reported statement)
Clinical encounters	The number of clinical encounters in the 6 months following randomisation, by type and location and reason
Hospital admissions	The number and percentage of patients admitted to hospital, and the number of hospitalisations, by type, location and reason

The HADS anxiety and depression inventories will be scored using the approach of Zigmond and Snaith (7). Each domain will be calculated as the sum of seven questions, each of which is scored 0-3, giving a total score which ranges from 0 and 21. In the case of partially completed questionnaires, the domain will be scored and upweighted provided at least four of the seven questions have been answered.

Self-completed revised ALS functional rating scale (ALS-FRS-R) consists of 12 questions scoring 0-4 (8). Sub-domains include upper limb, lower limb, bulbar and respiratory.

Pain score: the current level of pain (0-10 likely scale) and the average current weekly level of pain (0-10) will be represented as a mean and SD.

Modified CSS MND saliva score is awaiting validation. The total score is the total of all answers scoring 0-3 for each question A to J (9). The percentage of patients reporting a change on the saliva clinical change assessment will be reported.

6.6.2 Carer clinical outcomes

The following carer-reported QoL outcomes will be presented is the same manner as described for the patients.

- RAND 36
- HADS

Hobson E, *et al. BMJ Open* 2019; 9:e028526. doi: 10.1136/bmjopen-2018-028526

- Zarit Burden Index

The each of the 12 items in the shortened Zarit burden inventory is scored 0 (Never) to 4 (nearly always) (10). A total score between 0 and 48 will be reported.

6.6.3 Health economic outcomes

A complete health economic analysis is beyond the scope of this plan. However, descriptions of the following clinical outcomes will be reported, by group and overall:

Clinical encounters	The number of clinical encounters recorded at each encounter following randomisation, by type and location.
Hospital admissions	The number and percentage of patients admitted to hospital recorded at each encounter. the number of hospitalisations
Informal care requirements	The number of hours of informal care recorded by patients.
Formal care requirements	The number of hours of formal care recorded by patients.

6.6.4 Patient experiences

The following will be reported by group and overall.

Patient care experience	Percentage of patients agreeing and disagreeing with each satisfaction statement
Carer care experience	Percentage of carer agreeing and disagreeing with each satisfaction statement
Patient TiM experience (intervention only)	Percentage of patients agreeing and disagreeing with each satisfaction statement
Carer TiM experience (intervention only)	Percentage of carer agreeing and disagreeing with each satisfaction statement

All free text responses will be reported.

6.6.5 Safety

Adverse events are recorded at every clinic appointment and patients will report health resource use and hospital admissions. Reported admissions will be followed up by the

TM and records as serious adverse events. HADS carer scores will be calculated and reported to the TM for action on an ongoing basis during the trial if either the depression or the anxiety subscore exceeds 11. These will be reported to the TSC during the trial and reported in the analysis and recorded as adverse events.

Advents Events (AEs) will be reported as number and percentage of patients overall and by treatment group but no formal statistical analysis is planned. The following summaries will be presented;

AEs	The number and percentage* of patients reporting an AE and the number of AEs in total
AEs by category	The number and percentage* of patients reporting an AE and the number of AEs for each pre-defined category (pain, acute infection, fractures)
Serious AEs (SAEs)	The number and percentage* of patients reporting an SAE and the number of SAEs in total
Treatment-related AEs	The number and percentage* of patients reporting a treatment related AE and the number of treatment related AEs
All AEs	A listing of all AEs including<br>- Description / Site / Signs and Symptoms<br>- Severity<br>- Relationship<br>- Action taken<br>- Outcome<br>- Seriousness

*defined as a percentage of all patients randomised.

6.7 Estimation of primary outcome and sample size for a main trial

The variability in clinical outcomes will be reported as standard deviation by treatment group and overall alongside their upper 80% confidence limits to get a robust estimate of SD (as recommended by Kieser, 2007), and observed treatment difference.

Descriptive assessment will be used to inform sample size calculations for the definitive study. These assessments will be calculated for candidate measures for the full trial (RAND-36 and ALSAQ40), and will be based on:

- Observed treatment difference at 6 and 12 months
- Standard Deviation;
- Correlation between baseline and 6 month measurements;
- The extent of missing data in each outcome;
- Participant feedback on the most appropriate assessment (analysed qualitatively).

Hobson E, *et al. BMJ Open* 2019; 9:e028526. doi: 10.1136/bmjopen-2018-028526

The standard deviation used in the sample size calculation will be derived from the residual variance of the regression model for which the outcome is the 6-month response and the covariates are treatment group and baseline.
A table of sample size estimates for a definitive study stratified by outcome measure and power (80%, 90%) will be provided. E.g. Table 5

6.8 Economic Evaluation Analysis

No economic analysis will be conducted but patient health resource use will be reported.

6.9 TiM process evaluation

The following will be reported:

Patient and carer feasibility:

- The time taken to complete each TiM session by patient and carer (mean, range). TiM session time is automatically recorded by the application but total time between starting and completing and session is recorded. This includes any time delay because the patient pauses using the session and recommences it later e.g. the next day. Outliers will be identified and excluded with definition of outlier reported (e.g. > 600% of the average time);
- Adherence to weekly TiM sessions (see 6.5);

Clinical feasibility:

- Number, range and % of patient and carer sessions that trigger an overall red, amber and green flag;
- Number, range and % of patient and carer sub-sections that trigger an overall red, amber and green flag;
- Time taken for nurse to use the telehealth system per week, collected by nurse diary (mean, range, SD and time per patient enrolled in the system);
- Number of notes entered per patient.
- Shadow monitoring protocol (intervention)
    - Number of pre-clinic shadow monitoring forms completed
    - Number of clinic shadow monitoring forms completed
    - Clinician satisfaction: % agree/disagree with each statement
    - Free text comments will be reported.

7 Detailed Statistical Methods & Calculations

7.1 Missing Spurious & Unused Data

The extent of missing data will be reported. No sensitivity analyses involving imputation for missing data will be performed. Any spurious data will be queried and checked for consistency with data management before data lock.

Patient and carer questionnaires will be scored only if all relevant items that make up a domain are completed with the exception of RAND 36, HADS and ALSAQ40.

8 Implementation of the Analysis Plan

This SAP will be used as a work description for the statistician involved in the trial. All analyses will be performed by the TM (under the supervision of Trial Statistician MB).

Initially, blinded data will be delivered to the TM and MB by the data manager to define analysis sets and test statistical programs. Any queries will be communicated to the study and data manager prior to database lock. The database will be locked after agreement between the statistician, data manager and study manager. No changes will be made once the data has been locked. Database freeze and lock will be conducted in accordance with SOP DM012.

9 Modifications to the Original Protocol Analysis Statement

None

Hobson E, *et al. BMJ Open* 2019; 9:e028526. doi: 10.1136/bmjopen-2018-028526

10 Appendix

Figure 1: CONSORT flow diagram

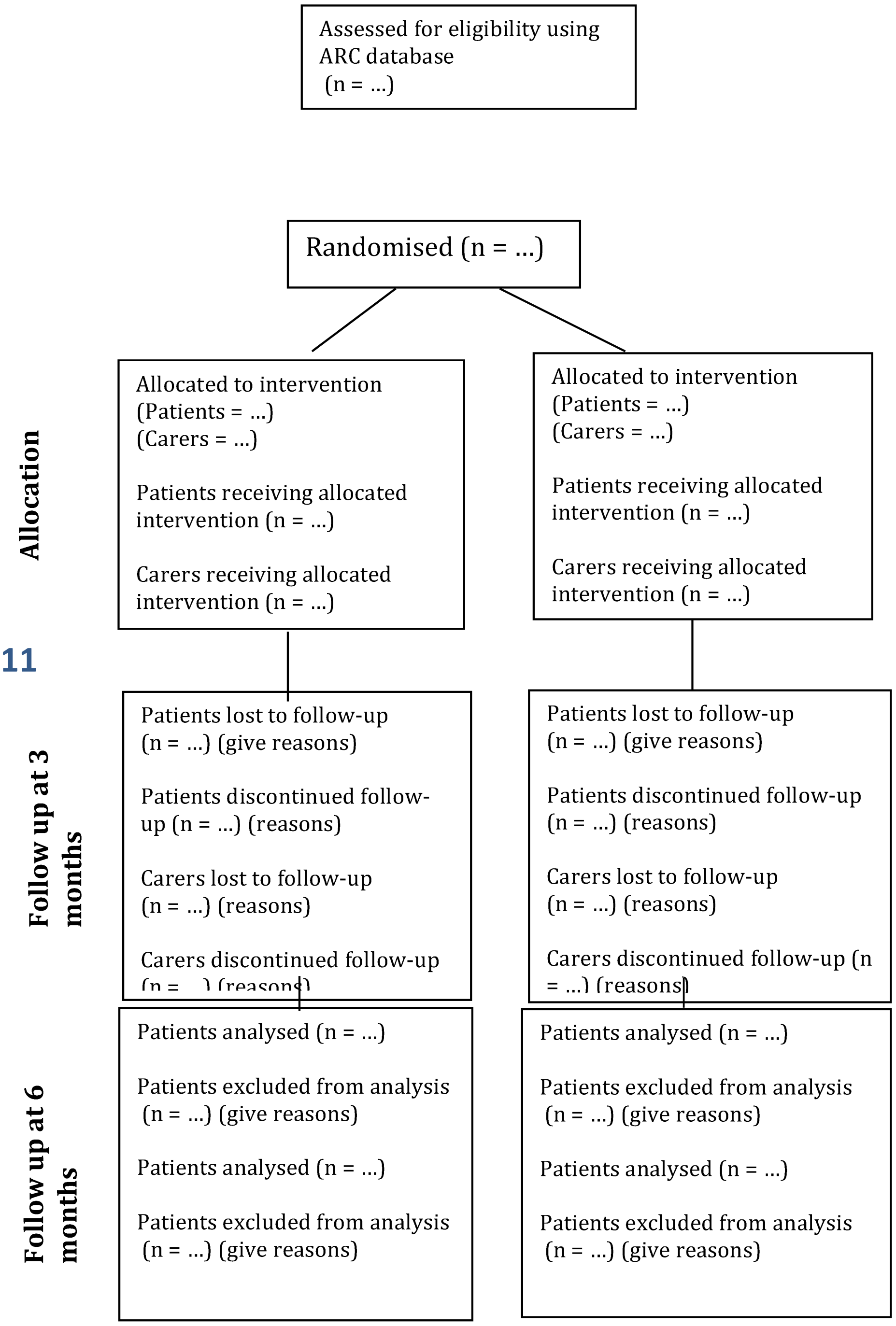

11

Hobson E, *et al. BMJ Open* 2019; 9:e028526. doi: 10.1136/bmjopen-2018-028526

Figure 2: Mean difference in ALSAQ40 with confidence intervals

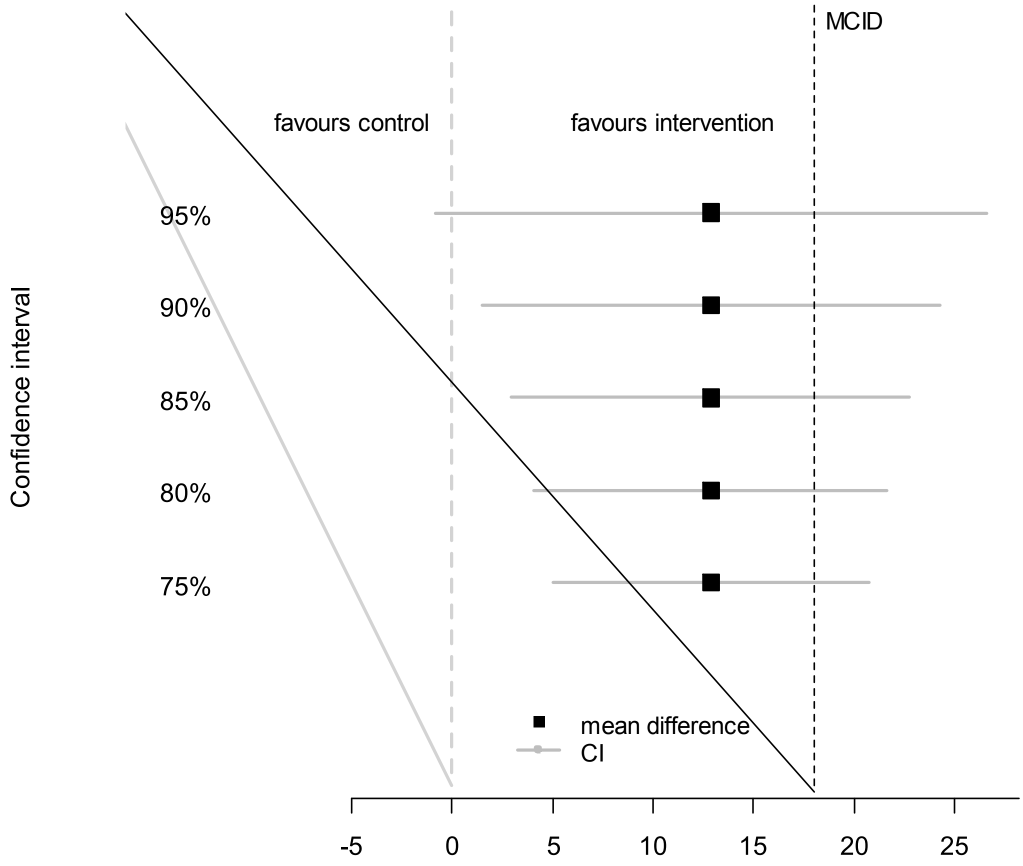

10.2 Example Tables and Figures

Note: The following tables are examples and do not include all outcome measures that will be included in the analysis.

Table 1: Participant status

	3 months	6 months	12 months	18 months
**Telehealth**				
Completed	N=	N=		
Not completed				
Died				
Withdrew from study				
Completed questionnaire but not within time window				
Incomplete				
**Control**				
Completed	N=	N=		
Not completed				
Died				
Withdrew from study				
Completed questionnaire but not within time window				
Incomplete				

*Completed includes questionnaires that were sufficiently complete to be used in the statistical analysis. Uncompleted refers to questionnaire booklets that were not returned. Incomplete refers to questionnaire booklets that were returned but insufficiently complete to be used in statistical analysis.

Table 2: Participant baseline characteristics by treatment group

Characteristic	Scoring	Control (n=xx)	Intervention (n=xx)	All (n=xx)
Age (years)	Mean(SD, range)	x (xx)	x (xx)	x (xx)
	N	n	n	n
Gender	Male	n (%)	n (%)	n (%)
	Female	n (%)	n (%)	n (%)
	N	N	n	n
ALS-FRS-R	Mean(SD, range)	x (xx)	x (xx)	x (xx)
	N	n	n	n
King's clinical stage	Stage 1	x (xx)	x (xx)	x (xx)
	Stage 2…etc.			
	N	n	n	n

This will be extended to include the other baseline variables measured.

Table 3: Display of outcome data by time, illustrated for pain

	Baseline	3 months			
Outcome		Mean (SD)	Change from baseline	*…repeat for other timepoints*	
Current pain: Mean (SD)	Mean (SD)	N= Mean (SD)	Mean (CI)		
Control	Mean (SD)	N= Mean (SD)	Mean (CI)		
Average pain: Mean (SD)	Mean (SD)	N= Mean (SD)	Mean (CI)		
Control	Mean (SD)	N= Mean (SD)	Mean (CI)		

Hobson E, *et al. BMJ Open* 2019; 9:e028526. doi: 10.1136/bmjopen-2018-028526

Table 4: Clinical outcomes at six months: control vs intervention

	Change from baseline						
	Intervention			Control			
Outcome	n	Mean	SD	n	Mean	SD	Mean difference (95% CI)
ALSAQ40	x	xx	xx	x	xx	xx	xx (xx to xx)
RAND 36 (agg. physical)	x	xx	xx	x	xx	xx	xx (xx to xx)
RAND 36 (agg. mental)	x	xx	xx	x	xx	xx	xx (xx to xx)
HADS anxiety	x	xx	xx	x	xx	xx	xx (xx to xx)
HADS depression	x	xx	xx	x	xx	xx	xx (xx to xx)
Pain	x	xx	xx	x	xx	xx	xx (xx to xx)
…							

Hobson E, *et al*. *BMJ Open* 2019; 9:e028526. doi: 10.1136/bmjopen-2018-028526

Table 5: Sample size considerations for candidate primary outcome measures

Outcome	Effect size MCID	Observed*	Standard deviation		Power (%)	Number /group	Number /group + attrition
ALSAQ-40 total	xx	Xx	Observed	xx	80	NN	NN
					90	NN	NN
			Upper 80%CI	xx	80	NN	NN
					90	NN	NN
RAND-36							
Agg physical	5	Xx	Observed	xx	80	NN	NN
					90	NN	NN
			Upper 80%CI	xx	80	NN	NN
					90	NN	NN
Agg mental	5	xx	…				
…repeat for other candidate measures							

**nb Observed effect size is for reference and is not used in sample size calculation*

TiM Telehealth in Motor Neurone Disease

Shadow monitoring form

[Intervention]

T ☐☐☐

Appointment summary

Date of clinic ☐☐ ☐☐ ☐☐☐☐
d d m m y y y y

Neurologist completing form ☐ CMD ☐ PJS ☐ Other → ☐ *specify*

Mode of contact ☐ Face-to-face ☐ Telephone

Type of appointment ☐ Routine ☐ Unscheduled

Related to telehealth ☐ Yes ☐ No

Details ☐

Next scheduled visit ☐☐ weeks

Adverse events

Any adverse events (including hospital visits) since last visit? ☐ Yes ☐ No

*Please complete **Adverse event** or **Hospital attendance** form and contact CI immediately*

Any adverse drug reactions since last visit? ☐ Yes ☐ No

*Please complete **Adverse event** form and contact CI immediately*

TiM

	Completely disagree	Disagree	Neutral	Agree	Completely agree
The TiM data gave an accurate picture of the patient's current condition	☐	☐	☐	☐	☐
The TiM data could enable me to make appropriate decisions about the patient without seeing them in clinic	☐	☐	☐	☐	☐
The TiM data gave useful information about the carer's current condition	☐	☐	☐	☐	☐
The TiM was a positive influence on the consultation	☐	☐	☐	☐	☐
Prior to the patient attending you said you would arrange the patient to attend clinic in ______ weeks. Do you think this was an appropriate decision?	☐	☐	☐	☐	☐

<date> v1 draft <Page number>
