# Supplementary material for: Process evaluation and exploration of telehealth in motor neuron disease in a UK specialist centre
Source: BMJ Open. 2019 Oct 22;9(10):e028526. doi: 10.1136/bmjopen-2018-028526 (PMC6830641; doi:10.1136/bmjopen-2018-028526)
Supplement: Supplementary data [file bmjopen-2018-028526supp002.pdf]

Telehealth in motor neurone disease to increase access to specialist multidisciplinary care: a process evaluation.

Supplementary data file

Table of Contents

Appendix table 1 Participants' initial impressions of using the TiM app. 2
Appendix table 2 The characteristics of those lacking confidence in technology user and their approach to using technology. 3
Appendix table 3 Negative attitudes to technology expressed by participants....... 4
Appendix table 4 Facilitators for TiM use. 5
Appendix table 5 Participants attitudes towards the frequency of TiM sessions... 6
Appendix table 6 Reasons for frequent and infrequent adherence to TiM identified during the interviews. 7
Appendix table 7 Participants' expectations of the potential communication and monitoring benefits of the TiM service. 8
Appendix table 8 Participants' expectations of the TiM service. 9
Appendix table 9 The acceptability of the TiM questions. 10
Appendix table 10 The validity / accuracy of the TiM questions. 11
Appendix table 11 Participants understanding of the TiM service. 12
Appendix table 12 Nurses' experience of using the TiM clinical portal. All quotes are from the Telehealth Nurse. 13
Appendix table 13 The accuracy and sensitive of the TiM information. Quotes are from the Telehealth Nurse (N) unless otherwise stated. 14
Appendix table 14 Participants' experiences of how the TiM impacted on their care. 15
Appendix table 15 The Telehealth Nurse's attitudes towards the value of the TiM. 16
Appendix table 16 Participants experiences of clinician feedback. 17
Appendix table 17 The mismatch between the participants' and nurses' expectations of the TiM service. 18
Appendix table 18 The problems associated with excessive TiM alerts. All quotes are from the Telehealth Nurse unless indicated. 19
Appendix table 19 The problems associated with excessive TiM alerts. 20
Appendix table 20 The future of the TiM. 21
Appendix table 21 The Telehealth Nurse's attitudes towards how the TiM might be used in the future. 22
Appendix table 22 How the TiM might be used by other teams 23

Hobson E, *et al. BMJ Open* 2019; 9:e028526. doi: 10.1136/bmjopen-2018-028526

Appendix table 1 Participants' initial impressions of using the TiM app.

**Initial use of the app**	
Easy to use	*"It's so easy to do; it literally takes five minutes from home." P317 [daily technology user]*
Quick to use	*"It's not a problem is it? It takes minutes, it's not like you have to sit there for half a day and do it, it literally, it's, it's sort of minutes." P248 [daily technology user]*
TiM accessible and acceptable to the elderly	*"So this lady [P217], she's an elderly lady who you thought wouldn't have embraced anything like this. But she has done and she, her and her husband both send it back; she's very disabled but able to use her hands quite well…they're both in their eighties; and she's a very good replier, sends it in." Telehealth nurse*
TiM accessible to those with significant disabilities	*"[There are] some are patients that, I'm surprised they took it up. I'm surprised; actually that, that, I never thought that he would use that… and he sends his back very, very well." Telehealth nurse*
Confidence the information would be held securely	*"I'm assuming that your department is one that's reasonably secure and has got reasonable standards… I can only make my judgement on the people I meet who are involved in it." C166*

Appendix table 2 The characteristics of those lacking confidence in technology user and their approach to using technology.

**Characteristics of those with low technology confidence**	
Unsure if they would be able to use the TiM	*"…we're quite happy to deal with it, as long as it wasn't too techy" C062 [uses technology a few times a week]*<br>*"…as long as I can do it" P392 [uses technology daily]*
Lack of experience using technology	*"I don't use the internet for my job and it's all still paperwork orientated" C395 [uses technology daily]*
Difficulties with language	*"C: It's the system that's all. I don't catch on very well with it, that's ok. Like phones, I don't bother them really.*<br>*P: Couldn't read a text message.*<br>*C: couldn't do anything like that." C217 [never uses technology]*
Frightened they might make the TiM go wrong	*"Q: Have you used any of the other things that are on the tablet, so the education things or the website?*<br>*P: Oh I daren't touch all that stuff in case it goes wrong. (laughs)…I pressed that one day and I got a bit panicky (laughter) so I… left it…I just use it for the questionnaire now and that's it." P145 [uses technology daily]*
Frightened they might break it	*"…plus it doesn't belong to us and if we broke it we'd be devastated" C145 [uses technology daily]*
Frightened they will enter wrong information	*"My fingers are too heavy. … I'll end up phoning somebody." P378 [once a week technology user]*
Technology use is stressful	*"But I can… if I'm, if I'm taught …without aggravation." C380 [uses technology every few weeks]*
Problems using technology perceived as failure	*"I tried to order a book on it last week and I can't do it (laughs) it kept, I've lost passwords and all sorts…" C392 [uses technology daily]*
**Low user's approach to technology**	
Use technology for only a limited number of familiar purposes	*"I have used the computer for various things, and if I'm using it for something and I know what I'm doing, that's fine; I can get on for certain things now…" C380 [uses technology every few weeks]*
Avoids using unfamiliar technology	*"Q: And, and you say you were a little bit worried about the technical side?*<br>*C: Well I am, I was… I can manage things… I use Kindle and a laptop. Computers are a bit more of a thinker… (laughs) and…I don't do them all the time so I'm all right with basic stuff." C062 [uses technology a few times a week]*<br>*"I know basically how to navigate [his own device], whereas [the TiM] I don't." P145 [uses technology daily]*
Unable to problem solve: relies on others	*"Q: And what do you do if you get, get stuck on a problem?*<br>*C: Have to wait till me daughter-in-law comes up and get her to sort it out for me." C392 [uses technology daily]*

3

Appendix table 3 Negative attitudes to technology expressed by participants.

**Negative attitudes towards technology**	
Lacking an interest in technology	*"It doesn't really interest me, if I'm being honest." C392 [uses technology daily]*
Not seeing a need for technology	*"I'm not against it but I don't want it and I can do without it and I don't need it." C166 [never uses technology]*
Feeling pressured to use technology	*"All the time we're being pressured [by people] that take all this marvellous technology up; and every day it is getting worse and worse, because it's getting beyond the control of the average person how to manage it properly." C166 [never uses technology]*
Feeling excluded/missing out if not using technology	*"I do feel…a slight social outcast, but I seem to be very much in the minority." C166 [never uses technology]*
Worried about the consequences of technology misuse	*"… certain things are very useful [but] once it gets to the mass market…it of gets exploded and misused. … You've got to the point of small businesses being blackmailed by hackers." C166 [never uses technology]*
Worried about online security	*"And as for banking online, God, it's a laugh, I would not touch it…if something goes wrong you have the devil's own job to try and put it right because there's no proof." C166 [never uses technology]*
Worried about intrusion into their privacy	*"I wouldn't have a Smartphone for a start, you know cos Big Brother's up there already." C166 [never uses technology]*
Previous negative experience using technology that was unreliable	*"…eventually these programs got more and more complicated and the time it was taking for sending information down and getting back was absolutely ludicrous; the thing would crash say at eleven o'clock on a Monday morning and you'd have to start all over again." C166 [never uses technology]*
Technology is an unwanted replacement for human contact	*"They can't pick up a phone normally anymore because they're so busy faffing about with their smart phones." C166 [never uses technology]*

Hobson E, *et al. BMJ Open* 2019; 9:e028526. doi: 10.1136/bmjopen-2018-028526

Appendix table 4 Facilitators for TiM use.

**Facilitators for TiM use**	
**Improving confidence using the TiM**	
Face to face training	*"I would take it up if I was shown that the program was spot on" C166*
Clear instructions	*"…That the instructions were aimed at an idiot like me… and not on the assumption that I would know what to do, cos half the instructions are based on the person knowing what they're gonna do, so it seems to me." C166*
Participants realising it's easy to use	*"I thought I wouldn't be able to do it (laughter) but I can… I'm not really… I don't want computer or anything like that, you know, so it's only that only because of that." C228*
Using it weekly to develop skills	*"I was quite happy using it weekly, cos things can change so quickly…I'm a terrible, I'd forget when I'd done it the last time," C392*
Enabling others to help them use it	*"He's got used to it as long as I set it up." P217*
Seeing a purpose to use technology	*"It's unbelievable that sort of technology, but equally it's open to anybody to get into it. So, I might be persuaded eventually, but [laughs] it's a slow process" C166*
**Facilitators to use by those with disabilities**	
Stylus pen helped patients with upper limb disabled	*"Q: You mention you've got some problems with your hands. How do you find using your iPad?<br>P: It's a lot better since you gave me this [stylus] So I don't hold it like a button. Other than that it's great.<br>Q: Have you got any other gadgets like that?<br>P: No. [to C] You ordered some more because you liked it didn't you?" P172 [daily technology user]*
Touch screen is accessible	*"It's fine. I mean it's getting more awkward to me, for me cos obviously my hands are [weak], but, it's quite easy. Yeah, the touch screen, it's better than if you had to press this keyboard sort of thing." P056 [daily technology user]*
Use patients' own device	*"I think it'll probably have more of a take-up with people using their own device, because … I'm comfortable with my phone." P134 [daily technology user]*
Family/carers enter information on patients' behalf	*"Q: … is it something that you'd ask your carers to do or your family…?<br>P: Yeah, yeah, definitely.<br>Q: … would you have any concerns in them seeing your answers and that sort of thing?<br>P: No, I'd rather have the family do it rather than the carer do it, but I wouldn't have any problem, the family seeing it at all." P056 [daily technology user]*

Appendix table 5 Participants attitudes towards the frequency of TiM sessions.

**TiM session frequency**	
Weekly sessions acceptable	*"Maybe once or twice a week would be enough." C063 (Frequent user)*
Weekly sessions make it part of routine	*"I'll keep it to Tuesday and do it every Tuesday while I'm working at home and I'll have peace and quiet." P122 (Excellent user)*<br>*"I was quite happy using it weekly, cos things can change so quickly…I'm a terrible, I'd forget when I'd done it the last time and … doing it maybe on the thirtieth of every month, I'd forget…" C392 (excellent user)*
Varying frequency according to speed of progression	*"Q: How often do you think you'd need to do it to make it worthwhile…?*<br>*P: Probably every month.*<br>*Q: …and if things changed how would you then approach it, would you go back and do it again or would you wait for the month…?*<br>*P: If things were changing quicker I'd go back in and do it again…" P423 (Excellent user)*
Carers may do it less often	*"Maybe carers don't need to do it quite as often but … maybe you would ask people how often they wanted to do it and maybe they would start at once a month and then as things progressed they did it a bit more often" C378 (control)*

Hobson E, *et al. BMJ Open* 2019; 9:e028526. doi: 10.1136/bmjopen-2018-028526

Appendix table 6 Reasons for frequent and infrequent adherence to TiM identified during the interviews.

**Reasons for missing individual sessions**	
Forgetting to use the TiM	*"I can't stand on my own so C has to bring it in here because I don't like to do it when he's working. So then I forget." P172 (Infrequent user)*
Patient and carer not doing it together	*"I'll do it and just leave it on the arm of the chair or something and say, "You need to do it". I don't know if she has on not." P122 (High user)*
Holidays	*"Oh dead easy; I know we do miss some weeks, but we've been, you know, if we, if we're away and we don't take it with us, we leave it here." P317 (High user)*
Acute illness	*"We just didn't do it, he weren't fit enough to do it." C228 (Frequent user)*<br>*"...when he was not very well he just couldn't be bothered." C366 (High user)*
Technical difficulties	See Table 6.1 & 6.2
**Reasons for infrequent adherence**	
Poor health state at the start of the study	Reported in field notes made during calls with relatives.
Being too busy / having other priorities	*"It's just, with, with all the renovations and stuff it's sort of not become a priority at the moment." P134 (Infrequent user)*
Not receiving feedback and becoming demoralized with the TiM system	*"I have not received any feedback / contact from the STH MND team while using the TiM"* P047
No current need for MND services so not seeing a benefit of TiM to their care	*"Well there'd be no point, would there?" P317 (Infrequent user)*
Unable to access the TiM tablet out without assistance	*"I can't stand on my own so C has to bring it in here because I don't like to do it when he's working. So then I forget." P172 (Infrequent user)*
**Facilitators of frequent adherence**	
Using TiM on the same day each week	*"We always, always remember that it's on a Thursday." P392 (Excellent user)*
Phone alarm reminders	*"Q: If it sends you an alarm reminder, would that be an annoyance...?*<br>*P: No, it would probably be more of a trigger for me to do it" P134 (Infrequent user)*
Family members reminding them	*"Our grand daughter rings us at about 7 o'clock to remind us. Then we finish up doing it on a Thursday morning." P217 (Moderate user)*
Being able to use TiM on their own device	*"If it worked on my phone there would be no issue whatsoever, cos I do everything on there; I shop... I can control lights, heating, everything on the phone. I think if TiM worked on there then you would get your responses every week." P134 (Infrequent user)*

Appendix table 7 Participants' expectations of the potential communication and monitoring benefits of the TiM service.

**Potential benefits of the TiM identified by participants**	
**Improved communication with MND team**	
Increase speed of communication	*"If I put it on the tablet and I send it to you, you get it there and then. So if anything … happens to me in that period of time you know straightaway … So the quicker you can pick up on so, something it's better for you as a doctor as well as me as a patient." P091*
Increase frequency of communication	*"And you know somebody within the team is going to look at that information. So you are connected, once a week. So it's not every three months." C184*
Provides a direct connection with specialists	*"Well I'd read up about the telemed and I thought, well it gives you a direct connection with Sheffield and the team. I know it's an IT link but it's a definite link because once a week you are communicating with a team." C184*
Improve communication and liaison with other members of the MDT	*"Well I can't see there's a different way to what we're doing now; I'm quite surprised that you're all in communication really….I didn't know you spoke to [community MND team], and I didn't that [GP] and you and the hospital were quite so connected, so that seemed to be quite a positive thing." P354*
**Increased monitoring**	
To identify problems quickly	*"It just seemed a way of being able to communicate with, with my care team and letting them know on a regular basis how you're doing; and no doubt at the end of it (…) it flags up that I need help or I don't need help." P134*
To identify problems between clinic appointments	*"I thought it'd be a good idea for both of us, but mainly for P and, because it is a long time between the hospital appointments, and I thought well it's a good idea to monitor symptoms if they crop up out of the blue and we're not sure whether it's anything..." C062*
Enables a better understanding of the disease	*"When, when P started off with the home journey we, it was obviously dizzy spells…. So I said well we'll just keep a diary of what's happening so you yourself know what's happening, so that's what he's doing, and in effect, this is what, your telehealth could do eventually is sort of monitor people…" C091*
Enable self monitoring	*"So we can monitor ourselves as well at the same time. So it's helping you in the long run as well…I think you've gotta monitor yourself so you know how, what you can do and what you can't do. It's, you can't just rely on everybody on the end of a phone, pick up and say, you know, I've got this, that and the other. You've gotta look at health and safety for yourself as well, for whether you can walk or whether you can run or whether you can open a door or open a lid or carry a glass or whatever, you know, at the end of the day that's monitoring yourself, so you've gotta do it." P091*

Hobson E, *et al*. *BMJ Open* 2019; 9:e028526. doi: 10.1136/bmjopen-2018-028526

Appendix table 8 Participants' expectations of the TiM service.

Potential benefits of the TiM identified by participants	
**Psychological benefits**	
Clinicians take an interest	*"Somebody's taking an interest, somebody's wanting to know" C184*
Reducing isolation	*"You're not going to feel isolated." C184*
Reassurance	*"Sometimes it's just the fact that, "should somebody know about this?" or "should you be telling somebody that." Wouldn't it be nice if somebody knew this. Just small details that you sometimes think, "Does it make a difference if somebody knew about it?" And that telemed makes that difference." C184*
**Improve accessibility of the MND service**	
Reduce clinic appointments	*"If you can glean enough information of what I send through so I don't have to come to the hospital, I think that's a positive" P354*
Provide access to MDT when unable to travel to clinic	*" We just thought it would be quite good cos not everybody can get into clinic and thinking about the later stages, but it's still important to monitor how things are going" P480*
Reduce travel time	*"that would then cut down my travel time to clinic, you know, clinic doesn't really fulfil a great deal in my life other than ticking some boxes and sort of knowing where the progression's going but, you know, I think most of that now can be done through this server, through TiM." P134*

Hobson E, *et al. BMJ Open* 2019; 9:e028526. doi: 10.1136/bmjopen-2018-028526

Appendix table 9 The acceptability of the TiM questions.

**Acceptability of the TiM questions**	
Patients wanted the TiM to include questions about all aspects of MND	*"I've found the questions, I think they're all relevant. And, … it might be in the future when things do, if things do get worse it might be I might need those questions to highlight things and that's quite, quite comforting." P423*<br>*"There's one question about you use a feeding PEG. I don't at the minute so that's not relevant to me at the moment. But obviously as time goes by it, it will be. I think I just put, "no", I don't use one... But I think it should be on there because, … in time I probably will be using one." P145*
Emotional wellbeing questions were welcomed	*"I think it's important to find out, not just physically, how a disease affects somebody but how, emotionally, mentally it affects them. As a say, I'm quite a positive person, but, I have some off days." P122*
Carers happy sharing their sensitive information	*"Q: The sort of questions that they're asking you, quite, they're quite personal questions, aren't they...? How you feel about sharing those?*<br>*C: No problem." C392*
Questions made patients think about the consequences of their disease, but this was not a problem	*"Q: How does it feel having to sort of be faced with that question [about gastrostomy tubes]?*<br>*P: … when I first did that it was dead easy cos I just put "no" … and just moved on. But since then, since we've been to this Advance Care [planning] thing. ...And that, that sort of brought it to the fore …. it's a thought that I wouldn't really wanna think about, but you've gotta think about, so I think on that one, is that something I shall maybe ask more to get more advice on." P248*
Reporting a deterioration on the TiM was not distressing	*"I think: am I getting worse? But, yes, am I getting worse…. I don't think any of the questions are really frightening. You know it might happen." P172*<br>*"in a sense it's good that you can change, you know, the, keep every, everybody up-to-date. Cos you don't know when things are going to change anyway, do we, we don't, you know, it's just, it's like a bit of a, a grey area isn't it? … as long as people who need to know see that information then it's … not waiting till your next visit at the clinic is it...?" P122*
Wanting questions that assessed how they were coping	*"Maybe if they say, "No, I've not been out for a fortnight", that it makes them think: well, actually why haven't I? And it would highlight that person's not getting out of the house and not doing something themselves and they are continually there. Do they need a bit more support?" C122*
Questions were repetitive	*" It's repetitive. … I pick it up and I think oh God, here we go again, same old, same old. [I] put a couple ...more different ones in,… but no, the questions are right..." C145*
Questions become irrelevant if disability severe	*"As I am unable to use my arms and legs at all, many of the questions have become irrelevant to me beyond the first time of using it."* P047

Appendix table 10 The validity / accuracy of the TiM questions.

**The validity/accuracy of the TiM questions**	
Patients want answers to be accurate	*"If I've got a form I like to fill it in as accurately as possible." P248*
Patients were concerned that their answers would be misleading	*"I have lost weight [a few weeks ago] but I haven't [lost weight in the last week]. The way I'm reading it now… sounds like I'm still losing weight, well I'm not…I wouldn't like 'em to think looking at it, or you looking at it in Sheffield and thinking "God, he's still losing weight" P248*
The answers are insufficient to reflect small changes	*"Sometimes they answers to chose are too far apart so if the answer is one day a week, I answer no, or several, it's nearer several. So it's accurate but it's wide." P172*<br>*"The questions are quite in a narrow band, and because it's a slow-burner not much changes unless there's a step change, such as …the chest infection the other week." P354*
The condition varied from day to day making weekly assessments difficult	*"I thought: if I was doing the questionnaire on that day, "what sort of assistance do you get from your family? The answer would be off the scale somewhere. I thought: some of the questions don't quite fit the answer. So I try to put the most representative" C172*<br>*"I think some of the questions are a bit too general and wide rather than, for instance… what can I not do with my hand that I used to do, and how am I dealing with it." P166*
Informal care requirement questions were difficult to answer	*"The margins are too wide. One of the questions is, how much time do you spend in the day looking after her? And, there's a sort of, there's 3-4 hours. But I don't even look after myself for 3-4 hours, I just potter around. That's always gonna be the same answer." C172*
Some questions contradicted each other	*There are some questions on there that are bit ambiguous for me [for example] being able to use the stairs. Well I can't use the stairs but I still have a bathroom upstairs so I still walk from the top of the stairs to our bedroom." P134*
Patients wanted to provide more information to clarify their answers	*"… if you have any more questions to ask you've got that… availability to ask [the MND team] if you have any problems…" P056*<br>*"If you want to ask anything maybe you could type it in instead of it just being yes/no" CaT Patient 317*<br>*"I think that, that would be a good addition. Because you, you could answer a question and at the end of the section just put in, and just expand on what, why you've answered that, if you see what I mean? I think a comments box would be a good idea." P122*
Same problems identified every week	*"It's a probably the same answer I give every week… It says; "Do you stumble or feel that you fall, or have you fallen?" And I do, every single week, I guarantee that" P122*
Answer affected by other health problems	*"So at the minute I'm slightly doctoring the answer; so I'm saying I can dress myself but I can't, but it's not the MND." P381*

Appendix table 11 Participants understanding of the TiM service.

**Aims of the TiM service:**	
Monitoring	*"For you to be aware of it, and monitor it..." P402*
Relay information to the MND team	*"It shows how you're feeling which gets then relays to the nursing staff. If you've any questions you've got the item on it where you can ... ask that as well... I would think... it's read, isn't it, and then probably recorded somewhere." P056*
Look for trends	*"I would say they are probably going on to some sort of graph that detects a trend. I mean with me, you wont get a trend because sometimes you are up and sometimes I'm down. It'll look like a [gestured up and down]. But I would imagine the ideal situation is when you can detect a trend but that does depend on what's happening." C172*
Provides trends and alerts to problems	*"I'm guessing that it comes to someone's desk and that they're able to see a chart... from week to week, and I would imagine that there would be a series of red flags for you or some traffic light system... that red's intervention required, amber's a warning, green's get on with it.." P134*
Prioritise patients in need	*"I could see the reason why you were doing it; I realised that all the data was going to be collated and you can see then at a glance... you can see the statistics and everything, and it would highlight to you... if I had a dramatic change." P317*
Data would be used for research	*"I didn't realise that that's what was going to happen, I thought it was just being used for research." C392*<br>*"I would assume it goes to some databank somewhere and they try and correlate the answers I've given with, perhaps somebody else ... to see if there is any common ground."P122*<br>*"I imagine it goes onto a database ... and then if yourself and [Professor] are ever doing any research they may be able to...or if there's any trials you might be able to select from that who would be suitable for that trial." P 317*
Both clinical care and research	*"I imagine it goes on a survey of all the people you're doing to compare us with each other, and also to follow my own progress." P166*
**The role of the Telehealth Nurse**	
TiM Nurse looked at data	*"It shows how you're feeling which gets then relays to the nursing staff, doesn't it." P056*
EH was looking at data	*"I didn't realise that [Telehealth Nurse] was involved and she would ring us if our answers drastically changed, cos obviously they've stayed very much the same. Then I'm quite encouraged by that, I just presume that you do it yourself...and if you'd got a problem you'd ring [Telehealth Nurse] up for a bit of help." C392*
Unsure who looked at data	*"I'm guessing that it comes to someone's desk and that they're able to see a chart" P134*<br>*"The ones that have spoke about it to me I don't think they realise that I'm seeing their responses." Telehealth Nurse*

Hobson E, *et al. BMJ Open* 2019; 9:e028526. doi: 10.1136/bmjopen-2018-028526

Appendix table 12 Nurses' experience of using the TiM clinical portal. All quotes are from the Telehealth Nurse.

**Using the clinical portal**	
Easy to use	*"The training that I've had from seeing it this side was brief from Esther and it's, it's never, it's not difficult so it, I find I, right from the beginning I found logging on has been the most difficult thing, so; but once you're on finding your way around it is very, very easy, very easy,"*
Low burden	*"It only takes minutes"*
Easy to understand the flag system	*"If everything's OK it's green, … [if] there's maybe some elements that might need to be looked at, it's an orange or yellow, and then if there's an alert it's a red one."*
IT support for passwords required	*"My main problem was me accessing it to begin with, and that was very problematic. It's just asked me to change me password."*
Nurse didn't know how to use the patient TiM app or resolve technical problems	*" N: I have had a couple of phone calls, cos [the participants have] got my contact number through this, and they've rung with a problem, usually a logging on problem and…*<br>*Q: Forgotten their password?*<br>*N: Yeah…I've asked Esther, to be honest, because I'm not familiar with their device."*

Hobson E, *et al. BMJ Open* 2019; 9:e028526. doi: 10.1136/bmjopen-2018-028526

Appendix table 13 The accuracy and sensitive of the TiM information. Quotes are from the Telehealth Nurse (N) unless otherwise stated.

**Accuracy of the TiM information**	
Information accurate to help make some decisions	*"N: [EH] has led me to believe that it's quite easy to red flag on the carer. So this chap is triggering ... [reads the PHQ4 questions] So I mean ... the four that are triggering, they're all about...<br>Q: Worry, stress?<br>N: Yeah, thoughts: yeah. I know how poorly this lady is, I know how disabled she is, and I do know him, and when you speak to him he's very blasé about it all, which actually is more of a worry."*
Information in TiM wasn't sufficiently detailed enough to be sensitive	*"N: ...I think if they're routinely sending us an update every week I think you're more likely to pick up on problems, but I don't... I thought that would be so.<br>Q: Is that, do you think that's happened?<br>N: No not really. I don't know that the questions are sensitive enough, and I suppose the thing is that it might, if it triggers a contact phone call then you may well pick up on things sooner." (early interview)*
Needed more information	*"N: It's almost like you need a two-way thing (laughs) you need to ask them a question, [for example] how long have you been coughing... are you bringing anything up when you're coughing... you know, that type of thing... have you had a temperature... it's almost like [laughs] you need a two-way communication, cos this is just a snapshot, isn't it..."*
Discussions are required to fully understand a problem	*"I think some of the problems with patients using NIV, very specific problems, you only pick up from a conversation with them. I don't think you pick it up on the Tele, cos it's [the way] it's structured, it might show a problem but not a specific problem."*
An additional comment box would help gain more detailed information	*"Q: Yeah. Do you have, would you have time to read the comment boxes?<br>TW: I would hope so. You'd have to make time. If it was a problem that was coming up all the time, yes, I would hope that that; in some ways that might save you time."*

Appendix table 14 Participants' experiences of how the TiM impacted on their care.

**Impact on if TiM on patient care**	
Improves knowledge	*"Because since I've done it I'm taking more notice of my weight but I didn't before so I know it very carefully. It made me aware of that." P172*
Close monitoring	*"I'm certainly not left alone for more than a month, ever…Sheffield is there if I really need them, but on the other hand [local team] is here." P166*
Reassurance to know being monitored	*"I think the benefit to P is real. Because… somebody is there on hand looking at things… Because it's slow with P and he doesn't need as much attention and care, it's easy to feel detached from any positive interaction." C122*
Keeping in touch with specialist	*"…it's knowing that somebody else is in your corner." C423*<br>*Q: Do you think there might have been a point in your disease where those questions …were useful?*<br>*P: Nearer the beginning, definitely. I think, and I can only guess, that it must be the same for lots of long-term conditions; you know, I've lived with this for over five years now… you get to understand your own body and you know it's, it's normal for me now and for the first year no day was normal, no day looked like any other day in my life before that. So… if I could have camped in [MND consultant]'s house for the first six months I would have done, just so she was there, so I could say, but what about this, what about that; and you imagine symptoms, or I did, you know, you think God, this is happening and that must be related to the MND... So in the first year I would have filled that in every day, just to have that touch point," P047*
Nurse giving advice	*"When I came back on Tuesday last week and I did the second questionnaire, and within a day [Telehealth Nurse] saying "I've got a red flag on one of your answers." And it's the fact that I'd fallen twice while I was away on holiday and I'd put on it, you know: it said "have you fallen recently, how many times?" and I'd fallen twice while I was on holiday. So she phoned me, and said, "Are you ok? Is there a reason why you fell?" No, just my usual clumsiness....*<br>*Q: Were you expecting her to call?*<br>*P: No, I wasn't actually. It was just a bolt out of the view...I find that quite positive. It shows that the whole idea of it works.*<br>*Q: Has it changed your behaviour at all?*<br>*P: No. Not really." P122*
Nurse identifying problems	*"Q: [Telehealth Nurse] called, I think she spoke to you about when you fell...*<br>*P: Mm.*<br>*Q: ...what did you think about that when that happened?*<br>*P: It was useful wasn't it?*<br>*C: Well you weren't in so she spoke to me.*<br>*P: Yes, because I'd tripped over the bedroom chair…*<br>*C: that's right and, yes, that was the main thing, that's right, yeah. No, it was interesting that that, that had been picked up because we weren't, we don't know how it was picked up." P&C166*
Identifying problems between clinic visits	*"When I filled it in last week, and within a day [Telehealth Nurse] was phoning me. How much better could you have that? Instead of, two months down the line and I attend the clinic and they say "how have you been, have you fallen?" and I say "oh yeah I did: two months ago". See where I'm coming from? You've got that instantaneous contact with this technology that perhaps you don't have without it. So I'm a great believer in that, I'm a great believer in technology." P122*
Supporting important decision making	*"Q: The other question I had was that we kept an eye on your weight and I wonder whether you think that may have influenced your decision to have a feeding tube or not; do you think it, do you think it had?*<br>*P: [writing] I was frightened by the speed of loss of weight but was convinced how much muscle I lost." P409*
Help accept the disease	*" The questions nudged me to facing what I could do and not what I can't." P409*

Appendix table 15 The Telehealth Nurse’s attitudes towards the value of the TiM.

Quotes are all from the Telehealth Nurse

**The Telehealth Nurses’ attitudes towards the value of the TiM**	
TiM could be valuable	*“As a tool to aid the patient and then aid the nurse”*
To identify problems between clinic visits	*“A number of patients with MND and their carers will wait till a clinic, instead of contacting us with, if they’re, they’re worried about something, or there’s a change in their, you know, their condition, and I think if they’re routinely sending us an update every week I think you’re more likely to pick up on problems.”*
Alerting to problems earlier	*“Q: So it could have a use maybe in alerting you to earlier need for intervention?*<br>*N: Yeah, yeah.”*
Help monitoring trends	*“I mean the weights have been quite interesting, cos if they can use the weighing scales … that’s been quite interesting, so we can monitor their weight…. cos otherwise you wouldn’t necessarily see that variation.”*
TiM wouldn’t have a negative impact on the service	*“Q: do you think a patient will be concerned this is trying to take away a part of the service, or do you think the clinical team might feel it’s taking away part of the service?*<br>*N: I don’t think either side would. From a clinical side I think that anybody would be willing to make it as easy for the patient as possible.”*
Clinics are a burden to patient	*“I think from the patient’s point of view I think it becomes very burdensome, the travel into clinic, very much so…But certainly the, the travel and the amount of time and effort for them to come to clinic to sit in clinic to then go home again, it’s very difficult for them.”*
Patients do not see value in attending clinic	*“… [patients] sometimes say;* “nothing, I don’t get anything out of coming to clinic because you’re reiterating the same things, I know I’m getting worse…*” So there are some patients that don’t see the value of coming to clinic anyway. Now whether they would use a system like this and see the value of that I don’t know.”*
TiM could allow patients to be managed remotely	*“ I think it could. I think it’s one of those difficult things that at the moment, because it’s not something we systematically do. And they are attending clinic or I have connections with their community care team, so I am in touch with what’s happening and things are getting monitored. I don’t know.”*
Some problems need face to face assessment	*“Q: How reliable this kind of technology would be in deciding when or, when to cancel an appointment for a patient?*<br>*A: Oh I don’t know, I don’t know.*<br>*Q: Would you feel secure looking at that saying that patient doesn’t need to come in?*<br>*A: I think, I think it depends, so when I look at, if it’s a breathing problem and if I didn’t know them and they were having problems with their breathing …that would alert me and I would [think]: right we need… to contact, we need to be looking at this and monitoring it…I think it depends.”*

Appendix table 16 Participants experiences of clinician feedback.

**To act upon problems and provide feedback**	
Feedback reinforces the benefit of the TiM	*"I find that quite positive. It shows that the whole idea of it works." P122*
Not receiving feedback is demoralising	*"The emotional psychological depths that I've been to, I was putting the stuff in Telehealth and thinking but nobody's acknowledged this or contacted me about it, and I thought: well they're not going to because that isn't what the clinic's about, and that made me stop using it." P047*<br>*"...it's like all forms and all surveys... they say "your opinion is important please fill in the following..." and you say something that you think is absolutely dramatic and mind blowing and nobody comes back to you on it. And you think: well how important is that survey?" C172*
To provide feedback even if nothing had changed	*"Q: And would you expect perhaps someone to tell you what's going on with the tablet or the answers?*<br>*P: Yes. Because if I hadn't changed much, I would have thought I would have had some feedback."P172*
To acknowledge problems even if nothing can be done	*"[falls] ... knocks your confidence ...I probably were putting too much onus on Sheffield Hallam because (laughs) we've got this and there's not jack shit they can really do about this and we know that...*<br>*Q: But that kind of acknowledgement's quite important, do you think, of the, what happens ...?*<br>*C: I do, yeah, it's a bit of support, in't it, it's knowing that somebody else is in your corner." C423*
MND team couldn't solve their problem	*"Q: If your answers changed what do you expect of the MND service?*<br>*P: I don't, to be honest. Let's say if I thought I'd got a problem I wouldn't necessarily come to you because I don't think, I think, you know, I, the impression that it's medical to do with P, and if I have got a problem really; because when you flash up it does say if you, is to contact the, there is, so I would probably, I mean that's not part of your remit, is it, me really if I've got a problem?" C381*<br>*"[emotional support] That's not what that clinic's about, that clinic's about physical wellbeing and physical health... truthfully I thought I don't know why these questions are in here, because that clinic isn't equipped to deal with that." P047*
Feedback thought to be pointless if nothing can be done	*"But then again, what's the point of coming back if you can't say anything?" C172*

Hobson E, *et al. BMJ Open* 2019; 9:e028526. doi: 10.1136/bmjopen-2018-028526

Appendix table 17 The mismatch between the participants' and nurses' expectations of the TiM service.

**Mismatch between patient and nurse expectations**	
Patients expected contact if they experienced important problems:	
Falls<br>Chest infections<br>Dysphagia	*"Q: ...do you, do you think they're important things that she needs to know about?<br>P: I would say yeah… cos one of the questions is have you fallen or tripped? Obviously if I am falling more often, (well I am, I fell a few weeks ago, didn't I? but that, that's first time in months...). If I was falling … over every week I would say that, yeah, she needs to know something like that, or if I'm starting to not eat me food properly, if I'm starting to cough, I think obviously she needs to know that" P145*
Emotional difficulties	*"Most of my problems at the time are emotional, and the TiM does not appear to trigger any intervention." P047*
Problems may not be seen as important to nurse	*"She red flagged that she'd fallen, which is quite a common occurrence on a lot of patients, and I don't particularly worry unless they've been very, very well and then suddenly." Telehealth Nurse*
Problems may not be seen as important to patients	*"Q: When you put that you had fallen, what were you expecting to happen?<br>P : No. No.<br>Q: And when you heard that [your OT] had heard about it, what did you think about that?<br>P: Nothing really.<br>Q: And, do you think in the future, if something were to happen to you, and you put on here, what would you expect to happen, what would you like to happen?<br>P: I would think [Telehealth nurse] would ring. But the only thing is the over balancing, so far, isn't it." C217*
Problem may not be seen to be under the MND team's remit	*"[emotional support is] not what that clinic's about, that clinic's about physical wellbeing and physical health. To me, and in my own head, I thought it's never been about psychological wellbeing and your mental wellness and therefore; and truthfully I thought I don't know why these questions are in here, because that clinic isn't equipped to deal with that." P047*
Participants expecting contact when stable	*" It would be nice for someone to phone occasionally … particularly if anything changes.: C172*
Not expecting a particular action: expect nurse to decide	*"Q: And what would you expect to happen if you did change your answers like that week?<br>P: Not really, no, cos you know more than I do." P056<br>"I would tend to leave it to them to solve anything that needed solving." C217*

Appendix table 18 The problems associated with excessive TiM alerts. All quotes are from the Telehealth Nurse unless indicated.

**Excessive TiM system alerts**	
The same alerts appear every week	*"We have some of our patients that do this every time… They … cough or choke at least once a week."*
Alerts increase the time required to use the system and can cause frustration	*"Then when it was coming up every week… I knew I'd spoken to them about that [problem] and that was their choice. And I'd kept putting [a comment in the TiM notes] but you're sorta thinking; why do I have to keep putting it on every time?"*
Nurse can't control the alerts	*"If you know somebody's got a problem and they're not really trying to do anything about it, then you know that [alert is] gonna keep coming back every week; and I haven't been putting comments on all the time."*
Nurses may appear to not be acting on potentially dangerous problems	[1]*"[reads]* "Do you ever cough or choke on food?"*And then he's put:* "Occasionally", *and that's fine."*
Problems flagged even though the patient has chosen not to medical advice to avoid the problem	*"cos I know that patient and I know that they've chosen to eat and that it is problematic. But they've got a feeding tube and they should really be using their feeding tube, but they're [also eating]. Then when it was coming up every week, I knew I'd spoken to them about that and that was that choice?"*
Reporting the same problems without solutions could be demoralising to patients	*"Maybe to them the fact that it's the same thing week in week out, they've got an insight into that problem, it's not changing and, so they're not looking for something to help with it really."*
Repeated problems on which no action could be taken should be paused to avoid excessive alerts	*"Q: Are there any other, other things that you'd change at the moment to make it, to improve the system?*<br>*TW: Apart from the, the same red flags coming up every week with the same problem, having some way of either taking them off or say, or putting a comment in that, that would take it off by saying you've addressed this problem, it's, it's still gonna be there, it's not gonna change."*

Appendix table 19 The problems associated with excessive TiM alerts.

All quotes are from the Telehealth Nurse.

Nurse reaction to alerts	
Calls patient for more information	*"[I] ring the patient about it and ask them"*
Liaises with the community team to get more information	*"I try and get a bit more information, cos the, the community team may have been out and seen them and seen them face-to-face and have got something a little bit more useful back; so I have used it for that circumstance. And I'm in touch with the community team quite often, so sometimes if I'm ringing about something else I'll ask about one of the patients that's on here."*
Prefers to wait until clinic	*"I've seen somebody that was red flagged, cos they're coughing more..I've put a [TiM note] that we'll review in clinic next week."*
Will chose not respond to problems she thought were common in MND	*"She red flagged that she'd fallen, which is quite a common occurrence on a lot of patients. I don't particularly worry unless they've been very, very well and then suddenly. So if it happens over a few weeks and I've spoken to them and I know the situation…I'm not always alerted by that red flag."*
Using all the TiM information on the to make a decision	*"They were having problems with their breathing but I would look: are they on a breathing machine? No. That would alert me. So I think it depends."*
Patients and nurses prioritise different problems	*"I did the second questionnaire, and within a day the [Telehealth Nurse] calls saying "I've got a red flag on one of your answers." And it's the fact that I'd fallen twice while I was away on holiday… she phoned me, and said, "Are you ok? Is there a reason why you fell?" No, just my usual clumsiness….*<br>*Q: Were you expecting her to call?*<br>*P: No, I wasn't...It was just a bolt out of the view… I find that quite positive. It shows that the whole idea of it works." P122*
Having a relationship with the patient made it easier for the nurse to call the patient	*"I think it helps me that I know the patients. So I know this chap very, very well, I have a relationship with him and his wife; ringing up's quite easy to do, they wouldn't, they wouldn't be fazed by that. My response would be; "*oh there's been a red flag on the Telemedicine that you sent through, a message that you've sent through, and that's why I'm ringing"*. And that was quite an easy one."*
Harder to ring those who she didn't know	*"There are a small number of patients that I don't know, it just so happens that they've been very well through, so there hasn't really been any major alerts."*
Ringing carers may be difficult	*"If we've had a carer's response that's red flagging I don't always find that an easy phone call to make to a carer who's struggling."*
Patients not expecting her call	*"I rang a lady who I didn't know and she red flagged that she'd fallen… It was the lady's husband … he was quite shocked that I'd rung cos I didn't know them and I just explained about it, and he just said "No, it was just a little trip, she's absolutely fine, no problem."*
Nurse doesn't feel her calls benefit patient	*"I've said "Oh you've had some problem with this?...I know because you sent a Telemedicine and I'm the one that looks at the problems and sees what's what." And they said "Oh right, oh yes, it's nothing, it's fine." I don't know…"*

Appendix table 20 The future of the TiM.

**Preferred way to communicate with MND team**	
Modes of communication depends on the individual	*Q: How do you think is the best way for people contact you?<br>P: I think for me it'd be by phone, but again that will be depending on the carer and my speech. I mean I'm currently OK with the phone.<br>Q: And how do you feel about email contact or contact through the Telehealth?<br>P: Again that's fine by me, I use email, computer all the time, but again you'd have to judge that on the individual." P381*
Contact needs to be convenient	*"And phoning you up, I don't know about you but I hate phone calls: you are just settling down to have a nice cup of tea or a chocolate biscuit or whatever and the phone rings, you have someone trying to persuade you to change your heating systems" C172*
Alternative methods of communication add flexibility	*"I'm really happy ...communicating by email, because for me I can do it then in my own time, because I haven't got use of my hands and arms; so if I know that there's an email that I can read and then reply to in my own time, that's far more relaxed for me actually than the telephone... I can speak fine but the telephone, somebody has to hold it for me...if I'm not in exactly the right position it's not comfortable to take a r." P047*
Telephone may be uncomfortable for some	*"I feel a bit awkward on the phone, and you can't get across how you're really feeling on a phone anyway..I tend to get nervous when I'm on the phone and stuff and I forget what I'm saying to people." P423*
Dysarthria makes telephone hard	*C: [to P] Well telephone calls aren't that practical are they for you. Because if you are tired, I mean it's difficult to convey."C172*
Email may be impersonal	*"I think that's a bit impersonal email, you know, that, that's what I thought" C423*
Telephone useful for problems needing an immediate answer	*"If it's something that needs doing, dealing with here and now: the telephone, email is, is my favourite way of communicating, because it suits my condition.." P047*
Some subjects are better discussed face to face	*"I think it's not personal... I'd rather see somebody or talk to somebody than, than read about it on something on a screen." C366*
Happy to receive feedback in clinic	*" I realise that you are probably busy people, so I'm quite happy for someone to say, when we're down there, "oh by the way, your survey has altered and, you know, do you want to talk to you about it." C172*
Happy to wait for a reply as long as their message had been acknowledged	*Q: And if you put in a question... How long would you expect before you got an answer?<br>P: Well normally [Telehealth Nurse] gets back to me within a day or so. So I would expect two days, almost maximum.<br>Q: OK. So, reasonably quickly.<br>P: Even if, it was: "we've got your question :we're thinking about it"<br>Q: So, just an acknowledgement that you've email?<br>P: Yeah. It may be [Telehealth Nurse] can't answer me first time. Like the citalopram. What she said was: "I'll discuss it with, (I think) you" and then came back to me." P381*
Importance of respiratory monitoring	*"Q: What do you think, how do you think this would fit in around your clinic visits and the support you would get from [Telehealth Nurse]? If this was to become...rather than a research project, part of standard care, how do you think you would suggest using it?<br>P: Well I think in my case I would be happy to use that and lengthen the time between visits. I mean, the only difference to me is the breathing test." P381*
Happy to share the information with other members of the care team	*"Q: And is there, is there someone locally that you think would be, so that would be able to see your answers ...?<br>P: Well a good one would be the GP, wouldn't they? Or even the hospice, or, even the district nurses, probably more the district nurses, cos obviously they've got a regular visit now, haven't they?" P056*

Appendix table 21 The Telehealth Nurse's attitudes towards how the TiM might be used in the future.

Quotes are all from the Telehealth Nurse

**How would the TiM be used?**	
Delegating TiM duties may not be possible	*"I agreed to do it originally cos I said the thing is when a red flag comes if it's somebody else doing it and looking at it they're gonna come to me."*
The nurse wants to use her judgement, not follow a strict protocol	*"The way this study is run at the moment I can respond in, in the way I think is appropriate" Telehealth Nurse*<br>*"if it was written down, [that] I had to ring and I had to ring straightaway, … I don't know whether, I would have found that quite difficult not being able to use my initiative and how I'm familiar with the patients and, don't know I might not have, I might have found that a bit more difficult."*
Some nurse may be more willing to follow a protocol and call more often	*"N: It's difficult, I think you would probably get more useful information out of the research nurses, but you would have to have a system where they'd be able to go to somebody to act upon what was [needed]" Telehealth Nurse*<br>*"I think if it was a bigger study and.. it was a very … carefully monitored study, I think maybe the person doing this, looking at this … looking at what the replies have been, if they had to contact them that might work better."*

Appendix table 22 How the TiM might be used by other teams

**Use by community teams**	
Community teams would need capacity	*"I think if they have the capacity it would be useful," Telehealth Nurse*
It would increase information available for community teams	*"Q: Could [you] see [the TiM] fitting into the role you play or whether you think it's not very helpful?<br>N: I think it probably could because, like you've just said.. that [patient has] reported on here that she's … coughing/choking so many times but she never said that to me." Community nurse*
Community staff may not have access to computers	*"I'm not very good at computers. I have to say, that is the thing I find most stressful about working here. You I'm home based. I go on NHS website...so I'm not on, well I can get onto it but I do not cos I don't spend a lot of time [in the hospital]."Community nurse*
Community staff happy to receive information from Telehealth Nurse	*"Q: If someone else was looking at this, telling you there's a problem?<br>N: Yeah, no, that would be fine, that would be fine, yeah, yeah."Community nurse*
**Use by other MND centres**	
Other services would respond positively	*"I think their gut reaction is that that sounds something potentially helpful to us; I haven't really had any negativity" Telehealth Nurse*
Other services would want TiM if it saved them time	*"I think that if it saves them time I think that they would definitely embrace it." Telehealth Nurse*
Other services would want TiM if it benefited the patient	*"From a clinical side I think that anybody would be willing to make it as easy for the patient as possible, as long as it was useful." Telehealth Nurse*
Nurses would use it if it were part of their usual role	*"Q: But then if you were thinking about implementing this into the NHS who would you get the most useful information from?<br>TW: I think if it was implemented and it was part of somebody's role every day they would just do it, they would do it automatically; and, yes, I could envision that it would be very useful. So I suppose I'm a bit, it's half and half." Telehealth Nurse*

Hobson E, *et al. BMJ Open* 2019; 9:e028526. doi: 10.1136/bmjopen-2018-028526
