# Supplementary material for: Process evaluation and exploration of telehealth in motor neuron disease in a UK specialist centre
Source: BMJ Open. 2019 Oct 22;9(10):e028526. doi: 10.1136/bmjopen-2018-028526 (PMC6830641; doi:10.1136/bmjopen-2018-028526)
Supplement: Author's manuscript [file bmjopen-2018-028526.draft_revisions.pdf]

BMJ Open is committed to open peer review. As part of this commitment we make the peer review history of every article we publish publicly available.

When an article is published we post the peer reviewers' comments and the authors' responses online. We also post the versions of the paper that were used during peer review. These are the versions that the peer review comments apply to.

The versions of the paper that follow are the versions that were submitted during the peer review process. They are not the versions of record or the final published versions. They should not be cited or distributed as the published version of this manuscript.

BMJ Open is an open access journal and the full, final, typeset and author-corrected version of record of the manuscript is available on our site with no access controls, subscription charges or pay-per-view fees (http://bmjopen.bmj.com).

If you have any questions on BMJ Open's open peer review process please email info.bmjopen@bmj.com

BMJ Open

# Telehealth in motor neuron disease: a process evaluation and exploration of digitally-enabled access to specialist services.

| | |
|---|---|
| Journal: | *BMJ Open* |
| Manuscript ID | bmjopen-2018-028526 |
| Article Type: | Research |
| Date Submitted by the Author: | 16-Dec-2018 |
| Complete List of Authors: | Hobson, Esther; University of Sheffield, Sheffield Institute for Translational Neuroscience; Sheffield Teaching Hospitals NHS Trust, Department of neurology<br>Baird, Wendy; University of Sheffield, School of Health and Related Research<br>Bradburn, Mike; University of Sheffield, Clinical Trials Research Unit, School of Health and Related Research<br>Cooper, Cindy; University of Sheffield, Clinical Trials Research Unit, School of Health and Related Research<br>Mawson, Susan; University of Sheffield, School of Health and Related Research<br>Quinn, Ann; Sheffield Motor Neurone Disease Association Research Advisory Group, SITraN<br>Shaw, Pamela; University of Sheffield, Sheffield Institute for Translational Neuroscience; Sheffield Teaching Hospitals NHS Trust, Department of neurology<br>Walsh, Theresa; University of Sheffield, Sheffield Institute for Translational Neuroscience; Sheffield Teaching Hospitals NHS Trust, Department of neurology<br>McDermott, Christopher; University of Sheffield, Sheffield Institute for Translational Neuroscience; Sheffield Teaching Hospitals NHS Trust, Department of neurology |
| Keywords: | Telemedicine < BIOTECHNOLOGY & BIOINFORMATICS, Motor neurone disease < NEUROLOGY, Organisation of health services < HEALTH SERVICES ADMINISTRATION & MANAGEMENT, Adult palliative care < PALLIATIVE CARE |
| | |

SCHOLARONE™
Manuscripts

# Telehealth in motor neuron disease: a process evaluation and exploration of digitally-enabled access to specialist services

*Dr Esther V Hobson[1,2], Professor Wendy O Baird[2], Mr M Bradburn[2,3], Professor Cindy L Cooper[2,3], Professor Sue Mawson[2, 4], Mrs Ann Quinn[5], Professor Pamela J Shaw[1], Sister Theresa Walsh[1], Professor Christopher J McDermott[1]

*corresponding author

1 Sheffield Institute for Translational Neuroscience, University of Sheffield (SITraN), Sheffield, UK, 2 School of Health and Related Research, University of Sheffield (ScHARR), Sheffield, UK, 3 Clinical Trials Research Unit, School of Health and Related Research, University of Sheffield, Sheffield, UK, 4 NIHR CLAHRC Yorkshire and Humber, UK, 5 Sheffield Motor Neuron Disease Research Advisory Group, Sheffield, UK.

**Author email addresses**
e.hobson@sheffield.ac.uk
w.o.baird@sheffield.ac.uk
m.bradburn@sheffield.ac.uk
c.l.cooper@sheffield.ac.uk
s.mawson@sheffield.ac.uk
Ann Quinn: c/o e.hobson@sheffield.ac.uk
pamela.shaw@sheffield.ac.uk
theresa.walsh@sheffield.ac.uk
c.j.mcdermott@sheffield.ac.uk

**Keywords**
Telehealth, telemedicine, motor neuron disease, amyotrophic lateral sclerosis, qualitative, process evaluation

**Word count**
5914

For peer review only

## ABSTRACT

### Objectives
To evaluate the processes involved in using a novel digitally enabled healthcare system (TiM: Telehealth in Motor neuron disease) in people living with motor neuron disease (MND) and their informal carers. We examined TiM implementation, potential mechanisms of impact and contextual factors that might influence TiM implementation or impact.

### Design
An 18-month, single-centre process evaluation within a randomised, pilot and feasibility study.

### Intervention
TiM telehealth plus usual care vs. usual care.

### Setting
A specialist UK MND care centre.

### Participants
Patients with MND and their primary informal carers

### Primary and secondary outcome measures
Patient, carer and staff outcomes and experiences using semi-structured interviews. Descriptive data on implementation and use of TiM telehealth.

### Results
The TiM was acceptable and accessible to all 40 patients, 37 carers and staff. Intervention uptake and adherence were good: 14 (70%) patients completed a TiM session at least fortnightly. Barriers to TiM use (such as technology experience and disability) were overcome with well-designed technology and face-to-face training. Reported potential benefits of TiM included improved communication and care coordination, reassurance, identification of complications and the potential for TiM to be an alternative or addition to clinic. Benefits depended on patients current level of needs or disability.

The main challenges were the large number of alerts that were generated by TiM, how the clinicians responded to these alerts and the mismatch between patient/carer expectations and nurses actions. This could be improved by better communication systems and adjusting the alerts algorithm.

### Conclusions
TiM has the potential to facilitate access to specialist care but further iterative developments to the intervention and process evaluations of the TiM in different services are required.

For peer review only

## ARTICLE SUMMARY

### Strengths and limitations of the study

- This is an evaluation of a digitally-enabled care system that aims to provide accessible, specialist holistic care to patients with motor neuron disease and their carers.
- The study has provide an in-depth understanding of the processes involved in the new care service, the potential value of TiM and factors which are likely to influence its use, implementation and success.
- The study was based in a single care centre and used a small number of patients.

## BACKGROUND

Motor neuron disease (MND) causes progressive muscle paralysis, severe disability and eventually death within, on average, two to four years (1). Attendance at a specialist multidisciplinary clinics (MDC), is associated with improved survival and use of assistive aids, devices and proven therapies (2-7). Patients face many barriers accessing specialist services meaning many patients fail to gain the full benefit from these services (8). There is some evidence that digital-enabled technology care service, either telehealth (remote monitoring of patient symptoms and biomarkers) or video-consultations (telemedicine) can improve access to specialist care (9). However, no system can monitor the wide range of problems faced by patients with MND. In addition, few interventions offer support to informal carers who provide the majority of care to patients.

We employed a user-centred design process to develop the TiM telehealth system (10). This allows patients and carers to send information about their condition to the specialist care team (10). Early testing has already been conducted with a small number of potential users but further testing in the clinical environment was required (10). Telehealth services are complex interventions, consisting of different component parts and whose success relies on the context and individual behaviours of those using and delivering the intervention (11). Clinical trials of complex interventions such as telehealth face problems with patient and staff engagement and often lack of understanding of how the intervention is used, what works, and for whom (12-15). People living with MND and their carers face additional barriers to using digitally-enabled care due to their disability and age. The complexity of the disease and the holistic aims of care in this terminal disease mean telehealth systems will need to differ from more simplistic, target-driven care systems (such as blood pressure management). With these challenges in mind, the Medical Research Council recommend that development of a telehealth service is iterative with learning from development, piloting, evaluation and implementation informing each step (16).

### Aim of the study

We conducted a pilot and feasibility randomised controlled trial (RCT) in patients and carers of usual care plus TiM versus usual care. The results of the


For peer review only - http://bmjopen.bmj.com/site/about/guidelines.xhtml

pilot and feasibility trial are described in parallel publication. In this paper we describe a parallel process evaluation (16) which aimed to gain a deeper understanding of the use of the TiM system.

We aimed to:

- Explore TiM implementation, fidelity and quality, and the acceptability and feasibility of using TiM to deliver specialist, multidisciplinary care at a distance.
- Clarify potential mechanisms of impact (both intended and unintended).
- Identify contextual factors that might influence the implementation or impact.
- Identify, implement and begin to evaluate changes to improve the intervention.

## METHODS

### Study design

The methods of the pilot and feasibility RCT are described in a parallel publication. In brief, patients with MND and their primary informal carer currently receiving care from the Sheffield MDC were recruited and randomised to receive either telehealth plus usual care or usual care alone.

### Intervention

A detailed description of TiM has been published (10). The TiM Patient/Carer App contains a set of patient and carer questions assessing the disease progression, complications of MND, use of medical interventions and patient and carer wellbeing. The app was loaded onto a 7-inch Samsung Galaxy tablet with 3G internet. We asked participants to complete the questions weekly, more frequently if they desired. The results were sent using the internet to a clinical portal used by the MDC specialist nurse (the telehealth nurse) who had over 15 years experience of MND care. She could liaise with specialists in the wider multidisciplinary team (MDT) and provide telephone advice. All patients and carers continued to receive usual care, Sheffield MDC appointments and telephone access to the telehealth nurse. Clinic appointments could not be postponed but could be expedited. The nurse was required to have a discussion with the patient, carer or clinician to confirm the accuracy of the information prior to making any clinical decisions. Also loaded onto the tablet were educational materials (known as the Knowledge Centre) and within the app was a section where patients could make a list of issues the participant wished to discuss at clinic (the Problem List) and educational messages which appeared between questions.

EH recruited participants at home. Participants were given the tablet and wifi-enabled standing weighing scales. They were given face-to-face training, written information, telephone and email support. EH contacted patients after two weeks if they did not submit a TiM session and visited at one and six months for interviews which provided an opportunity to resolve technical issues. EH trained clinicians to use the clinical portal in face-to-face training, which took

For peer review only

approximately ten minutes. During the trial we gained feedback from users in order to improve the system. The trial started in October 2014 and one major change to the patient app and the clinical portal was implemented in October 2015.

**Data collection**

A detailed description of all data collection is described in the accompanying feasibility paper. To gain a deeper understanding of the processes involved we evaluated patient/carer satisfaction and adverse events. Semi-structured interviews were conducted by EH at baseline (control participants), one and six months (intervention participants). Interviews with the telehealth nurse were conducted at 14 and 18 months. Additionally an interview with an MND specialist community nurse who cared for a number of participants in both treatment arms during the study was conducted at 18 months. An online supplementary file contains additional supporting quotes. The topic guide included participants' experience of specialist MDC and community care, attitudes towards technology and digitally enabled care, expectations and experiences using TiM, reasons for good and poor TiM usage, potential impacts TiM could have on MND care, and improvements and future applications of TiM. MDC physicians completed a satisfaction questionnaire ("Shadow monitoring form") when they saw a patient in clinic using TiM capturing opinions on the accuracy, feasibility and acceptability of TiM. Field notes were also taken to describe any problems with TiM, solutions that were used and their outcomes. At the end of the trial we downloaded all data collected by TiM into Excel. This contained the time and date of every session, all answers provided by patients and carers, all alerts generated by the system and all clinical notes made by the telehealth nurse. We attempted to record the telehealth nurse's time spent using TiM using diaries but these were incomplete. Instead, this was explored using interviews.

**Patient and public involvement**

During development of the intervention and the protocol we consulted patients, carers and the Sheffield MND Research Advisory group (a patient and public involvement group). They reviewed the intervention, principles of the trial, trial design, outcome measures and participant information leaflets and provided comments on their feasibility and accessibility. They were not involved in recruitment. Results of the study have been communicated at various public meetings, through the Sheffield MND Research Advisory group and local branch of the MND Association and a lay summary will be circulated. Members of this group attended the trial steering and trial management groups. AQ was a member of the trial management group, provided advice on the research methods, interpretation of the data and dissemination and is a co-author on this paper.

For peer review only

## RESULTS

### Participants

40 patients and 37 informal carers (three patients had no carers) were recruited. Two TiM patients withdrew due to severe illness and one died before six months, the rest used TiM for between six and 18 months. Participant characteristics are reported in full in the parallel paper. The patients broadly represented those attending an MDC including patients at all stages of disease (Table 1). Whilst patients' use of digital technologies was common, some participants had little or no experience using technology. 38 (95%) patients had home broadband and 33 (83%) had mobile internet reception.

For peer review only


For peer review only - http://bmjopen.bmj.com/site/about/guidelines.xhtml

Table 1 Participant characteristics

| | **Telehealth (n=20)** | **Control (n=20)** |
|---|---|---|
| Patient gender, male | 14 (70%) | 14 (70%) |
| Age (years) Mean (SD), range | 60.4 (11.7), 30-78 | 60.0 (10.0), 39-73 |
| Disease duration (months) Mean (SD), range | 53 (48), 12-197 | 46 (35), 7-123 |
| King's ALS clinical stage[a] | | |
| 1 | 3 (15%) | 2 (10%) |
| 2 | 4 (20%) | 5 (25%) |
| 3 | 5 (25%) | 8 (40%) |
| 4 | 8 (40%) | 5 (25%) |
| Use of the TIM App | | |
| Independently | 17 (85%) | 17 (85%) |
| Help from carer | 1 (5%) | 1 (5%) |
| Patient instructs carer | 2 (10%) | 2 (15%) |
| Patient technology use[b] | | |
| Daily | 14 (70%) | 18 (90%) |
| A few times per week | 3 (15%) | 1 (5%) |
| Once a week | 1 (5%) | 1 (5%) |
| Every few weeks | 0 (0%) | 0 (0%) |
| Never | 2 (10%) | 0 (0%) |
| | **Telehealth (n=19)** | **Control (n=18)** |
| Carer gender, male | 4 (21%) | 5 (28%) |
| Carer age (years) Mean (SD), range | 59 (12, 42-84) | 60.8 (11,38-73) |
| Relationship to patient | | |
| Partner | 18 (95%) | 16 (89%) |
| Child | 0 (0%) | 1 (6%) |
| Parent | 1 (5%) | 1 (6%) |
| Carer technology use[b] | | |
| Daily | 12 (67%) | 16 (84%) |
| A few times per week | 1 (6%) | 0 (0%) |
| Once a week | 1 (6%) | 2 (11%) |
| Every few weeks | 0 (0%) | 1 (5%) |
| Never | 4 (22%) | 0 (0%) |

[a]King's stage 1 refers to patients with functional deficit in 1 domain, stage 2 refers to disability in 2 domains, stage 3 refers to disability in 3 domains and stage 4 refers to patients requiring NIV and/or gastrostomy. King's stage was calculated using the ALS-FRS-R scale at baseline. [b]Technology use: computer, tablet, smart phone. SD: standard deviation

For peer review only



For peer review only - http://bmjopen.bmj.com/site/about/guidelines.xhtml

**TiM technology set-up and delivery**

All participants allocated the intervention received the TiM system and could use it using mobile or home broadband. Technical problems and solutions were all resolved and had little impact on the trial except the wifi-enabled scales which were not found to be practical due to a lack of reliability connecting to the internet (Table 2). At six months all the patients and 14 (93%) of the carers felt TiM was easy to use (Figure 1). 14 (93%) patients and carers did not think the system was tiring and 13 (87%) did not think the questions were distressing, nor the use of the system intrusive.

*"It's so easy to do; it literally takes five minutes from home." Patient 317*

27 patients (68%) reported some difficulty using technology due to upper limb disability but 34 (85%) could use the patient app independently (although they might need a carer to help charge the tablet). One patient could use the app but during training it was noticed that his answers were not accurate (Patient 073). He was unable to communicate verbally and exhibited behaviours suggestive of mild cognitive deficits. In this case, he was happy for carers to help him use it.

Participants found face-to-face training important. Many were pleasantly surprised by their achievements.

*"I thought I wouldn't be able to do it (laughter) but I can…." Carer 228*

The main perceived barrier was participants' experience using technology.

*"…we're quite happy to deal with it, as long as it wasn't too techy" Carer 062*

Many participants (including those using technology daily) perceived themselves lacking an intrinsic ability to use technology, saying that they were "*bad*" at it, thinking others were more "*wired*" to using technology. They shared common attitudes towards their abilities, experience and approach to technology. They reported struggling to learn to use new technology finding it stressful and were fearful of making a mistake or breaking the device. They did not feel in control of technology, expressing frustration when technology did not "*obey*" them. This meant they were not confident adapting to unfamiliar technology. They tended to use technology for a limited number of basic tasks, lacking confidence to problem solve when technology *"went wrong"*, relying instead on others. Some low users explained they never learnt, or did not see the need to use technology, particularly if their partner used it.

One couple (Patient 166) had no experience of technology and were highly critical of the perceived intrusion of technology in their lives. They described themselves as *"technophobes"*. However, they trusted their care team would provide them with a secure and safe system. Only one carer in his 80s (Carer 217) struggled to *"catch on"* needing help from his partner. He had little prior experience with technology and it was later noted that he had difficulties with language due to another medical condition.


For peer review only - http://bmjopen.bmj.com/site/about/guidelines.xhtml

Table 2. Technology problems encountered during the trial

| Problem | Solution adopted | Impact on the use of the TiM | Recommendations for future TiM use |
|---|---|---|---|
| **TiM Patient App software** | | | |
| Poor finger dexterity | Handheld stylus provided. | 85% used TiM independently. | Provide stylus to all patients. |
| | Carer help to use device | Interview data suggested that help from carers was acceptable. | Encourage carer support. |
| Difficulties entering login password | Telephone support | Problem resolved with second TiM app release | Provide face-to-face training. Provide telephone technical support. |
| | Login page redesigned | No further problems reported. | Make local staff familiar with the software. |
| Lack of confidence using the app or other features on the TiM | Face to face training Partner/family helped | None: all participants could use the app. | Provide face-to-face training plus an additional contact after a few weeks to reinforce learning. Identify low confidence/experience users & provide extra training. |
| Patients not giving correct answers | Patient completing TiM with their family | Uncertain impact . | Capacity assessment at recruitment. Check TiM answers in training and in clinic. |
| **Tablet** | | | |
| Tablet stored in place not accessible to patient | No solution available. | Interviews suggested that adherence was reduced for two patients. | Use patients' own equipment where possible. |
| Tablet battery drained, unable to switch on | Telephone advice. | No impact. | Use patients' own equipment. Familiarise local staff with hardware. |
| Unexpected screens/ software updates | Telephone advice. | Reduced user confidence in tablet but no impact on use. | Use patients' own equipment. Use a basic tablet that only displays the TiM app. |
| User fear of "breaking" the tablet | Face to face training to improve user confidence | Patients/carers reluctant to use the additional features on the tablet | Use patients' own equipment. Face to face training. |
| **Internet connection** | | | |
| Poor 3G phone signal | Used patients' own broadband. | None: solution found for all patients (3G or patients' own broadband) | Use patients' own broadband. Check internet availability prior to TiM enrolment. |
| Patients switched wifi off | Home visit required. | Several TiM sessions failed to download. | Monitor adherence. Alert team if adherence is low. |
| Unreliable connection between scales and tablet/broadband | A separate 3G Wi-Fi route provided ("Mifi"). No solution if using broadband. | Additional home visits required and loss of weight data for several weeks. | Manual weight recording Avoid using peripheral devices. |
| **Clinician portal** | | | |
| Password/login problems | Support by external IT team. | Delayed access during MDC visit. | Local systems access support. |


For peer review only - http://bmjopen.bmj.com/site/about/guidelines.xhtml

**Participant adherence to TiM**.

Participants preferred to complete the TiM weekly (13, 87% of patients, 8, 53% of carers). They reported that weekly sessions helped them remember to use the TiM. Some suggested they would complete TiM less frequently if nothing had changed. The telehealth nurse suggested that fortnightly information was sufficient to enable her to assess a patient's progress, and even less frequently for carers. Adherence was calculated by dividing the total number of sessions completed by the number of weeks participants were in the trial (Figure 2 and Figure 3). 14 (70%) patients and 10 (55%) carers completed TiM sessions on average, at least fortnightly. 13 (70%) carers completed sessions, on average, at least every three weeks. Some participants who completed fewer sessions still continued to use TiM regularly. Adherence dropped during the study, but at 12 months 80% of patients were still using it weekly. The main reasons for low adherence were forgetting, holidays and deterioration in their illness. A lack of feedback about the answers participants gave was also a disincentive to use TiM, particularly when if they were expecting feedback.

*"It's like all surveys... they say "your opinion is important..." You say something absolutely and nobody comes back to you. And you think: well how important is that survey?" Carer 228*

The use of the additional features of the tablet was not monitored during the trial. Unlike the main TiM app, participants reported that they were less likely to use these although most were aware of these resources, particularly the short messages that appear whilst completing the TiM questions. Some participants reported reading some of the additional material (such as the Problem List and Knowledge Centre) but no one felt strongly that these had had a major positive impact with only half agreeing that they were useful (Figure 1).

**Participants' attitudes towards TiM**

14 (87%) patients and carers agreed that TiM questions were relevant to them and 12 (73%) patients and carers felt able to report all the problems they experienced using TiM (Figure 1). 13 (80%) patients and 12 (73%) carers would use it again and 14 (87%) patients and 12 (73%) carers would recommend it to another patient.

*"I think it's probably one of the best ideas to come out of the NHS for years." Patient 122*

Participants did not find using TiM intrusive or upsetting. They explained they were reminded about their disease everyday already and knew themselves if they had deteriorated without using the TiM.

*"It doesn't bother me. When I was first diagnosed I was not keen on talking about it. I've since got better and I think that helps. I'm quite happy to be reminded. I think you are reminded every day. That tablet makes no difference to that." Patient 381*

Some participants thought the questions were repetitive, particularly carers. Some noticed they reported the same problems every week. Some felt the questions were insufficiently sensitive to reflect the day-to-day fluctuation in


For peer review only - http://bmjopen.bmj.com/site/about/guidelines.xhtml

their condition. One carer suggested that the questions should use other ways to assess carers lives more critically, which could be used to encourage carers to consider their own wellbeing and adopt coping strategies.

**Clinicians experiences using TiM**

The telehealth nurse said that using the clinical portal was "*very, very easy*". taking *"minutes"* per use. She explained that initially she looked at the system every day but by the end of the trial she looked weekly, and sometimes a little less often. She would look at each patient but focused on new and changing alerts. She thought a green flag indicated everything was "*ok*", orange meant "*there's maybe some elements that might need to be looked at*" whilst a red flag indicated a problem.

*Safety and accuracy of TiM*

No adverse events related to TiM were reported and no participant reported delaying seeking medical attention as a result of TiM. 38 clinician satisfaction forms were completed when patients using TiM attended clinic. 20 (54%) were completed by the investigator (EH) who saw the patient in clinic, 18 (46%) were completed by other physicians. All agreed that TiM gave a useful and accurate picture of the patient and carer's condition and was a positive influence on the consultation.

**The reported potential impacts of TiM**

Reported potential impacts included improved monitoring and communication with the clinical team and improved effectiveness of MDC visits and care coordination. Other potential impacts included improved patient awareness of their disease and the ability to identify carer distress.

*Improving links with the clinical team*

Participants thought TiM could provide a "*direct link*" with the care team, increasing monitoring; providing better connection with specialists and enabling earlier identification of problems. Participants (particularly carers) also felt reassured that they were being thoroughly monitored. Reassurance was particularly beneficial for those who had infrequent contact with clinicians, either because they were early in the disease or were progressing slowly.

*"P… for the first year no day was normal… You imagine symptoms, … you think…that must be related to the MND…?*
*Q: Do you think there might have been a point in your disease where those questions …were useful?*
*P: Nearer the beginning, definitely…If I could have camped in [neurologist]'s house for the first six months I would have done, just so she was there, so I could say; "but what about this, what about that?"… in the first year I would have filled that in every day, just to have that touch point" Patient 047*

*Improved awareness of the disease*

Many participants thought that TiM improved their awareness of their condition. Whilst they felt that they did not need to track their progress using the system (they knew themselves how they were changing), patients become more aware


For peer review only - http://bmjopen.bmj.com/site/about/guidelines.xhtml

of their weight and nutrition. Rapid progression was something all patients feared so those with slowly progressive disease noticed that their answers did not change rapidly, making them feel more positive.

*Early identification of problems*

The telehealth nurse described TiM as "*a tool to aid the patient and then aid the nurse*". She could use TiM to gain more information and alert her earlier to problems. In one example, the TiM detected that one patient's condition was declining quickly and the carer reported high levels of strain. The couple was struggling to accept his decline and the patient tried to minimise his symptoms. TiM alerted the telehealth nurse to this patient's rapidly declining weight and bulbar function (Figure 4) She contacted him, discussed this information and encouraged him to consider gastrostomy insertion, which he accepted. He was asked about whether TiM affected this decision and explained that the information had been helpful:

*"The questions nudged me to facing what I could do and not what I can't...*
*I was frightened by the speed of loss of weight but was convinced how much muscle I lost. [sic]" Written quote Patient 409*

Other patients had reported problems on the app that they had not disclosed to their care team. The telehealth and community nurse speculated that this was because the patients were struggling to accept the change in their disease or need for treatment and might have felt that they can be more honest using technology. They found this information useful to gain a more complete picture of the situation.

*Improving the multidisciplinary clinic*

The telehealth nurse and physicians suggested that TiM could improve the MDC clinic. The telehealth nurse said if TiM could reduce the burden faced by patients attending clinic it would be welcomed by nurses. Potential benefits included facilitating sharing of information, collecting information and identifying problems before the visit could help plan the MDC visit to address the pertinent issues, shorten the time spent in hospital and make telephone appointments easier and a better alternative to face-to-face.

Following 19 (80%) appointments clinicians agreed that TiM could be used to make appropriate decisions remotely (in 4 (17%) they answered "*neutral*"). 14 (93%) patients said they would use TiM if they were unable to travel to clinic and 11 (73%) felt that they would be happy to have their appointment delayed if the doctor felt they were stable. Three (20%) were unsure and one (7%) disagreed. When interviewed, participants thought that the individual should choose how they used TiM depending on their needs, preferences and speed of progression. Some patients who were progressing slowly did not feel frequent MDCs were valuable. They were happy to use TiM and reduce the frequency of appointments as long as they could access the team quickly if required. Others wanted to attend the MDC to receive psychological support or address their problems and did not want to reduce visits. Some were not keen on telephone appointments, particularly when discussing sensitive matters or when they had


For peer review only - http://bmjopen.bmj.com/site/about/guidelines.xhtml

speech difficulties. The telehealth nurse explained face-to-face meetings helped her develop her relationship and gain information about the patient and carer. She felt some aspects of their care would need a combination of TiM system and communication with the patients/carers and other members of the MDT. Both clinicians and patients identified the need for face-to-face consultation to fully evaluate respiratory symptoms.

**Impact on carers**

Carers felt that their wellbeing was directly linked to the patients' wellbeing: interventions that improved the patient's life or carer's ability to provide care could improve a carer's wellbeing. Carers felt reassured that their loved one was being monitored and that problems could be identified and solved more rapidly.

*"I think the benefit to P is real. Because … somebody is there on hand looking at things...Because it's slow with P and he doesn't need as much attention and care, it's easy to feel detached from any positive interaction. Whereas with [TiM], you know somebody's there and if there was something you'd pick up quite quickly as apposed to, waiting until your next twelve week appointment" Carer 122*

Carers and the telehealth nurse reported that the information highlighted by TiM system could help couples accept the diagnosis and the problems they were facing, and enable them to look positively towards receiving additional medical and social care. Carers reported TiM prompted them to consider their own wellbeing and consider help if they were experiencing difficulties. They explained that usually the patient was present in meetings and healthcare professionals tended to focus on the patient's needs so it was difficult for them to discuss their own concerns because of the potential the impact this may have on the patient. Carers thought that TiM provided an opportunity for them to express their feelings in private, separately from the patient but still in the convenience of the home environment, without having to leave the patient. The impersonal nature of TiM was also an advantage as it enabled carers to be more honest about their difficulties.

*"I think it's a really good way of doing it. Because whereas I probably try and flower things up a lot of the time ...I found that, because it was just me and the tablet and I was able just to be totally honest about how I was… feeling at that particular time… The impersonal format… of the way it was actually done... for me, has been a real help just to be able… to do that." Carer 409*

**Problems with TiM**

Whilst the participants did react positively to TiM, many felt it had not had a significant impact on their care. At six months, only seven (47%) patients and five (33%) of carers agreed, *"The MND team contacted me quickly if my condition changed or I had a problem*". In some cases participants felt their condition had not changed during the trial so TiM had not highlighted any problems. In other cases, problems were reported and help was offered but declined either because the participant did not want it or because they did not think the MDT could help. However, the main explanation for this perceived lack of impact related to the large number of alerts generated by TiM, the way in which the telehealth nurse


For peer review only - http://bmjopen.bmj.com/site/about/guidelines.xhtml

acted upon information and lack the interaction between the participants and the telehealth nurse when problems occurred.

*TiM system alerts*

Of the total 585 TiM sessions completed, only 19 (3%) sessions were flagged as green whilst 322 (55%) of sessions alerts were red and 244 (42%) were amber (Figure 5a). We analysed individual answers and clinical notes for the ten randomly selected patients (334 sessions). Bulbar questions caused most red alerts (usually due to swallowing difficulties or excessive saliva), followed by limb function (usually due to falls) and respiratory symptoms. In the 334 sessions, 99 notes were made by clinicians. 32 (32%) notes reported the telehealth nurse taking action (Figure 5b): commonly providing telephone advice or liaising with the MDT to share information and coordinate care. 17 (17%) times she planned to review the problem when the patient next came to clinic. In over half of occasions the telehealth nurse documented that she took no action and in only 18 (18%) occasions did she contact the patient or carer. She explained that she usually took no action because she already was aware of the problem, the participant was awaiting treatment or had declined to accept the recommended treatment/advice. She felt that she was seeing the same problems repeatedly. This was particularly frustrating when she had already taken action or when she felt there was no action required. Whilst she did not feel TiM took up a lot of her time, these multiple problems for which she saw no solution caused an element of psychological burden. She could not control how the alerts were generated and would prefer to be able to "*pause*" these alerts.

*Patient-nurse interaction and feedback*

Some participants the telehealth nurse contacting them after reporting a problem on the TiM. The nurse would usually offer reassurance or new advice. Whilst the telehealth nurse felt that these interactions did not have an impact, in the face of a relentlessly progressing disease, these interactions were welcomed by patients and carers and it reinforced their perception that specialists were monitoring them closely.

*"...I did the second questionnaire, and within a day [Telehealth nurse] called.*
*Q: Were you expecting her to call?*
*P: No, I wasn't actually. It was just a bolt out of the blue...I find that quite positive. It shows that the whole idea of it works." Patient 122*

The telehealth nurse prioritised problems that were unexpected and placed less priority on problems she expected to occur in MND. Contacting the patient depended on whether the problem needed or had a solution.

*"She red flagged that she'd fallen, which is quite a common occurrence on a lot of patients, and I don't particularly worry unless they've been very, very well and then suddenly." telehealth nurse*

Problems the nurse perceived not to be significant were often felt to be important to patients and carers. For them, acknowledgement was helpful, even if no solution was needed.



For peer review only - http://bmjopen.bmj.com/site/about/guidelines.xhtml

*"[falls] ... knocks your confidence ...I probably were putting too much onus on [the hospital MDC] because (laughs) we've got this and there's not [much] they can really do about this and we know that...*
*Q: But that kind of acknowledgement's quite important?*
*C: I do, yeah, it's a bit of support, in't it, it's knowing that somebody else is in your corner."*
*Carer 423*

The telehealth nurse found it easier to manage patients and carers whom she already knew well. She developed relationships through hospital visits and a telephone helpline and through liaison with the community MDT. When the telehealth nurse had less knowledge of the patient it could be more difficult to interpret the information from TiM and liaison with the MDT was less easy. She also explained that she often did not know carers well and sometimes felt there were less obvious solutions to carer distress making her reluctant to telephone. In contrast, most carers felt extremely positive that the nurse provided vital counseling and advice.

The telehealth nurse felt that a more formalised protocol, outlining how she should respond to alerts, would likely increase the number of telephone calls made to patients. However, she felt it was important that she could use her knowledge and experience to decide how to respond to the information presented and would find it difficult working to an overly prescriptive protocol.

**Improvements made to TiM**
Additional questions were added to the patient app: these recorded bladder and bowel symptoms and recorded disease progression in those with more severe disability (using the ALSFRS-EX (17)). Participants felt it was very important that the information provided was accurate and sometimes wanted to give more information in their answers. A free text facility was added but this was used very infrequently. We added a heatmap (Figure 4) to the clinical portal which displayed a visual representation of patients' answers over the previous six months. We added the ability for the telehealth nurse to temporarily pause alerts. The telehealth nurse found the heatmap helpful to enable her to get an overview of things she thought were important, such as weight and to see which category had changed. She also found the ability to pause alerts helpful.

**Recommended improvements to TiM**
The most important improvement we identified was to facilitate communication between the nurse and participants. Most participants did not want automatic feedback on their progress from TiM: they could judge their progress themselves. They were also worried how they would react to seeing objective measures of decline without any interpretation from their clinical team. However, many expected some feedback. The minimum expected was to know that their information had been received and reviewed and for their problems to be acknowledged in some way, even if no action was taken. Participants were asked about their preferences for communication. Telephone and face-to-face conversations were felt to be important for sensitive or complex discussions.


For peer review only - http://bmjopen.bmj.com/site/about/guidelines.xhtml

*"I think it's not personal... I'd rather see somebody or talk to somebody than, than read about it on a screen." Carer 366*

Patients found email helped them communicate at their own pace. This was particularly helpful for those with speech problems where a phone call would be difficult. Participants were happy waiting for a definitive answer if they received an acknowledgement. Participants also thought TiM could be used to arrange appointments and other administrative matters and they were happy for their answers to be shared with other clinicians. The Community MND nurse felt TiM information would be useful but the clinical portal would be difficult to access in the community without a computer. She also explained she was *"not good with computers."* but would be happy receiving alerts and information from someone else.

**DISCUSSION**

This detailed study of a digitally-enabled MND service confirmed it was acceptable and feasible for patients, carers and staff. As this was a feasibility study differences in outcomes between the two groups were not formally assessed. However, our findings suggest the TiM was accurate and safe with no relevant adverse events reported. The study also gained a deeper understanding of the potential value of TiM and the context in which benefits may might be seen (18). We identified some key barriers and enablers to acceptance and use of telehealth which are applicable to other digitally enabled care systems. A key initial barrier for participants was their perceived ability to master the technology. However, uptake was good with most participants (even those with severe disabilities) finding that they were able to use the system and sustain a good level of TiM adherence. The interviews suggested that this was because of the accessible software and face-to-face training provided. This is in contrast to other telehealth projects which found acceptance and adherence were major barriers (19, 20). This study aligns with other evidence that suggests that patients have a positive attitude to digital products and, in the right setting, respond positively to digitally-enabled care, as long as the service is accessible and continues to offer personal contact with healthcare professionals (21). We recommend new interventions should adopt our user-centred design process and offer additional support to those without confidence in technology to make the intervention accessible.

The potential impacts of digitally-enabled care may differ depending on a patient's needs. TiM offered those with early or slowly progressive disease reassurance, information and the ability to remain in contact with the care team when they needed it as well as offering an alternative to clinic visits. Early identification and coordinated management of complications of the disease appears to be most valuable to those patients at a later stage of the disease or those with more rapidly changing disease. These factors may explain why MDC care improves survival. The study also highlighted the potential value of TiM to carers who could receive support separately from the patient, whilst still allowing them to fulfill their role as a carer. Patients and carers already use


For peer review only - http://bmjopen.bmj.com/site/about/guidelines.xhtml

digital technology to seek reliable and relevant information in order to self-manage (21) and the TiM can be used to provide information and promote self-management and self-efficacy (something which has a positive influence of patient quality of life (22)). This type of interaction also promotes good adherence to telehealth (20). It was therefore interesting to find that participants in this study did not use the additional education services on TiM that frequently. This may be because many patients wish to deal with problems as they occur, rather than learning extensively about their condition (21). Telehealth could address this by providing a more bespoke information resource with topics and pace appropriate for the user depending on the answers provided on TiM.

The main weakness of TiM was the mismatch between staff actions and the expectations of participants. This was due to lack of communication and high numbers of unnecessary alerts generated by TiM. MND progression is experienced as a series of losses (23,24) and it is important for staff to acknowledge these problems and recognise the value they offer to patients and carers. Remote technology risks disrupting the traditional way in which the nurse would gather information, develop relationships and respond to patient difficulties (25). There is a risk of care becoming less patient centre an patients becoming demoralised as the aims of patients/carers and the clinical team no longer align. We recommend care is taken to develop these interpersonal relationships and for staff to be aware of the potential pitfalls of remote management. Key improvements include improving the algorithms to reduce alerts and facilitating better communication between users (such as chat facilities, email or telemedicine).

The key strength of this study was the depth in which we examined the processes occurring in order to explain not just what happened, but why events occurred and how TiM could be improved and implemented. However, this was a small study involving only one centre. A maximum of 17 patients were using the TiM at any one time meaning larger studies are needed to gain a true understanding of what would occur when the service is offered to all patients and used as *business as usual* in different centres. The limitations of evaluating complex interventions such as telehealth in traditional trials are discussed in detail in the parallel paper. We recommend that future evaluations should retain the methods employed in this trial to understand processes in detail, and examine implementation in different contexts, continuing to examine factors such as acceptability, adoption, fidelity, safety, costs and sustainability.

## Conclusion

The TiM system was acceptable to patients and carers with good uptake and adherence. Key to its success will be continued user and staff engagement and ensuring the system complements the existing services. Improvements to TiM should be based on the findings of this evaluation. Future process evaluations should also examine the implementation of TiM at other sites to determine whether the findings from this study can be generalised.



For peer review only - http://bmjopen.bmj.com/site/about/guidelines.xhtml

**Funding and acknowledgements**

The TiM was developed within a collaboration between the University of Sheffield, Sheffield Teaching Hospitals NHS Trust, Mylan Ltd and Abbott Healthcare. This trial was funded by a National Institute for Health Research (NIHR) Doctoral Research Fellowship grant to EH (DRF-2013-06-076) and the Motor Neuron Disease Association. The trial was supported by the Sheffield Teaching Hospitals NHS Trust NIHR Clinical Research Facility and the University of Sheffield Clinical Trials Unit. Mylan Ltd supplied the software and hardware required for the trial. EH is also funded by an NIHR Clinical Lecturer award. PJS is supported by NIHR Senior Investigator award NF-SI-0512-10082 and PJS and CMD by the NIHR Sheffield Biomedical Research Centre (Translational Neuroscience) IS-BRC-1215-20017.

This paper presents independent research funded by the National Institute for Health Research. The views expressed are those of the author(s) and not necessarily those of the NHS, the NIHR or the Department of Health.

**Conflict of interest**

The TiM intellectual property is owned by Mylan and the University of Sheffield. There are no individual conflicts of interest.

**Author contributions**

The following were involved: intervention development (CMD, EH, TW, PJS), trial design and management (EH, MB, WO, CC, AQ, CMD), clinical oversight (CMD and PJS), recruitment and intervention delivery (EH and TW) data collection (EH, WB), data analysis (EH, MB, WB) and manuscript preparation (all authors). The authors are grateful to Sheffield MND Research Advisory Group for their guidance on the intervention and research methods.

For peer review only

1. McDermott CJ, Shaw PJ. Diagnosis and management of motor neurone disease. *BMJ*. 2008;336:658–62.

2. Traynor BJ, Alexander M, Corr B *et al.* Effect of a multidisciplinary amyotrophic lateral sclerosis (ALS) clinic on ALS survival: a population based study, 1996-2000. *J Neurol Neurosurg & Psychiatry*. 2003;74:1258–61.

3. Ng L, Khan F. Multidisciplinary care for adults with amyotrophic lateral sclerosis (ALS)/motor neuron disease (MND). *Cochrane Database Syst Rev.* 2009;13:105.

4. Aridegbe T, Kandler R, Walters SJ *et al.* The natural history of motor neuron disease: assessing the impact of specialist care. *Amyotroph Lateral Scler Frontotemporal Degener*. 2013;14:13–9.

5. Rooney J, Byrne S, Heverin M *et al*. A multidisciplinary clinic approach improves survival in ALS: a comparative study of ALS in Ireland and Northern Ireland. *J Neurol Neurosurg & Psychiatry*. 2015;86:496–501.

6. Martin S, Trevor-Jones, A, Khan S *et al*. The benefit of evolving multidisciplinary care in ALS: a diagnostic cohort survival comparison. *Amyotroph Lateral Scler Frontotemporal Degener*; 2017;7-8:569-575.

7. Moura MC, Casulari LA, Garbi Novaes MRC. Multidisciplinary Care Improves Survival of Patients with Amyotrophic Lateral Sclerosis in the Unique Health System (SUS) in Brazil. *J Neurol Disord*. 2017;05:1–7.

8. Motor Neurone Disease Association UK. Personal communication. 2016.

9. Hobson EV, Baird WO, Cooper CL *et al*. Using technology to improve access to specialist care in amyotrophic lateral sclerosis: A systematic review. *Amyotroph Lateral Scler Frontotemporal Degener.* 2016;5-6:313–24.

10. Hobson EV, O BW, Baird, WO, Palmer R *et al.* The TiM system: developing a novel telehealth service to improve access to specialist care in motor neurone disease using user-centered design. *Amyotroph Lateral Scler Frontotemporal Degener*; 2018;19:351-361

11. Medical Research Council. A framework for development and evaluation of RCTs for complex interventions to improve health. MRC; 2000.

12. Steventon A, Bardsley M, Billings J *et al*. Effect of telehealth on use of secondary care and mortality: findings from the Whole System Demonstrator cluster randomised trial. *BMJ*. 2012;344:e3874–4.

13. Fan VS, Gaziano JM, Lew R *et al*. A comprehensive care management program to prevent chronic obstructive pulmonary disease hospitalizations: a randomized, controlled trial. *Ann Intern Med*;




For peer review only - http://bmjopen.bmj.com/site/about/guidelines.xhtml

2012;156:673–83.

14. Takahashi PY, Pecina JL, Upatising B *et al*. A randomized controlled trial of telemonitoring in older adults with multiple health issues to prevent hospitalizations and emergency department visits. *Arch Intern Med*. 2012;172:773–9.

15. Moore GF, Audrey S, Barker M *et al*. Process evaluation of complex interventions: Medical Research Council guidance. *BMJ*. 2015 350:h1258.

16. Craig P, Dieppe P, Macintyre S *et al.* Developing and evaluating complex interventions: the new Medical Research Council guidance. *BMJ*. 2008;337:a1655–5.

17. Wicks P, Massagli MP, Wolf C *et al*. Measuring function in advanced ALS: validation of ALSFRS-EX extension items. *Eur J Neurol*. 2009;3:353–9.

18. Lamont T, Barber N, de Pury J *et al*. New approaches to evaluating complex health and care systems. *BMJ*. 2016;352:i154–5.

19. Sanders C, Rogers A, Bowen R *et al*. Exploring barriers to participation and adoption of telehealth and telecare within the Whole System Demonstrator trial: a qualitative study. *BMC Health Serv Res*. 2012;12:1–12.

20. Maeder A, Poultney N, Morgan G *et al*. Patient Compliance in Home-Based Self-Care Telehealth Projects. J *Telemed Telecare*. 2015;8:439–42.

21. Hobson EV, Fazal S, Shaw PJ *et al.* "Anything that makes life's journey better." Exploring the use of digital technology by people living with motor neurone disease. *Amyotroph Lateral Scler Frontotemporal Degener*. 2017;34:1–10.

22. Shir G, Irit H, Noa B *et al*. Hope and self-efficacy are associated with better satisfaction with life in people with ALS. *Amyotroph Lateral Scler Frontotemporal Degener*; 2018; 19, 611-618.

23. Foley G, Timonen V, Hardiman O. Exerting control and adapting to loss in amyotrophic lateral sclerosis. *Soc Sci Med*; 2014;101:113–9.

24. Mistry K, Simpson J. Exploring the transitional process from receiving a diagnosis to living with motor neurone disease. *Psychol Health*. 2013;8:939–53.

25. Greenhalgh T, A'Court C, Shaw S. Understanding heart failure; explaining telehealth – a hermeneutic systematic review. *BMC Cardiovasc Disord*. 2017;17,1-16

For peer review only

## Figure legends

Figure 1
Patient and carer satisfaction with TiM telehealth at 6 months.

Figure 2
A visual representation of individual patient adherence over the duration of the trial. Each black dot indicates a completed TiM session. A coloured dot indicates the end of follow-up.

Figure 3
A visual representation of individual carer adherence over the duration of the trial. Each black dot indicates a completed TiM session. A coloured dot indicates the end of follow-up.

Figure 4
The "heat map" for Patient 409. This is a screenshot from the clinical portal. The heatmap reports the highest level flag for each section of the questionnaire (mobility, bulbar, breathing, wellbeing, nutrition) at each week. This indicates the patient has red and amber flags for both bulbar and later mobility. Below is the weight compared to the patients' weight at baseline demonstrating a greater than 15% weight loss.

Figure 5
Figure 5a (left) The frequency of flags generated by the TiM sessions. The total sessions reports the frequency of "top level" flag (green, amber, red) for all 585 sessions completed. Below are the frequencies of "section flags" generated by 10 patients who completed a total of 334 sessions. 5b (left) Actions described in 99 clinical notes. P: patient, C: carer, TN: telehealth nurse

For peer review only

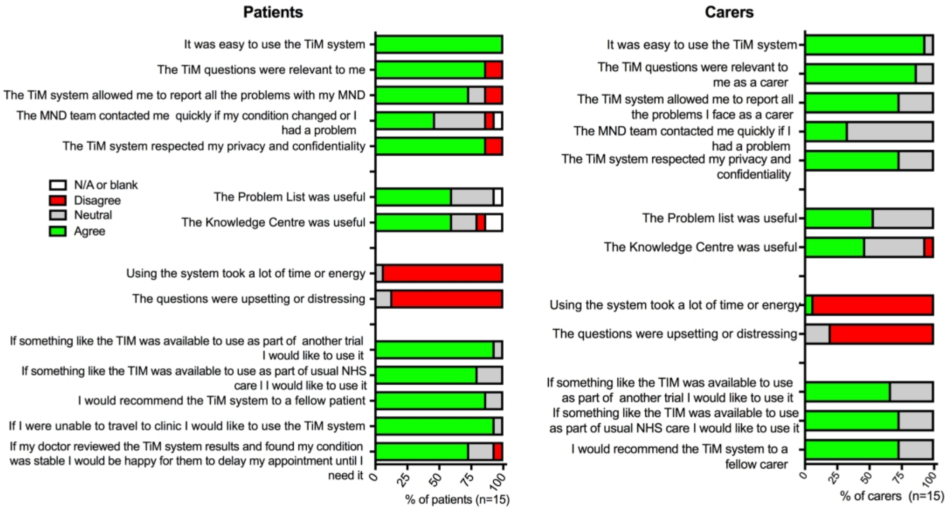


Patient and carer satisfaction with TiM telehealth at 6 months.

264x138mm (300 x 300 DPI)

| TiM ID | Age | Adherence |
|---|---|---|
| 423 | 30s | 104% |
| 166 | 70s | 99% |
| 381 | 60s | 97% |
| 392 | 50s | 93% |
| 228 | 70s | 91% |
| 122 | 60s | 85% |
| 145 | 40s | 83% |
| 366 | 70s | 77% |
| 248 | 60s | 73% |
| 217 | 70s | 73% |
| 56 | 50s | 69% |
| 409 | 60s | 65% |
| 63 | 60s | 60% |
| 354 | 50s | 53% |
| 317 | 50s | 47% |
| 19 | 50s | 41% |
| 172 | 60s | 35% |
| 134 | 40s | 22% |
| 73 | 60s | 22% |
| 47 | 50s | 13% |

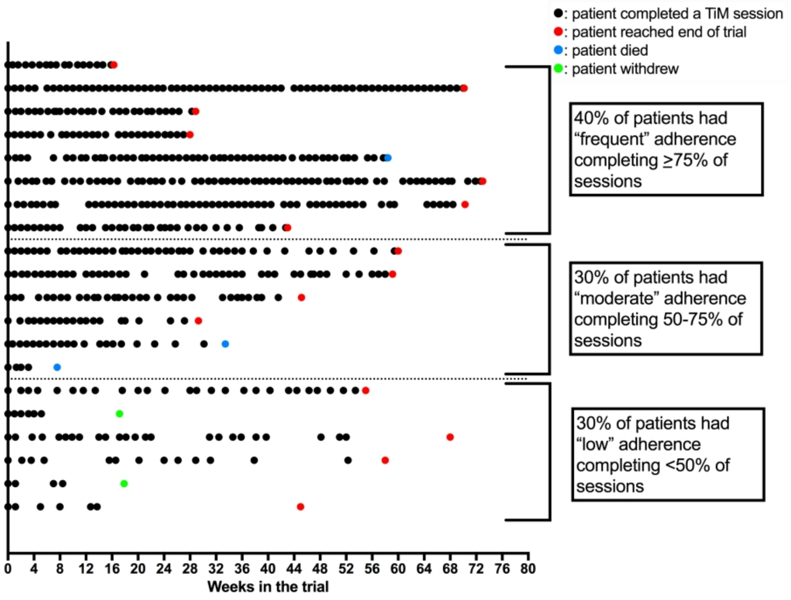


A visual representation of individual patient adherence over the duration of the trial. Each black dot indicates a completed TiM session. A coloured dot indicates the end of follow-up.

275x166mm (300 x 300 DPI)

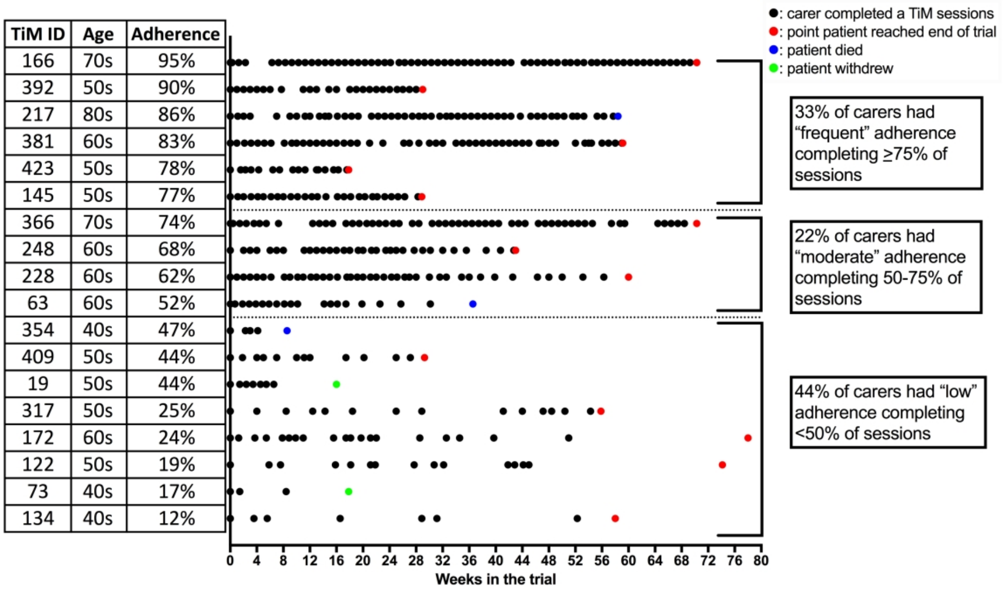

| TiM ID | Age | Adherence |
|---|---|---|
| 166 | 70s | 95% |
| 392 | 50s | 90% |
| 217 | 80s | 86% |
| 381 | 60s | 83% |
| 423 | 50s | 78% |
| 145 | 50s | 77% |
| 366 | 70s | 74% |
| 248 | 60s | 68% |
| 228 | 60s | 62% |
| 63 | 60s | 52% |
| 354 | 40s | 47% |
| 409 | 50s | 44% |
| 19 | 50s | 44% |
| 317 | 50s | 25% |
| 172 | 60s | 24% |
| 122 | 50s | 19% |
| 73 | 40s | 17% |
| 134 | 40s | 12% |



A visual representation of individual carer adherence over the duration of the trial. Each black dot indicates a completed TiM session. A coloured dot indicates the end of follow-up.

277x165mm (300 x 300 DPI)

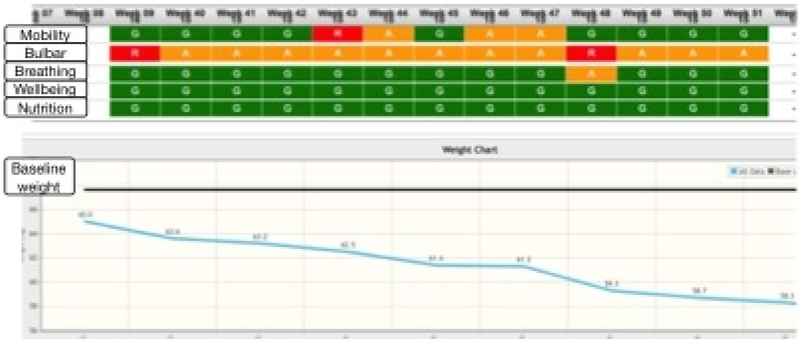


The “heat map” for Patient 409. This is a screenshot from the clinical portal. The heatmap reports the highest level flag for each section of the questionnaire (mobility, bulbar, breathing, wellbeing, nutrition) at each week. This indicates the patient has red and amber flags for both bulbar and later mobility. Below is the weight compared to the patients’ weight at baseline demonstrating a greater than 15% weight loss.

140x59mm (72 x 72 DPI)

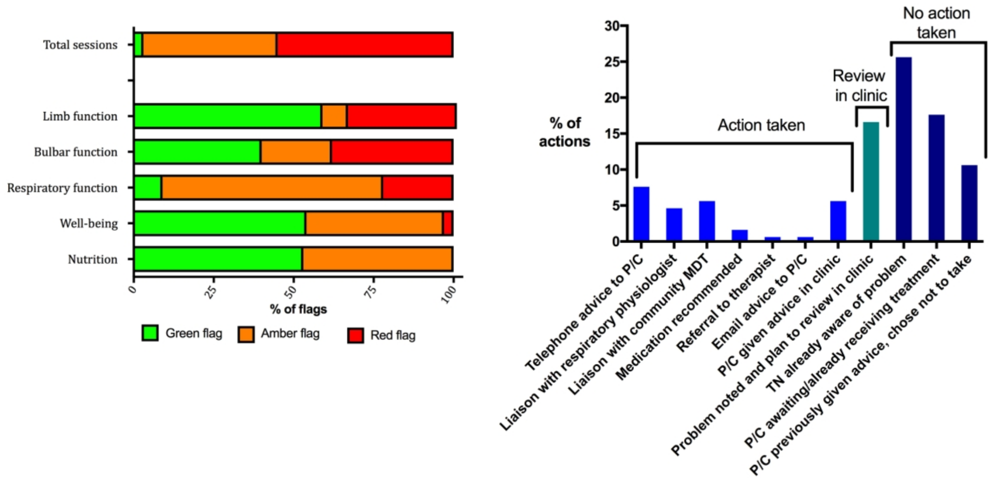


Figure 5a (left) The frequency of flags generated by the TiM sessions. The total sessions reports the frequency of “top level” flag (green, amber, red) for all 585 sessions completed. Below are the frequencies of “section flags” generated by 10 patients who completed a total of 334 sessions. 5b (left) Actions described in 99 clinical notes. P: patient, C: carer, TN: telehealth nurse

193x97mm (300 x 300 DPI)

**Telehealth in motor neurone disease to increase access to specialist multidisciplinary care: a process evaluation.**

**Supplementary data file**

## Table of Contents

Appendix table 1 Participants' initial impressions of using the TiM app. .................. 2
Appendix table 2 The characteristics of those lacking confidence in technology user and their approach to using technology. ........ 3
Appendix table 3 Negative attitudes to technology expressed by participants....... 4
Appendix table 4 Facilitators for TiM use. ........ 5
Appendix table 5 Participants attitudes towards the frequency of TiM sessions... 6
Appendix table 6 Reasons for frequent and infrequent adherence to TiM identified during the interviews........ 7
Appendix table 7 Participants' expectations of the potential communication and monitoring benefits of the TiM service. ........ 8
Appendix table 8 Participants' expectations of the TiM service........ 9
Appendix table 9 The acceptability of the TiM questions. ........ 10
Appendix table 10 The validity / accuracy of the TiM questions........ 11
Appendix table 11 Participants understanding of the TiM service........ 12
Appendix table 12 Nurses' experience of using the TiM clinical portal. All quotes are from the Telehealth Nurse. ........ 13
Appendix table 13 The accuracy and sensitive of the TiM information. Quotes are from the Telehealth Nurse (N) unless otherwise stated........ 14
Appendix table 14 Participants' experiences of how the TiM impacted on their care........ 15
Appendix table 15 The Telehealth Nurse's attitudes towards the value of the TiM. ........ 16
Appendix table 16 Participants experiences of clinician feedback........ 17
Appendix table 17 The mismatch between the participants' and nurses' expectations of the TiM service. ........ 18
Appendix table 18 The problems associated with excessive TiM alerts. All quotes are from the Telehealth Nurse unless indicated. ........ 19
Appendix table 19 The problems associated with excessive TiM alerts........ 20
Appendix table 20 The future of the TiM........ 21
Appendix table 21 The Telehealth Nurse's attitudes towards how the TiM might be used in the future........ 22
Appendix table 22 How the TiM might be used by other teams ........ 23


For peer review only - http://bmjopen.bmj.com/site/about/guidelines.xhtml

## Appendix table 1 Participants' initial impressions of using the TiM app.

| | |
|---|---|
| **Initial use of the app** | |
| Easy to use | *"It's so easy to do; it literally takes five minutes from home." P317 [daily technology user]* |
| Quick to use | *"It's not a problem is it? It takes minutes, it's not like you have to sit there for half a day and do it, it literally, it's, it's sort of minutes." P248 [daily technology user]* |
| TiM accessible and acceptable to the elderly | *"So this lady [P217], she's an elderly lady who you thought wouldn't have embraced anything like this.  But she has done and she, her and her husband both send it back; she's very disabled but able to use her hands quite well...they're both in their eighties; and she's a very good replier, sends it in." Telehealth nurse* |
| TiM accessible to those with significant disabilities | *"[There are] some are patients that, I'm surprised they took it up. I'm surprised; actually that, that, I never thought that he would use that... and he sends his back very, very well." Telehealth nurse* |
| Confidence the information would be held securely | *"I'm assuming that your department is one that's reasonably secure and has got reasonable standards... I can only make my judgement on the people I meet who are involved in it." C166* |

For peer review only

## Appendix table 2 The characteristics of those lacking confidence in technology user and their approach to using technology.

| **Characteristics of those with low technology confidence** | |
|---|---|
| Unsure if they would be able to use the TiM | *"…we're quite happy to deal with it, as long as it wasn't too techy" C062 [uses technology a few times a week]*<br>*"…as long as I can do it" P392 [uses technology daily]* |
| Lack of experience using technology | *"I don't use the internet for my job and it's all still paperwork orientated" C395 [uses technology daily]* |
| Difficulties with language | *"C: It's the system that's all. I don't catch on very well with it, that's ok. Like phones, I don't bother them really.*<br>*P: Couldn't read a text message.*<br>*C: couldn't do anything like that." C217 [never uses technology]* |
| Frightened they might make the TiM go wrong | *"Q: Have you used any of the other things that are on the tablet, so the education things or the website?*<br>*P: Oh I daren't touch all that stuff in case it goes wrong. (laughs)…I pressed that one day and I got a bit panicky (laughter) so I… left it…I just use it for the questionnaire now and that's it." P145 [uses technology daily]* |
| Frightened they might break it | *"…plus it doesn't belong to us and if we broke it we'd be devastated" C145 [uses technology daily]* |
| Frightened they will enter wrong information | *"My fingers are too heavy. … I'll end up phoning somebody." P378 [once a week technology user]* |
| Technology use is stressful | *"But I can… if I'm, if I'm taught …without aggravation." C380 [uses technology every few weeks]* |
| Problems using technology perceived as failure | *"I tried to order a book on it last week and I can't do it (laughs) it kept, I've lost passwords and all sorts…" C392 [uses technology daily]* |
| **Low user's approach to technology** | |
| Use technology for only a limited number of familiar purposes | *"I have used the computer for various things, and if I'm using it for something and I know what I'm doing, that's fine; I can get on for certain things now…" C380 [uses technology every few weeks]* |
| Avoids using unfamiliar technology | *"Q: And, and you say you were a little bit worried about the technical side?*<br>*C: Well I am, I was… I can manage things… I use Kindle and a laptop. Computers are a bit more of a thinker… (laughs) and…I don't do them all the time so I'm all right with basic stuff." C062 [uses technology a few times a week]*<br>*"I know basically how to navigate [his own device], whereas [the TiM] I don't." P145 [uses technology daily]* |
| Unable to problem solve: relies on others | *"Q: And what do you do if you get, get stuck on a problem?*<br>*C: Have to wait till me daughter-in-law comes up and get her to sort it out for me." C392 [uses technology daily]* |



For peer review only - http://bmjopen.bmj.com/site/about/guidelines.xhtml

## Appendix table 3 Negative attitudes to technology expressed by participants.

| **Negative attitudes towards technology** | |
|---|---|
| Lacking an interest in technology | *"It doesn't really interest me, if I'm being honest." C392 [uses technology daily]* |
| Not seeing a need for technology | *"I'm not against it but I don't want it and I can do without it and I don't need it." C166 [never uses technology]* |
| Feeling pressured to use technology | *"All the time we're being pressured [by people] that take all this marvellous technology up; and every day it is getting worse and worse, because it's getting beyond the control of the average person how to manage it properly." C166 [never uses technology]* |
| Feeling excluded/missing out if not using technology | *"I do feel…a slight social outcast, but I seem to be very much in the minority." C166 [never uses technology]* |
| Worried about the consequences of technology misuse | *"… certain things are very useful [but] once it gets to the mass market…it of gets exploded and misused. … You've got to the point of small businesses being blackmailed by hackers." C166 [never uses technology]* |
| Worried about online security | *"And as for banking online, God, it's a laugh, I would not touch it…if something goes wrong you have the devil's own job to try and put it right because there's no proof." C166 [never uses technology]* |
| Worried about intrusion into their privacy | *"I wouldn't have a Smartphone for a start, you know cos Big Brother's up there already." C166 [never uses technology]* |
| Previous negative experience using technology that was unreliable | *"…eventually these programs got more and more complicated and the time it was taking for sending information down and getting back was absolutely ludicrous; the thing would crash say at eleven o'clock on a Monday morning and you'd have to start all over again." C166 [never uses technology]* |
| Technology is an unwanted replacement for human contact | *"They can't pick up a phone normally anymore because they're so busy faffing about with their smart phones." C166 [never uses technology]* |



For peer review only - http://bmjopen.bmj.com/site/about/guidelines.xhtml

# Appendix table 4 Facilitators for TiM use.

| Facilitators for TiM use | |
|---|---|
| **Improving confidence using the TiM** | |
| Face to face training | *"I would take it up if I was shown that the program was spot on" C166* |
| Clear instructions | *"…That the instructions were aimed at an idiot like me… and not on the assumption that I would know what to do, cos half the instructions are based on the person knowing what they're gonna do, so it seems to me." C166* |
| Participants realising it's easy to use | *"I thought I wouldn't be able to do it (laughter) but I can… I'm not really… I don't want computer or anything like that, you know, so it's only that only because of that." C228* |
| Using it weekly to develop skills | *"I was quite happy using it weekly, cos things can change so quickly…I'm a terrible, I'd forget when I'd done it the last time," C392* |
| Enabling others to help them use it | *"He's got used to it as long as I set it up." P217* |
| Seeing a purpose to use technology | *"It's unbelievable that sort of technology, but equally it's open to anybody to get into it. So, I might be persuaded eventually, but [laughs] it's a slow process" C166* |
| **Facilitators to use by those with disabilities** | |
| Stylus pen helped patients with upper limb disabled | *"Q: You mention you've got some problems with your hands. How do you find using your iPad?<br>P: It's a lot better since you gave me this [stylus] So I don't hold it like a button. Other than that it's great.<br>Q: Have you got any other gadgets like that?<br>P: No. [to C] You ordered some more because you liked it didn't you?" P172 [daily technology user]* |
| Touch screen is accessible | *"It's fine. I mean it's getting more awkward to me, for me cos obviously my hands are [weak], but, it's quite easy. Yeah, the touch screen, it's better than if you had to press this keyboard sort of thing." P056 [daily technology user]* |
| Use patients' own device | *"I think it'll probably have more of a take-up with people using their own device, because … I'm comfortable with my phone." P134 [daily technology user]* |
| Family/carers enter information on patients' behalf | *"Q: … is it something that you'd ask your carers to do or your family…?<br>P: Yeah, yeah, definitely.<br>Q: … would you have any concerns in them seeing your answers and that sort of thing?<br>P: No, I'd rather have the family do it rather than the carer do it, but I wouldn't have any problem, the family seeing it at all." P056 [daily technology user]* |



For peer review only - http://bmjopen.bmj.com/site/about/guidelines.xhtml

## Appendix table 5 Participants attitudes towards the frequency of TiM sessions.

| **TiM session frequency** | |
|---|---|
| Weekly sessions acceptable | *"Maybe once or twice a week would be enough." C063 (Frequent user)* |
| Weekly sessions make it part of routine | *"I'll keep it to Tuesday and do it every Tuesday while I'm working at home and I'll have peace and quiet." P122 (Excellent user)*<br>*"I was quite happy using it weekly, cos things can change so quickly…I'm a terrible, I'd forget when I'd done it the last time and … doing it maybe on the thirtieth of every month, I'd forget…" C392 (excellent user)* |
| Varying frequency according to speed of progression | *"Q: How often do you think you'd need to do it to make it worthwhile…?*<br>*P: Probably every month.*<br>*Q: …and if things changed how would you then approach it, would you go back and do it again or would you wait for the month…?*<br>*P: If things were changing quicker I'd go back in and do it again…" P423 (Excellent user)* |
| Carers may do it less often | *"Maybe carers don't need to do it quite as often but … maybe you would ask people how often they wanted to do it and maybe they would start at once a month and then as things progressed they did it a bit more often" C378 (control)* |



For peer review only - http://bmjopen.bmj.com/site/about/guidelines.xhtml

## Appendix table 6 Reasons for frequent and infrequent adherence to TiM identified during the interviews.

| | |
|---|---|
| **Reasons for missing individual sessions** | |
| Forgetting to use the TiM | *"I can't stand on my own so C has to bring it in here because I don't like to do it when he's working. So then I forget." P172 (Infrequent user)* |
| Patient and carer not doing it together | *"I'll do it and just leave it on the arm of the chair or something and say, "You need to do it". I don't know if she has on not." P122 (High user)* |
| Holidays | *"Oh dead easy; I know we do miss some weeks, but we've been, you know, if we, if we're away and we don't take it with us, we leave it here." P317 (High user)* |
| Acute illness | *"We just didn't do it, he weren't fit enough to do it." C228 (Frequent user)*<br>*"...when he was not very well he just couldn't be bothered." C366 (High user)* |
| Technical difficulties | See Table 6.1 & 6.2 |
| **Reasons for infrequent adherence** | |
| Poor health state at the start of the study | Reported in field notes made during calls with relatives. |
| Being too busy / having other priorities | *"It's just, with, with all the renovations and stuff it's sort of not become a priority at the moment." P134 (Infrequent user)* |
| Not receiving feedback and becoming demoralized with the TiM system | *"I have not received any feedback / contact from the STH MND team while using the TiM"* P047 |
| No current need for MND services so not seeing a benefit of TiM to their care | *"Well there'd be no point, would there?" P317 (Infrequent user)* |
| Unable to access the TiM tablet out without assistance | *"I can't stand on my own so C has to bring it in here because I don't like to do it when he's working. So then I forget." P172 (Infrequent user)* |
| **Facilitators of frequent adherence** | |
| Using TiM on the same day each week | *"We always, always remember that it's on a Thursday." P392 (Excellent user)* |
| Phone alarm reminders | *"Q: If it sends you an alarm reminder, would that be an annoyance...?*<br>*P: No, it would probably be more of a trigger for me to do it" P134 (Infrequent user)* |
| Family members reminding them | *"Our grand daughter rings us at about 7 o'clock to remind us. Then we finish up doing it on a Thursday morning." P217 (Moderate user)* |
| Being able to use TiM on their own device | *"If it worked on my phone there would be no issue whatsoever, cos I do everything on there; I shop... I can control lights, heating, everything on the phone. I think if TiM worked on there then you would get your responses every week." P134 (Infrequent user)* |

For peer review only

## Appendix table 7 Participants' expectations of the potential communication and monitoring benefits of the TiM service.

| **Potential benefits of the TiM identified by participants** | |
|---|---|
| **Improved communication with MND team** | |
| Increase speed of communication | *"If I put it on the tablet and I send it to you, you get it there and then. So if anything … happens to me in that period of time you know straightaway … So the quicker you can pick up on so, something it's better for you as a doctor as well as me as a patient." P091* |
| Increase frequency of communication | *"And you know somebody within the team is going to look at that information. So you are connected, once a week. So it's not every three months." C184* |
| Provides a direct connection with specialists | *"Well I'd read up about the telemed and I thought, well it gives you a direct connection with Sheffield and the team. I know it's an IT link but it's a definite link because once a week you are communicating with a team." C184* |
| Improve communication and liaison with other members of the MDT | *"Well I can't see there's a different way to what we're doing now; I'm quite surprised that you're all in communication really….I didn't know you spoke to [community MND team], and I didn't that [GP] and you and the hospital were quite so connected, so that seemed to be quite a positive thing." P354* |
| **Increased monitoring** | |
| To identify problems quickly | *"It just seemed a way of being able to communicate with, with my care team and letting them know on a regular basis how you're doing; and no doubt at the end of it (…) it flags up that I need help or I don't need help." P134* |
| To identify problems between clinic appointments | *"I thought it'd be a good idea for both of us, but mainly for P and, because it is a long time between the hospital appointments, and I thought well it's a good idea to monitor symptoms if they crop up out of the blue and we're not sure whether it's anything..." C062* |
| Enables a better understanding of the disease | *"When, when P started off with the home journey we, it was obviously dizzy spells…. So I said well we'll just keep a diary of what's happening so you yourself know what's happening, so that's what he's doing, and in effect, this is what, your telehealth could do eventually is sort of monitor people…" C091* |
| Enable self monitoring | *"So we can monitor ourselves as well at the same time. So it's helping you in the long run as well…I think you've gotta monitor yourself so you know how, what you can do and what you can't do. It's, you can't just rely on everybody on the end of a phone, pick up and say, you know, I've got this, that and the other. You've gotta look at health and safety for yourself as well, for whether you can walk or whether you can run or whether you can open a door or open a lid or carry a glass or whatever, you know, at the end of the day that's monitoring yourself, so you've gotta do it." P091* |



For peer review only - http://bmjopen.bmj.com/site/about/guidelines.xhtml

## Appendix table 8 Participants' expectations of the TiM service.

| | |
|---|---|
| **Potential benefits of the TiM identified by participants** | |
| **Psychological benefits** | |
| Clinicians take an interest | *"Somebody's taking an interest, somebody's wanting to know" C184* |
| Reducing isolation | *"You're not going to feel isolated." C184* |
| Reassurance | *"Sometimes it's just the fact that, "should somebody know about this?" or "should you be telling somebody that." Wouldn't it be nice if somebody knew this. Just small details that you sometimes think, "Does it make a difference if somebody knew about it?" And that telemed makes that difference." C184* |
| **Improve accessibility of the MND service** | |
| Reduce clinic appointments | *"If you can glean enough information of what I send through so I don't have to come to the hospital, I think that's a positive" P354* |
| Provide access to MDT when unable to travel to clinic | *" We just thought it would be quite good cos not everybody can get into clinic and thinking about the later stages, but it's still important to monitor how things are going" P480* |
| Reduce travel time | *"that would then cut down my travel time to clinic, you know, clinic doesn't really fulfil a great deal in my life other than ticking some boxes and sort of knowing where the progression's going but, you know, I think most of that now can be done through this server, through TiM." P134* |


For peer review only - http://bmjopen.bmj.com/site/about/guidelines.xhtml

# Appendix table 9 The acceptability of the TiM questions.

| Acceptability of the TiM questions | |
|---|---|
| Patients wanted the TiM to include questions about all aspects of MND | *"I've found the questions, I think they're all relevant. And, … it might be in the future when things do, if things do get worse it might be I might need those questions to highlight things and that's quite, quite comforting." P423*<br>*"There's one question about you use a feeding PEG. I don't at the minute so that's not relevant to me at the moment. But obviously as time goes by it, it will be. I think I just put, "no", I don't use one... But I think it should be on there because, … in time I probably will be using one." P145* |
| Emotional wellbeing questions were welcomed | *"I think it's important to find out, not just physically, how a disease affects somebody but how, emotionally, mentally it affects them. As a say, I'm quite a positive person, but, I have some off days." P122* |
| Carers happy sharing their sensitive information | *"Q: The sort of questions that they're asking you, quite, they're quite personal questions, aren't they...? How you feel about sharing those?*<br>*C: No problem." C392* |
| Questions made patients think about the consequences of their disease, but this was not a problem | *"Q: How does it feel having to sort of be faced with that question [about gastrostomy tubes]?*<br>*P: … when I first did that it was dead easy cos I just put "no" … and just moved on. But since then, since we've been to this Advance Care [planning] thing. …And that, that sort of brought it to the fore …. it's a thought that I wouldn't really wanna think about, but you've gotta think about, so I think on that one, is that something I shall maybe ask more to get more advice on." P248* |
| Reporting a deterioration on the TiM was not distressing | *"I think: am I getting worse? But, yes, am I getting worse…. I don't think any of the questions are really frightening. You know it might happen." P172*<br>*"in a sense it's good that you can change, you know, the, keep every, everybody up-to-date. Cos you don't know when things are going to change anyway, do we, we don't, you know, it's just, it's like a bit of a, a grey area isn't it? … as long as people who need to know see that information then it's … not waiting till your next visit at the clinic is it...?" P122* |
| Wanting questions that assessed how they were coping | *"Maybe if they say, "No, I've not been out for a fortnight", that it makes them think: well, actually why haven't I? And it would highlight that person's not getting out of the house and not doing something themselves and they are continually there. Do they need a bit more support?" C122* |
| Questions were repetitive | *" It's repetitive. … I pick it up and I think oh God, here we go again, same old, same old. [I] put a couple …more different ones in,… but no, the questions are right..." C145* |
| Questions become irrelevant if disability severe | *"As I am unable to use my arms and legs at all, many of the questions have become irrelevant to me beyond the first time of using it."* P047 |



For peer review only - http://bmjopen.bmj.com/site/about/guidelines.xhtml

## Appendix table 10 The validity / accuracy of the TiM questions.

| The validity/accuracy of the TiM questions | |
|---|---|
| Patients want answers to be accurate | *"If I've got a form I like to fill it in as accurately as possible." P248* |
| Patients were concerned that their answers would be misleading | *"I have lost weight [a few weeks ago] but I haven't [lost weight in the last week]. The way I'm reading it now… sounds like I'm still losing weight, well I'm not…I wouldn't like 'em to think looking at it, or you looking at it in Sheffield and thinking "God, he's still losing weight" P248* |
| The answers are insufficient to reflect small changes | *"Sometimes they answers to chose are too far apart so if the answer is one day a week, I answer no, or several, it's nearer several. So it's accurate but it's wide." P172*<br>*"The questions are quite in a narrow band, and because it's a slow-burner not much changes unless there's a step change, such as …the chest infection the other week." P354* |
| The condition varied from day to day making weekly assessments difficult | *"I thought: if I was doing the questionnaire on that day, "what sort of assistance do you get from your family? The answer would be off the scale somewhere. I thought: some of the questions don't quite fit the answer. So I try to put the most representative" C172*<br>*"I think some of the questions are a bit too general and wide rather than, for instance… what can I not do with my hand that I used to do, and how am I dealing with it." P166* |
| Informal care requirement questions were difficult to answer | *"The margins are too wide. One of the questions is, how much time do you spend in the day looking after her? And, there's a sort of, there's 3-4 hours. But I don't even look after myself for 3-4 hours, I just potter around. That's always gonna be the same answer." C172* |
| Some questions contradicted each other | *There are some questions on there that are bit ambiguous for me [for example] being able to use the stairs. Well I can't use the stairs but I still have a bathroom upstairs so I still walk from the top of the stairs to our bedroom." P134* |
| Patients wanted to provide more information to clarify their answers | *"… if you have any more questions to ask you've got that… availability to ask [the MND team] if you have any problems…" P056*<br>*"If you want to ask anything maybe you could type it in instead of it just being yes/no" CaT Patient 317*<br>*"I think that, that would be a good addition. Because you, you could answer a question and at the end of the section just put in, and just expand on what, why you've answered that, if you see what I mean? I think a comments box would be a good idea." P122* |
| Same problems identified every week | *"It's a probably the same answer I give every week… It says; "Do you stumble or feel that you fall, or have you fallen?" And I do, every single week, I guarantee that" P122* |
| Answer affected by other health problems | *"So at the minute I'm slightly doctoring the answer; so I'm saying I can dress myself but I can't, but it's not the MND." P381* |

For peer review only

## Appendix table 11 Participants understanding of the TiM service.

| | |
|---|---|
| **Aims of the TiM service:** | |
| Monitoring | *"For you to be aware of it, and monitor it..." P402* |
| Relay information to the MND team | *"It shows how you're feeling which gets then relays to the nursing staff. If you've any questions you've got the item on it where you can ... ask that as well... I would think... it's read, isn't it, and then probably recorded somewhere." P056* |
| Look for trends | *"I would say they are probably going on to some sort of graph that detects a trend. I mean with me, you wont get a trend because sometimes you are up and sometimes I'm down. It'll look like a [gestured up and down]. But I would imagine the ideal situation is when you can detect a trend but that does depend on what's happening." C172* |
| Provides trends and alerts to problems | *"I'm guessing that it comes to someone's desk and that they're able to see a chart... from week to week, and I would imagine that there would be a series of red flags for you or some traffic light system... that red's intervention required, amber's a warning, green's get on with it.." P134* |
| Prioritise patients in need | *"I could see the reason why you were doing it; I realised that all the data was going to be collated and you can see then at a glance... you can see the statistics and everything, and it would highlight to you... if I had a dramatic change." P317* |
| Data would be used for research | *"I didn't realise that that's what was going to happen, I thought it was just being used for research." C392*<br>*"I would assume it goes to some databank somewhere and they try and correlate the answers I've given with, perhaps somebody else ... to see if there is any common ground."P122*<br>*"I imagine it goes onto a database ... and then if yourself and [Professor] are ever doing any research they may be able to...or if there's any trials you might be able to select from that who would be suitable for that trial." P 317* |
| Both clinical care and research | *"I imagine it goes on a survey of all the people you're doing to compare us with each other, and also to follow my own progress." P166* |
| **The role of the Telehealth Nurse** | |
| TiM Nurse looked at data | *"It shows how you're feeling which gets then relays to the nursing staff, doesn't it." P056* |
| EH was looking at data | *"I didn't realise that [Telehealth Nurse] was involved and she would ring us if our answers drastically changed, cos obviously they've stayed very much the same. Then I'm quite encouraged by that, I just presume that you do it yourself...and if you'd got a problem you'd ring [Telehealth Nurse] up for a bit of help." C392* |
| Unsure who looked at data | *"I'm guessing that it comes to someone's desk and that they're able to see a chart" P134*<br>*"The ones that have spoke about it to me I don't think they realise that I'm seeing their responses." Telehealth Nurse* |


For peer review only - http://bmjopen.bmj.com/site/about/guidelines.xhtml

## Appendix table 12 Nurses' experience of using the TiM clinical portal. All quotes are from the Telehealth Nurse.

| **Using the clinical portal** | |
|---|---|
| Easy to use | *"The training that I've had from seeing it this side was brief from Esther and it's, it's never, it's not difficult so it, I find I, right from the beginning I found logging on has been the most difficult thing, so; but once you're on finding your way around it is very, very easy, very easy,"* |
| Low burden | *"It only takes minutes"* |
| Easy to understand the flag system | *"If everything's OK it's green, … [if] there's maybe some elements that might need to be looked at, it's an orange or yellow, and then if there's an alert it's a red one."* |
| IT support for passwords required | *"My main problem was me accessing it to begin with, and that was very problematic. It's just asked me to change me password."* |
| Nurse didn't know how to use the patient TiM app or resolve technical problems | *" N: I have had a couple of phone calls, cos [the participants have] got my contact number through this, and they've rung with a problem, usually a logging on problem and…*<br>*Q: Forgotten their password?*<br>*N: Yeah…I've asked Esther, to be honest, because I'm not familiar with their device."* |



For peer review only - http://bmjopen.bmj.com/site/about/guidelines.xhtml

## Appendix table 13 The accuracy and sensitive of the TiM information. Quotes are from the Telehealth Nurse (N) unless otherwise stated.

| **Accuracy of the TiM information** | |
|---|---|
| Information accurate to help make some decisions | *"N: [EH] has led me to believe that it's quite easy to red flag on the carer. So this chap is triggering ... [reads the PHQ4 questions] So I mean ... the four that are triggering, they're all about...<br>Q: Worry, stress?<br>N: Yeah, thoughts: yeah. I know how poorly this lady is, I know how disabled she is, and I do know him, and when you speak to him he's very blasé about it all, which actually is more of a worry."* |
| Information in TiM wasn't sufficiently detailed enough to be sensitive | *"N: ...I think if they're routinely sending us an update every week I think you're more likely to pick up on problems, but I don't... I thought that would be so.<br>Q: Is that, do you think that's happened?<br>N: No not really. I don't know that the questions are sensitive enough, and I suppose the thing is that it might, if it triggers a contact phone call then you may well pick up on things sooner." (early interview)* |
| Needed more information | *"N: It's almost like you need a two-way thing (laughs) you need to ask them a question, [for example] how long have you been coughing... are you bringing anything up when you're coughing... you know, that type of thing... have you had a temperature... it's almost like [laughs] you need a two-way communication, cos this is just a snapshot, isn't it..."* |
| Discussions are required to fully understand a problem | *"I think some of the problems with patients using NIV, very specific problems, you only pick up from a conversation with them. I don't think you pick it up on the Tele, cos it's [the way] it's structured, it might show a problem but not a specific problem."* |
| An additional comment box would help gain more detailed information | *"Q: Yeah. Do you have, would you have time to read the comment boxes?<br>TW: I would hope so. You'd have to make time. If it was a problem that was coming up all the time, yes, I would hope that that; in some ways that might save you time."* |



For peer review only - http://bmjopen.bmj.com/site/about/guidelines.xhtml

# Appendix table 14 Participants' experiences of how the TiM impacted on their care.

| Impact on if TiM on patient care | |
|---|---|
| Improves knowledge | *"Because since I've done it I'm taking more notice of my weight but I didn't before so I know it very carefully. It made me aware of that." P172* |
| Close monitoring | *"I'm certainly not left alone for more than a month, ever...Sheffield is there if I really need them, but on the other hand [local team] is here." P166* |
| Reassurance to know being monitored | *"I think the benefit to P is real. Because... somebody is there on hand looking at things... Because it's slow with P and he doesn't need as much attention and care, it's easy to feel detached from any positive interaction." C122* |
| Keeping in touch with specialist | *"...it's knowing that somebody else is in your corner." C423*<br>*Q: Do you think there might have been a point in your disease where those questions ...were useful?*<br>*P: Nearer the beginning, definitely. I think, and I can only guess, that it must be the same for lots of long-term conditions; you know, I've lived with this for over five years now... you get to understand your own body and you know it's, it's normal for me now and for the first year no day was normal, no day looked like any other day in my life before that. So... if I could have camped in [MND consultant]'s house for the first six months I would have done, just so she was there, so I could say, but what about this, what about that; and you imagine symptoms, or I did, you know, you think God, this is happening and that must be related to the MND... So in the first year I would have filled that in every day, just to have that touch point," P047* |
| Nurse giving advice | *"When I came back on Tuesday last week and I did the second questionnaire, and within a day [Telehealth Nurse] saying "I've got a red flag on one of your answers." And it's the fact that I'd fallen twice while I was away on holiday and I'd put on it, you know: it said "have you fallen recently, how many times?" and I'd fallen twice while I was on holiday. So she phoned me, and said, "Are you ok? Is there a reason why you fell?" No, just my usual clumsiness....*<br>*Q: Were you expecting her to call?*<br>*P: No, I wasn't actually. It was just a bolt out of the view...I find that quite positive. It shows that the whole idea of it works.*<br>*Q: Has it changed your behaviour at all?*<br>*P: No. Not really." P122* |
| Nurse identifying problems | *"Q: [Telehealth Nurse] called, I think she spoke to you about when you fell...*<br>*P: Mm.*<br>*Q: ...what did you think about that when that happened?*<br>*P: It was useful wasn't it?*<br>*C: Well you weren't in so she spoke to me.*<br>*P: Yes, because I'd tripped over the bedroom chair...*<br>*C: that's right and, yes, that was the main thing, that's right, yeah. No, it was interesting that that, that had been picked up because we weren't, we don't know how it was picked up." P&C166* |
| Identifying problems between clinic visits | *"When I filled it in last week, and within a day [Telehealth Nurse] was phoning me. How much better could you have that? Instead of, two months down the line and I attend the clinic and they say "how have you been, have you fallen?" and I say "oh yeah I did: two months ago". See where I'm coming from? You've got that instantaneous contact with this technology that perhaps you don't have without it. So I'm a great believer in that, I'm a great believer in technology." P122* |
| Supporting important decision making | *"Q: The other question I had was that we kept an eye on your weight and I wonder whether you think that may have influenced your decision to have a feeding tube or not; do you think it, do you think it had?*<br>*P: [writing] I was frightened by the speed of loss of weight but was convinced how much muscle I lost." P409* |
| Help accept the disease | *" The questions nudged me to facing what I could do and not what I can't." P409* |



For peer review only - http://bmjopen.bmj.com/site/about/guidelines.xhtml

# Appendix table 15 The Telehealth Nurse's attitudes towards the value of the TiM.

Quotes are all from the Telehealth Nurse

| **The Telehealth Nurses' attitudes towards the value of the TiM** | |
|---|---|
| TiM could be valuable | *"As a tool to aid the patient and then aid the nurse"* |
| To identify problems between clinic visits | *"A number of patients with MND and their carers will wait till a clinic, instead of contacting us with, if they're, they're worried about something, or there's a change in their, you know, their condition, and I think if they're routinely sending us an update every week I think you're more likely to pick up on problems."* |
| Alerting to problems earlier | *"Q: So it could have a use maybe in alerting you to earlier need for intervention?*<br>*N: Yeah, yeah."* |
| Help monitoring trends | *"I mean the weights have been quite interesting, cos if they can use the weighing scales … that's been quite interesting, so we can monitor their weight…. cos otherwise you wouldn't necessarily see that variation."* |
| TiM wouldn't have a negative impact on the service | *"Q: do you think a patient will be concerned this is trying to take away a part of the service, or do you think the clinical team might feel it's taking away part of the service?*<br>*N: I don't think either side would. From a clinical side I think that anybody would be willing to make it as easy for the patient as possible."* |
| Clinics are a burden to patient | *"I think from the patient's point of view I think it becomes very burdensome, the travel into clinic, very much so…But certainly the, the travel and the amount of time and effort for them to come to clinic to sit in clinic to then go home again, it's very difficult for them."* |
| Patients do not see value in attending clinic | *"… [patients] sometimes say;* "nothing, I don't get anything out of coming to clinic because you're reiterating the same things, I know I'm getting worse…" *So there are some patients that don't see the value of coming to clinic anyway. Now whether they would use a system like this and see the value of that I don't know."* |
| TiM could allow patients to be managed remotely | *" I think it could. I think it's one of those difficult things that at the moment, because it's not something we systematically do. And they are attending clinic or I have connections with their community care team, so I am in touch with what's happening and things are getting monitored. I don't know."* |
| Some problems need face to face assessment | *"Q: How reliable this kind of technology would be in deciding when or, when to cancel an appointment for a patient?*<br>*A: Oh I don't know, I don't know.*<br>*Q: Would you feel secure looking at that saying that patient doesn't need to come in?*<br>*A: I think, I think it depends, so when I look at, if it's a breathing problem and if I didn't know them and they were having problems with their breathing …that would alert me and I would [think]: right we need… to contact, we need to be looking at this and monitoring it…I think it depends."* |


For peer review only - http://bmjopen.bmj.com/site/about/guidelines.xhtml

## Appendix table 16 Participants experiences of clinician feedback.

| To act upon problems and provide feedback | |
|---|---|
| Feedback reinforces the benefit of the TiM | *"I find that quite positive. It shows that the whole idea of it works." P122* |
| Not receiving feedback is demoralising | *"The emotional psychological depths that I've been to, I was putting the stuff in Telehealth and thinking but nobody's acknowledged this or contacted me about it, and I thought: well they're not going to because that isn't what the clinic's about, and that made me stop using it." P047*<br>*"...it's like all forms and all surveys... they say "your opinion is important please fill in the following..." and you say something that you think is absolutely dramatic and mind blowing and nobody comes back to you on it. And you think: well how important is that survey?" C172* |
| To provide feedback even if nothing had changed | *"Q: And would you expect perhaps someone to tell you what's going on with the tablet or the answers?*<br>*P: Yes. Because if I hadn't changed much, I would have thought I would have had some feedback."P172* |
| To acknowledge problems even if nothing can be done | *"[falls] ... knocks your confidence ...I probably were putting too much onus on Sheffield Hallam because (laughs) we've got this and there's not jack shit they can really do about this and we know that...*<br>*Q: But that kind of acknowledgement's quite important, do you think, of the, what happens ...?*<br>*C: I do, yeah, it's a bit of support, in't it, it's knowing that somebody else is in your corner." C423* |
| MND team couldn't solve their problem | *"Q: If your answers changed what do you expect of the MND service?*<br>*P: I don't, to be honest. Let's say if I thought I'd got a problem I wouldn't necessarily come to you because I don't think, I think, you know, I, the impression that it's medical to do with P, and if I have got a problem really; because when you flash up it does say if you, is to contact the, there is, so I would probably, I mean that's not part of your remit, is it, me really if I've got a problem?" C381*<br>*"[emotional support] That's not what that clinic's about, that clinic's about physical wellbeing and physical health... truthfully I thought I don't know why these questions are in here, because that clinic isn't equipped to deal with that." P047* |
| Feedback thought to be pointless if nothing can be done | *"But then again, what's the point of coming back if you can't say anything?" C172* |



For peer review only - http://bmjopen.bmj.com/site/about/guidelines.xhtml

## Appendix table 17 The mismatch between the participants' and nurses' expectations of the TiM service.

| Mismatch between patient and nurse expectations | |
|---|---|
| Patients expected contact if they experienced important problems: | |
| Falls<br>Chest infections<br>Dysphagia | *"Q: ...do you, do you think they're important things that she needs to know about?<br>P: I would say yeah… cos one of the questions is have you fallen or tripped? Obviously if I am falling more often, (well I am, I fell a few weeks ago, didn't I? but that, that's first time in months...). If I was falling … over every week I would say that, yeah, she needs to know something like that, or if I'm starting to not eat me food properly, if I'm starting to cough, I think obviously she needs to know that" P145* |
| Emotional difficulties | *"Most of my problems at the time are emotional, and the TiM does not appear to trigger any intervention." P047* |
| Problems may not be seen as important to nurse | *"She red flagged that she'd fallen, which is quite a common occurrence on a lot of patients, and I don't particularly worry unless they've been very, very well and then suddenly." Telehealth Nurse* |
| Problems may not be seen as important to patients | *"Q: When you put that you had fallen, what were you expecting to happen?<br>P : No. No.<br>Q: And when you heard that [your OT] had heard about it, what did you think about that?<br>P: Nothing really.<br>Q: And, do you think in the future, if something were to happen to you, and you put on here, what would you expect to happen, what would you like to happen?<br>P: I would think [Telehealth nurse] would ring. But the only thing is the over balancing, so far, isn't it." C217* |
| Problem may not be seen to be under the MND team's remit | *"[emotional support is] not what that clinic's about, that clinic's about physical wellbeing and physical health. To me, and in my own head, I thought it's never been about psychological wellbeing and your mental wellness and therefore; and truthfully I thought I don't know why these questions are in here, because that clinic isn't equipped to deal with that." P047* |
| Participants expecting contact when stable | *" It would be nice for someone to phone occasionally … particularly if anything changes.: C172* |
| Not expecting a particular action: expect nurse to decide | *"Q: And what would you expect to happen if you did change your answers like that week?<br>P: Not really, no, cos you know more than I do." P056<br>"I would tend to leave it to them to solve anything that needed solving." C217* |


For peer review only - http://bmjopen.bmj.com/site/about/guidelines.xhtml

## Appendix table 18 The problems associated with excessive TiM alerts. All quotes are from the Telehealth Nurse unless indicated.

| **Excessive TiM system alerts** | |
|---|---|
| The same alerts appear every week | *"We have some of our patients that do this every time… They … cough or choke at least once a week."* |
| Alerts increase the time required to use the system and can cause frustration | *"Then when it was coming up every week… I knew I'd spoken to them about that [problem] and that was their choice. And I'd kept putting [a comment in the TiM notes] but you're sorta thinking; why do I have to keep putting it on every time?"* |
| Nurse can't control the alerts | *"If you know somebody's got a problem and they're not really trying to do anything about it, then you know that [alert is] gonna keep coming back every week; and I haven't been putting comments on all the time."* |
| Nurses may appear to not be acting on potentially dangerous problems | [1]*"[reads]* "Do you ever cough or choke on food?"*And then he's put:* "Occasionally", *and that's fine."* |
| Problems flagged even though the patient has chosen not to medical advice to avoid the problem | *"cos I know that patient and I know that they've chosen to eat and that it is problematic. But they've got a feeding tube and they should really be using their feeding tube, but they're [also eating]. Then when it was coming up every week, I knew I'd spoken to them about that and that was that choice?"* |
| Reporting the same problems without solutions could be demoralising to patients | *"Maybe to them the fact that it's the same thing week in week out, they've got an insight into that problem, it's not changing and, so they're not looking for something to help with it really."* |
| Repeated problems on which no action could be taken should be paused to avoid excessive alerts | *"Q: Are there any other, other things that you'd change at the moment to make it, to improve the system?*<br>*TW: Apart from the, the same red flags coming up every week with the same problem, having some way of either taking them off or say, or putting a comment in that, that would take it off by saying you've addressed this problem, it's, it's still gonna be there, it's not gonna change."* |



For peer review only - http://bmjopen.bmj.com/site/about/guidelines.xhtml

## Appendix table 19 The problems associated with excessive TiM alerts.

All quotes are from the Telehealth Nurse.

| **Nurse reaction to alerts** | |
|---|---|
| Calls patient for more information | *"[I] ring the patient about it and ask them"* |
| Liaises with the community team to get more information | *"I try and get a bit more information, cos the, the community team may have been out and seen them and seen them face-to-face and have got something a little bit more useful back; so I have used it for that circumstance. And I'm in touch with the community team quite often, so sometimes if I'm ringing about something else I'll ask about one of the patients that's on here."* |
| Prefers to wait until clinic | *"I've seen somebody that was red flagged, cos they're coughing more..I've put a [TiM note] that we'll review in clinic next week."* |
| Will chose not respond to problems she thought were common in MND | *"She red flagged that she'd fallen, which is quite a common occurrence on a lot of patients. I don't particularly worry unless they've been very, very well and then suddenly. So if it happens over a few weeks and I've spoken to them and I know the situation…I'm not always alerted by that red flag."* |
| Using all the TiM information on the to make a decision | *"They were having problems with their breathing but I would look: are they on a breathing machine? No. That would alert me. So I think it depends."* |
| Patients and nurses prioritise different problems | *"I did the second questionnaire, and within a day the [Telehealth Nurse] calls saying "I've got a red flag on one of your answers." And it's the fact that I'd fallen twice while I was away on holiday… she phoned me, and said, "Are you ok? Is there a reason why you fell?" No, just my usual clumsiness….*<br>*Q: Were you expecting her to call?*<br>*P: No, I wasn't...It was just a bolt out of the view… I find that quite positive. It shows that the whole idea of it works." P122* |
| Having a relationship with the patient made it easier for the nurse to call the patient | *"I think it helps me that I know the patients. So I know this chap very, very well, I have a relationship with him and his wife; ringing up's quite easy to do, they wouldn't, they wouldn't be fazed by that. My response would be;* "oh there's been a red flag on the Telemedicine that you sent through, a message that you've sent through, and that's why I'm ringing"*. And that was quite an easy one."* |
| Harder to ring those who she didn't know | *"There are a small number of patients that I don't know, it just so happens that they've been very well through, so there hasn't really been any major alerts."* |
| Ringing carers may be difficult | *"If we've had a carer's response that's red flagging I don't always find that an easy phone call to make to a carer who's struggling."* |
| Patients not expecting her call | *"I rang a lady who I didn't know and she red flagged that she'd fallen… It was the lady's husband … he was quite shocked that I'd rung cos I didn't know them and I just explained about it, and he just said "No, it was just a little trip, she's absolutely fine, no problem."* |
| Nurse doesn't feel her calls benefit patient | *"I've said "Oh you've had some problem with this?...I know because you sent a Telemedicine and I'm the one that looks at the problems and sees what's what." And they said "Oh right, oh yes, it's nothing, it's fine." I don't know…"* |


For peer review only - http://bmjopen.bmj.com/site/about/guidelines.xhtml

# Appendix table 20 The future of the TiM.

| Preferred way to communicate with MND team | |
|---|---|
| Modes of communication depends on the individual | *Q: How do you think is the best way for people contact you?<br>P: I think for me it'd be by phone, but again that will be depending on the carer and my speech. I mean I'm currently OK with the phone.<br>Q: And how do you feel about email contact or contact through the Telehealth?<br>P: Again that's fine by me, I use email, computer all the time, but again you'd have to judge that on the individual." P381* |
| Contact needs to be convenient | *"And phoning you up, I don't know about you but I hate phone calls: you are just settling down to have a nice cup of tea or a chocolate biscuit or whatever and the phone rings, you have someone trying to persuade you to change your heating systems" C172* |
| Alternative methods of communication add flexibility | *"I'm really happy ...communicating by email, because for me I can do it then in my own time, because I haven't got use of my hands and arms; so if I know that there's an email that I can read and then reply to in my own time, that's far more relaxed for me actually than the telephone... I can speak fine but the telephone, somebody has to hold it for me...if I'm not in exactly the right position it's not comfortable to take a r." P047* |
| Telephone may be uncomfortable for some | *"I feel a bit awkward on the phone, and you can't get across how you're really feeling on a phone anyway..I tend to get nervous when I'm on the phone and stuff and I forget what I'm saying to people." P423* |
| Dysarthria makes telephone hard | *C: [to P] Well telephone calls aren't that practical are they for you. Because if you are tired, I mean it's difficult to convey."C172* |
| Email may be impersonal | *"I think that's a bit impersonal email, you know, that, that's what I thought" C423* |
| Telephone useful for problems needing an immediate answer | *"If it's something that needs doing, dealing with here and now: the telephone, email is, is my favourite way of communicating, because it suits my condition.." P047* |
| Some subjects are better discussed face to face | *"I think it's not personal... I'd rather see somebody or talk to somebody than, than read about it on something on a screen." C366* |
| Happy to receive feedback in clinic | *" I realise that you are probably busy people, so I'm quite happy for someone to say, when we're down there, "oh by the way, your survey has altered and, you know, do you want to talk to you about it." C172* |
| Happy to wait for a reply as long as their message had been acknowledged | *Q: And if you put in a question... How long would you expect before you got an answer?<br>P: Well normally [Telehealth Nurse] gets back to me within a day or so. So I would expect two days, almost maximum.<br>Q: OK. So, reasonably quickly.<br>P: Even if, it was: "we've got your question :we're thinking about it"<br>Q: So, just an acknowledgement that you've email?<br>P: Yeah. It may be [Telehealth Nurse] can't answer me first time. Like the citalopram. What she said was: "I'll discuss it with, (I think) you" and then came back to me." P381* |
| Importance of respiratory monitoring | *"Q: What do you think, how do you think this would fit in around your clinic visits and the support you would get from [Telehealth Nurse]? If this was to become...rather than a research project, part of standard care, how do you think you would suggest using it?<br>P: Well I think in my case I would be happy to use that and lengthen the time between visits. I mean, the only difference to me is the breathing test." P381* |
| Happy to share the information with other members of the care team | *"Q: And is there, is there someone locally that you think would be, so that would be able to see your answers ...?<br>P: Well a good one would be the GP, wouldn't they? Or even the hospice, or, even the district nurses, probably more the district nurses, cos obviously they've got a regular visit now, haven't they?" P056* |



For peer review only - http://bmjopen.bmj.com/site/about/guidelines.xhtml

## Appendix table 21 The Telehealth Nurse's attitudes towards how the TiM might be used in the future.

Quotes are all from the Telehealth Nurse

| **How would the TiM be used?** | |
|---|---|
| Delegating TiM duties may not be possible | *"I agreed to do it originally cos I said the thing is when a red flag comes if it's somebody else doing it and looking at it they're gonna come to me."* |
| The nurse wants to use her judgement, not follow a strict protocol | *"The way this study is run at the moment I can respond in, in the way I think is appropriate" Telehealth Nurse*<br>*"if it was written down, [that] I had to ring and I had to ring straightaway, ... I don't know whether, I would have found that quite difficult not being able to use my initiative and how I'm familiar with the patients and, don't know I might not have, I might have found that a bit more difficult."* |
| Some nurse may be more willing to follow a protocol and call more often | *"N: It's difficult, I think you would probably get more useful information out of the research nurses, but you would have to have a system where they'd be able to go to somebody to act upon what was [needed]" Telehealth Nurse*<br>*"I think if it was a bigger study and.. it was a very ... carefully monitored study, I think maybe the person doing this, looking at this ... looking at what the replies have been, if they had to contact them that might work better."* |

For peer review only

## Appendix table 22 How the TiM might be used by other teams

| | |
|---|---|
| **Use by community teams** | |
| Community teams would need capacity | *"I think if they have the capacity it would be useful," Telehealth Nurse* |
| It would increase information available for community teams | *"Q: Could [you] see [the TiM] fitting into the role you play or whether you think it's not very helpful?<br>N: I think it probably could because, like you've just said.. that [patient has] reported on here that she's … coughing/choking so many times but she never said that to me." Community nurse* |
| Community staff may not have access to computers | *"I'm not very good at computers. I have to say, that is the thing I find most stressful about working here. You I'm home based. I go on NHS website...so I'm not on, well I can get onto it but I do not cos I don't spend a lot of time [in the hospital]."Community nurse* |
| Community staff happy to receive information from Telehealth Nurse | *"Q: If someone else was looking at this, telling you there's a problem?<br>N: Yeah, no, that would be fine, that would be fine, yeah, yeah."Community nurse* |
| **Use by other MND centres** | |
| Other services would respond positively | *"I think their gut reaction is that that sounds something potentially helpful to us; I haven't really had any negativity" Telehealth Nurse* |
| Other services would want TiM if it saved them time | *"I think that if it saves them time I think that they would definitely embrace it." Telehealth Nurse* |
| Other services would want TiM if it benefited the patient | *"From a clinical side I think that anybody would be willing to make it as easy for the patient as possible, as long as it was useful." Telehealth Nurse* |
| Nurses would use it if it were part of their usual role | *"Q: But then if you were thinking about implementing this into the NHS who would you get the most useful information from?<br>TW: I think if it was implemented and it was part of somebody's role every day they would just do it, they would do it automatically; and, yes, I could envision that it would be very useful. So I suppose I'm a bit, it's half and half." Telehealth Nurse* |



For peer review only - http://bmjopen.bmj.com/site/about/guidelines.xhtml

BMJ Open

# Telehealth in motor neuron disease: a process evaluation and exploration of digitally-enabled access to specialist services.

| | |
|---|---|
| Journal: | *BMJ Open* |
| Manuscript ID | bmjopen-2018-028526.R1 |
| Article Type: | Original research |
| Date Submitted by the Author: | 23-May-2019 |
| Complete List of Authors: | Hobson, Esther; University of Sheffield, Sheffield Institute for Translational Neuroscience; Sheffield Teaching Hospitals NHS Trust, Department of neurology<br>Baird, Wendy; University of Sheffield, School of Health and Related Research<br>Bradburn, Mike; University of Sheffield, Clinical Trials Research Unit, School of Health and Related Research<br>Cooper, Cindy; University of Sheffield, Clinical Trials Research Unit, School of Health and Related Research<br>Mawson, Susan; University of Sheffield, School of Health and Related Research<br>Quinn, Ann; Sheffield Motor Neurone Disease Association Research Advisory Group, SITraN<br>Shaw, Pamela; University of Sheffield, Sheffield Institute for Translational Neuroscience; Sheffield Teaching Hospitals NHS Trust, Department of neurology<br>Walsh, Theresa; University of Sheffield, Sheffield Institute for Translational Neuroscience; Sheffield Teaching Hospitals NHS Trust, Department of neurology<br>McDermott, Christopher; University of Sheffield, Sheffield Institute for Translational Neuroscience; Sheffield Teaching Hospitals NHS Trust, Department of neurology |
| <b>Primary Subject Heading</b>: | Neurology |
| Secondary Subject Heading: | Health services research, Palliative care, Patient-centred medicine |
| Keywords: | Telemedicine < BIOTECHNOLOGY & BIOINFORMATICS, Motor neurone disease < NEUROLOGY, Organisation of health services < HEALTH SERVICES ADMINISTRATION & MANAGEMENT, Adult palliative care < PALLIATIVE CARE |
| | |

SCHOLARONE™ Manuscripts

# Telehealth in motor neuron disease: a process evaluation and exploration of digitally-enabled access to specialist services

*Dr Esther V Hobson[1,2], Professor Wendy O Baird[2], Mr M Bradburn[2,3], Professor Cindy L Cooper[2,3], Professor Susan Mawson[2, 4], Mrs Ann Quinn[5], Professor Pamela J Shaw[1], Sister Theresa Walsh[1], Professor Christopher J McDermott[1]

*corresponding author

1 Sheffield Institute for Translational Neuroscience, University of Sheffield (SITraN), Sheffield, UK, 2 School of Health and Related Research, University of Sheffield (ScHARR), Sheffield, UK, 3 Clinical Trials Research Unit, School of Health and Related Research, University of Sheffield, Sheffield, UK, 4 NIHR CLAHRC Yorkshire and Humber, UK, 5 Sheffield Motor Neuron Disease Research Advisory Group, Sheffield, UK.

**Author email addresses**
e.hobson@sheffield.ac.uk
w.o.baird@sheffield.ac.uk
m.bradburn@sheffield.ac.uk
c.l.cooper@sheffield.ac.uk
s.mawson@sheffield.ac.uk
Ann Quinn: c/o e.hobson@sheffield.ac.uk
pamela.shaw@sheffield.ac.uk
theresa.walsh@sheffield.ac.uk
c.j.mcdermott@sheffield.ac.uk

**Keywords**
Telehealth, telemedicine, motor neuron disease, amyotrophic lateral sclerosis, qualitative, process evaluation

**Word count**
6668

For peer review only

## ABSTRACT

### Objectives

To evaluate the processes involved in using a novel digitally enabled healthcare system (TiM: Telehealth in Motor neuron disease) in people living with motor neuron disease (MND) and their informal carers. We examined TiM implementation, potential mechanisms of impact and contextual factors that might influence TiM implementation or impact.

### Design

An 18-month, single-centre process evaluation within a randomised, pilot and feasibility study.

### Intervention

TiM telehealth plus usual care vs. usual care.

### Setting

A specialist UK MND care centre.

### Participants

Patients with MND and their primary informal carers

### Primary and secondary outcome measures

Patient, carer and staff outcomes and experiences using semi-structured interviews. Descriptive data on implementation and use of TiM telehealth.

### Results

The TiM was acceptable and accessible to all 40 patients, 37 carers and staff. Intervention uptake and adherence were good: 14 (70%) patients completed a TiM session at least fortnightly. Barriers to TiM use (such as technology experience and disability) were overcome with well-designed technology and face-to-face training. Reported potential benefits of TiM included improved communication and care coordination, reassurance, identification of complications and the potential for TiM to be an alternative or addition to clinic. Benefits depended on patients' current level of needs or disability.

The main challenges were the large number of alerts that were generated by TiM, how the clinicians responded to these alerts and the mismatch between patient/carer expectations and nurses actions. This could be improved by better communication systems and adjusting the alerts algorithm.

### Conclusions

TiM has the potential to facilitate access to specialist care but further iterative developments to the intervention and process evaluations of the TiM in different services are required.




For peer review only - http://bmjopen.bmj.com/site/about/guidelines.xhtml

## ARTICLE SUMMARY

### Strengths and limitations of the study

- This is the first evaluation of a digitally-enabled care system that aims to provide accessible, specialist holistic care to patients with motor neuron disease and their carers.
- The use of mixed-methods provided an in-depth understanding of the processes involved in the new care service, the potential value of TiM and factors which are likely to influence its use, implementation and success.
- This was a small, single centre study using only one nurse to deliver the intervention.

## BACKGROUND

Motor neuron disease (MND) causes progressive muscle paralysis, severe disability and eventually death within on average, two to four years (1). Attendance at a specialist multidisciplinary clinics (MDC) is associated with improved survival and use of assistive aids, devices and proven therapies (2-7). Patients face many barriers accessing specialist services meaning many patients fail to gain the full benefit from these services (8). There is some evidence that digital-enabled technologies can improve access to specialist care (9). Video-consultation (telemedicine) is used in some centres and appears to be feasible and acceptable (10-13). However, between consultations changes in patients' condition may not be recognised. There are several technologies in development that can frequently record aspects of MND progress (telehealth) such as speech or hand function. They have predominately been developed for research but telehealth could be used for clinical care purposes (14-17). No digital system has yet been evaluated that can monitor the full range of problems faced by patients with MND in order to detect and act upon issues that arise in a timely manner.

There also remains a need to develop interventions to support carers of those with MND. Medical interventions such as non-invasive ventilation and gastrostomy feeding require carers to conduct complex tasks with little training (18). Carers report that these interventions are associated with increased physical strain and carer time (19-21). Provision of disease specific information and signposting to external support services have been identified as helpful (22,23) and whilst one small study of intensive case management reported positive feedback (24), another showed no improvement in carer strain (25).

We employed a user-centred design process to develop the TiM telehealth system (Telehealth in MND) (26). This allows patients and carers to send information about their condition to the specialist care team (26). Early testing has already been conducted with a small number of potential users but further testing in the clinical environment was required (26). Telehealth services are complex interventions, consisting of different component parts and whose success relies on the context and individual behaviours of those using and delivering the intervention (27). Clinical trials of complex interventions such as telehealth face problems with patient and staff engagement and often lack of



For peer review only - http://bmjopen.bmj.com/site/about/guidelines.xhtml

understanding of how the intervention is used, what works, and for whom (28-30). People living with MND and their carers face additional barriers to using digitally-enabled care due to their disability and age. The complexity of the disease and the holistic aims of care in this terminal disease mean telehealth systems will need to differ from more simplistic, target-driven care systems (such as blood pressure management). With these challenges in mind, the Medical Research Council recommend that development of a telehealth service is iterative with learning from development, piloting, evaluation and implementation informing each step (31).

### Aim of the study

We conducted a pilot and feasibility randomised controlled trial (RCT) in patients and carers of usual care plus TiM, versus usual care. The results of the pilot and feasibility trial are described in parallel publication. In this paper we describe a parallel process evaluation (32) which aimed to gain a deeper understanding of the use of the TiM system.

We aimed to observe the processes that occurred when the TiM was used to deliver specialist multidisciplinary care in MND exploring:

- TiM technology set-up and delivery
- Participants' adherence to TiM
- Participants' attitudes towards TiM
- Clinicians' attitudes towards TiM
- Potential impacts of the TiM on participants and staff (both intended and unintended)
- The mechanisms and contextual factors that may affect the impact and implementation on a larger scale.

We also aimed to:

- Identify problems with TiM
- Begin to implement and evaluate improvements
- Identify further improvements and uses of the TiM.

## METHODS

### Study design

The methods of the pilot and feasibility RCT are described in a parallel publication. A supplementary file [Online supplementary file Methods TiM protocol and statistics analysis plan Process evaluation 2019] contains the TiM protocol, topic guides and statistics analysis plan. In brief, patients with MND (amyotrophic lateral sclerosis, progressive muscular atrophy and primary lateral sclerosis) and their primary informal carer currently receiving care from the Sheffield MDC were recruited and randomised to receive either the intervention (TiM plus usual care) or control (usual care alone). Usual care involved invitations to the MDC two to six monthly plus access between visits to the multidisciplinary team via a MND specialist nurse by telephone, email or through liaison with other healthcare professionals.



For peer review only - http://bmjopen.bmj.com/site/about/guidelines.xhtml

## Intervention

A detailed description of TiM has been published (26). The TiM Patient/Carer App contains a set of patient and carer questions assessing the disease progression, complications of MND, use of medical interventions and patient and carer wellbeing. The app was loaded onto a 7-inch Samsung Galaxy tablet with 3G internet. We asked participants to complete the questions weekly or more frequently if they desired. The results were sent using the internet to a clinical portal used by the MDC specialist nurse (the telehealth nurse) who had over 15 years experience of MND care. The portal assigned a red, amber or green flag to all the answers depending on a predetermined algorithm created by the TiM developers. The telehealth nurse was expected to review the answers and could liaise with specialists in the wider multidisciplinary team (MDT) and provide telephone advice. All patients and carers continued to receive usual care, Sheffield MDC appointments and telephone/email access to the telehealth nurse. Clinic appointments could not be postponed but could be expedited. The nurse was required to have a discussion with the patient, carer or clinician to confirm the accuracy of the information prior to making any clinical decisions. Also loaded onto the tablet were educational materials (known as the Knowledge Centre) and within the app was a section where patients could make a list of issues the participant wished to discuss at clinic (the Problem List) and educational messages, which appeared between questions.

EH recruited participants at home. Participants were given the tablet and wifi-enabled standing weighing scales. They were given face-to-face training, written information, telephone and email support. EH contacted patients after two weeks if they did not submit a TiM session and visited at one and six months for interviews which provided an opportunity to resolve technical issues. EH trained clinicians to use the clinical portal in face-to-face training, which took approximately ten minutes. During the trial we gained feedback from users in order to improve the system. The trial started in October 2014 and one major change to the patient app and the clinical portal was implemented in October 2015.

## Data collection

A detailed description of all data collection is described in the accompanying feasibility paper and in the protocol [Online supplementary file Methods TiM protocol and statistics analysis plan Process evaluation 2019]. Patient/carer reported outcome measures including quality of life and carer strain were collected at 0, 3, 6,12 and 18 months. To gain a deeper understanding of the processes involved we evaluated patient, carer and staff experiences of the TiM. We used satisfaction questionnaires, collected adverse and conducted semi-structured interviews. Interviews were conducted by EH at baseline (control participants), one and six months (intervention participants). Interviews with the telehealth nurse were conducted at 14 and 18 months. Additionally an interview with an MND specialist community nurse who cared for a number of participants in both treatment arms during the study was conducted at 18 months. Topic guides [Online supplementary file Methods TiM protocol and


For peer review only - http://bmjopen.bmj.com/site/about/guidelines.xhtml

statistics analysis plan Process evaluation 2019 page 41) included participants' experience of specialist MDC and community care, attitudes towards technology and digitally enabled care, expectations and experiences using TiM, reasons for good and poor TiM usage, potential impacts TiM could have on MND care, and improvements and future applications of TiM. MDC physicians completed a satisfaction questionnaire ("Shadow monitoring form", see [Online supplementary file Methods TiM protocol and statistics analysis plan Process evaluation 2019] page 71) when they saw a patient in clinic using TiM capturing opinions on the accuracy, feasibility and acceptability of TiM. Field notes were also taken to describe any problems with TiM, solutions that were used and their outcomes. At the end of the trial we downloaded all data collected by TiM into Excel. This contained the time and date of every session, all answers provided by patients and carers, all alerts generated by the system and all clinical notes made by the telehealth nurse. We attempted to record the telehealth nurse's time spent using TiM using diaries but these were incomplete. Instead, this was explored using interviews.

**Patient and public involvement**

During development of TiM (26) and the protocol we consulted patients, carers and the Sheffield MND Research Advisory group (a patient and public involvement group). They reviewed the intervention, principles of the trial, trial design, outcome measures and participant information leaflets and provided comments on their feasibility and accessibility. They were not involved in recruitment. Results of the study have been communicated at various public meetings, through the Sheffield MND Research Advisory group and local branch of the MND Association and a lay summary will be circulated. Members of this group attended the trial steering and trial management groups. AQ was a member of the trial management group, provided advice on the research methods, interpretation of the data and dissemination and is a co-author on this paper.

For peer review only

## RESULTS

### Participants

40 patients and 37 informal carers (three patients had no carer) were recruited. Two TiM patients withdrew due to severe illness and one died before six months, the rest used TiM for between six and 18 months. Participant characteristics are reported in full in the parallel paper. The patients broadly represented those attending an MDC including patients at all stages of disease (Table 1). Whilst patients' use of digital technologies was common, some participants had little or no experience using technology. 38 (95%) patients had home broadband and 33 (83%) had mobile internet reception.

For peer review only

Table 1 Participant characteristics

| | **Telehealth (n=20)** | **Control (n=20)** |
|---|---|---|
| Patient gender, male | 14 (70%) | 14 (70%) |
| Age (years) Mean (SD), range | 60.4 (11.7), 30-78 | 60.0 (10.0), 39-73 |
| Disease duration (months) Mean (SD), range | 53 (48), 12-197 | 46 (35), 7-123 |
| King's ALS clinical stage[a] | | |
| 1 | 3 (15%) | 2 (10%) |
| 2 | 4 (20%) | 5 (25%) |
| 3 | 5 (25%) | 8 (40%) |
| 4 | 8 (40%) | 5 (25%) |
| Use of the TIM App | | |
| Independently | 17 (85%) | 17 (85%) |
| Help from carer | 1 (5%) | 1 (5%) |
| Patient instructs carer | 2 (10%) | 2 (15%) |
| Patient technology use[b] | | |
| Daily | 14 (70%) | 18 (90%) |
| A few times per week | 3 (15%) | 1 (5%) |
| Once a week | 1 (5%) | 1 (5%) |
| Every few weeks | 0 (0%) | 0 (0%) |
| Never | 2 (10%) | 0 (0%) |
| | **Telehealth (n=19)** | **Control (n=18)** |
| Carer gender, male | 4 (21%) | 5 (28%) |
| Carer age (years) Mean (SD), range | 59 (12, 42-84) | 60.8 (11,38-73) |
| Relationship to patient | | |
| Partner | 18 (95%) | 16 (89%) |
| Child | 0 (0%) | 1 (6%) |
| Parent | 1 (5%) | 1 (6%) |
| Carer technology use[b] | | |
| Daily | 12 (67%) | 16 (84%) |
| A few times per week | 1 (6%) | 0 (0%) |
| Once a week | 1 (6%) | 2 (11%) |
| Every few weeks | 0 (0%) | 1 (5%) |
| Never | 4 (22%) | 0 (0%) |

[a]King's stage 1 refers to patients with functional deficit in 1 domain, stage 2 refers to disability in 2 domains, stage 3 refers to disability in 3 domains and stage 4 refers to patients requiring NIV and/or gastrostomy. King's stage was calculated using the ALS-FRS-R scale at baseline. [b]Technology use: computer, tablet, smart phone. SD: standard deviation



For peer review only - http://bmjopen.bmj.com/site/about/guidelines.xhtml

## TiM technology set-up and delivery

*[Online Supplementary data file Results Quotes and Figures Process evaluation 2019 tables 1-4]*

All participants allocated the intervention received the TiM system and could use it using mobile or home broadband. Technical problems and solutions were all resolved and had little impact on the trial except the wifi-enabled scales which were not found to be practical due to a lack of reliability connecting to the internet (Table 2). At six months all the patients and 14 (93%) of the carers felt TiM was easy to use (Figure 1). 14 (93%) patients and carers did not think the system was tiring and 13 (87%) did not think the questions were distressing, nor the use of the system intrusive.

*"It's so easy to do; it literally takes five minutes from home." Patient 317*

27 patients (68%) reported some difficulty using technology due to upper limb disability but 34 (85%) could use the patient app independently (although they might need a carer to help charge the tablet). One patient could use the app but during training it was noticed that his answers were not accurate (Patient 073). He was unable to communicate verbally and exhibited behaviours suggestive of mild cognitive deficits. In this case, he was happy for carers to help him use it.

Participants found face-to-face training important. Many were pleasantly surprised by their achievements.

*"I thought I wouldn't be able to do it (laughter) but I can…." Carer 228*

The main perceived barrier was participants' experience using technology.

*"…we're quite happy to deal with it, as long as it wasn't too techy" Carer 062*

Many participants (including those using technology daily) perceived themselves lacking an intrinsic ability to use technology, saying that they were "*bad*" at it, thinking others were more "*wired*" to using technology. They shared common attitudes towards their abilities, experience and approach to technology. They reported struggling to learn to use new technology finding it stressful and were fearful of making a mistake or breaking the device. They did not feel in control of technology, expressing frustration when technology did not "*obey*" them. This meant they were not confident adapting to unfamiliar technology. They tended to use technology for a limited number of basic tasks, lacking confidence to problem solve when technology *"went wrong"*, relying instead on others. Some low users explained they never learnt, or did not see the need to use technology, particularly if their partner used it.

One couple (Patient 166) had no experience of technology and were highly critical of the perceived intrusion of technology in their lives. They described themselves as *"technophobes"*. However, they trusted their care team would provide them with a secure and safe system. Only one carer in his 80s (Carer 217) struggled to *"catch on"*, needing help from his partner. He had little prior experience with technology and it was later noted that he had difficulties with



For peer review only - http://bmjopen.bmj.com/site/about/guidelines.xhtml

language due to another medical condition.

For peer review only



For peer review only - http://bmjopen.bmj.com/site/about/guidelines.xhtml

Table 2. Technology problems encountered during the trial

| Problem | Solution adopted | Impact on the use of the TiM | Recommendations for future TiM use |
|---|---|---|---|
| **TiM Patient App software** | | | |
| Poor finger dexterity | Handheld stylus provided. | 85% used TiM independently. | Provide stylus to all patients. |
| | Carer help to use device | Interview data suggested that help from carers was acceptable. | Encourage carer support. |
| Difficulties entering login password | Telephone support | Problem resolved with second TiM app release | Provide face-to-face training. Provide telephone technical support. |
| | Login page redesigned | No further problems reported. | Make local staff familiar with the software. |
| Lack of confidence using the app or other features on the TiM | Face to face training Partner/family helped | None: all participants could use the app. | Provide face-to-face training plus an additional contact after a few weeks to reinforce learning. Identify low confidence/experience users & provide extra training. |
| Patients not giving correct answers | Patient completing TiM with their family | Uncertain impact . | Capacity assessment at recruitment. Check TiM answers in training and in clinic. |
| **Tablet** | | | |
| Tablet stored in place not accessible to patient | No solution available. | Interviews suggested that adherence was reduced for two patients. | Use patients' own equipment where possible. |
| Tablet battery drained, unable to switch on | Telephone advice. | No impact. | Use patients' own equipment. Familiarise local staff with hardware. |
| Unexpected screens/ software updates | Telephone advice. | Reduced user confidence in tablet but no impact on use. | Use patients' own equipment. Use a basic tablet that only displays the TiM app. |
| User fear of "breaking" the tablet | Face to face training to improve user confidence | Patients/carers reluctant to use the additional features on the tablet | Use patients' own equipment. Face to face training. |
| **Internet connection** | | | |
| Poor 3G phone signal | Used patients' own broadband. | None: solution found for all patients (3G or patients' own broadband) | Use patients' own broadband. Check internet availability prior to TiM enrolment. |
| Patients switched wifi off | Home visit required. | Several TiM sessions failed to download. | Monitor adherence. Alert team if adherence is low. |
| Unreliable connection between scales and tablet/broadband | A separate 3G Wi-Fi route provided ("Mifi"). No solution if using broadband. | Additional home visits required and loss of weight data for several weeks. | Manual weight recording Avoid using peripheral devices. |
| **Clinician portal** | | | |
| Password/login problems | Support by external IT team. | Delayed access during MDC visit. | Local systems access support. |



For peer review only - http://bmjopen.bmj.com/site/about/guidelines.xhtml

**Participant adherence to TiM**.
*[Online Supplementary data file Results Quotes and Figures Process evaluation 2019 tables 4-6]*
Participants preferred to complete the TiM weekly (13, 87% of patients, 8, 53% of carers). They reported that weekly sessions helped them remember to use the TiM. Some suggested they would complete TiM less frequently if nothing had changed. The telehealth nurse suggested that fortnightly information was sufficient to enable her to assess a patient's progress, and even less frequently for carers. Adherence was calculated by dividing the total number of sessions completed by the number of weeks participants were in the trial (Figure 2 and Figure 3). 14 (70%) patients and 10 (55%) carers completed TiM sessions on average, at least fortnightly. 13 (70%) carers completed sessions, on average, at least every three weeks. Some participants who completed fewer sessions still continued to use TiM regularly. Adherence dropped during the study, but at 12 months 80% of patients were still using it weekly. The main reasons for low adherence were forgetting, holidays and deterioration in their illness. A lack of feedback about the answers participants gave was also a disincentive to use TiM, particularly when if they were expecting feedback.

*"It's like all surveys... they say "your opinion is important…" You say something absolutely and nobody comes back to you. And you think: well how important is that survey?" Carer 228*

The use of the additional features of the tablet (the educational messages, Knowledge Centre or Problem list) was not monitored during the trial. Unlike the main TiM app, participants reported that they were less likely to use these. Most were aware of these resources, particularly the short messages that appear whilst completing the TiM questions. Some participants reported reading some of the additional material but no one felt strongly that these had had a major positive impact with only half agreeing that they were useful (Figure 1).

**Participants' attitudes towards TiM**
*[Online Supplementary data file Results Quotes and Figures Process evaluation 2019 tables 7-11]*
14 (87%) patients and carers agreed that TiM questions were relevant to them and 12 (73%) patients and carers felt able to report all the problems they experienced using TiM (Figure 1). 13 (80%) patients and 12 (73%) carers would use it again. 14 (87%) patients and 12 (73%) carers would recommend it to another patient.

*"I think it's probably one of the best ideas to come out of the NHS for years." Patient 122*

Participants did not find using TiM intrusive or upsetting. They explained they were reminded about their disease everyday already and knew themselves if they had deteriorated without using the TiM.

*"It doesn't bother me. When I was first diagnosed I was not keen on talking about it. I've since got better and I think that helps. I'm quite happy to be reminded. I think you are reminded every day. That tablet makes no difference to that." Patient 381*


For peer review only - http://bmjopen.bmj.com/site/about/guidelines.xhtml

Some participants thought the questions were repetitive, particularly carers. Some noticed they reported the same problems every week. Some felt the questions were insufficiently sensitive to reflect the day-to-day fluctuation in their condition. One carer suggested that the questions should use other ways to assess carers lives more critically, which could be used to encourage carers to consider their own wellbeing and adopt coping strategies.

**Clinicians experiences using TiM**

*[Online Supplementary data file Results Quotes and Figures Process evaluation 2019 tables 12-13]*

The telehealth nurse said that using the clinical portal was "*very, very easy*". taking *"minutes"* per use. She explained that initially she looked at the system every day but by the end of the trial she looked weekly, and sometimes a little less often. She would look at each patient but focused on new and changing alerts. She explained that if she saw a patient/carer's answer was associated with a green flag it indicated everything was "*ok*", orange meant "*there's maybe some elements that might need to be looked at*" whilst a red flag indicated a problem.

*Safety and accuracy of TiM*

No adverse events related to TiM were reported and no participant reported delaying seeking medical attention as a result of TiM. 38 clinician satisfaction forms were completed when patients using TiM attended clinic. All agreed that TiM gave a useful and accurate picture of the patient and carer's condition and was a positive influence on the consultation. However, 20 (54%) were completed by the investigator (EH) who saw the patient in clinic, 18 (46%) were completed by other physicians.

**The reported potential impacts of TiM**

*[Online Supplementary data file Results Quotes and Figures Process evaluation 2019 tables 14,15]*

Reported potential impacts included improved monitoring and communication with the clinical team and improved effectiveness of MDC visits and care coordination. Other potential impacts included improved patient awareness of their disease and the ability to identify carer distress.

*Improving links with the clinical team*

Participants thought TiM could provide a "*direct link*" with the care team, increasing monitoring, providing better connection with specialists and enabling earlier identification of problems. Participants (particularly carers) also felt reassured that they were being thoroughly monitored. Reassurance was particularly beneficial for those who had infrequent contact with clinicians, either because they were early in the disease or were progressing slowly.

*"P… for the first year no day was normal… You imagine symptoms, … you think...that must be related to the MND...?*
*Q: Do you think there might have been a point in your disease where those questions …were useful?*



For peer review only - http://bmjopen.bmj.com/site/about/guidelines.xhtml

*P: Nearer the beginning, definitely…If I could have camped in [neurologist]'s house for the first six months I would have done, just so she was there, so I could say; "but what about this, what about that?"… in the first year I would have filled that in every day, just to have that touch point" Patient 047*

*Improved awareness of the disease*

Many participants thought that TiM improved their awareness of their condition. They felt that they did not need to track their progress using TiM as they knew themselves how they were changing, but some had become more aware of their weight and nutrition. Rapid progression was something all patients feared so those with slowly progressive disease noticed that their answers did not change rapidly. This made them feel more positive.

*Early identification of problems*

The telehealth nurse described TiM as "*a tool to aid the patient and then aid the nurse*". She could use TiM to gain more information and alert her earlier to problems. In one example, the TiM detected that one patient's condition was declining quickly and the carer reported high levels of strain. The couple was struggling to accept his decline and the patient tried to minimise his symptoms. TiM alerted the telehealth nurse to this patient's rapidly declining weight and bulbar function (Figure 4) She contacted him, discussed this information and encouraged him to consider gastrostomy insertion, which he accepted. He was asked about whether TiM affected this decision and explained that the information had been helpful:

*"The questions nudged me to facing what I could do and not what I can't...*
*I was frightened by the speed of loss of weight but was convinced how much muscle I lost. [sic]" Written quote Patient 409*

Other patients had reported problems on the app that they had not disclosed to their care team. The telehealth and community nurse speculated that this was because the patients were struggling to accept the change in their disease or need for treatment and might have felt that they can be more honest using technology. They found this information useful to gain a more complete picture of the situation.

*Improving the multidisciplinary clinic*

The telehealth nurse and physicians suggested that TiM could improve the MDC clinic. The telehealth nurse said if TiM could reduce the burden faced by patients attending clinic it would be welcomed by nurses. Potential benefits included facilitating sharing of information, collecting information and identifying problems before the visit. This could help plan the MDC visit to address the pertinent issues, shorten the time spent in hospital and make telephone appointments easier and a better alternative to face-to-face.

After seeing patients in their clinic using TiM, clinicians completed a feedback from (Shadow monitoring protocol). 23 forms were completed. On 19 (79%) occasions clinicians agreed that the information gathered by TiM could be used to make appropriate decisions without the patient attending in person. After 4



For peer review only - http://bmjopen.bmj.com/site/about/guidelines.xhtml

(17%) appointments they answered "*neutral*". 14 (93%) patients said they would use TiM if they were unable to travel to clinic and 11 (73%) felt that they would be happy to have their appointment delayed if the doctor felt they were stable. Three (20%) were unsure and one (7%) disagreed. When interviewed, participants thought that the individual should choose how they used TiM depending on their needs, preferences and speed of progression. Some patients who were progressing slowly did not feel frequent MDCs were valuable. They were happy to use TiM and reduce the frequency of appointments as long as they could access the team quickly if required. Others wanted to attend the MDC to receive psychological support or address their problems and did not want to reduce visits. Some were not keen on telephone appointments, particularly when discussing sensitive matters or when they had speech difficulties. Similarly, the telehealth nurse explained face-to-face meetings helped her develop her relationship and gain information about the patient and carer. She felt some aspects of their care would need a combination of TiM system and communication with the patients/carers and other members of the MDT. Both clinicians and patients identified the need for face-to-face consultation to fully evaluate respiratory symptoms.

**Impact on carers**

Carers felt that their wellbeing was directly linked to the patients' wellbeing: interventions that improved the patient's life or carer's ability to provide care could improve a carer's wellbeing. Carers felt reassured that their loved one was being monitored and that problems could be identified and solved more rapidly.

*"I think the benefit to P is real. Because ... somebody is there on hand looking at things...Because it's slow with P and he doesn't need as much attention and care, it's easy to feel detached from any positive interaction. Whereas with [TiM], you know somebody's there and if there was something you'd pick up quite quickly as apposed to, waiting until your next twelve week appointment" Carer 122*

Carers and the telehealth nurse reported that the information highlighted by TiM system could help couples accept the diagnosis and the problems they were facing, and enable them to look positively towards receiving additional medical and social care. Carers reported TiM prompted them to consider their own wellbeing and consider help if they were experiencing difficulties. They explained that usually the patient was present in meetings and healthcare professionals tended to focus on the patient's needs. This meant it was difficult for them to discuss their own concerns because of the potential impact this may have on the patient. Carers thought that TiM provided an opportunity for them to express their feelings in private, separately from the patient but still in the convenience of the home environment, without having to leave the patient. The impersonal nature of TiM was also an advantage as it enabled carers to be more honest about their difficulties.

*"I think it's a really good way of doing it. Because whereas I probably try and flower things up a lot of the time ...I found that, because it was just me and the tablet and I was able just to be totally honest about how I was… feeling at that particular time… The impersonal*


For peer review only - http://bmjopen.bmj.com/site/about/guidelines.xhtml

*format… of the way it was actually done... for me, has been a real help just to be able… to do that." Carer 409*

## Problems with TiM

*[Online Supplementary data file Results Quotes and Figures Process evaluation 2019 tables 16-18]*

Whilst the participants did react positively to TiM, many felt it had not had a significant impact on their care. At six months, only seven (47%) patients and five (33%) of carers agreed, *"The MND team contacted me quickly if my condition changed or I had a problem*". In some cases participants felt their condition had not changed during the trial so TiM had not highlighted any problems. In other cases, problems were reported and help was offered but declined either because the participant did not want it or because they did not think the MDT could help. However, the main explanation for this perceived lack of impact related to the large number of alerts generated by TiM, the way in which the telehealth nurse acted upon information and lack of interaction between the participants and the telehealth nurse when problems occurred.

*TiM system alerts*

Of the total 585 TiM sessions completed, only 19 (3%) of all the patient/carer sessions return answers all of which were flagged as green. 322 (55%) of sessions contained answers which generated at least one red flag and 244 (42%) generated amber alerts (Figure 5a). We analysed individual answers and clinical notes for ten randomly selected patients (334 sessions). Bulbar questions caused most red alerts (usually due to swallowing difficulties or excessive saliva), followed by limb function (usually due to falls) and respiratory symptoms. In the 334 sessions analysed we identified 99 notes made by clinicians. 32 (32%) notes reported the telehealth nurse taking action (Figure 5b): commonly providing telephone advice or liaising with the MDT to share information and coordinate care. 17 (17%) times she planned to review the problem when the patient next came to clinic. In over half of occasions the telehealth nurse documented that she took no action and in only 18 (18%) occasions did she contact the patient or carer. She explained that she usually took no action because she already was aware of the problem, the participant was awaiting treatment or had declined to accept the recommended treatment/advice. She felt that she was seeing the same problems repeatedly. This was particularly frustrating when she had already taken action or when she felt there was no action required. Whilst she did not feel TiM took up a lot of her time, these multiple problems for which she saw no solution caused an element of psychological burden. She could not control how the alerts were generated and would prefer to be able to "*pause*" these alerts.

*Patient-nurse interaction and feedback*

Some participants recalled the telehealth nurse contacting them after reporting a problem on the TiM. The nurse would usually offer reassurance or new advice. Whilst the telehealth nurse felt that these interactions did not have an impact, in the face of a relentlessly progressing disease, these interactions were welcomed



For peer review only - http://bmjopen.bmj.com/site/about/guidelines.xhtml

by patients and carers and it reinforced their perception that specialists were monitoring them closely.

*"...I did the second questionnaire, and within a day [Telehealth nurse] called.*
*Q: Were you expecting her to call?*
*P: No, I wasn't actually. It was just a bolt out of the blue...I find that quite positive. It shows that the whole idea of it works." Patient 122*

The telehealth nurse prioritised problems that were unexpected and placed less priority on problems she expected to occur in MND. Contacting the patient depended on whether the problem needed or had a solution.

*"She red flagged that she'd fallen, which is quite a common occurrence on a lot of patients, and I don't particularly worry unless they've been very, very well and then suddenly." telehealth nurse*

Problems the nurse perceived not to be significant were often felt to be important to patients and carers. For them, acknowledgement was helpful, even if no solution was needed.

*"[falls] ... knocks your confidence ...I probably were putting too much onus on [the hospital MDC] because (laughs) we've got this and there's not [much] they can really do about this and we know that...*
*Q: But that kind of acknowledgement's quite important?*
*C: I do, yeah, it's a bit of support, in't it, it's knowing that somebody else is in your corner." Carer 423*

The telehealth nurse found it easier to manage patients and carers whom she already knew well. She developed relationships through hospital visits and a telephone helpline and through liaison with the community MDT. When the telehealth nurse had less knowledge of the patient it could be more difficult to interpret the information from TiM and liaison with the MDT was less easy. She also explained that she often did not know carers well and sometimes felt there were less obvious solutions to carer distress making her reluctant to telephone. In contrast, most carers felt extremely positive that the nurse provided vital counseling and advice.

The telehealth nurse felt that a more formalised protocol, outlining how she should respond to alerts, would likely increase the number of telephone calls made to patients. However, she felt it was important that she could use her knowledge and experience to decide how to respond to the information presented and would find it difficult working to an overly prescriptive protocol.

**Improvements made to TiM during trial**

Additional questions were added to the patient app: these recorded bladder and bowel symptoms and recorded disease progression in those with more severe disability (using the ALSFRS-EX (33)). Participants felt it was very important that the information provided was accurate and sometimes wanted to give more information in their answers. A free text facility was added but this was used



For peer review only - http://bmjopen.bmj.com/site/about/guidelines.xhtml

very infrequently. We added a heatmap (Figure 4) to the clinical portal which displayed a visual representation of patients' answers over the previous six months. We added the ability for the telehealth nurse to temporarily pause alerts. The telehealth nurse found the heatmap helpful to enable her to get an overview of things she thought were important, such as weight and to see which category had changed. She also found the ability to pause alerts helpful.

**Recommended improvements to TiM**

*[Online Supplementary data file Results Quotes and Figures Process evaluation 2019 tables 20-22)*

The most important improvement we identified was to facilitate communication between the nurse and participants. Most participants did not want automatic feedback on their progress from TiM: they could judge their progress themselves. They were also worried how they would react to seeing objective measures of decline without any interpretation from their clinical team. However, many expected some feedback. The minimum expected was to know that their information had been received and reviewed and for their problems to be acknowledged in some way, even if no action was taken. Participants were asked about their preferences for communication. Telephone and face-to-face conversations were felt to be important for sensitive or complex discussions.

*"I think it's not personal... I'd rather see somebody or talk to somebody than, than read about it on a screen." Carer 366*

Patients found email helped them communicate at their own pace. This was particularly helpful for those with speech problems where a phone call would be difficult. Participants were happy waiting for a definitive answer if they received an acknowledgement. Participants also thought TiM could be used to arrange appointments and other administrative matters and they were happy for their answers to be shared with other clinicians. The Community MND nurse felt TiM information would be useful but the clinical portal would be difficult to access in the community without a computer. She also explained she was *"not good with computers."* but would be happy receiving alerts and information from someone else.

## DISCUSSION

This detailed study of a digitally-enabled MND service confirmed TiM was acceptable and feasible for patients, carers and staff. Differences in outcomes between the two groups were not formally assessed, however, our findings suggest that the TiM was accurate and safe. The study also gained a deeper understanding of the potential value of TiM and the context in which benefits may might be seen (34). We identified some key barriers and enablers to acceptance and use of telehealth which are applicable to other digitally enabled care systems. A key initial barrier for participants was their perceived ability to master the technology. However, uptake was good with most participants (even those with severe disabilities) finding that they were able to use the system and sustain a good level of TiM adherence. The interviews suggested that this was

For peer review only

because of the accessible software and face-to-face training provided. This is in contrast to other telehealth projects which found acceptance and adherence were major barriers (35). This study aligns with other evidence that suggests that patients have a positive attitude to digital products and, in the right setting, respond positively to digitally-enabled care, as long as the service is accessible and continues to offer personal contact with healthcare professionals (36,37) (36). We recommend new interventions should adopt our user-centred design process and offer additional support to those without confidence in technology to make the intervention accessible.

The potential impacts of digitally-enabled care may differ depending on a patient's needs. TiM offered those with early or slowly progressive disease reassurance, information and the ability to remain in contact with the care team when they needed it as well as offering an alternative to clinic visits. Early identification and coordinated management of complications of the disease appears to be most valuable to those patients at a later stage of the disease or those with more rapidly changing disease. These factors may explain why MDC care improves survival. The study also highlighted the potential value of TiM to carers who could receive support separately from the patient, whilst still allowing them to fulfill their role as a carer. Patients and carers already use digital technology to seek reliable and relevant information in order to self-manage (36) and the TiM can be used to provide information and promote self-management and self-efficacy (something which has a positive influence of patient quality of life (38)). This type of interaction also promotes good adherence to telehealth (39). It was therefore interesting to find that participants in this study did not use the additional education services on TiM that frequently. This may be because many patients wish to deal with problems as they occur, rather than learning extensively about their condition (36). Telehealth could address this by providing a more personalised information resource with topics and pace appropriate for the user according to their circumstances and the answers they provide on TiM.

The main weakness of TiM was the mismatch between staff actions and the expectations of participants. This was due to lack of communication and high numbers of unnecessary alerts generated by TiM. MND progression is experienced as a series of losses (40,41) and it is important for staff to acknowledge these problems and recognise the value that interactions with clinicians offer to patients and carers. Remote technology risks disrupting the traditional way in which the nurse would gather information, develop relationships and respond to patient difficulties (42). There is a risk of care becoming less patient centre an patients becoming demoralised as the aims of patients/carers and the clinical team no longer align. We recommend care is taken to develop these interpersonal relationships and for staff to be aware of the potential pitfalls of remote management. Key improvements include improving the algorithms to reduce alerts, facilitating feedback and better communication between patients, carers and staff using technology (such as chat facilities, email or telemedicine) and better training of staff who are required to work in ways that differ from the traditional face-to-face model.


For peer review only - http://bmjopen.bmj.com/site/about/guidelines.xhtml

The key strength of this study was the depth in which we examined the processes occurring in order to explain not just what happened, but why events occurred and how TiM could be improved and implemented. However, this was a small study involving only one centre. As some patients died or withdrew early in the study, of the 20 assigned the TiM a maximum of 17 patients were using the TiM at any one time. Only a small number of clinicians, many of whom had been helped with the TiM development were involved. In particular, only one telehealth nurse used TiM and this study showed that clinicians' behaviour clearly influences the way in which the intervention is delivered. Larger studies are needed to gain a true understanding of what would occur when the service is offered to all patients as "*business as usual*", in different centres using different staff members. Other staff may behave differently and our results are a reminder that they way they use the TiM warrants monitoring to ensure intervention fidelity and safety. The other limitations of evaluating complex interventions such as telehealth in traditional trials are discussed in detail in the parallel paper. We recommend that future evaluations should retain the methods employed in this trial to understand processes in detail, and examine implementation in different contexts, continuing to examine factors such as acceptability, adoption, fidelity, safety, costs and sustainability.

**Conclusion**

The TiM system was acceptable to patients and carers with good uptake and adherence. Key to its success will be continued user and staff engagement and ensuring the system complements the existing services. Improvements to TiM should be based on the findings of this evaluation. Future process evaluations should also examine the implementation of TiM at other sites to determine whether the findings from this study can be generalised.

**Funding and acknowledgements**

The TiM was developed within a collaboration between the University of Sheffield, Sheffield Teaching Hospitals NHS Trust, Mylan Ltd and Abbott Healthcare. This trial was funded by a National Institute for Health Research (NIHR) Doctoral Research Fellowship grant to EH (DRF-2013-06-076) and the Motor Neuron Disease Association. The trial was supported by the Sheffield Teaching Hospitals NHS Trust NIHR Clinical Research Facility and the University of Sheffield Clinical Trials Unit. Mylan Ltd supplied the software and hardware required for the trial. EH is also funded by an NIHR Clinical Lecturer award. PJS is supported by NIHR Senior Investigator award NF-SI-0512-10082 and PJS and CMD by the NIHR Sheffield Biomedical Research Centre (Translational Neuroscience) IS-BRC-1215-20017.

This paper presents independent research funded by the National Institute for Health Research. The views expressed are those of the author(s) and not necessarily those of the NHS, the NIHR or the Department of Health.



For peer review only - http://bmjopen.bmj.com/site/about/guidelines.xhtml

## Conflict of interest

The TiM intellectual property is owned by Mylan and the University of Sheffield. There are no individual conflicts of interest.

## Author contributions

The following were involved: intervention development (CMD, EH, TW, PJS), trial design and management (EH, MB, WO, CC, AQ, CMD), clinical oversight (CMD and PJS), recruitment and intervention delivery (EH and TW) data collection (EH, WB), data analysis (EH, MB, WB, CMD, CC, SM, AQ) and manuscript preparation (All). All authors reviewed and commented on the manuscript. The authors are grateful to Sheffield MND Research Advisory Group for their guidance on the research methods.

For peer review only

## Figure legends

Figure 1
Patient and carer satisfaction with TiM telehealth at 6 months.

Figure 2
A visual representation of individual patient adherence over the duration of the trial. Each black dot indicates a completed TiM session. A coloured dot indicates the end of follow-up.

Figure 3
A visual representation of individual carer adherence over the duration of the trial. Each black dot indicates a completed TiM session. A coloured dot indicates the end of follow-up.

Figure 4
The "heat map" for Patient 409. This is a screenshot from the clinical portal. The heatmap reports the highest level flag for each section of the questionnaire (mobility, bulbar, breathing, wellbeing, nutrition) at each week. This indicates the patient has red and amber flags for both bulbar and later mobility. Below is the weight compared to the patients' weight at baseline demonstrating a greater than 15% weight loss.

Figure 5
Figure 5a (left) The frequency of flags generated by the TiM sessions. The total sessions reports the frequency of "top level" flag (green, amber, red) for all 585 sessions completed. Below are the frequencies of "section flags" generated by 10 patients who completed a total of 334 sessions. 5b (left) Actions described in 99 clinical notes. P: patient, C: carer, TN: telehealth nurse

For peer review only

1. McDermott CJ, Shaw PJ. Diagnosis and management of motor neurone disease. *BMJ*. 2008;336:658–62.

2. Traynor BJ, Alexander M, Corr B *et al.* Effect of a multidisciplinary amyotrophic lateral sclerosis (ALS) clinic on ALS survival: a population based study, 1996-2000. *J Neurol Neurosurg & Psychiatry*. 2003;74:1258–61.

3. Ng L, Khan F. Multidisciplinary care for adults with amyotrophic lateral sclerosis (ALS)/motor neuron disease (MND). *Cochrane Database Syst Rev.* 2009;13:105.

4. Aridegbe T, Kandler R, Walters SJ *et al.* The natural history of motor neuron disease: assessing the impact of specialist care. *Amyotroph Lateral Scler Frontotemporal Degener*. 2013;14:13–9.

5. Rooney J, Byrne S, Heverin M *et al*. A multidisciplinary clinic approach improves survival in ALS: a comparative study of ALS in Ireland and Northern Ireland. *J Neurol Neurosurg & Psychiatry*. 2015;86:496–501.

6. Martin S, Trevor-Jones, A, Khan S *et al*. The benefit of evolving multidisciplinary care in ALS: a diagnostic cohort survival comparison. *Amyotroph Lateral Scler Frontotemporal Degener*; 2017;7-8:569-575.

7. Moura MC, Casulari LA, Garbi Novaes MRC. Multidisciplinary Care Improves Survival of Patients with Amyotrophic Lateral Sclerosis in the Unique Health System (SUS) in Brazil. *J Neurol Disord*. 2017;05:1–7.

8. Motor Neurone Disease Association UK. Personal communication. 2016.

9. Hobson EV, Baird WO, Cooper CL *et al*. Using technology to improve access to specialist care in amyotrophic lateral sclerosis: A systematic review. *Amyotroph Lateral Scler Frontotemporal Degener.* 2016;5-6:313–24.

10. Andrew G, Courtney W, Anne M, Susan W, Bethany S, Zachary S. Incorporation of telehealth into a multidisciplinary ALS Clinic: feasibility and acceptability. Amyotroph Lateral Scler Frontotemporal Degener. Informa UK Limited, trading as Taylor & Francis Group; 2017 Nov 8;0(0):555–61.

11. Selkirk SM, Washington MO, McClellan F *et al*. Delivering tertiary


For peer review only - http://bmjopen.bmj.com/site/about/guidelines.xhtml

centre specialty care to ALS patients via telemedicine: a retrospective cohort analysis. *Amyotroph Lateral Scler Frontotemporal Degener.* 2017;(5-6):324–32.

12. Van De Rijn Marc, Sabrina P, Mark L-W, Katharine C, Amy SE, Juan E, et al. Experience with telemedicine in a multi- disciplinary ALS clinic. *Amyotroph Lateral Scler Frontotemporal Degener*; 2018;0(0):1–6.

13. Henderson RD, Hutchinson N, Douglas JA, Douglas C. Telehealth for motor neurone disease. *Med J Aust*. 2014;201(1):31.

14. Care augmentation by location-link messaging. Call-Me. www.isrctn.com/ISRCTN71271888. [Website. Date accessed April 2019]

15. ALS at home. als-at-home.org. [Website. Date accessed April 2019]

16. Answer ALS. www.answerals.org [Website. Date accessed April 2019]

17. ALS Analyzer. www.alsanalyzer.com. [Website. Date accessed April 2019]

18. O'Brien M, Whitehead B. Optimisation of service for people with Motor Neurone Disease (MND) in Lancashire and South Cumbria. 2010 Jul.

19. Baxter SK, Baird WO, Thompson S *et al*. The impact on the family carer of motor neurone disease and intervention with noninvasive ventilation. *J Palliat Med.* 2013;16(12):1602–9.

20. ProGas Study Group. Gastrostomy in patients with amyotrophic lateral sclerosis (ProGas): a prospective cohort study. *Lancet Neurol.* 2015;14(7):702–700.

21. Stavroulakis T, Baird WO, Baxter SK *et al*. The impact of gastrostomy in motor neurone disease: challenges and benefits from a patient and carer perspective. *BMJ Support Palliat Care.* British Medical Journal Publishing Group; 2016;6(1):52–9.

22. Bergin S, Mockford C. Recommendations to support informal carers of people living with motor neurone disease. *British Journal of Community Nursing*. 2016;21(10):518–24.

23. Galvin M, Carney S, Corr B, *et al*. Needs of informal caregivers across the caregiving course in amyotrophic lateral sclerosis: a qualitative analysis. *BMJ Open.* 2018;8(1):e018721.

24. Vitacca M, Comini L, Tentorio M, *et al*. A pilot trial of telemedicine-assisted, integrated care for patients with advanced amyotrophic



For peer review only - http://bmjopen.bmj.com/site/about/guidelines.xhtml

lateral sclerosis and their caregivers. *J Telemed Telecare.* 2010;16(2):83–8.

25. Creemers H, Veldink JH, Grupstra H, *et al* Cluster RCT of case management on patients' quality of life and caregiver strain in ALS. *Neurology*. 2014;82(1):23–31.

26. Hobson EV, Baird WO, Partridge R, *et al*. The TiM system: developing a novel telehealth service to improve access to specialist care in motor neurone disease using user-centered design. *Amyotroph Lateral Scler Frontotemporal Degener.* 2018;19:351-361

27. Medical Research Council. A framework for development and evaluation of RCTs for complex interventions to improve health. *MRC*. 2000.

28. Steventon A, Bardsley M, Billings J *et al*. Effect of telehealth on use of secondary care and mortality: findings from the Whole System Demonstrator cluster randomised trial. *BMJ*. 2012;344:e3874–4.

29. Fan VS, Gaziano JM, Lew R *et al*. A comprehensive care management program to prevent chronic obstructive pulmonary disease hospitalizations: a randomized, controlled trial. *Ann Intern Med*; 2012;156:673–83.

30. Takahashi PY, Pecina JL, Upatising B *et al*. A randomized controlled trial of telemonitoring in older adults with multiple health issues to prevent hospitalizations and emergency department visits. *Arch Intern Med*. 2012;172:773–9.

31. Craig P, Dieppe P, Macintyre S, *et al*. Developing and evaluating complex interventions: the new Medical Research Council guidance. *BMJ*; 2008;337:a1655–5.

32. Moore GF, Audrey S, Barker M, *et al*. Process evaluation of complex interventions: Medical Research Council guidance. *BMJ*. 2015 Mar 19;350:h1258.

33. Wicks P, Massagli MP, Wolf C, *et al*. Measuring function in advanced ALS: validation of ALSFRS-EX extension items. *Eur J Neurol*. 2009;16(3):353–9.

34. Lamont T, Barber N, de Pury J *et al*. New approaches to evaluating complex health and care systems. *BMJ*. 2016;352:i154–5.

35. Sanders C, Rogers A, Bowen R *et al*. Exploring barriers to participation and adoption of telehealth and telecare within the Whole System Demonstrator trial: a qualitative study. *BMC Health Serv Res*. 2012;12:1–12.

For peer review only

36. Hobson EV, Fazal S, Shaw PJ *et al.* "Anything that makes life's journey better." Exploring the use of digital technology by people living with motor neurone disease. *Amyotroph Lateral Scler Frontotemporal Degener*. 2017;34:1–10.

37. James N, Power E, Hogden A, Vucic S. Patients' perspectives of multidisciplinary home-based e-Health service delivery for motor neurone disease. *Disabil Rehabil Assist Technol*. 2018;14:1–7.

38. Galin S, Heruti I, Barak N, *et al*. Hope and self-efficacy are associated with better satisfaction with life in people with ALS. *Amyotroph Lateral Scler Frontotemporal Degener*. 2018;19(7-8):611–8.

39. Maeder A, Poultney N, Morgan G, Lippiatt R. Patient Compliance in Home-Based Self-Care Telehealth Projects. *J Telemed Telecare*. 2015;21(8):439–42.

40. Foley G, Timonen V, Hardiman O. Exerting control and adapting to loss in amyotrophic lateral sclerosis. *Soc Sci Med*. 2014;101:113–9.

41. Mistry K, Simpson J. Exploring the transitional process from receiving a diagnosis to living with motor neurone disease. *Psychol Health*. 2013;8:939–53.

42. Greenhalgh T, A'Court C, Shaw S. Understanding heart failure; explaining telehealth – a hermeneutic systematic review. *BMC Cardiovasc Disord*. 2017;17,1-16

For peer review only



For peer review only - http://bmjopen.bmj.com/site/about/guidelines.xhtml

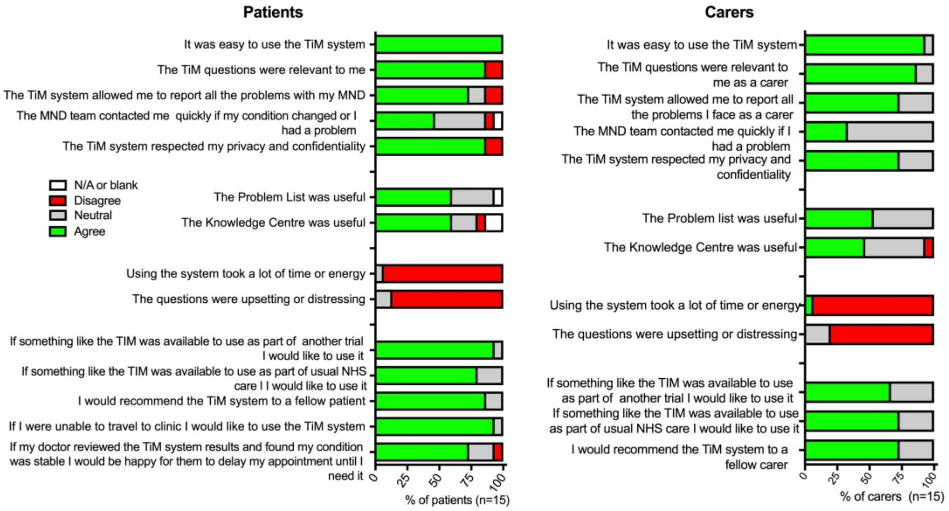


Patient and carer satisfaction with TiM telehealth at 6 months.

264x138mm (300 x 300 DPI)

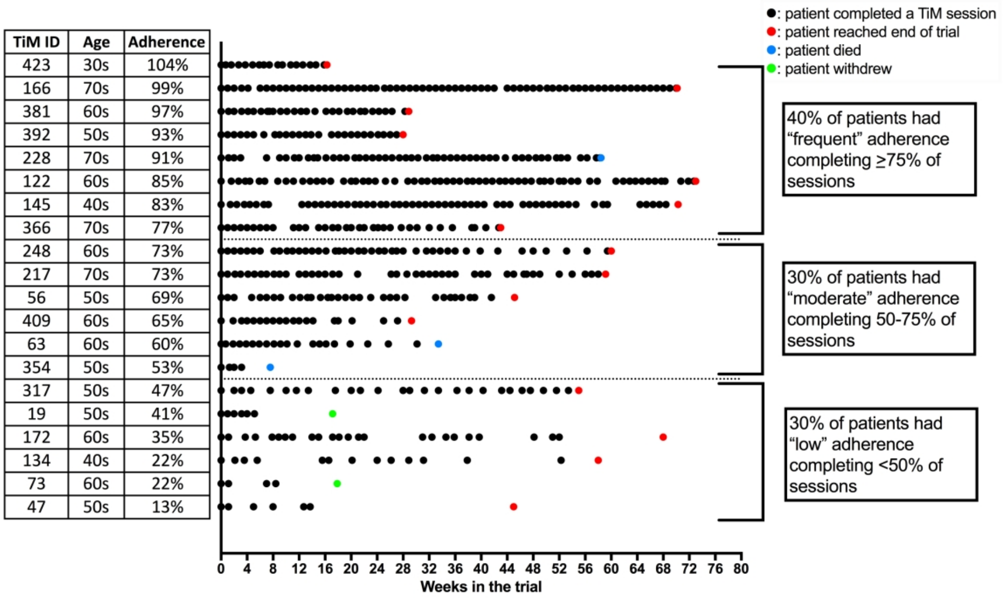


A visual representation of individual patient adherence over the duration of the trial. Each black dot indicates a completed TiM session. A coloured dot indicates the end of follow-up.

275x166mm (300 x 300 DPI)

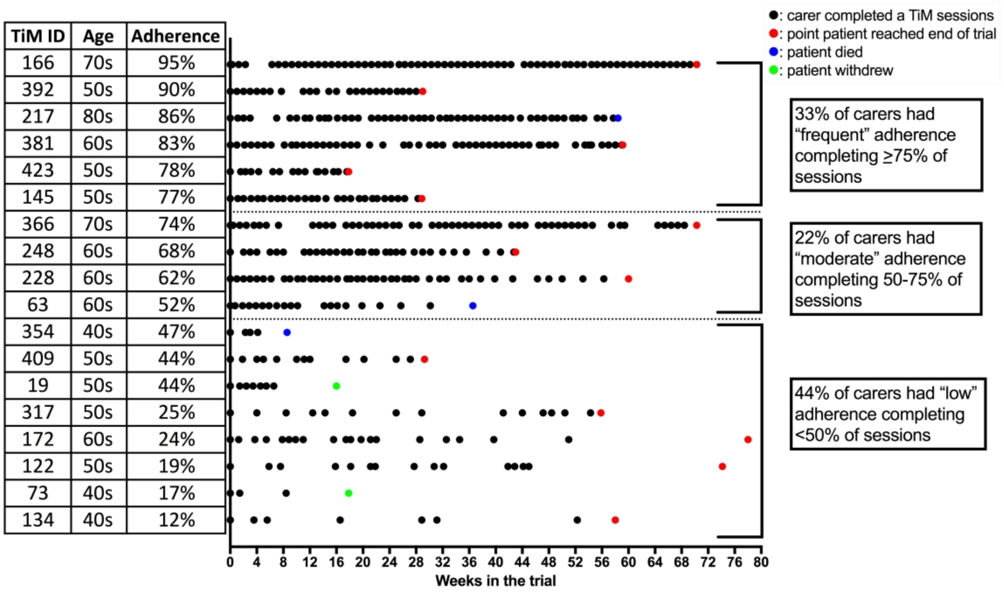

| TiM ID | Age | Adherence |
|---|---|---|
| 166 | 70s | 95% |
| 392 | 50s | 90% |
| 217 | 80s | 86% |
| 381 | 60s | 83% |
| 423 | 50s | 78% |
| 145 | 50s | 77% |
| 366 | 70s | 74% |
| 248 | 60s | 68% |
| 228 | 60s | 62% |
| 63 | 60s | 52% |
| 354 | 40s | 47% |
| 409 | 50s | 44% |
| 19 | 50s | 44% |
| 317 | 50s | 25% |
| 172 | 60s | 24% |
| 122 | 50s | 19% |
| 73 | 40s | 17% |
| 134 | 40s | 12% |



A visual representation of individual carer adherence over the duration of the trial. Each black dot indicates a completed TiM session. A coloured dot indicates the end of follow-up.

277x165mm (300 x 300 DPI)

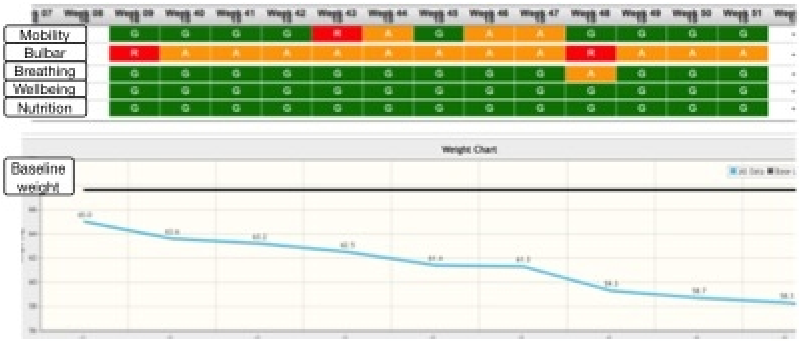


The “heat map” for Patient 409. This is a screenshot from the clinical portal. The heatmap reports the highest level flag for each section of the questionnaire (mobility, bulbar, breathing, wellbeing, nutrition) at each week. This indicates the patient has red and amber flags for both bulbar and later mobility. Below is the weight compared to the patients’ weight at baseline demonstrating a greater than 15% weight loss.

140x59mm (72 x 72 DPI)

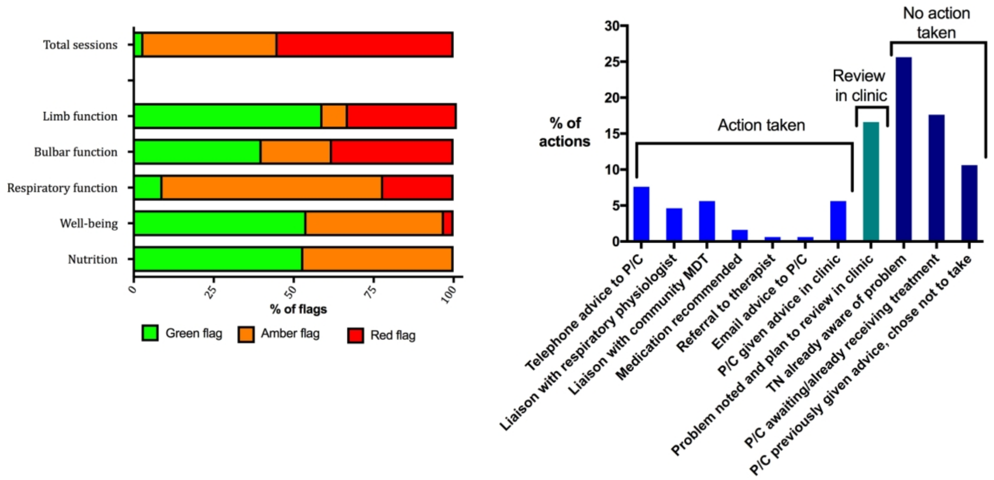


Figure 5a (left) The frequency of flags generated by the TiM sessions. The total sessions reports the frequency of "top level" flag (green, amber, red) for all 585 sessions completed. Below are the frequencies of "section flags" generated by 10 patients who completed a total of 334 sessions. 5b (right) Actions described in 99 clinical notes. P: patient, C: carer, TN: telehealth nurse

193x97mm (300 x 300 DPI)

**Telehealth in motor neurone disease to increase access to specialist multidisciplinary care: a pilot, feasibility study.**

Hobson EV., Bradburn M., Baird WO, Cooper CL, Mawson S, Quinn A, Shaw PJ, Walsh T, McDermott CJ

**Supplementary data files**

**TiM protocol V1.5.................................................Page 2**
**TiM statistics analysis plan V1.3.......................Page 45**
**Shadow monitoring form ..................................Page 71**

For peer review only

TiM™

**Telehealth in Motor Neurone Disease**

# Telehealth in Motor Neurone Disease: A single centre, randomised controlled feasibility and pilot study of the use of the TiM telehealth system to deliver highly specialised care in Motor Neurone Disease at a distance

For peer review only

The University Of Sheffield.

Sheffield Teaching Hospitals NHS
NHS Foundation Trust

D4D
Devices for Dignity

SITraN
Sheffield Institute for Translational Neuroscience

CLAHRC for South Yorkshire



For peer review only - http://bmjopen.bmj.com/site/about/guidelines.xhtml

## General Information

**Sponsor identifier**: 17165
**National Institute for Health Research Portfolio ID**: 17022
**Trial Identifier:** ISRCTN26675465

**Sponsor**

Sheffield Teaching Hospitals NHS Foundation Trust
Tanya.turgoose@sth.nhs.uk
Sheffield Teaching Hospitals NHS Foundation Trust Research Department
11 Broomfield Road Sheffield S10 2SE
Tel: (0114) 2265942
Fax: (0114) 2265937
Email: Tanya.Turgoose@sth.nhs.uk

**Principal investigator & Trial Manager**

Dr Esther Hobson
National Institute for Health Research Doctoral Research Fellow
Neurology Specialty Registrar
Sheffield Institute of Translational Neuroscience University of Sheffield
385a Glossop Road
Sheffield
S10 2HQ
Tel: (0114) 2222261
Fax: (0114) 2222290
Email: esther.hobson@gmail.com

**Chief investigator**

Dr. Christopher McDermott
Reader and Honorary Consultant Neurologist
Sheffield Institute of Translational Neuroscience University of Sheffield
385a Glossop Road
Sheffield
S10 2HQ
Tel: (0114) 2222261
Fax: (0114) 2222290
Email: c.j.mcdermott@sheffield.ac.uk

This study is a non-commercial, portfolio study supported by the DeNDRoN (Dementia and Neurodegenerative Diseases Clinical Research Network). It is funded through a National Institute for Health Research Doctoral Research Fellowship grant.



For peer review only - http://bmjopen.bmj.com/site/about/guidelines.xhtml

Abbreviations

| | |
|---|---|
| AE | Adverse event |
| ALS | Amyotrophic lateral sclerosis |
| ALSAQ-40 | Amyotrophic Lateral Sclerosis Assessment Questionnaire – long form |
| ALS-FRS-R | Amyotrophic lateral sclerosis rating scale-revised |
| BiPAP | Bi-level positive pressure non-invasive ventilation |
| CBI | Carer burden inventory |
| CI | Chief Investigator |
| CLRN | Comprehensive Local Research Network |
| CONSORT | Consolidated standards of reporting trials |
| CRF | Clinical Research Facility, Sheffield Teaching Hospitals |
| CSS-MND | Clinical Saliva Scale for Motor Neurone disease |
| CTRU | Clinical trials research unit, University of Sheffield |
| EQ-5D-3L | EuroQol Group Health Questionnaire, |
| GCP | Good clinical practice |
| GP | General practitioner |
| HADS | Hospital Anxiety and Depression Scale |
| HTA | Health Technology Assessment |
| MCSI | Modified Caregiver Strain Index |
| MDT | Multidisciplinary team |
| MND | Motor neurone disease |
| NICE | National Institute for Health and Clinical Excellence |
| NHS | National Health Service |
| NIHR | National Institute for Heath Research |
| NIV | Non-invasive ventilation |
| PHQ | Patient Health Questionnaire |
| PI | Principal Investigator |
| SAE | Serious adverse event |
| ScHARR | School of Health and Related Research, University of Sheffield |
| SF-36 RAND | 36-Item Short Form Survey from the RAND Medical Outcomes Study |
| SITraN | Sheffield Institute of Translational Neuroscience |
| STH | Sheffield Teaching Hospitals |
| SU | Sheffield University |
| Telecare | A system of sensors, alarms or communication in the home used to support safe living |
| Telehealth | Remote monitoring of patients physiology or patient reported measures, forwarded to a central service with the aim to diagnoses or monitor a medical condition |
| Telemedicine | Videoconferencing consultation |
| TMG | Trial management Group |
| TSC | Trial Steering Committee |
| TiM | Telehealth in Motor neurone disease |
| TM | Trial manager |

For peer review only

**Contents**

1. Title and abstract
2. Lay summary
3. Background
4. Summary and hypothesis
5. Research objectives
    a. Objective of the pilot study
    b. Objectives of the full scale study
    c. Justification of the pilot study
6. Study Methodology
    a. Participant recruitment and selection
    b. Consent
    c. Randomisation
    d. Inclusion and exclusion criteria
    e. Withdrawal
    f. Compliance
    g. Sample size
    h. Blinding
7. Study treatment
8. Data collection
    a. Quantitative data collection
    b. Qualitative sub-study
    c. Shadow monitoring protocol
    d. Process evaluation
9. Analysis
    a. Feasibility and quantitative analysis
    b. Qualitative analysis
10. Data entry, security and confidentiality
11. Safety and safety assessments
12. Ethical considerations
13. Finance and indemnity
14. Dissemination and publication
15. References

Appendix 1 Drugs produced by Abbott Healthcare Ltd.
Appendix 2 CONSORT flow diagram
Appendix 3 Qualitative interview topic guides

This document describes a clinical study. The protocol is not intended for use as a guide to the treatment of other patients. Amendments may be necessary; these will be circulated to investigators in the study.



For peer review only - http://bmjopen.bmj.com/site/about/guidelines.xhtml

## 1. Telehealth in Motor Neurone Disease: The TiM ™ Study

Telehealth in Motor Neurone Disease: A single centre, randomised controlled pilot study of the use of the TiM ™ telehealth system to deliver highly specialised care in Motor Neurone Disease at a distance.


### Abstract

### Objectives

People with motor neurone disease benefit from the specialist care provided by multidisciplinary teams. As their disease progresses patients struggle to attend hospital and find it difficult to access the care they need. The aim of the TiM system is to improve access to this specialist care by using technology to monitor, educate and communicate with our patients and their carers.

This is a pilot study of the TiM ™ telehealth system. The pilot study is designed to assess the feasibility, acceptability and safety of the telehealth system in clinical practice and of conducting a full study of the system. It will also allow a process evaluation of the system to determine how the telehealth system could be effectively utilised within an NHS service.

### Methods

This is a single-centre, randomized controlled mixed methods pilot study of the TiM ™ telehealth system. It will recruit 40 patients along with their primary informal carer. 20 will be assigned to use the TiM system for a minimum of 6 months (intervention) and 20 will be assigned usual care (control). Quantitative outcome data will be collected at baseline, three and six months, six monthly thereafter and at the end of the trial. Qualitative interviews with participants and staff and analysis of the system in use will enable a process evaluation of the system and the trial methodology. It will also assess the safety of the system in a clinical setting.

### Results

Results of this pilot will determine whether a large, multi-centre full trial is appropriate and enable further development of the TiM system. It is proposed that the TiM system could be adopted into the care of patients with MND throughout the UK.




For peer review only - http://bmjopen.bmj.com/site/about/guidelines.xhtml

## 2. Lay summary

Motor neurone disease is a condition affecting approximately 5 000 people in the UK. It results in progressive weakness in muscles causing paralysis, disability, and eventually death after an average of only three to five years. To receive the expert care provided by motor neurone disease multidisciplinary teams most patients have to travel to regional centres, whilst community-based care is usually provided by non-specialist teams. Between clinic appointments and towards the end of their lives when patients are unable to travel to clinic and they may be unable to access the specialist assessment and care provided in these centres.

Telehealth has been shown to increase access to specialist care in patients with chronic disease, regardless of geography or the ability to travel. The overall purpose of this pilot study is to test the feasibility and acceptability of the TiM telehealth system. The TiM system is web-based system that enables weekly monitoring of patients and carers' health and wellbeing. It has been developed by the Sheffield Motor Neurone Disease Care Centre team in partnership with industry (Abbott Healthcare Products Ltd and Carematix) and other experts within the NHS and the University of Sheffield. The study will also determine whether it would be feasible to conduct a larger study of the system to examine the effectiveness of the TiM system.

Patients with motor neurone disease who are cared for by the Sheffield motor neurone disease clinic will be invited to take part in the trial. 40 patients will be recruited. Their primary informal carer (usually their spouse or close relative) will also be invited to participate in carer monitoring. All patients will continue their usual care but half will also be randomised to use the TiM telehealth system for a minimum of six months.

Information about the participants will be collected at the start, three and six months, and then every six months until the end of the trial by postal questionnaires and during routine appointments. Up to 20 participants in the control group will be interviewed at the start at the trial to explore their experiences on completing the postal questionnaires. Up to 20 participants who are using the TiM system will be interviewed at one and six months to understand the effect the trial and the system has on their lives in more depth. The clinical staff will also be interviewed at the end of the trial.

A pilot study is a small-scale study that is carried out to determine whether a larger study is practical. It will also enable the identification and resolution of any problems with either the telehealth system or the trial procedures.



For peer review only - http://bmjopen.bmj.com/site/about/guidelines.xhtml

## 3. Background

There are approximately 5 000 people in the UK suffering from motor neurone disease (MND) at any one time (1). MND is an incurable disease causing progressive weakness of muscles involving the limbs, speech and swallowing leading to progressive disability and eventual respiratory failure. The average life expectancy following diagnosis is two to three years but the course of MND can vary from only a few months to over 10 years. The distress and burden of the disease affects patients, their family and carers and the relenting progression of disability causes social, emotional and financial strain (2, 3).

There are 22 specialist multidisciplinary MND care centres in the UK. Expert clinicians and therapists offer interventions such as riluzole (which can improve survival by approximately two to three months) and gastrostomy feeding to promote good nutrition (4). Treatment of respiratory failure with non-invasive ventilation (NIV) improves both quality of life and life-expectancy by, on average, 11 months (5). Attendance at specialist MND clinics has also been reported to improve survival independent of these other interventions (6, 7).

The traditional model of care is to review patients at the MND centre at fixed regular intervals. This model is not responsive to patient or carer needs (which can change rapidly) and requires the patient and their family to undertake progressively more difficult journeys to clinic at a time predicted by the clinician at their last meeting. Given the burden associated with travelling to clinic, it is important that visits occur when they are most needed. Some patients whose needs have not changed may not benefit from a clinic appointment at the previously predicted interval whereas others may need more timely intervention.

The highly specialist services provided by the MND clinic are contrasted by the services most patients receive in their community (8). These community teams, who have limited experience in caring for patients with MND, are usually the first point of contact for patients between clinic visits. Lack of expertise in MND amongst community teams and limited access to specialist staff and equipment (particularly at the end stages of the disease) causes patients and their carers to experience significant difficulties (2, 3, 9-12). Research conducted in SITraN and by others, highlights the major impact that caring for someone with MND has on the physical and emotional well being of carers, as well as patients (3, 11, 13-16). Where access to specialist services and community support is limited, this impact is even more notable (3, 11, 13-16). This is particularly a problem in the later stages, when it is usually impossible to attend clinic, when arguably the most care is needed. It is therefore essential that the input from the specialist centre is still possible both between visits and when patients become unable to travel.

### Telehealth to provide specialist care in MND

In the last few years, technology has developed sufficiently to allow high quality communication between patient and their care team at a potentially reasonable cost.



For peer review only - http://bmjopen.bmj.com/site/about/guidelines.xhtml

Trials have shown that telehealth is an acceptable way to improve access to specialist expertise and facilitate self-management patients with long-term health conditions (17-21). In some cases this approach has been associated with a reduction in hospital admissions (17, 18, 20). In 2012, in response to research evidence and the need to provide cost-effective care to an expanding population of patients with chronic disease, the UK government created the "3millionlives" campaign (22). This project in aims foster NHS, academic and industry collaboration in order to provide telehealth services to up to three million people with chronic health and social care needs in the UK.

The problems faced in MND are unlike many common chronic diseases in which telehealth has been previously trialed. Care for MND requires holistic and multi-disciplinary expertise and the use of uncommon interventions such as non-invasive ventilation and gastrostomy feeding. To date, use of telehealth in MND is limited, although small studies do show promise in certain niche areas. Telehealth systems using telephone consultation have been developed with some success in Italy and Portugal, to remotely manage patients who require home ventilation. These systems were associated with a reduction in emergency healthcare usage; more efficient use of staff time and potential cost savings (23-28) In Holland and rural Scotland, MND services have used video-conferencing (29, 30). Both approaches have potential benefits but telehealth used in this way is labour intensive and costly and care is driven by the priorities identified by the clinician rather than those of the patient. No telehealth system has been developed to provide frequent, holistic and highly specialist care to patients with MND at all stages of their illness.

We propose that telehealth could enable people with MND to have better access to the specialist monitoring and care that they require. Patients with MND are able to accurately report their level of disability and appropriate questions can identify new symptoms or early signs of respiratory failure (31-33). These features would suggest that a system of remote, question-based monitoring could provide regular, accurate clinical information to enable the clinician to detect and better manage problems without the patient needing to attend hospital. Telehealth provides the opportunity to provide education and reassurance and support to enable patients to better manage their own care (a core requirement of the National Service Framework for Long-term Conditions (34)).

There are estimated to be 10 million people in the UK living with a neurological condition (1). Both the common diseases such as Parkinson's disease and epilepsy and rarer conditions such as muscular dystrophy and MND require specialist, multidisciplinary support from specialist services. A successful telehealth system may therefore be able to improve the services provided to many patients in the UK and their families.

**4. Summary and hypothesis**

**Summary**



For peer review only - http://bmjopen.bmj.com/site/about/guidelines.xhtml

We will undertake a pilot study of the use of the TiM telehealth system to improve the care of patients and their carers living with motor neurone disease. Whilst telehealth services have been used successfully in other long-term conditions, no service of this kind exists for patients with motor neurone disease. A pilot randomised controlled trial will employ a mixed methods approach to explore the feasibility and acceptability of using the TiM system to improve access to specialist care in MND. The pilot study will also explore the feasibility of a full-scale trial.

**Hypothesis**

The TiM telehealth system will:

- Improve the quality of life of patients with MND
- Improved clinical outcomes for patients with MND
- Improve quality of life and other measures of well being for the primary informal carers of patients living with MND.
- Be acceptable to patients, carers and staff
- Lead to more cost effective utilisation of heath care resources

For peer review only


For peer review only - http://bmjopen.bmj.com/site/about/guidelines.xhtml

## 5 Research objectives

### 5a. Objectives of the pilot study

- Determine the requirements of a full-scale study of the TiM system
  - Determine recruitment, retention and withdrawal rates.
  - Determine the most acceptable and appropriate outcome measure(s) that reflect the impact of the TiM system on patients and carers and health resources.
  - Provide an estimate of the resources required to conduct a full-scale study.
- Study the use of the TiM system in clinical practice
  - Assess the relationship between the benefits of the TiM system perceived by staff and participants with those captured by the outcome measures
  - Assessing participants' use and compliance with the TiM system
  - Health-care staff qualitative interviews and focus group
- Assess the safety of the TiM system using:
  - A shadow monitoring protocol
  - Health-care staff qualitative interviews and focus group
  - Analysis of technical and clinical adverse events

### 5b. Objectives of the full-scale study

**Proposed primary end-point**

- Patient quality of life (outcome measure(s) to be determined in the pilot trial)

**Proposed secondary end-points**

Patient outcomes

- Severity of pain
- Severity of oropharygeal secretions
- Incidence of depression and anxiety
- Time from diagnosis to death

Carer outcomes

- Quality of life
- Carer Burden
- Incidence of depression and anxiety

Health economic outcomes involving a cost utility analysis using costs of the system, costs of associated care requirements, EQ5D and patient survival

Safety of the TiM system

- Frequency of adverse events

### 5c. Justification of the pilot study



For peer review only - http://bmjopen.bmj.com/site/about/guidelines.xhtml

Since this type of telehealth has never been evaluated in those with MND a pilot study is necessary to determine how a full-scale evaluation of its clinical and cost-effectiveness could be conducted and to gain a better understanding of how the TiM system would work. This includes evaluating recruitment and retention, as well and resource requirements. By evaluating compliance and safety monitoring and using qualitative the study will also enable a better understanding of how the telehealth system is used by patients, carers and staff.

A number of the proposed benefits of telehealth such as improving quality of life, providing reassurance and support, prompting self-care and a more efficient use of resources (18, 35-40) may be difficult to quantify. The validated measures of quality of life most commonly used in research (EQ5D and SF-36) were not specifically designed for patients with MND or their carers. The ALSAQ-40 tool better encompasses dimensions of life that are particularly affected by MND such as social and emotional function but it is unclear whether the ALSAQ-40 would fully reflect the impact of telehealth (41).

Data from quantitative elements of a randomised controlled trial will not, in isolation determine which outcome measures best reflect the impact of the intervention. It would also not fully explain how the TiM system would be used in the real world and what factors would influence its adoption and success. Utilising mixed methods will allow the combination of quantitative data with more in-depth results from qualitative interviews that will explore participants' experiences in more depth. It will also allow explanation of outcomes that occurred (particularly those that were unexpected) and understand why (and in what context) aspects of the system were successful or unsuccessful which could lead to improvements in the TiM system.


For peer review only - http://bmjopen.bmj.com/site/about/guidelines.xhtml

## 6. Study Methodology

We will conduct a randomised controlled pilot trial comparing the TiM telehealth service and standard care with standard care alone. The intervention and follow-up period will be a minimum of 6 months. Quantitative data will be collected at 0, 3 and 6 months then every six months until the patient finishes the trial. Qualitative data will be collected at baseline in the control arm and at 1 and 6 months for a selection of patients in the intervention arm.

### 6a. Participant recruitment and selection

Pre-screening will identify a list of potential patients who cared for by the Sheffield Teaching Hospitals MND care centre clinic as part of usual care using the MND care centre "ARC" clinical database. Each patient will be assigned a number. The Clinical Research Facility at the Royal Hallamshire Hospital, Sheffield Teaching Hospitals NHS trust will generate random numbers to identify the patients to invite. These patients will be sent a letter of invitation to participate. This will be accompanied by patient and carer information leaflets and a return slip to indicate their interest. Those who do not return the slip will be followed up by telephone or at clinic, if appropriate a minimum of once and a maximum of twice. A log will be kept in order to complete the CONSORT diagram (Appendix 2) (42).

Patients and their primary carer who express an interest will be invited to discuss the trial in a face-to-face meeting with the PI and also via telephone with their consultant neurologist (Dr. Christopher McDermott or Professor Dame Pamela Shaw, Sheffield Teaching Hospitals MND Care Centre). The Sheffield MND Care Centre sees approximately 120 new patients with MND per year. At any time there are approximately 300 patients attending clinic. We expect to be able to recruit a minimum of four patients per month.

Participants in the intervention arm will be invited to participate in qualitative interviews, conducted at month one and month six. Purposive sampling will be used to reflect the variation and predefined patient prognostic factors thereby capturing a range of experiences. Interviews will continue until data saturation is reached or 20 interviews have been conducted. All participants assigned to the control arm will be invited to be interviewed after completion of the baseline questionnaires to determine the feasibility and acceptability of these measures and their views on participating in the trial. The qualitative component will provide information not easily obtained from questionnaires that will facilitate understanding of the intervention from the perspective of all stakeholder groups.



For peer review only - http://bmjopen.bmj.com/site/about/guidelines.xhtml

**6b. Consent**

Following indication of their interest to participant potential participants will be met at a mutually agreeable location, preferably the patients' home. They will have further opportunity to discuss the trial with the PI and decide whether they wish to participate. Willing participants will be asked to give informed written consent or use an appropriate witnessed alternative (which may include verbal consent or via a communication device) for screening and involvement in the trial. Carer consent will be obtained by full written consent.

In versions prior to V1.5 of the protocol both patient and carer consent were required. V1.5. has amended the inclusion criteria to allow a patient to participate with carer participation.

If one or both consent to the study a member of the study team will initiate the screening process. Participants will be screened and recruited by the PI according to the CONSORT principles and Good Clinical Practice (42, 43).

Those who decline participation will be invited to give their reasons in order to identify common factors; this may help recruitment strategies and identify potential problems for compliance. Basic anonymised details of these patients (age, gender, reason for exclusion) will be collected on all eligible patients in order to fulfill the CONSORT flow chart (Appendix 2) (42, 43).

**6c. Randomisation**

Once recruited, randomisation will be performed using the independent web-based system http://www.sealedenvelope.com using block randomisation. All patients and carers will be assigned an anonymous individual study code, and a recruitment log held by the research team in SITraN.

For peer review only



For peer review only - http://bmjopen.bmj.com/site/about/guidelines.xhtml

## 6b. Inclusion and exclusion criteria

Inclusion criteria:

- Patients aged 18 years or over who have attended the MND clinic at the Royal Hallamshire Hospital, Sheffield.
- Patients with amyotrophic lateral sclerosis diagnosed by a consultant neurologist with symptom onset within the last three years.
  - Or
- Patients with amyotrophic lateral sclerosis, primary muscular atrophy or progressive lateral sclerosis diagnosed by a consultant neurologist with a deterioration in their condition as evidenced by a deterioration in the ALS functional rating score (ALSFRS-R) by at least two points during the previous 18 months.
- Live within 120 minute drive from Sheffield

Exclusion criteria:

The main circumstances where patients or carers will be excluded are those in which individuals would be unable to use the telehealth system or give informed consent.

- Patients attend another MND care centre in the UK.
- Significant impairment in decision making capacity preventing informed consent by the subject due to a major mental disorder including fronto-temporal dementia.
- Patient unable to use the TiM system due to physical, intellectual or language difficulties and unwilling to permit carer to operate it on their behalf. Patients will be asked to complete two questions used within the TiM system, with, or without the help to their carer to verify their ability to use the system.
- ~~The patient has no eligible informal carer willing to participate in the trial~~ (V1.5)
- Insufficient mobile telephone reception in the patients' home to use the TiM system.
- Any other major impairment that may affect their ability to participate in the study

Carer inclusion criteria

- Age 18 years or older
- Person identified by the patient as the major provider of informal care (emotional and/or practical support) to the patient and provides more than one hour per week of unpaid care
- Carer willing to allow data they provide during the trial to be shared by the research team with their own doctor in the event of serious clinical need.

Carer exclusion criteria

- Significant decision making capacity preventing informed consent due to a major mental disorder.
- Carer unable to use the TiM system due to physical, intellectual or language difficulties. Carers will be asked to complete two questions used within the TiM system to verify their ability to use the system.



For peer review only - http://bmjopen.bmj.com/site/about/guidelines.xhtml

- Inability to participate in the study due to other major physical or mental illness or language difficulties.
- Professional carers receiving direct payment for their services.

**6e. Withdrawal**

Participants will be followed up until the end of the study, death, or withdrawal. Those wishing to withdraw will be given the opportunity to speak to a member of the study team. Participants are free to withdraw from the intervention or study at any time. As a pilot study, importance of understanding reasons for withdrawal is recognised. This will be explained to the participants in the information leaflets. The importance of understanding reasons for withdrawal and the characteristics of these participants is recognised given the nature of the study. This will be explained to the participants in the information leaflets.

**Withdrawal criteria**

1. Patient request
2. Carer request
3. Patient loses capacity to continue to provide consent

If a Patient withdraws from the study arrangements will be made for the equipment to be collected or returned. Where appropriate participants will be invited to give the reasons for withdrawal.

Patients will also be given the option of:

1. Withdrawal from the intervention but remain within the study. Study data will only be collected at clinic visits at 3 and 6 months and six monthly until the end of the study.
2. Withdrawal from the study. Unless the participant objects, any data collected up to this point would be retained and used in study analysis. Participant agrees to allow contact to give safety and survival data.
3. Withdrawal from the study entirely. Unless the participant objects, any data collected up to this point would be retained and used in study analysis. If the participant does not wish to be contacted with regard to safety or survival data, no further contact with regard to this study will be made.

In the event that the patient dies or loses the capacity to provide consent they will be withdrawn from the trial but any data collected up to that point would be retained and used in study analysis. Carers would also be withdrawn at this point.

If the carer participant withdraws the patient can opt to continue to use the system or withdraw. If appropriate, carers will be invited to give reasons for withdrawal.

**6f. Compliance**

Steps have been taken to encourage compliance with the weekly schedule. Patients will receive regular messages on the system to invite them to complete a scheduled telehealth session. They will receive feedback on their compliance record and an


For peer review only - http://bmjopen.bmj.com/site/about/guidelines.xhtml

encouraging message at the start of each visit. Those who fail to complete the session within one day will be reminded by text and via the telehealth system. If after two weeks they do not enter data they will be contacted by the PI and offered more support and training. Compliance data will be analysed as part of the process evaluation.

Compliance with the patient reported outcome measures would be monitored by the CRF nurse. She will contact the participants to support data collection, identify and chase missing data and feedback to the PI. In the event of missing data the CRF nurse will telephone the patients/carer after two weeks. She will contact them a minimum of twice and a maximum of three times to chase the data. A contact log will be kept.

**6g. Sample size**

The study aims to recruit a total of 40 patients and their carers. 20 patients and their primary carer will be randomised to the intervention arm (a minimum of 6 months use of the TiM telehealth plus usual care) and 20 patents and their carer in the control arm (usual care).

Since the proposed trial is primarily an assessment of the acceptability of the intervention and the feasibility of a full trial, the proposed sample size is not based on standard statistical parameters such as a clinically relevant difference between groups. Instead, the sample size is justified on the grounds of quantifying patient variance (i.e. the standard deviation) in the proposed outcome measures (in particular quality of life measures) and on feasibility of the full trial, as follows:

- A sample size of 40 patients allows a standard deviation to be estimated to within a precision of ±20% of its true underlying value with 90% confidence. This estimate will be synthesised with standard deviations observed in other published studies (e.g. (41, 44-47) and on-going trials within SITraN (48, 49), to provide a robust estimate for use in the sample size calculation for the full trial.

- Given the rarity of MND, any definitive study will be infeasible if the required sample size is substantial. Assuming the upper limit for feasible UK study is around 200-300 patients in total, it follows that the full study would need powering to detect a standardised effect size of at least 0.4 SDs. This pilot trial will provide a preliminary assessment of whether the intervention might feasibly achieve this, and inform the choice of outcome measures for the proposed full study.

This sample size is also in keeping with the proposal of 12 evaluable patients per arm in a pilot study (after withdrawal or drop-out) (50).

**6h. Blinding**

The PI will not be blinded to the randomisation as they are responsible for training the participants to use the TiM system and any on-going technical or training requirements. The treating clinicians will not be blinded to the arm of the intervention as they are responsible for the clinical care of the patient and reviewing the data and the Shadow Monitoring System.



For peer review only - http://bmjopen.bmj.com/site/about/guidelines.xhtml

The PI will enter screening baseline data. Participant reported outcome measures after this will be collected by an independent research nurse from the Sheffield Teaching Hospitals Clinical Research Facility who will facilitate collection of these surveys by post or, if preferred by the patient, in person. They will enter the details into the study database.

Following the end of the trial and database lockdown the PI will analyse the two groups of data whilst remaining blinded to the allocation of the two groups. The STH CRF will hold the database code to identify the allocated groups.

The PI will conduct the qualitative interviews and collect the system use data and will not be blinded to these measures.

## 7. Study treatment

### Standard clinical care – Intervention arm and Standard care arm

Usual clinical care will continue throughout for participants in both arms of the study. All participants will continue to be invited to the Sheffield MND Care Centre Clinic for routine review. They will be seen by their consultant neurologist and the MND multidisciplinary team, according to their routine two- to three-monthly schedule. All patients will have access to the MND telephone helpline provided by the Sheffield MND care team.

### The TiM system - Intervention arm

Those in the intervention arm will use the TiM system, in addition to standard clinical care. All necessary hardware, software, data transfer and support costs for the TiM system will be met by Abbott Healthcare Products Ltd in collaboration with Carematix.

Patients will be provided with a TiM patient hub: a handheld, touch screen Samsung Galaxy tablet computer that communicates with the MND specialist nurse's TiM clinician system at the Sheffield MND Care Centre. Patients will be asked to use the system at least weekly. Each week the telehealth hub asks the patient a series of questions to detect common problems found in MND, such as worsening mobility, or swallow, symptoms of depression, anxiety, pain, saliva and spasms. Some of the questions closely match validated scoring scales (e.g. the ALS Revised Functional Rating Score (51) and the depression and anxiety short screen: PHQ-4) but others have been specifically designed by the clinical team to be used in the telehealth system. The TiM system also enquires about symptoms of respiratory insufficiency and infection, nutrition and social care. Patients using specialist equipment, such as non-invasive ventilation or gastrostomy tubes will additionally be asked to report problems related to the intervention. The TiM system can also weigh patients weekly and monitor patients' overnight oximetry using established telehealth monitors.



For peer review only - http://bmjopen.bmj.com/site/about/guidelines.xhtml

Carers will also be asked to complete a weekly telehealth session in order to monitor their well being. The TiM system includes a carer strain screen and the PHQ-4 depression and anxiety screen. There is also the opportunity with the telehealth system for patients or carers to trigger an adhoc session if issues arise during the week about which they wish to inform the centre. Interspersed through the questions are educational messages and users have access to a bank of educational resources within the hub.

The patient and carer responses are transmitted (via an encrypted 3G mobile signal) to the Carematix server. The responses undergo immediate computational analysis, using pre-determined clinical algorithms, which assigns an alert level to each response. This limits the amount of nurse's time required to use the system. An automated acknowledgement is sent back to the user indicating whether to expect contact from the MND centre based on the results and the timescale for the response.

Each day the MND nurse will log into the TiM system and will be presented with the responses from all patients using the TiM system. They will be automatically alerted to any important changes. Urgent alerts include any new and severe symptom or any new problem that poses a major risk to the patient or carer (e.g. choking, falling, respiratory insufficiency). Routine alerts include any other deterioration in the patient's ALSFRS-R or any new symptom. An appropriately timely response will be made to each alert level. Patients will be reminded to seek urgent medical attention in an emergency.

The information on patient status may facilitate rescheduling of appointments according to patient need rather than the fixed intervals used at present (e.g. the appointment could be delayed if the patient is well, and the TiM system has activated no new alerts). As the feasibility and safety of the TiM system has not been previously evaluated, during the TiM trial patients will continue to attend routine clinic appointments and no patient will have their clinic delayed. The feasibility and safety of rescheduling appointments will be examined using a shadow monitoring protocol (detailed later).

Patients and carers will undergo a training session and a follow-up telephone call after two weeks. The TiM system has been designed to be user friendly and to encourage compliance with the weekly sessions. Face-to-face training with the hub system will be offered at the start of the intervention. Support will be available throughout the trial in the hub. Compliance will be monitored and should patients not complete the TiM system for three weeks in a row, contact will be made to offer more training or support.

The TiM system has been designed by the applicant in collaboration with her supervisors, the Sheffield MND team, Abbott Healthcare Products Ltd. and Carematix. Carematix have experience in delivering similar home telemonitoring systems in other diseases. In developing the system, expertise has also been sought from those developing telehealth services in other diseases in the University of Sheffield and NIHR CLARHC for South Yorkshire. These included the School of Health and Related Research (ScHARR) SMART consortium (Self Management supported by Assistive, Rehabilitation and Telecare technologies), NIHR CLAHRC SY Telehealth & Care Technologies (TaCT) for Long Term Conditions theme, and Devices for Dignity (52).


For peer review only - http://bmjopen.bmj.com/site/about/guidelines.xhtml

For peer review only



For peer review only - http://bmjopen.bmj.com/site/about/guidelines.xhtml

## 8. Data collection

### 8a. Quantitative data collection

Data collection will occur at baseline, three months and six months at the end of the study. Participant data will be completed using postal and telephone questionnaires to minimize patient burden and cost. The study will continue for a minimum of six months. Follow-up will continue until the last participant has used the system for six months. The maximum proposed follow-up will be 18 months.

**Patient measures**

Baseline measures:

- Age
- Gender
- Experience with technology (frequency of use of a computer, tablet or smart phone)
- Major health condition that could impact on the use of telehealth (including mood disorder, other symptomatic chronic disease)
- Medication

Outcome measures will be collected at 0, 3 and 6 months, then every six months until the end of the study and finally at the end of the study:

- Quality of life measures
  - ALSAQ-40 (an MND disease specific quality of life score (41))
  - SF-36-RAND
  - EQ-5D+D (EQ-5Q-3L with a dignity bolt-on)
- Clinical outcomes
  - ALSFRS-R (an MND disease specific functional rating score (51)
  - Pain score (modified Likert scale)
  - CSS-MND Saliva Severity Scale (designed for use with MND patients) plus global change scale
  - Hospital Anxiety and Depression Scale
- Health resource usage questionnaire
- Patient experience questionnaire



For peer review only - http://bmjopen.bmj.com/site/about/guidelines.xhtml

**Carer measures**

Baseline measures:

- Age
- Gender
- Frequency of use of a computer, tablet or smart phone
- Major health that could impact on the use of telehealth
- Relationship to patient
- Number of hours spent per week providing care for patient

Outcome measures will be collected at 0, 3 and 6 months and at the end of the study:

- SF-36 RAND
- 12 item Zarit Burden Inventory (53)
- Hospital Anxiety and Depression Scale (54)
- Carer satisfaction questionnaire

Data will be collected to evaluate the conduct of the trial including:

- Participant compliance with the weekly telehealth session
- Rate of completion of outcome measures
- Rates of recruitment and withdrawal
- Participant actual and perceived time burden associated with the system
- Time spent by the MND nurse using TiM system and responding to alerts or queries generated by the system.



For peer review only - http://bmjopen.bmj.com/site/about/guidelines.xhtml

## 8b. Qualitative sub-study

### Intervention patient and carer interviews

Qualitative semi-structured interviews will be conducted with patients and carers in the intervention arm. Participants randomized into the intervention arm will be invited to take part in interviews. Baseline interviews will occur at one month after the intervention is started. A further interview will be conducted at 6 months. Six months is considered an appropriate timeframe for patients to become familiar with the intervention and its impact on quality of life.

Interviews will be conducted until data saturation is reached. The interviews will draw directly upon peoples' own experience and views, within the context of everyday lives to explore topics including

- Participants experience and expectations of technology
- Participants' expectations of telehealth services
- Barriers and aids to recruitment
- Compliance with the TiM system
- How the TiM system is used at home by patients and their carers
- The impact of using the TiM system on their lives and well-being
- The impact of education on their day-to-day lives
- The experiences of carers monitoring
- Whether the outcome measures used capture the changes in participants well being associated with using the TiM system.
- How the system would be used outside a trial

The early phase interview will explore participants' expectations of technology and the TiM system, the views on the system, their experiences of training and using the equipment. The later phase will explore further how the TiM system influenced their care and quality of life, mental well being as their condition changed. It will also identify barriers and facilitators to adoption of the TiM system.

The applicant will agree pre-defined topic schedules (see Appendix B) developed from the literature, expert consensus and discussion with the trial management group with supervision from Dr. Wendy Baird, an experienced qualitative researcher (School of Health and Related Research, Sheffield University). The PI will conduct interviews in the participants' home. The PI will conduct qualitative interviews until data saturation is reached (55). Interviews will be audio- recorded, transcribed verbatim and analysed with coding and retrieval of data supported by NVivo software.

Due to the nature of MND consideration will be given to participants' needs. The research team has experience in conducting qualitative interviews with patients and carers and these interviews will be conducted in a similar fashion. Often patients with MND prefer to be interviewed with their carer. This also aids communication where patients have speech difficulties and allows participants to support each other whilst discussing sensitive issues. Patients can use communication devices and all participants will be provided with a brief topic guide prior to the interview to facilitate participation for those with communication difficulties. Interviews will be limited to approximately one hour to reduce burden and fatigue. If participants prefer to be interviewed together carers will also be offered separate interviews where possible.



For peer review only - http://bmjopen.bmj.com/site/about/guidelines.xhtml

Field notes will also be collected by the PI during the face-to-face training using the TiM system to determine participants' early reactions to using the system and their needs for training.

**Control group interviews**

Following randomization, those patients and carers who are assigned the control arm will complete the baseline questionnaires. They will then have a short (15-20 minute) semi-structured interview with the PI. This will focus on their experiences and opinions of the baseline questionnaires. It will examine whether they were easy or difficult to complete, whether they were acceptable or caused distress to complete and which questions most reflected their condition and current quality of life.

The interviews, topic guides and analysis will be conducted in the same way as described in the previous section. Topic guides will not be provided before the interview but participants invited to submit any further comments to the research team either in writing or telephone following the interview. It is expected that patients and carers will be interviewed together. Interviews will continue until data saturation is reached or a maximum of 10 interviews conducted.

**Staff interviews**

At least five staff that care for the participants will undergo one-to-one semi structured interview by the PI during and at the end of the intervention. This will include the two responsible consultant clinicians (Dr Chris McDermott and Professor Pamela Shaw), at least one MND specialist nurse who has used the telehealth system and two members of the MND community team who have cared for participants. They will allow them to draw on their experiences of the TiM system in more depth. A staff information leaflet will be provided and written consent will be required prior to any interview.

Topics will include

- The day-to-day use of the TiM system
- The impact of the TiM system on clinical care of patients and carers
- The safety and accuracy of the system
- Barriers and aids to adoption of the TiM system.
- Views on amending the appointment schedule

These will be planned and conducted in the same manner as the participant interviews under the supervision of Dr Wendy Baird. An interview with the MND nurse and clinicians using the system will be scheduled early in the trial to capture any problems with training and set up of the system. At the end of the trial further interviews will be held with the MND team as described above.

Following the interviews a focus group with the clinical team will be held to draw together all the information gathered from the patient and staff interviews. It will be chaired by Dr Wendy Baird, independent qualitative researcher, transcribed and analysed by the PI under her supervision.


For peer review only - http://bmjopen.bmj.com/site/about/guidelines.xhtml

The qualitative findings will facilitate the exploration of any issues and challenges, which may arise from using TiM from the perspective of all stakeholder groups. The findings will enhance understanding of the feasibility of using TiM and assist with the interpretation of the clinical data from the perspective of patients and clinicians.

**8c. Shadow monitoring protocol**

In order to determine the safety of a remote monitoring system that may enable clinicians to make decisions regarding a patient's management the trial will also collect data on clinicians' opinion on the accuracy of the data displayed by the TiM system. This is referred to as the shadow monitoring protocol.

Prior to each patient's face-to-face visit (depending on their appointment schedule) the treating MND doctor will be asked to conduct a remote assessment of the patient by reviewing the TiM system clinical information. They will be asked to indicate, given the information provided by the TiM system, whether they would change their patient's appointment. The patient would attend the appointment as scheduled and after the appointment the clinician would be asked whether the appointment schedule time was correct. They would also be asked to indicate whether they felt that the information displayed on the TiM system was a safe and accurate reflection of the patient's condition and whether it influenced their clinic visit. Clinicians will also indicate whether the TiM system had affected the consultation. They will also report any adverse events identified. Should patients be unable to travel to clinic they will be offered a telephone consultation at the usual scheduled time. The same Shadow Monitoring questions and need to report adverse events will apply.

The results of this shadow monitoring will be triangulated with the qualitative sub-study and will influence the later interview topic guide.

**8d Process evaluation**

Data regarding the TiM system use by patients, carers and staff will be will be collected in order to understand how the system could be used in the NHS MND care process. It will also collect data regarding the extra time and resources required to manage the problems generated by the TiM system. It will be triangulated with data gained from the qualitative sub-study, adverse event log and shadow monitoring protocol.


For peer review only - http://bmjopen.bmj.com/site/about/guidelines.xhtml

## 9. Analysis

The PI will conduct analysis with regular supervision from the TMG.

### 9a. Feasibility and quantitative analysis

The feasibility of a full trial will be determined by analysis of

- Recruitment rates
- Retention rates
- Compliance rates
- Sample size calculations as detailed above

The safety, acceptability and feasibility of use of the TiM system

- Incidence of adverse events (clinical and related to the TiM system functionality)
- Information collected using the Shadow Monitoring process
- Qualitative data analysis

The PI and the CRF study nurse will be responsible for chasing missing data. The CRF nurse is responsible for chasing the questionnaire data and will telephone the patients a minimum of once and maximum of twice to chase unreturned questionnaires or clarify missing data within the questionnaire packs. They will report monthly to the PI. For the main outcome measures, (SF-36 and ALSAQ-40) protocols are provided for managing missing data if necessary. Participants who withdraw will be encouraged to continue to be followed up and reasons for withdrawal ascertained where possible. In the proposed larger, efficacy trial intention to treat analysis will be adopted.

Quantitative analysis will be undertaken in a similar manner for all endpoints. The change from baseline at each time point will be analysed using analysis of covariance in which the covariates are treatment group and the baseline value. For instance, the change in ALSFRS-r at six months will be analysed with treatment group and baseline ALSFRS-r as covariates. The mean (standard deviation) change in each group, the difference between groups and its associated 95% confidence interval will be reported. No formal hypothesis testing will be undertaken for this pilot study.

For peer review only



For peer review only - http://bmjopen.bmj.com/site/about/guidelines.xhtml

**9b. Qualitative analysis**

Data from the interviews will be recorded, transcribed and undergo Framework analysis (56). Although Framework analysis was developed for applied policy it has proved useful in applied health research. Analysis will be ongoing and iterative involving concurrent data collection and analysis, with systematics efforts to check and refine developing categories of data. Themes and hypothesis identified in the early phases of data collection will inform the areas of investigation in later interviews. Regular meetings with supervisors will review the data analysis, explore respondents' underlying reasoning, discuss deviant cases and reach agreement on recurrent themes and findings. The PI's field notes and reflexive diary will also be reviewed and used to inform the analysis of qualitative data. Dr Wendy Baird, an independent, experienced qualitative research, will supervise this stage of the work.

Results from the qualitative analysis will be triangulated, for example, to explore the reasons why problems with the trial methodology or TiM system have occurred. Both themes and anonymous verbatim comments will be published to demonstrate the findings.

For peer review only



For peer review only - http://bmjopen.bmj.com/site/about/guidelines.xhtml

**10. Data entry, security and confidentiality**

Clinical quantitative data input will be the responsibility of the PI (baseline) and CRF study nurses (months 3 and 6, and at the end of the study). Data quality will be the responsibility of CRF nurses and PI who will report back to the TMC and TSC. The qualitative data and system usage data will be the responsibility of the PI. Data (including audio-recordings) will be collected and retained in accordance with the Data Protection Act 1998 and Caldicott Principles. Anonymised study data will be entered onto a validated database system designed to an agreed specification between the PI and Sheffield CTRU and securely stored on the SU intranet. The PI and the CRF research nurses will have access to data on the database through the use of usernames and encrypted passwords. Study documents will be retained in a secure location during and after the study has finished.

All source documents will be retained for a period of at least 5 years following the end of the study, as per the CTRU SOP. Where study related information is documented in medical records those records will be retained for at least 5 years after the last patient last visit

The data provided through the TiM system will be collected using a secure web-app accessed by the participants by a unique username and password. It will be stored on a secure server that will be available through a web-portal hosted by Carematix to the clinical team using secure usernames and password.

For the purposes of the trial each participant will be given a unique TiM system code. This will allow all data to be relayed through the web-app without any associated patient identifiable features. This code will be held separately and stored securely on the STH intranet to allow individual identification by the MND care team. The clinician will display only the anonymous code. This will be accessed through a secure portal with usernames and passwords. No identifiable information will be stored on the patient hub or on the TiM server. The technology providers will have no access to patient identifiable information. Any technology problems will be dealt with by the research team and participants will have no contact with the technology providers.

The system has a full electronic audit trail and will be regularly backed up and will be held in a way that conforms to STH information governance procedures.

**Access to source data**

Monitoring and audit by the relevant health authorities will be permitted by the sponsor. These include the Research Ethics Committee and local R&D departments. The sponsor will be allowed to monitor and audit the study at each site and be allowed access to source data and documents for these purposes.

For peer review only

## 11. Safety and safety assessments

We do not envisage any serious safety or adverse events associated with the intervention. The system does not give individual advice to a patient or recommend change in management without input from a clinician. The trial protocol requires patients to continue with their usual care including planned outpatient appointments and the Shadow Monitoring Protocol will evaluate whether the data provided by the TiM system is felt to accurately reflect the patients' clinical condition. The responsible clinician who is a consultant neurologist with specialist experience in MND and research will continue to review the patient on a regular basis (unless the patient is unable to attend clinic) and will have overall responsibility for their care throughout the trial. The specialist MND nurses using the TiM system have extensive experience in managing patients via the existing MND helpline.

The database will automatically alert the trial manager to any carer scoring 11 or more of the Hospital Anxiety and Depression score collected as part of the outcome measures. This will allow the trial manger to identify those carers who may require further support.

**Adverse Event Reporting**
All adverse events will be reported in accordance with the Sheffield CTRU Adverse Event and Serious Adverse Events SOP.

Participants will be monitored for adverse clinical events and efforts will be made to ascertain whether the TiM system influenced the event or could have predicted the event. These include unplanned admissions and deaths. Non-clinical events relating to the use of the telehealth hub will recorded e.g. failure to record or deliver information to and from the clinical interface.

In research other than CTIMPs an adverse event is defined as: is any unfavorable and unintended sign (including an abnormal laboratory finding), symptom, or disease having been absent at baseline, or, if present at baseline, appears to worsen AND is temporally associated with medical treatment or procedure, **REGARDLESS** of the attribution (i.e., relationship of event to medical treatment or procedure).

**Serious Adverse Event (SAE)**
In research other than CTIMPs, the National Research Ethics Service defines a Serious Adverse Event (SAE) is defined as an untoward occurrence that:
(a) results in death;
(b) is life-threatening*;
(c) requires hospitalization** or prolongation of existing hospitalization**;
(d) results in persistent or significant disability or incapacity;
(e) consists of a congenital anomaly or birth defect; or
(f) is otherwise considered medically significant by the investigator.

*"life-threatening" in the definition of "serious" refers to an event in which the patient was at risk of death at the time of the event; it does not refer to an event which hypothetically might have caused death if it were more severe. **Hospitalisation is defined as an inpatient admission, regardless of length of stay, even if the


For peer review only - http://bmjopen.bmj.com/site/about/guidelines.xhtml

hospitalisation is a precautionary measure for continued observation. Hospitalisations for a pre-existing condition, including elective procedures that have not worsened, do not constitute an SAE.

**Adverse event exclusions**
The only adverse event that will be excluded is:
1. Standard or expected disease progression.

**Adverse event inclusions**

All serious adverse events will be reported. These include deaths of participants and emergency admissions. We will attempt to determine whether the use of the TiM system contributed to the event, in particular whether there was any delay in seeking help due to the use of the system.

**Assessment of Adverse Events**
The following criteria will be used when assessing adverse events: Intensity (severity):
Mild - does not interfere with routine activities
Moderate - interferes with routine activities
Severe - impossible to perform routine activities

Relationship to the study treatment:
Unrelated - There is no evidence of any causal relationship. N.B. An alternative cause for the AE should be given
Unlikely - There is little evidence to suggest there is a causal relationship. There is another reasonable explanation for the event (e.g. the participant's clinical condition, other concomitant treatment).
Possible - There is some evidence to suggest a causal relationship. However, the influence of other factors may have contributed to the event (e.g. the participant's clinical condition, other concomitant treatments).
Probable - There is evidence to suggest a causal relationship and the influence of other factors is unlikely.
Definite - There is clear evidence to suggest a causal relationship and other possible contributing factors can be ruled out.
Not assessable - There is insufficient or contradictory information which cannot be supplemented or verified

**Reporting procedures**

All study participants will be encouraged to contact and inform their site research team if they experience any new medical problem or are admitted to hospital. Those that are not picked up through general contact will be identified at their routine 2-3 monthly outpatient appointments either in person or by telephone as part of the Shadow Monitoring Protocol. The patients' consultant neurologist (Dr. Chris McDermott or Professor Dame Pamela Shaw) will enquire about any adverse events since the previous visit and record these on the adverse event paper CRF and database. For any Serious Adverse Events an SAE paper CRF and database entry will be completed. The PI and consultant neurologist will assess the event and the CRF will be kept in the site file. Serious adverse events will be reported to the TSC, TMG and the sponsor if deemed by


For peer review only - http://bmjopen.bmj.com/site/about/guidelines.xhtml

either to be related to the trial. Reports of related and unexpected SAEs will be submitted to the ethics committee within 15 days of the chief investigator becoming aware of the event. This will use the National Research Ethics Service Report of Serious Adverse Event form. Information will also be included in the routine progress reports to the sponsor and ethics committee. Routine safety data and all SAEs that the TMG or TSC deems to be related to the trial will also be reported to the technology provider in the same manner.

Any suspected adverse drug reaction would be assessed and reported to the MHRA as part of clinicians' routine pharmacovigilance responsibilities using the Yellow Card Scheme. The technology provider, Abbott Healthcare Products Ltd. manufactures a number of drugs (listed in Appendix A). Any suspected adverse drug reaction involving an Abbott Healthcare Products Ltd. drug would also be reported to the manufacturer in the same manner, within 24 hours of receipt by the CI or the next working day for reports received out of hours. Such reports should be sent to ukpharmacovigilance@abbott.com. On a monthly basis the PI will send a reconciliation list of all the reports sent to the Abbott Pharmacovigilance Department within that month to ensure that all the appropriate information has been exchanged. Should any discrepancies arise, both parties will immediately seek to resolve them.

For peer review only

## 12. Ethical considerations

The study will be conducted in accordance to Good Clinical Practice Guidelines and subject to Research Ethics Committee favourable opinion. The study received a favorable approval from an independent panel representing the NIHR, which funds Dr. Esther Hobson's NIHR Doctoral Fellowship Award.

The study has approval from the Sheffield Teaching Hospitals NHS Foundation Trust's Research and Development department. It has also received favourable review from Dr. Mike Bradburn, study statistician at ScHARR, Dr. Cindy Cooper, director of CTRU and ScHARR, and Dr. Wendy Baird of the Yorkshire and Humber Research and Design service, Professor Alicia O'Cathain, Professor of Health Services Research, ScHARR and Professor Dame Pamela Shaw, SITraN. The application will be submitted through the IRAS central allocation system. The approval letter from the ethics committee and copy of approved patient information leaflet, consent forms, CRF's and questionnaires will be present in the site files before initiation of the study and patient recruitment.

It is recognized that patients with MND may be frail and nearing at the end stages of their lives. The research team has extensive experience in conducting clinical trials in this population. The study design has attempted to limit the burden imposed by the study by avoiding unnecessary study visits (by combining them with scheduled visits), collecting data in the participants' homes at their convenience and limiting the study procedures to the minimum necessary. The intervention has been designed in collaboration with patients and carers to maximize ease of use and minimize impact on participants' lives. It is appreciated that there are a number of questionnaires that require completion. Given one aim of the study is to determine the most appropriate outcome measures to evaluate efficacy of the TiM system there are more questions than would be used in a large scale trial. These have been reviewed by the Sheffield MND Research Advisory Group (the local PPI group) and the lay members of the TSC (David Stelmach) and TMG (Anne Quinn) to ensure acceptability. Participants will be supported by the CRF nurse to complete these at their convenience in a manner selected by the participant (either by post, telephone or in person).

There are other clinical studies ongoing in the Sheffield MND care centre. Involvement in other studies would not preclude patients from entering this study. Consideration of the burden involved in the study, potential impact on the outcome of the study and the patients' expressed priorities will be considered before patients are approached to be involved. If involvement in this study excludes patients from entering another clinical trial patients will be given the option to withdraw from this study.

The potential conflict of interest between the role of the clinical team in caring for patients and their role as researchers is recognized. The study design has considered the impact of this conflict on the participants choices and also any potential bias. Whilst PI is a doctor working within the MND team she is a specialty training registrar and overall responsibility for the patients' clinical care will remain with the consultant neurologist rather than the PI. Whilst she may have already cared for potential participants, following an invite to participate in the trial will no longer see these patients in their routine clinical appointments and her role will be as a researcher.


For peer review only - http://bmjopen.bmj.com/site/about/guidelines.xhtml

The dual role of the PI as a doctor and researcher has been previously evaluated. The professional background of a doctor may actually aid the building of a research relationship, allow patients to be more open and comfortable with discussing their health with someone who they already trust (56). In order successfully identify any potential bias the purpose of the research and nature of the PI's role will be emphasized throughout the study, the PI will keep a reflexive diary and field notes and identify any potential bias. Where bias is most likely, i.e. in the collection of outcome measures steps have been taken to limit this: the quantitative outcome measures will be collected by an independent study nurse and the qualitative interview structure and topic guides have been planned with supervision from an independent researcher Dr. Wendy Baird. The PI will be supervised, as part of her PhD by independent academics: Dr. Cindy Cooper and Dr. Mike Bradburn (focusing mainly on the trial methodology and conduct, and quantative data analysis), Dr. Wendy Baird and Professor Sue Mawson (qualitative work and service evaluation). If, during the research, participants identify any medical problems, the PI has a duty of care and will make arrangements to deal with these problems. This might involve signposting them to appropriate services or liaising with the clinical team. A log of these activities will be kept and reviewed by the TMG.

When the participants have prior knowledge of the researcher they may feel a sense of duty and feel pressurized to participate (57). Ground rules, informed consent, confidentiality, freedom to stop and what to expect will be discussed with all participants. Participants will be approached by letter and they will be required to contact the study team allowing them to consider the trial in detail first. It will be explained to the patient (both verbally and in the information leaflets and consent forms) that participation is voluntary and will not affect their ongoing care. The information leaflet differentiates the research process and their usual care. It will be made clear, particularly in the interview phase that the PI's role is as a researcher and the aim of the study is to critically analyse service provision and that whilst comments, particular negative comments, will be passed back to the care team they will treated with confidence and respect.

Whilst the carer participant is not a patient of the Sheffield MND team the research team have a duty of care to the carer. There may be circumstances where the carer may disclose information that requires medical care, for example disclosing symptoms of depression or anxiety. At the start of the trial the carer participants' GP will be informed of the trial. In the event of a serious risk being identified the research team will discuss this in confidence with the carer and make arrangements to resolve the problem. This might include referral to his or her own GP or other health professional. Carer participants will be informed of these procedures in the Carer information leaflet and consent form. Confidentiality will be maintained in accordance with the General Medical Council's guidance on Confidentiality (58).

Upon publication of the qualitative interviews it may be possible to identify participants' comments although this will be avoided if possible. This is explained to participants in the Interview Information Leaflets and on the consent form.


For peer review only - http://bmjopen.bmj.com/site/about/guidelines.xhtml

## 13. Finance and indemnity

The trial has been financed through an NIHR doctoral fellowship grant and details have been drawn up in a separate agreement.

This is an NHS sponsored study. If there is negligent harm during the clinical trial when the NHS body owes a duty of care to the person harmed, NHS indemnity will cover NHS staff, medical academic staff with honorary contracts and those conducting the trial.

The University of Sheffield has in place insurance against liabilities for which it may be legally liable and this cover includes any such liabilities arising out of this clinical trial.

## 14. Reporting and dissemination

Results of the study will be disseminated in peer reviewed scientific journals and clinical and academic conferences. Details of the study will also be made available on the SITraN and ScHARR websites, blogs and social media and through local MND groups. Summaries of the research will be updated periodically on the SITraN website to inform readers of the ongoing progress. Following publication contact with other UK MND care centres will be made to disseminate the findings and assess buy-in potential for a full study if this is appropriate.

For peer review only



For peer review only - http://bmjopen.bmj.com/site/about/guidelines.xhtml

## 15. References

1. The neurological alliance. Neuro numbers. A brief review of the numbers of people in the UK with a neurological condition.
2. Whitehead B, O'Brien MR, Jack BA, Mitchell D. Experiences of dying, death and bereavement in motor neurone disease: A qualitative study. Palliat Med. 2012;26(4):368-78.
3. O'Brien MR, Whitehead B, Jack BA, Mitchell D. The need for support services for family carers of people with motor neurone disease (MND): views of curent and former family caregivers a qualitative sutdy. Disabil Rehabil. 2012;34(3).
4. Miller RG, Mitchell JD, Moore DH. Riluzole for amyotrophic lateral sclerosis (ALS)/motor neuron disease (MND). Cochrane Database Syst Rev. 2012;3:CD001447.
5. Bourke SC, Tomlinson M, Williams TL, Bullock RE, Shaw PJ, Gibson GJ. Effects of non-invasive ventilation on survival and quality of life in patients with amyotrophic lateral sclerosis: a randomised controlled trial. The Lancet Neurology. 2006;5(2):140-7.
6. Aridegbe T, Kandler R, Walters SJ, Walsh T, Shaw PJ, McDermott CJ. The natural history of motor neuron disease: Assessing the impact of specialist care. Amyotroph Lateral Scler. 2012.
7. Chio A, Buffa C, Mutani R, Bora G, and the PARALS. Positive effects of tertiary centres for amyotrophic lateral sclerosis on outcome and use of hospital facilities. J Neurol Neurosurg Psychiatry. 2006;77(8):948-50.
8. Johnston B, Kidd L, Wengstrom Y, Kearney N. An evaluation of the use of Telehealth within palliative care settings across Scotland. Palliat Med. 2012;26(2):152-61.
9. Foley G, Timonen V, Hardiman O. Experience of Services as a Key Outcome in Amyotrophic Lateral Sclerosis (ALS) Care: The Case for a Better Understanding of Patient Experiences. Am J Hosp Palliat Care. 2012;29(5):362-7.
10. Motor Neurone Disease Association UK. MND statistics.
11. Baxter SK, Baird WO, Thompson S, Bianchi SM, Walters SJ, Lee E, et al. The initiation of non-invasive ventilation for patients with motor neuron disease: Patient and carer perceptions of obstacles and outcomes. Amyotroph Lateral Scler. 2012.
12. Foley G, Timonen V, Hardiman O. Patients' perceptions of services and preferences for care in amyotrophic lateral sclerosis: a review. Amyotroph Lateral Scler. 2012;13(1):11-24.
13. The use of non-invasive ventilation at end of life in patients with motor neurone disease. Submitted for publication. 2012.
14. Hecht MJ, Graesel E, Tigges S, Hillemacher T, Winterholler M, Hilz MJ, et al. Burden of care in amyotrophic lateral sclerosis. Palliat Med. 2003;17(4):327-33.
15. Peters M, Fitzpatrick R, Doll H, Playford ED, Jenkinson C. The impact of perceived lack of support provided by health and social care services to caregivers of people with motor neuron disease. Amyotroph Lateral Scler. 2012;13(2):223-8.
16. van Teijlingen ER, Friend E, Kamal AD. Service use and needs of people with motor neurone disease and their carers in Scotland. Health Soc Care Community. 2001;9(6):397-403.
17. McLean S, Chandler D, Nurmatov U, Liu J, Pagliari C, Car J, et al. Telehealthcare for asthma: a Cochrane review. CMAJ : Canadian Medical Association journal. 2011;183(11):E733-42.



For peer review only - http://bmjopen.bmj.com/site/about/guidelines.xhtml

18. McLean S, Nurmatov U, Liu JL, Pagliari C, Car J, Sheikh A. Telehealthcare for chronic obstructive pulmonary disease. Cochrane Database of Systematic Reviews (Online). 2011(7):CD007718.
19. Chua R, Craig J, Wootton R, Patterson V. Randomised controlled trial of telemedicine for new neurological outpatient referrals. 2001;71(1):63-6.
20. Steventon A, Bardsley M, Billings J, Dixon J, Doll H, Hirani S, et al. Effect of telehealth on use of secondary care and mortality: findings from the Whole System Demonstrator cluster randomised trial. Br Med J. 2012;344.
21. Corben S, Rosen R. Self-management for Long-term Conditions. Patients' perpective on the ways ahead. In: Conditions TKsFrML-t, editor. 2005.
22. 3millionlives Campaign. Available from: http://3millionlives.co.uk.
23. Vitacca M, Bazza A, Bianchi L, Gilè S, Assoni G, Porta R, et al. Tele-assistance in chronic respiratory failure: patients characterization and staff workload of 5-year activity. Telemedicine Journal and e-health : the official journal of the American Telemedicine Association. 2010;16(3):299-305.
24. Vitacca M, Comini L, Tentorio M, Assoni G, Trainini D, Fiorenza D, et al. A pilot trial of telemedicine-assisted, integrated care for patients with advanced amyotrophic lateral sclerosis and their caregivers. J Telemed Telecare. 2010;16(2):83-8.
25. Vitacca M, Bianchi L, Guerra A, Fracchia C, Spanevello A, Balbi B, et al. Tele-assistance in chronic respiratory failure patients: a randomised clinical trial. The European Respiratory Journal. 2009;33(2):411-8.
26. Vitacca M, Comini L, Assoni G, Fiorenza D, Gilè S, Bernocchi P, et al. Tele-assistance in patients with amyotrophic lateral sclerosis: long term activity and costs. Disability and rehabilitation Assistive technology. 2012.
27. Pinto A, Almeida JP, Pinto S, Pereira J, Oliveira AG, de Carvalho M. Home telemonitoring of non-invasive ventilation decreases healthcare utilisation in a prospective controlled trial of patients with amyotrophic lateral sclerosis. J Neurol Neurosurg Psychiatry. 2010;81(11):1238-42.
28. de Almeida JPL, Pinto AC, Pinto S, Ohana B, de Carvalho M. Economic cost of home-telemonitoring care for BiPAP-assisted ALS individuals. Amyotrophic lateral sclerosis : official publication of the World Federation of Neurology Research Group on Motor Neuron Diseases. 2012;13:533-7.
29. Sweeney V. Pilot scheme allowed Alan to be nursed at home. The Inverness Courier. 2009 24 Feb, 2009.
30. Nijeweme-d'Hollosy WO, Janssen EPF, Huis in 't Veld RMHA, Spoelstra J, Vollenbroek-Hutten MMR, Hermens HJ. Tele-treatment of patients with amyotrophic lateral sclerosis (ALS). J Telemed Telecare. 2006;12 Suppl 1:31-4.
31. Montes J, Levy G, Albert S, Kaufmann P, Buchsbaum R, Gordon PH, et al. Development and evaluation of a self-administered version of the ALSFRS-R. Neurology. 2006;67(7):1294-6.
32. Maier A, Holm T, Wicks P, Steinfurth L, Linke P, Munch C, et al. Online assessment of ALS functional rating scale compares well to in-clinic evaluation: a prospective trial. Amyotroph Lateral Scler. 2012;13(2):210-6.
33. Rafiq MK, Proctor AR, McDermott CJ, Shaw PJ. Respiratory management of motor neurone disease: a review of current practice and new developments. Pract Neurol. 2012;12(3):166-76.
34. Department of Health U. Supporting People with Long Term Conditions: An NHS and Social Care Model to supprt local innovation and integration. 2005.

For peer review only



For peer review only - http://bmjopen.bmj.com/site/about/guidelines.xhtml

35. McCall K, Keen J, Farrer K, Maguire R, McCann L, Johnston B, et al. Perceptions of the use of a remote monitoring system in patients receiving palliative care at home. Int J Palliat Nurs. 2008;14(9):426-31.
36. McCann L, Maguire R, Miller M, Kearney N. Patients's perceptions and experiences of using a mobile phone-based advanced symptom management system (ASyMS ©) to monitor and manage chemotherapy related toxicity. Eur J Cancer Care (Engl). 2009;18(2):156-64.
37. Kofoed S, Breen J, Gough K, Aranda S. Benefits of remote real-time side-effect monitoring systems for patients receiving cancer treatment. Oncology Reviews. 2012;6(7):1-13.
38. Bower P, Kennedy A, Reeves D, Rogers A, Blakeman T, Chew-Graham C, et al. A cluster randomised controlled trial of the clinical and cost-effectiveness of a 'whole systems' model of self-management support for the management of long- term conditions in primary care: trial protocol. Implement Sci. 2012;7:7.
39. Palmer R, Enderby P, Cooper C, Latimer N, Julious S, Paterson G, et al. Computer therapy compared with usual care for people with long-standing aphasia poststroke: a pilot randomized controlled trial. Stroke. 2012;43(7):1904-11.
40. Domingo M, Lupon J, Gonzalez B, Crespo E, Lopez R, Ramos A, et al. [Noninvasive remote telemonitoring for ambulatory patients with heart failure: effect on number of hospitalizations, days in hospital, and quality of life. CARME (CAtalan Remote Management Evaluation) study]. Rev Esp Cardiol. 2011;64(4):277-85.
41. Jenkinson C, Fitzpatrick R, Brennan C, Swash M. Evidence for the validity and reliability of the ALS assessment questionnaire: the ALSAQ-40. Amyotrophic lateral sclerosis and other motor neuron disorders : official publication of the World Federation of Neurology, Research Group on Motor Neuron Diseases. 1999;1(1):33-40.
42. Moher D, Hopewell S, Schulz KF, Montori V, Gotzsche PC, Devereaux PJ, et al. CONSORT 2010 explanation and elaboration: updated guidelines for reporting parallel group randomised trials. BMJ. 2010;340:c869.
43. Schulz KF, Altman DG, Moher D. CONSORT 2010 statement: updated guidelines for reporting parallel group randomised trials. BMJ. 2010;340:c332.
44. Jenkinson C, Peto V, Jones G, Fitzpatrick R. Interpreting change scores on the Amyotrophic Lateral Sclerosis Assessment Questionnaire (ALSAQ-40). Clin Rehabil. 2003;17(4):380-5.
45. Jenkinson C, Fitzpatrick R, Swash M, Jones G. Comparison of the 40-item Amyotrophic Lateral Sclerosis Assessment Questionnaire (ALSAQ-40) with a short-form five-item version (ALSAQ-5) in a longitudinal survey. Clin Rehabil. 2007;21(3):266-72.
46. Epton J, Harris R, Jenkinson C. Quality of life in amyotrophic lateral sclerosis/motor neuron disease: a structured review. Amyotroph Lateral Scler. 2009;10(1):15-26.
47. Jenkinson C, Hobart J, Chandola T, Fitzpatrick R, Peto V, Swash M. Use of the short form health survey (SF-36) in patients with amyotrophic lateral sclerosis: tests of data quality, score reliability, response rate and scaling assumptions. J Neurol. 2002;249(2):178-83.
48. McDermott CJ, Maguire C, Cooper CL, Ackroyd R, Baird WO, Baudouin S, et al. Protocol for diaphragm pacing in patients with respiratory muscle weakness due to motor neurone disease (DiPALS): a randomised controlled trial. BMC Neurol. 2012;12:74.



For peer review only - http://bmjopen.bmj.com/site/about/guidelines.xhtml

49. Evaluation of the impact of a CoughAssist® mechanical in-exsufflator (MI-E) device on morbidity, quality of life and survival in patients with motor neurone disease (MND) using non-invasive ventilation (NIV) [Internet].
50. Julious S. Sample size of 12 per group rule of thumb for a pilot study. Pharm Stat. 2005;4(4):287-91.
51. Cedarbaum JM, Stambler N, Malta E, Fuller C, Hilt D, Thurmond B, et al. The ALSFRS-R: a revised ALS functional rating scale that incorporates assessments of respiratory function. BDNF ALS Study Group (Phase III). J Neurol Sci. 1999;169(1-2):13-21.
52. NIHR CLAHRC South Yorkshire. Telehealth & Care Technologies (TaCT) for Long Term Conditions 2012. Available from: http://clahrc-sy.nihr.ac.uk/theme-tact-introduction.html.
53. O'Rourke N, Tuokko HA. Psychometric properties of an abridged version of The Zarit Burden Interview within a representative Canadian caregiver sample. Gerontologist. 2003;43(1):121-7.
54. Zigmond AS, Snaith RP. The hospital anxiety and depression scale. Acta Psychiatr Scand. 1983;67(6):361-70.
55. Francis JJ, Johnston M, Robertson C, Glidewell L, Entwistle V, Eccles MP, et al. What is an adequate sample size? Operationalising data saturation for theory-based interview studies. Psychology & health. 2010;25(10):1229-45.
56. Hoddinott P, Pill R. Qualitative research interviewing by general practitioners. A personal view of the opportunities and pitfalls. Fam Pract. 1997;14(4):307-12.
57. Richards HM, Schwartz LJ. Ethics of qualitative research: are there special issues for health services research? Fam Pract. 2002;19(2):135-9.
58. (UK) GMC. Confidentiality. 2009.

For peer review only



For peer review only - http://bmjopen.bmj.com/site/about/guidelines.xhtml

## Appendix 1: Drugs produced by Abbott Healthcare Ltd.

Fenofibrate
Pancreatin
Moxonidine
Estradiol/dydrogesterone
Mebeverine
Betahistine
Fluvoxamine maleate
Lactulose
Estradiol, oral applications
Influenza virus vaccine
Eprosartan mesylate
Ibuprofen
Flurbiprofen
Propafenone
Clarithromycin
Verapamil

For peer review only



For peer review only - http://bmjopen.bmj.com/site/about/guidelines.xhtml

# Appendix 2: CONSORT flow diagram

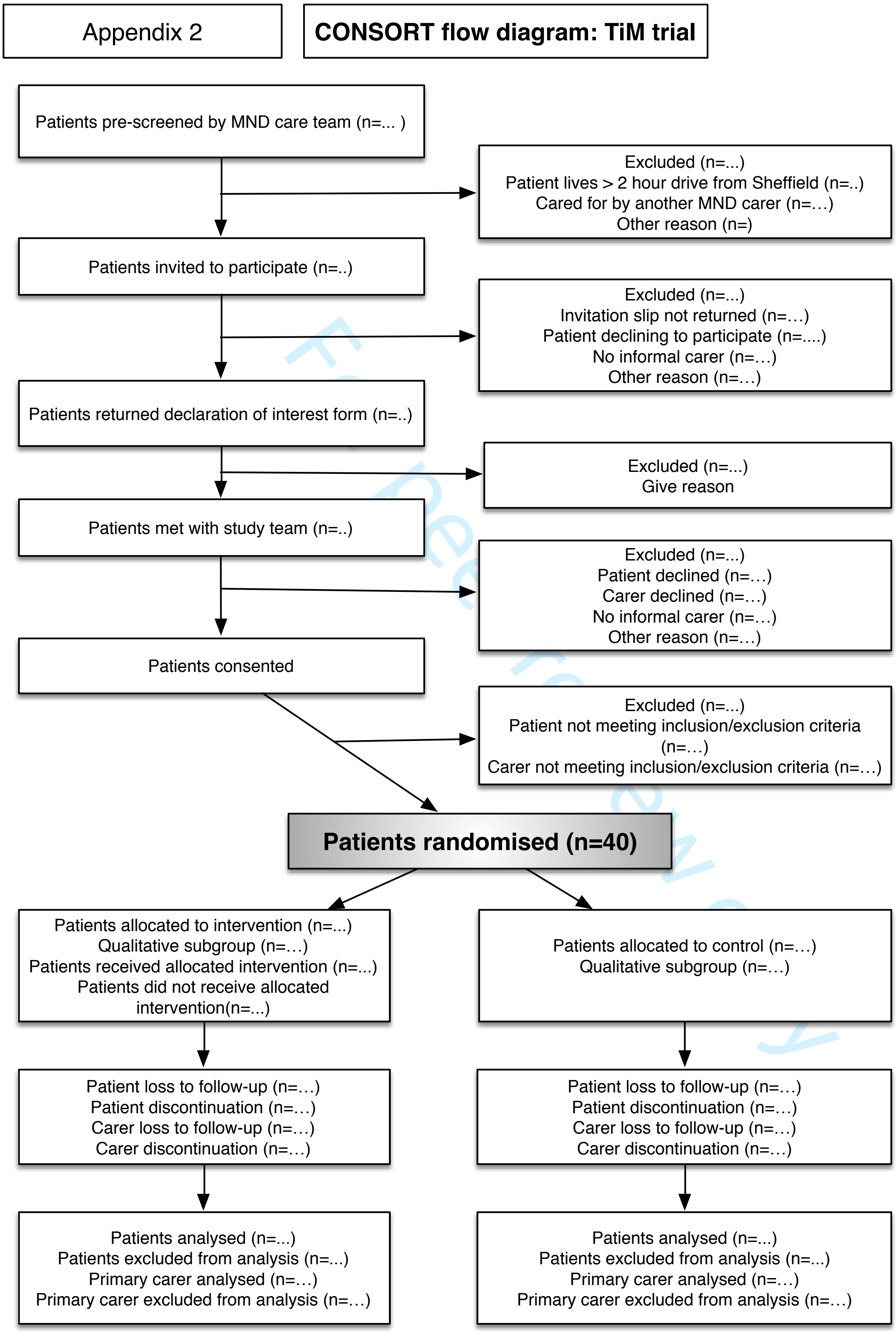




For peer review only - http://bmjopen.bmj.com/site/about/guidelines.xhtml

## Appendix 3 Qualitative interview topic guides

### Baseline interview (control group)

"I'd just like to reiterate that everything you say in the interview is confidential to me and the research team.  If anything you say is used in a publication then it will be anonymous.  I'll be recording the interview to make sure I've don't miss anything important.  It will take about 15 minutes but we can stop if you wish.  If you have any questions I can answer them now or at the end of the interview.  There aren't any right or wrong answers - I'm simply interested in your experience and your views.   I'd like to know how you found filling in the questionnaire booklet.  The questions are designed to understand more about your condition and experiences of MND."

For patients:

- Some are asking quite personal questions.  How do you feel about that?
- Do you think the questionnaires asked questions about your life with MND?
- Did you find any of the questionnaires confusing?
- Did you find any of the questionnaires upsetting?
- What would you think about filling in these questionnaires again?
- If so, how would you fill them in?
- If you needed help, how would you fill in your questionnaire?

For carers

- Did you find any of the questionnaires confusing?
- Did you find any of the questionnaires upsetting?
- Some are asking quite personal questions.  How do you feel about that?
- What would you think about filling in these questionnaires again?


For peer review only - http://bmjopen.bmj.com/site/about/guidelines.xhtml

**Intervention group**

Interviews will be conducted at 1 and 6 months. Early interview results will guide later interviews. Participants will be provided with the topic guide questions prior to the interview in order for them to communicate their answers easily. The main focus of the interviews is on:

- Participants experience and expectations of technology
- Participants' expectations of telehealth services
- Barriers and aids to recruitment and compliance with the TiM system
- How the TiM system is used at home by patients and their carers
- The impact of using the TiM system on their lives and well-being
- The impact of education on their day-to-day lives
- The experiences of carers monitoring
- Whether the outcome measures used capture the changes in participants well being associated with using the TiM system.
- How the system would be used outside a trial

**1-month interview (intervention group)**

"I'd just like to reiterate that everything you say in the interview is confidential to me and the research team. If anything you say is used in a publication then it will be anonymous. I'll be recording the interview to make sure I've don't miss anything important. It will take about an hour but we can stop at any time if you wish. If you have any questions I can answer them now or at the end of the interview. There aren't any right or wrong answers. I'd like to here about your experiences of starting using the TiM system and of MND care. You remember you received some questions in the post, we'll be going over those subjects again today"

Previous experiences in MND care

- Can you tell me a little about how you came to get the diagnosis of MND?
- What have your experiences been since then?
- Can you tell me about your last MND hospital clinic visit?
- Have you used the MND helpline?
- How do you manage if you have a question or problem?
- What would you say you are most worried about?
- How do you think your MND team have helped you?
- How do you think your care could be better?
- What problems do you think have been most troublesome?
- How much do you know about MND?


For peer review only - http://bmjopen.bmj.com/site/about/guidelines.xhtml

For carers

- How do you get the support you need as a carer?
- How do you find the help the MND team gives?
- How do you think your care could be better?
- What problems do you think have been most troublesome?
- How much do you know about MND?

Expectations of the TiM system

- Before the start of the study what technology did you use?
- What did you expect the TiM system would be like?
- Is there anything you would have hoped it would have?
- Is there anything that worried you about it?

Experiences of training and starting to use the TiM system

- What did you think when you first saw it?
- How did you find the training?
- Do you remember what it was like using it for the first time?

Barriers and facilitators to using the TiM system

- Is there anything things you like about it?
- Is there anything you don't like?
- Has it worked every time as you expected?
- Do you think you will continue to use it regularly? Why?
- What have you told your friends about it?

For the carer

- How have you found using the TiM system?
- What was it like using it for the first time?
- How have you found the questions?


For peer review only - http://bmjopen.bmj.com/site/about/guidelines.xhtml

**6-month interview (intervention group)**

"I'd just like to reiterate that everything you say in the interview is confidential to me and the research team. If anything you say is used in a publication then it will be anonymous. I'll be recording the interview to make sure I've don't miss anything important. It will take about an hour but we can stop at any time if you wish. If you have any questions I can answer them now or at the end of the interview. There aren't any right or wrong answers. I'd like to here about your experiences of using the TiM system and how it has affected your life and your MND care. You remember you received some questions in the post, we'll be going over those subjects again today"

For patients:

- How have you found using the TiM system?
- How often do you use it?
- Is it easy to use?
- Have there been any problems with it?
- Has the MND nurse contacted you about your answers?
- Have you talked about your answers during your clinic visits?
- How has it changed your MND care?
- Have you used the education section or the problem list?
- Would you like to use it as part of your routine care?
- How would you improve it?

For carers

- How have you found answering the questions?
- Has the MND nurse contacted you about your own well being?
- Would you like to use it as part of your routine care?

For peer review only



For peer review only - http://bmjopen.bmj.com/site/about/guidelines.xhtml

# Telehealth in Motor Neurone Disease

Telehealth in Motor Neurone Disease (TiM): A mixed methods, randomised controlled, pilot study of the use of the TiM telehealth system to deliver highly specialised care in Motor Neurone Disease, at a distance

**Statistical Analysis Plan Version 1.3 17/8/16**

*Authored by*


________________________________/___/_____

*Dr Esther Hobson* *Date*

*TiM trial manager & co-investigator*

*SITraN, University of Sheffield*


*Approved by*


________________________________/___/_____

*Dr Christopher McDermott* *Date*

*TiM Chief Investigator*

*SITraN, University of Sheffield*

________________________________/___/_____

*Mr Michael Bradburn* *Date*

*TiM Trial Statistician*

*CTRU, University of Sheffield*


Contents

1 Introduction, Study Design & Objectives 5



For peer review only

1.1 Study Background 5
1.2 Primary Objectives 5
1.2.1 Feasibility Outcomes 5
1.2.2 Clinical Outcomes 6
2 Sample Size Estimation 7
3 Randomisation & Blinding 8
4 Interim Analysis & Study Monitoring. 8
5 Data Sources, Evaluability & Study Populations 8
5.1 Data Sources 8
5.2 Data Collection 9
5.3 Protocol non compliances 9
5.4 Study Population 9
5.5 Analysis Populations 9
6 Statistical Analysis 10
6.1 General considerations 10
6.2 Recruitment and attrition rates 10
6.2.1 Eligibility 10
6.2.2 Participant Attrition 11
6.3 Status of participants and completion of outcome measures 11
6.4 Baseline Characteristics 11
6.5 TiM Treatment adherence 12
6.6 Clinical outcomes 13
6.6.1 Patient outcomes 13
6.6.2 Carer clinical outcomes 16
6.6.3 Health economic outcomes 16
6.6.4 Patient experiences 16
6.6.5 Safety 17
6.7 Estimation of primary outcome and sample size for a main trial 17
6.8 Economic Evaluation Analysis 18
6.9 TiM process evaluation 18
7 Detailed Statistical Methods & Calculations 19
7.1 Missing Spurious & Unused Data 19
8 Implementation of the Analysis Plan 19
9 Modifications to the Original Protocol Analysis Statement 19
10 Appendix 20
11 20
10.2 Example Tables and Figures 22
12 References 26


For peer review only - http://bmjopen.bmj.com/site/about/guidelines.xhtml

**List of Abbreviations**

| | |
|---|---|
| AE | Adverse event |
| ALS | Amyotrophic lateral sclerosis |
| ALSAQ-40 | Amyotrophic Lateral Sclerosis Assessment Questionnaire – long form |
| ALS-FRS-R | Amyotrophic lateral sclerosis rating scale-revised |
| CI | Confidence Interval |
| CONSORT | Consolidated standards of reporting trials |
| CRF | Case Report Form |
| CSS-MND | Clinical Saliva Scale for Motor Neurone disease |
| CTRU | Clinical trials research unit, University of Sheffield |
| EQ-5D-3L | EuroQol Group Health Questionnaire |
| EQ-5D+D | EQ-5D questionnaire with dignity bolt-on |
| HADS | Hospital Anxiety and Depression Scale |
| ICH | International Conference on Harmonisation of Technical Requirements for Registration of Pharmaceuticals for human use |
| ITT | Intention To Treat |
| QoL | Quality of life |
| MND | Motor neurone disease |
| NIV | Non-invasive ventilation |
| SAE | Serious adverse event |
| SAP | Statistical analysis plan |
| SD | Standard deviation |
| SF-36 RAND | 36-Item Short Form Survey from the RAND Medical Outcomes Study |
| SITraN | Sheffield Institute of Translational Neuroscience |
| SOP | Standard operating procedure |
| Telehealth | Remote monitoring of patients physiology or patient reported measures, forwarded to a central service with the aim to diagnoses or monitor a medical condition |
| TMG | Trial management Group |
| TSC | Trial Steering Committee |
| TiM | Telehealth in Motor neurone disease |
| TM | Trial manager (EH) |
| ZBI | Zarit Burden Index |

For peer review only



For peer review only - http://bmjopen.bmj.com/site/about/guidelines.xhtml

1. Introduction, Study Design & Objectives

This Statistical Analysis Plan (SAP) is written in conjunction with the ICH E9 (International Conference on Harmonisation of Technical Requirements for Registration of Pharmaceuticals for human use; ICH Harmonised Tripartite Guideline: Statistical Principles for Clinical Trials E9), applicable standard operating procedures (SOPs) from the Sheffield Clinical Trials Research Unit (CTRU) and trial documents (Protocol, case report form (CRF) and Data Validation Specifications). This SAP will guide the trial manager (TM) and Trial Statistician during the statistical analysis of all quantitative outcomes in order to answer the objectives of the study.

## 1.1 Study Background

This is a single-centre, pilot, mixed methods, randomised controlled trial to explore the feasibility and acceptability of using the TiM (Telehealth in Motor Neurone Disease (MND)) system in clinical practice and explore the feasibility of a larger, multicentre trial. This plans refers to the TiM trial protocol V1.5 April 2015.

All analyses will be performed in a validated statistical software package such as GraphPad prism.

## 1.2 Primary Objectives

As this is a pilot study, no formal primary clinical outcome will be defined. Instead, the trial will assess the feasibility and requirements of a full-scale study of the TiM as defined by the as successful recruitment of 40 eligible patients and their primary carer; and the feasibility, acceptability, safety and use of the TiM system within a health service. The specific objectives and outcomes of this study are separated into two groups: feasibility and clinical outcomes.

### 1.2.1 Feasibility Outcomes

**Feasibility of a full-scale study**

- To make a decision on the primary outcome for the main trial. The mechanism for choosing this outcome will be informed by statistical considerations which are detailed in section 6.7.
- Number of potentially eligible patients among the pool of patients under the care of the Sheffield MND care centre
- Number/characteristics of eligible patients approached for the study:
- List of reasons for declining/refused consent;
- Participant attrition rate
- List of reasons for attrition
- Number of missing values/incomplete cases (see **Error! Reference source not found.**)



For peer review only - http://bmjopen.bmj.com/site/about/guidelines.xhtml

- Treatment receipt/adherence;
- Patient, carer and clinician views on intervention/research protocol (using qualitative methods).

Feasibility/safety of TiM system:

- Treatment receipt/participant and staff adherence
- Participant and clinician acceptability of the intervention (using qualitative methods and the TiM system experience questionnaire and Shadow monitoring protocol)
- Patient, carer and clinician views on intervention (using qualitative methods);
- Incidence of TiM system technical problems;
- Incidence of adverse events related to intervention.

Participant and clinician views will be investigated using qualitative interviews (described in the protocol). Participant and clinician acceptability will be reported based on TiM system experience questionnaire and Shadow monitoring questionnaire.

### 1.2.2 Clinical Outcomes

The following clinical outcomes will be reported using self-completed questionnaires baseline, 3, 6, 12 and 18 months.

Patient outcomes

- Quality of life (QoL) measures
    - ALSAQ-40 (an MND disease specific quality of life score)
    - SF-36-RAND
    - EQ-5D+D (EQ-5Q-3L with a dignity bolt-on)
- Clinical outcomes
    - ALS-FRS-R (an MND disease specific functional rating score
    - Pain score (modified Likert scale)
    - CSS-MND Saliva Severity Scale (designed for use with MND patients) plus global change scale
    - Hospital Anxiety and Depression Scale (HADS)
- Health resource usage questionnaire
- Patient experience questionnaire
- TiM experience questionnaire

Carer outcomes

- SF-36 RAND
- 12 item Zarit Burden Inventory (ZBI) (53)
- Hospital Anxiety and Depression Scale (54)
- Carer satisfaction questionnaire



For peer review only - http://bmjopen.bmj.com/site/about/guidelines.xhtml

The following safety outcomes will be assessed at every clinical visit:

- Incidence of adverse events (AEs)
- Clinician satisfaction

## 2 Sample Size Estimation

The study aims to recruit a total of 40 patients and their carers. 20 patients and their primary carer will be randomised to the intervention arm (a minimum of 6 months use of the TiM telehealth plus usual care) and 20 patents and their carer in the control arm (usual care).

Since the proposed trial is primarily an assessment of the acceptability of the intervention and the feasibility of a full trial, the proposed sample size is not based on standard statistical parameters such as a clinically relevant difference between groups. Instead, the sample size is justified on the grounds of quantifying patient variance (i.e. the standard deviation) in the proposed outcome measures (in particular quality of life measures) and on feasibility of the full trial, as follows:

- A sample size of 40 patients allows a standard deviation to be estimated to within a precision of ±20% of its true underlying value with 90% confidence. This estimate will be synthesised by combining baselines measurements of quality of life measurement standard deviations with those observed in other published studies and on-going trials within SITraN, to provide a robust estimate for use in the sample size calculation for the full trial.

- Given the rarity of MND, any definitive study will be infeasible if the required sample size is substantial. Assuming the upper limit for feasible UK study is around 200-300 patients in total, it follows that the full study would need powering to detect a standardised effect size of at least 0.4 SDs. This pilot trial will provide a preliminary assessment of whether the intervention might feasibly achieve this, and inform the choice of outcome measures for the proposed full study.

This sample size is also in keeping with the proposal of 12 evaluable patients per arm in a pilot study (after withdrawal or drop-out) (1).



For peer review only - http://bmjopen.bmj.com/site/about/guidelines.xhtml

# 3 Randomisation & Blinding

Randomisation is conducted according to the protocol.

The patient, clinicians, TM and trial team are not blinded to the outcomes. Data entry for follow-up clinical outcomes was performed by an independent research nurse, not involved in the study. Blinding of this nurse was impractical given additional measures were collected for those in the intervention group. The TM will undertake the analysis under the supervision of the independent trial statistician. Blinding during analysis was impractical given the small number of participants who had with unique and characteristics which are likely to be identifiable to the TM. This will be reported as a limitation

# 4 Interim Analysis & Study Monitoring.

This is a pilot study with no planned interim analysis or early stopping. Two committees have been set up to govern the conduct of the study:

- Trial Steering Committee (TSC)
- Trial Management Group (TMG)

Decisions to stop the trial early on grounds of safety will be made by the Trial Steering Committee or funding body. There will not be a Data Monitoring and Ethics Committee for this study as it is considered low risk. No interim analysis is planned.

The TM will receive notifications of all carers whose Hospital Anxiety and Depression subscores exceed 11. These events will be recorded as AEs, reported to the TMG and TSG during the study and reported in the analysis.

# 5 Data Sources, Evaluability & Study Populations

## 5.1 Data Sources

Data used in this study will come from data entered onto CRFs and questionnaires and from data entered directly on the CTRU database (PROSPECT). The data will be stored on the database with the exception of the randomisation list which is held on www.sealedenvelope.com and allocation verified by the data management team. Electronic data will be extracted from the system during the trial for the purpose of checking (validating) and trial progress reports. Access to PROSPECT is controlled by usernames and encrypted passwords, and a privilege management feature will be used to ensure that users have access to only the minimum amount of data required to complete their tasks. This will be used to restrict access to personal identifiable data.



For peer review only - http://bmjopen.bmj.com/site/about/guidelines.xhtml

### 5.2 Data Collection

Data will be collected from the participants and their carers at:

- Consent and Screening, eligibility and baseline
- Month 3, 6, 12 and 18
- Each clinic visit (Shadow monitoring protocol)
- End of study (participant status alive/dead and date of death).

**Due to the pilot nature of the study there are no predefined protocol non compliances other than misrandomisation or randomisation in error. Intervention adherence will be assess as an outcome (see section 6.5).**

### 5.3 Protocol non compliances

**Due to the pilot nature of the study there are no predefined protocol non compliances other than misrandomisation or randomisation in error. Intervention adherence will be assess as an outcome (see section 6.5).**

### 5.4 Study Population

Described in the protocol.

### 5.5 Analysis Populations

The intention to treat population (ITT) includes all patients for whom consent is obtained and who are randomised to treatment. This is the primary analysis set and endpoints will be summarised for the intention to treat population unless stated otherwise.


For peer review only - http://bmjopen.bmj.com/site/about/guidelines.xhtml

# 6 Statistical Analysis

## 6.1 General considerations

As the trial is a pilot parallel group randomised controlled trial, data will be reported and presented according to the proposed modifications for reporting pilot trials as well as the Consolidated standards of reporting trials (CONSORT) statement (2,3). The analysis will be performed on an ITT basis. The final analysis will be performed after data lock by the TM under the supervision of the study statistician who will also be responsible for quality checking the results.

**Each planned follow-up timepoint will use a time window to ensure that responses have been collected within a reasonable time frame. The time windows allow a slippage of four weeks at 3 months and six weeks thereafter, as outlined below:**

**3 months:** **within 61-91 days following randomisation**

**6 months:** **within 140-224 days following randomisation**

**12 months:** **within 323-407days following randomisation**

**18 months:** **within 506-590 days following randomisation**

## 6.2 Recruitment and attrition rates

Relevant summaries related to recruitment, consent and patient throughput will be reported and presented in a CONSORT flow diagram (see appendix, Figure 1).

The following will be reported:
The number of (potential) participants;

- Potentially eligible as identified by the study team at participating centres,
- Approached for the study,
- Not randomised (with reasons),
- Randomised,
    - allocated to treatment
    - allocated to control
- Withdrawn and lost to follow up (with reasons),
- Discontinuing TiM intervention,
    - reasons for discontinuation
- Included and excluded from analysis,
    - Reasons for exclusion.

### 6.2.1 Eligibility

Described in the study protocol



For peer review only - http://bmjopen.bmj.com/site/about/guidelines.xhtml

### 6.2.2 Participant Attrition

The rate of attrition will be reported (defined as the proportion of the consented and randomised participants who withdrew or were lost to follow up). The reasons for attrition, where provided, will be reported as number and percentage in each category.

## 6.3 Status of participants and completion of outcome measures

We will report the status of patients and carers at each time point.

At each time-point we will report the number of patients and carers:

- Returning the postal questionnaire booklet
- Completing each questionnaire

We will report these by treatment group and overall.

For the patient and carer questionnaires the response rate at each time point (measured as the total number of questionnaires completed as a fraction of total number of patients alive) will be reported. An example table is given in section 0 (Table 1).

## 6.4 Baseline Characteristics

The baseline demographics and clinical characteristics of the participants will be reported. For the continuous variables, (e.g. age) either mean and standard deviation will be presented or median and inter quartile range (IQR) depending on the distribution of the data. The number of observations used in each calculation will be presented alongside the summaries. For the categorical variables, the number and percentage of participants in each of the categories and the total number of observations will be presented.

All baseline summaries will be presented and reported for each treatment group and in total. An example of the table of baseline summaries is given in section 0 (

For peer review only

Table 2). No statistical significance testing will be done to test baseline imbalances between the intervention arms but any noteworthy differences will be descriptively reported.

For peer review only



For peer review only - http://bmjopen.bmj.com/site/about/guidelines.xhtml

The following summaries will be presented:

| | |
|---|---|
| Demographics | Age, gender, technology use |
| MND Characteristics | Age of onset, disease duration, classification of MND (e.g. ALS, PMA, PLS), clinical stage of MND, use of non-invasive ventilation (NIV)/gastrostomy, riluzole use |
| Carer demographics | Age, gender, relationship to patient, technology use |
| Patient reported outcomes | ALS-FRS-R (including upper limb function), ALSAQ40, RAND36 and subscores, CSS-MND, HADS, pain score, EQ-5D+D, patient experience, health resource use (number and type of clinical encounters and hospital admissions in last 3 months, carer requirements) |
| Carer reported outcomes | RAND36, ZBI, HADS, carer experience |

## 6.5 TiM Treatment adherence

**Intervention adherence will be reported as the number of TiM sessions attended within between recruitment and the end of March 2016 and the mean and SD of percent adherence. We will also report adherence at 1, 3, 6, 9, 12, 15 and 18 months.**

**We will also report**

- **The number and percentage of participants that completed 50% and 75% of expected sessions.**
- **A description of the adherence of each patient and carer using the TiM over the course of the trial.**

**Any reasons for poor adherence will be reported where available although it was not possible to identify reasons for all missed sessions.**

**Cumulative session attendance will be displayed for each participant using a spaghetti plot to illustrate intervention adherence.**

**The number and percentage of participants that withdrew from the TiM intervention will be reported, alongside listings of:**


For peer review only - http://bmjopen.bmj.com/site/about/guidelines.xhtml

- **Reasons for withdrawing from intervention, where provided**
- **Number of TiM sessions (and %) before withdrawing from intervention**

## 6.6 Clinical outcomes

Descriptive statistics will be presented for the clinical outcomes; significance testing will not be undertaken. Continuous outcome measures will be presented as mean differences between groups and their associated 95% confidence intervals (CI). For categorical outcomes, the number and percentages falling into different categories and potential differences between groups in terms of the percentages in each category will be presented, together with their confidence intervals. Clinical outcomes will be presented for the ITT set with available 6 month and 12 month outcome data.

### 6.6.1 Patient outcomes

The following outcomes measured at 3, 6, 12, 18 months will be presented by group and overall.

The following patient –reported quality of life outcomes will be reported.

| | |
|---|---|
| ALSAQ-40 | Individual scores of five sub-scales and a summary aggregate score:<br>▪ physical mobility<br>▪ activities of daily living and independence<br>▪ eating and drinking<br>▪ communication<br>▪ emotional reactions |
| RAND-36 | A summary of the eight sub-scales and two aggregated scales:<br>▪ Physical Functioning<br>▪ Role Limitations due to Physical Problems<br>▪ General Health Perceptions<br>▪ Vitality<br>▪ Social Functioning<br>▪ Role Limitations due to Emotional Problems<br>▪ General Mental Health<br>▪ Health Transition<br>▪ Aggregate physical health<br>▪ Aggregate mental health |
| EQ-5D+D | Health utility (as derived from the five questions)<br>Thermometer health scale<br>Health utility plus dignity (as derived five questions plus |


For peer review only - http://bmjopen.bmj.com/site/about/guidelines.xhtml

| | |
|---|---|
| | dignity bolt-on) |

In each case the within-group results will be summarised as mean (SD), and the difference between the two as the mean difference together with its CI. Forest plots of confidence intervals of different widths (e.g. 95%, 90%, 80%) with respect to the treatment difference in the overall ALSAQ40 score and RAND36 (mental and physical domain) will be used to illustrate the strength of preliminary evidence (see Figure 2) (Lee, 2014).

In each case, the summaries will be presented by treatment group and time point (see Table 3 and

For peer review only



For peer review only - http://bmjopen.bmj.com/site/about/guidelines.xhtml

ll be presented for the clinical outcomes; significance testing will not be undertaken. significance testing will not be undertaken. Continuous outcome measures will be ignificance testing will not be undertaken. Continuous outcome measures will be presented as mean differences between groups and their associated 95% confidence erences between groups and their associated 95% confidence intervals (CI). For 5% confidence intervals (CI). For categorical outcomes, the number and percentages % confidence intervals (CI). For categorical outcomes, the number and percentages ntages falling into different categories and potential differences between groups in potential differences between groups in terms of the percentages in each category will potential differences between groups in terms of the percentages in each category will ome data.

ll be presented by group and overall.

tcomes will be reported.

ary aggregate score:

obility

g

-36

le 3 and

e number of patients alive and the number of patients completing the outcome

score mean and SD

ure will be scored as described below and compared, where possible and relevant to

ompared, where possible and relevant to population values.

be scored as described in Ware et al (4). In the case of partially completed 9D4-BDB0-

C034</uuid><priority>0</priority><publications></publications></citation>(4). In publications></citation>(4). In the case of partially completed questionnaires, scores questionnaires, scores will be calculated for domains in which at least 50% of the ons have been answered. Taking physical functioning as example, if at least five of the ns have been answered. Taking physical functioning as example, if at least five of the

lity (Q1-10), activities of daily living/independence (Q11-20), eating and drinking,

ity (Q1-10), activities of daily living/independence (Q11-20), eating and drinking, (Q21-

(Q21-23) communication (Q24-30), emotional functioning (Q31-40). Each question is unctioning (Q31-40). Each question is scored 0 (never) to 4 (always/cannot do at all).

nctioning (Q31-40). Each question is scored 0 (never) to 4 (always/cannot do at all).

5 +Q6 +Q7 +Q8 + Q9 + Q10)/40) x 100.

l).

urology, Neurosurgery & Psychiatry</title><type>-100</type><subtype>-0</subtype><uuid>0303282F-D994-4EC9-B986-

bundle><authors><author><firstName>C</firstName><lastName>Jenkinson</lastNa

or><firstName>C</firstName><lastName>Jenkinson</lastName></author><author><f

stName>Jenkinson</lastName></author><author><firstName>J</firstName><middle

tName>Jenkinson</lastName></author><author><firstName>J</firstName><middleN



For peer review only - http://bmjopen.bmj.com/site/about/guidelines.xhtml

The following other clinical outcomes will be presented:

| | |
|---|---|
| Hospital anxiety and depression score | The Anxiety and Depression subscores |
| Pain (Likert scale) | Current and average weekly score |
| ALS-FRS-R | Total score |
| CSS MND | Total score<br>% patients reporting a clinically significant improvement or worsening (according to Global change CSS-MND self-reported statement) |
| Clinical encounters | The number of clinical encounters in the 6 months following randomisation, by type and location and reason |
| Hospital admissions | The number and percentage of patients admitted to hospital, and the number of hospitalisations, by type, location and reason |

The HADS anxiety and depression inventories will be scored using the approach of Zigmond and Snaith (7). Each domain will be calculated as the sum of seven questions, each of which is scored 0-3, giving a total score which ranges from 0 and 21. In the case of partially completed questionnaires, the domain will be scored and upweighted provided at least four of the seven questions have been answered.

Self-completed revised ALS functional rating scale (ALS-FRS-R) consists of 12 questions scoring 0-4 (8). Sub-domains include upper limb, lower limb, bulbar and respiratory.

Pain score: the current level of pain (0-10 likely scale) and the average current weekly level of pain (0-10) will be represented as a mean and SD.

Modified CSS MND saliva score is awaiting validation. The total score is the total of all answers scoring 0-3 for each question A to J (9). The percentage of patients reporting a change on the saliva clinical change assessment will be reported.

### 6.6.2 Carer clinical outcomes

The following carer-reported QoL outcomes will be presented is the same manner as described for the patients.

- RAND 36
- HADS



For peer review only - http://bmjopen.bmj.com/site/about/guidelines.xhtml

- Zarit Burden Index

The each of the 12 items in the shortened Zarit burden inventory is scored 0 (Never) to 4 (nearly always) (10). A total score between 0 and 48 will be reported.

### 6.6.3 Health economic outcomes

A complete health economic analysis is beyond the scope of this plan. However, descriptions of the following clinical outcomes will be reported, by group and overall:

| | |
|---|---|
| Clinical encounters | The number of clinical encounters recorded at each encounter following randomisation, by type and location. |
| Hospital admissions | The number and percentage of patients admitted to hospital recorded at each encounter. the number of hospitalisations |
| Informal care requirements | The number of hours of informal care recorded by patients. |
| Formal care requirements | The number of hours of formal care recorded by patients. |

### 6.6.4 Patient experiences

The following will be reported by group and overall.

| | |
|---|---|
| Patient care experience | Percentage of patients agreeing and disagreeing with each satisfaction statement |
| Carer care experience | Percentage of carer agreeing and disagreeing with each satisfaction statement |
| Patient TiM experience (intervention only) | Percentage of patients agreeing and disagreeing with each satisfaction statement |
| Carer TiM experience (intervention only) | Percentage of carer agreeing and disagreeing with each satisfaction statement |

All free text responses will be reported.

### 6.6.5 Safety

Adverse events are recorded at every clinic appointment and patients will report health resource use and hospital admissions. Reported admissions will be followed up by the



For peer review only - http://bmjopen.bmj.com/site/about/guidelines.xhtml

TM and records as serious adverse events. HADS carer scores will be calculated and reported to the TM for action on an ongoing basis during the trial if either the depression or the anxiety subscore exceeds 11. These will be reported to the TSC during the trial and reported in the analysis and recorded as adverse events.

Advents Events (AEs) will be reported as number and percentage of patients overall and by treatment group but no formal statistical analysis is planned. The following summaries will be presented;

| | |
|---|---|
| *AEs* | The number and percentage* of patients reporting an AE and the number of AEs in total |
| *AEs by category* | The number and percentage* of patients reporting an AE and the number of AEs for each pre-defined category (pain, acute infection, fractures) |
| *Serious AEs (SAEs)* | The number and percentage* of patients reporting an SAE and the number of SAEs in total |
| *Treatment-related AEs* | The number and percentage* of patients reporting a treatment related AE and the number of treatment related AEs |
| *All AEs* | A listing of all AEs including<br>- Description / Site / Signs and Symptoms<br>- Severity<br>- Relationship<br>- Action taken<br>- Outcome<br>- Seriousness |

*defined as a percentage of all patients randomised.

## 6.7 Estimation of primary outcome and sample size for a main trial

The variability in clinical outcomes will be reported as standard deviation by treatment group and overall alongside their upper 80% confidence limits to get a robust estimate of SD (as recommended by Kieser, 2007), and observed treatment difference.

Descriptive assessment will be used to inform sample size calculations for the definitive study. These assessments will be calculated for candidate measures for the full trial (RAND-36 and ALSAQ40), and will be based on:

- Observed treatment difference at 6 and 12 months
- Standard Deviation;
- Correlation between baseline and 6 month measurements;
- The extent of missing data in each outcome;
- Participant feedback on the most appropriate assessment (analysed qualitatively).


For peer review only - http://bmjopen.bmj.com/site/about/guidelines.xhtml

The standard deviation used in the sample size calculation will be derived from the residual variance of the regression model for which the outcome is the 6-month response and the covariates are treatment group and baseline.

A table of sample size estimates for a definitive study stratified by outcome measure and power (80%, 90%) will be provided. E.g. Table 5

## 6.8 Economic Evaluation Analysis

No economic analysis will be conducted but patient health resource use will be reported.

## 6.9 TiM process evaluation

The following will be reported:

Patient and carer feasibility:

- The time taken to complete each TiM session by patient and carer (mean, range). TiM session time is automatically recorded by the application but total time between starting and completing and session is recorded. This includes any time delay because the patient pauses using the session and recommences it later e.g. the next day. Outliers will be identified and excluded with definition of outlier reported (e.g. > 600% of the average time);
- Adherence to weekly TiM sessions (see 6.5);

Clinical feasibility:

- Number, range and % of patient and carer sessions that trigger an overall red, amber and green flag;
- Number, range and % of patient and carer sub-sections that trigger an overall red, amber and green flag;
- Time taken for nurse to use the telehealth system per week, collected by nurse diary (mean, range, SD and time per patient enrolled in the system);
- Number of notes entered per patient.
- Shadow monitoring protocol (intervention)
    - Number of pre-clinic shadow monitoring forms completed
    - Number of clinic shadow monitoring forms completed
    - Clinician satisfaction: % agree/disagree with each statement
    - Free text comments will be reported.



For peer review only - http://bmjopen.bmj.com/site/about/guidelines.xhtml

# 7 Detailed Statistical Methods & Calculations

## 7.1 Missing Spurious & Unused Data

The extent of missing data will be reported. No sensitivity analyses involving imputation for missing data will be performed. Any spurious data will be queried and checked for consistency with data management before data lock.

Patient and carer questionnaires will be scored only if all relevant items that make up a domain are completed with the exception of RAND 36, HADS and ALSAQ40.

# 8 Implementation of the Analysis Plan

This SAP will be used as a work description for the statistician involved in the trial. All analyses will be performed by the TM (under the supervision of Trial Statistician MB).

Initially, blinded data will be delivered to the TM and MB by the data manager to define analysis sets and test statistical programs. Any queries will be communicated to the study and data manager prior to database lock. The database will be locked after agreement between the statistician, data manager and study manager. No changes will be made once the data has been locked. Database freeze and lock will be conducted in accordance with SOP DM012.

# 9 Modifications to the Original Protocol Analysis Statement

**None**

For peer review only

# 10 Appendix

*Figure 1: CONSORT flow diagram*

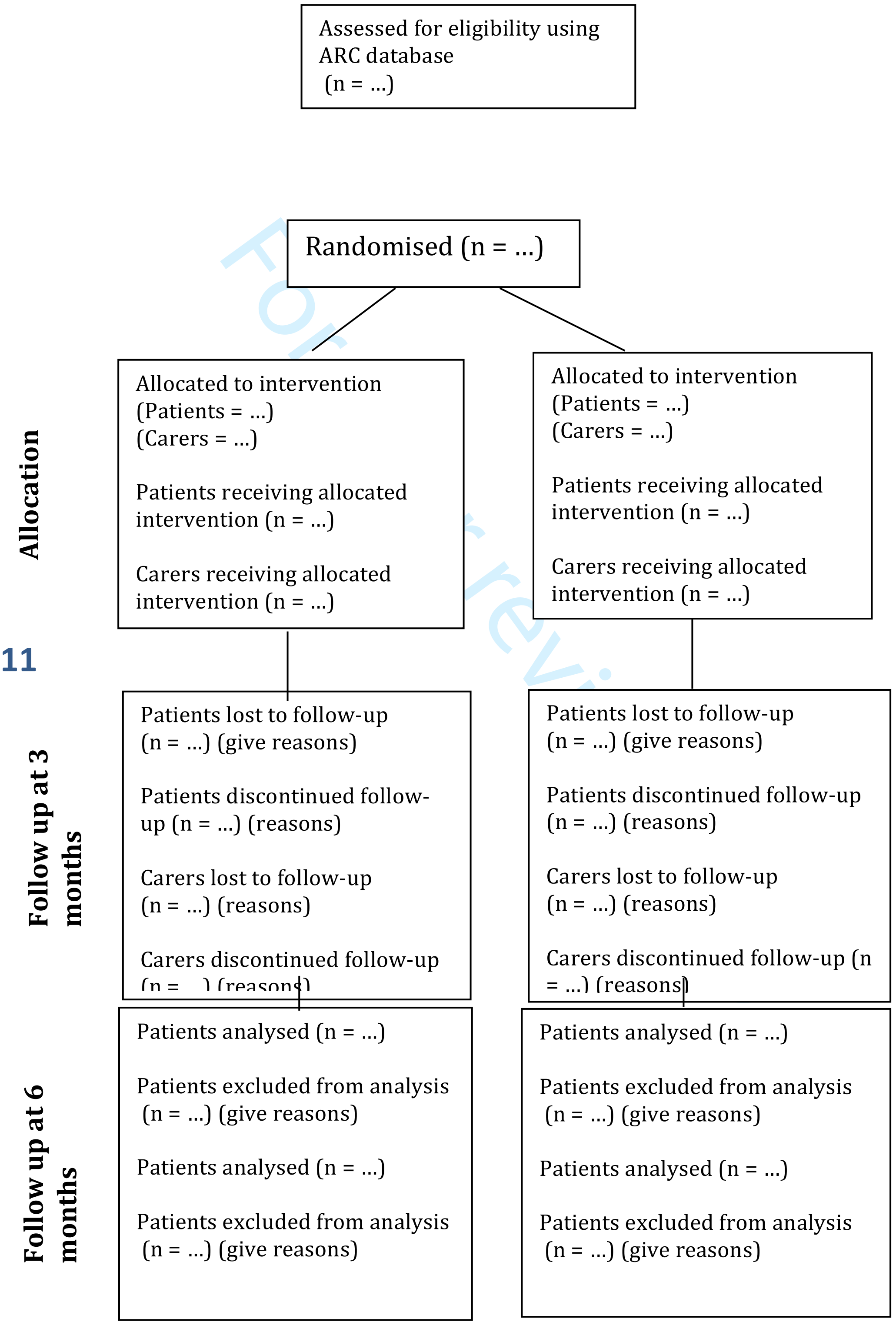


## 11



For peer review only - http://bmjopen.bmj.com/site/about/guidelines.xhtml

*Figure 2: Mean difference in ALSAQ40 with confidence intervals*

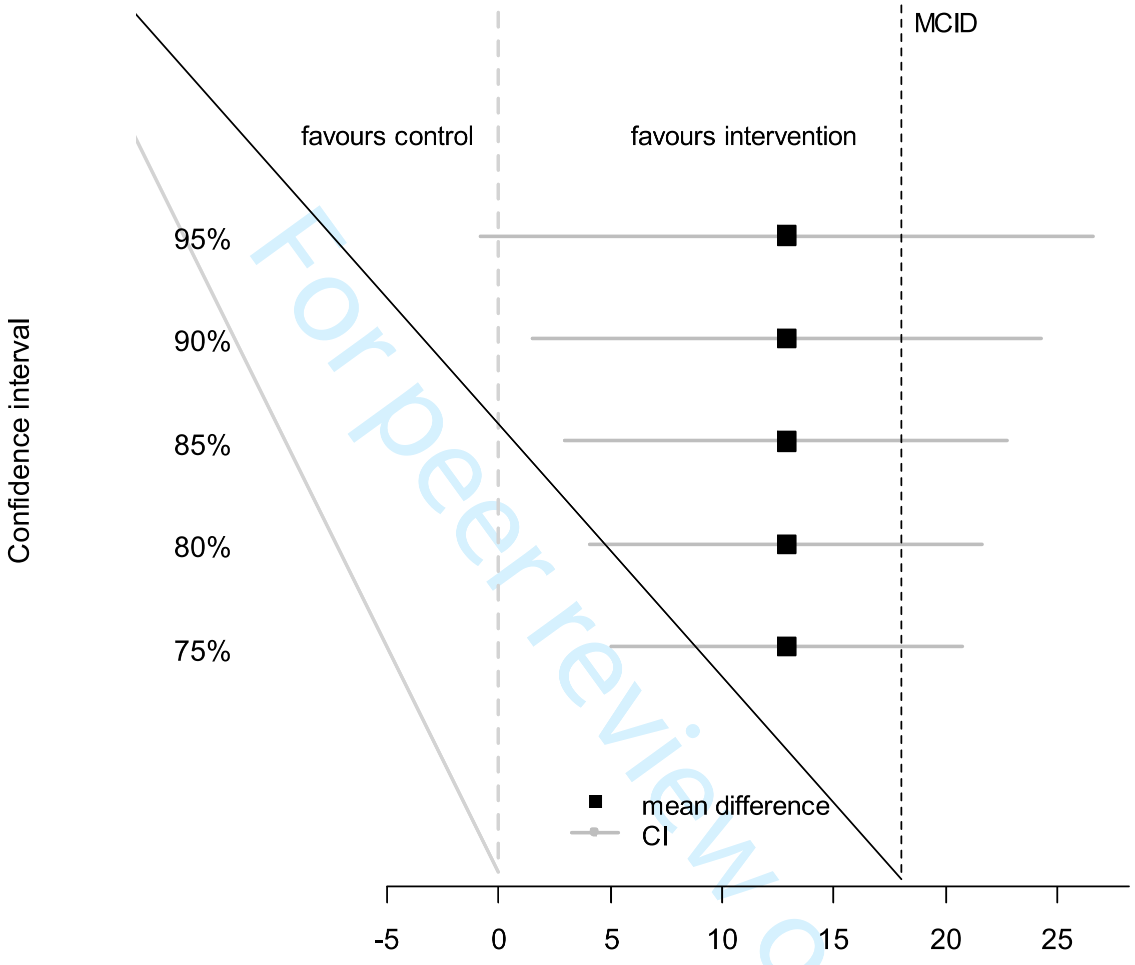


For peer review only

## 10.2 Example Tables and Figures

Note: The following tables are examples and do not include all outcome measures that will be included in the analysis.

*Table 1: Participant status*

| | 3 months | 6 months | 12 months | 18 months |
|---|---|---|---|---|
| **Telehealth** | | | | |
| Completed | N= | N= | | |
| Not completed | | | | |
| Died | | | | |
| Withdrew from study | | | | |
| Completed questionnaire but not within time window | | | | |
| Incomplete | | | | |
| **Control** | | | | |
| Completed | N= | N= | | |
| Not completed | | | | |
| Died | | | | |
| Withdrew from study | | | | |
| Completed questionnaire but not within time window | | | | |
| Incomplete | | | | |

*Completed includes questionnaires that were sufficiently complete to be used in the statistical analysis. Uncompleted refers to questionnaire booklets that were not returned. Incomplete refers to questionnaire booklets that were returned but insufficiently complete to be used in statistical analysis.

For peer review only



For peer review only - http://bmjopen.bmj.com/site/about/guidelines.xhtml

*Table 2: Participant baseline characteristics by treatment group*

| Characteristic | Scoring | Control (n=xx) | Intervention (n=xx) | All (n=xx) |
|---|---|---|---|---|
| Age (years) | Mean(SD, range) | x (xx) | x (xx) | x (xx) |
| | N | n | n | n |
| Gender | Male | n (%) | n (%) | n (%) |
| | Female | n (%) | n (%) | n (%) |
| | N | N | n | n |
| ALS-FRS-R | Mean(SD, range) | x (xx) | x (xx) | x (xx) |
| | N | n | n | n |
| King's clinical stage | Stage 1 | x (xx) | x (xx) | x (xx) |
| | Stage 2…etc. | | | |
| | N | n | n | n |

*This will be extended to include the other baseline variables measured.*

*Table 3: Display of outcome data by time, illustrated for pain*

| | Baseline | 3 months | | | |
|---|---|---|---|---|---|
| Outcome | | Mean (SD) | Change from baseline | *…repeat for other timepoints* | |
| Current pain: Mean (SD) | Mean (SD) | N= Mean (SD) | Mean (CI) | | |
| Control | Mean (SD) | N= Mean (SD) | Mean (CI) | | |
| Average pain: Mean (SD) | Mean (SD) | N= Mean (SD) | Mean (CI) | | |
| Control | Mean (SD) | N= Mean (SD) | Mean (CI) | | |



For peer review only - http://bmjopen.bmj.com/site/about/guidelines.xhtml

*Table 4: Clinical outcomes at six months: control vs intervention*

| | Change from baseline | | | | | | |
|---|---|---|---|---|---|---|---|
| | Intervention | | | Control | | | |
| Outcome | n | Mean | SD | n | Mean | SD | Mean difference (95% CI) |
| ALSAQ40 | x | xx | xx | x | xx | xx | xx (xx to xx) |
| RAND 36 (agg. physical) | x | xx | xx | x | xx | xx | xx (xx to xx) |
| RAND 36 (agg. mental) | x | xx | xx | x | xx | xx | xx (xx to xx) |
| HADS anxiety | x | xx | xx | x | xx | xx | xx (xx to xx) |
| HADS depression | x | xx | xx | x | xx | xx | xx (xx to xx) |
| Pain | x | xx | xx | x | xx | xx | xx (xx to xx) |
| ... | | | | | | | |



For peer review only - http://bmjopen.bmj.com/site/about/guidelines.xhtml

Table 5: Sample size considerations for candidate primary outcome measures

| | Effect size | | Standard deviation | | Power (%) | Number /group | Number /group + attrition |
|---|---|---|---|---|---|---|---|
| Outcome | MCID | Observed* | | | | | |
| ALSAQ-40 total | xx | Xx | Observed | xx | 80 | NN | NN |
| | | | | | 90 | NN | NN |
| | | | Upper 80%CI | xx | 80 | NN | NN |
| | | | | | 90 | NN | NN |
| RAND-36 | | | | | | | |
| Agg physical | 5 | Xx | Observed | xx | 80 | NN | NN |
| | | | | | 90 | NN | NN |
| | | | Upper 80%CI | xx | 80 | NN | NN |
| | | | | | 90 | NN | NN |
| Agg mental | 5 | xx | ... | | | | |
| *...repeat for other candidate measures* | | | | | | | |

**nb Observed effect size is for reference and is not used in sample size calculation*

For peer review only

TiM Telehealth in Motor Neurone Disease

# Shadow monitoring form

[Intervention]

| T | | | |
|---|---|---|---|

## Appointment summary

Date of clinic: d d / m m / y y y y

Neurologist completing form: ☐ CMD ☐ PJS ☐ Other → ________ *specify*

Mode of contact: ☐ Face-to-face ☐ Telephone

Type of appointment: ☐ Routine ☐ Unscheduled

Related to telehealth: ☐ Yes ☐ No

Details: ________

Next scheduled visit: ☐☐ weeks

## Adverse events

Any adverse events (including hospital visits) since last visit? ☐ Yes ☐ No

*Please complete* ***Adverse event*** *or* ***Hospital attendance*** *form and contact CI immediately*

Any adverse drug reactions since last visit? ☐ Yes ☐ No

*Please complete* ***Adverse event*** *form and contact CI immediately*

## TiM

| | Completely disagree | Disagree | Neutral | Agree | Completely agree |
|---|---|---|---|---|---|
| The TiM data gave an accurate picture of the patient's current condition | ☐ | ☐ | ☐ | ☐ | ☐ |
| The TiM data could enable me to make appropriate decisions about the patient without seeing them in clinic | ☐ | ☐ | ☐ | ☐ | ☐ |
| The TiM data gave useful information about the carer's current condition | ☐ | ☐ | ☐ | ☐ | ☐ |
| The TiM was a positive influence on the consultation | ☐ | ☐ | ☐ | ☐ | ☐ |
| Prior to the patient attending you said you would arrange the patient to attend clinic in ______ weeks. Do you think this was an appropriate decision? | ☐ | ☐ | ☐ | ☐ | ☐ |

**Telehealth in motor neurone disease to increase access to specialist multidisciplinary care: a process evaluation.**

**Supplementary data file**

## Table of Contents

Appendix table 1 Participants' initial impressions of using the TiM app. .................. 2
Appendix table 2 The characteristics of those lacking confidence in technology user and their approach to using technology. ........................................ 3
Appendix table 3 Negative attitudes to technology expressed by participants....... 4
Appendix table 4 Facilitators for TiM use. ........................................ 5
Appendix table 5 Participants attitudes towards the frequency of TiM sessions... 6
Appendix table 6 Reasons for frequent and infrequent adherence to TiM identified during the interviews. ........................................ 7
Appendix table 7 Participants' expectations of the potential communication and monitoring benefits of the TiM service. ........................................ 8
Appendix table 8 Participants' expectations of the TiM service. ........................................ 9
Appendix table 9 The acceptability of the TiM questions. ........................................ 10
Appendix table 10 The validity / accuracy of the TiM questions. ........................................ 11
Appendix table 11 Participants understanding of the TiM service. ........................................ 12
Appendix table 12 Nurses' experience of using the TiM clinical portal. All quotes are from the Telehealth Nurse. ........................................ 13
Appendix table 13 The accuracy and sensitive of the TiM information. Quotes are from the Telehealth Nurse (N) unless otherwise stated. ........................................ 14
Appendix table 14 Participants' experiences of how the TiM impacted on their care. ........................................ 15
Appendix table 15 The Telehealth Nurse's attitudes towards the value of the TiM. ........................................ 16
Appendix table 16 Participants experiences of clinician feedback. ........................................ 17
Appendix table 17 The mismatch between the participants' and nurses' expectations of the TiM service. ........................................ 18
Appendix table 18 The problems associated with excessive TiM alerts. All quotes are from the Telehealth Nurse unless indicated. ........................................ 19
Appendix table 19 The problems associated with excessive TiM alerts. ........................................ 20
Appendix table 20 The future of the TiM. ........................................ 21
Appendix table 21 The Telehealth Nurse's attitudes towards how the TiM might be used in the future. ........................................ 22
Appendix table 22 How the TiM might be used by other teams ........................................ 23



For peer review only - http://bmjopen.bmj.com/site/about/guidelines.xhtml

## Appendix table 1 Participants' initial impressions of using the TiM app.

| Initial use of the app | |
|---|---|
| Easy to use | *"It's so easy to do; it literally takes five minutes from home." P317 [daily technology user]* |
| Quick to use | *"It's not a problem is it? It takes minutes, it's not like you have to sit there for half a day and do it, it literally, it's, it's sort of minutes." P248 [daily technology user]* |
| TiM accessible and acceptable to the elderly | *"So this lady [P217], she's an elderly lady who you thought wouldn't have embraced anything like this. But she has done and she, her and her husband both send it back; she's very disabled but able to use her hands quite well…they're both in their eighties; and she's a very good replier, sends it in." Telehealth nurse* |
| TiM accessible to those with significant disabilities | *"[There are] some are patients that, I'm surprised they took it up. I'm surprised; actually that, that, I never thought that he would use that… and he sends his back very, very well." Telehealth nurse* |
| Confidence the information would be held securely | *"I'm assuming that your department is one that's reasonably secure and has got reasonable standards… I can only make my judgement on the people I meet who are involved in it." C166* |

For peer review only

## Appendix table 2 The characteristics of those lacking confidence in technology user and their approach to using technology.

| **Characteristics of those with low technology confidence** | |
|---|---|
| Unsure if they would be able to use the TiM | *"…we're quite happy to deal with it, as long as it wasn't too techy" C062 [uses technology a few times a week]*<br>*"…as long as I can do it" P392 [uses technology daily]* |
| Lack of experience using technology | *"I don't use the internet for my job and it's all still paperwork orientated" C395 [uses technology daily]* |
| Difficulties with language | *"C: It's the system that's all. I don't catch on very well with it, that's ok. Like phones, I don't bother them really.*<br>*P: Couldn't read a text message.*<br>*C: couldn't do anything like that." C217 [never uses technology]* |
| Frightened they might make the TiM go wrong | *"Q: Have you used any of the other things that are on the tablet, so the education things or the website?*<br>*P: Oh I daren't touch all that stuff in case it goes wrong. (laughs)…I pressed that one day and I got a bit panicky (laughter) so I… left it…I just use it for the questionnaire now and that's it." P145 [uses technology daily]* |
| Frightened they might break it | *"…plus it doesn't belong to us and if we broke it we'd be devastated" C145 [uses technology daily]* |
| Frightened they will enter wrong information | *"My fingers are too heavy. … I'll end up phoning somebody." P378 [once a week technology user]* |
| Technology use is stressful | *"But I can… if I'm, if I'm taught …without aggravation." C380 [uses technology every few weeks]* |
| Problems using technology perceived as failure | *"I tried to order a book on it last week and I can't do it (laughs) it kept, I've lost passwords and all sorts…" C392 [uses technology daily]* |
| **Low user's approach to technology** | |
| Use technology for only a limited number of familiar purposes | *"I have used the computer for various things, and if I'm using it for something and I know what I'm doing, that's fine; I can get on for certain things now…" C380 [uses technology every few weeks]* |
| Avoids using unfamiliar technology | *"Q: And, and you say you were a little bit worried about the technical side?*<br>*C: Well I am, I was… I can manage things… I use Kindle and a laptop. Computers are a bit more of a thinker… (laughs) and…I don't do them all the time so I'm all right with basic stuff." C062 [uses technology a few times a week]*<br>*"I know basically how to navigate [his own device], whereas [the TiM] I don't." P145 [uses technology daily]* |
| Unable to problem solve: relies on others | *"Q: And what do you do if you get, get stuck on a problem?*<br>*C: Have to wait till me daughter-in-law comes up and get her to sort it out for me." C392 [uses technology daily]* |

## Appendix table 3 Negative attitudes to technology expressed by participants.

| **Negative attitudes towards technology** | |
|---|---|
| Lacking an interest in technology | *"It doesn't really interest me, if I'm being honest." C392 [uses technology daily]* |
| Not seeing a need for technology | *"I'm not against it but I don't want it and I can do without it and I don't need it." C166 [never uses technology]* |
| Feeling pressured to use technology | *"All the time we're being pressured [by people] that take all this marvellous technology up; and every day it is getting worse and worse, because it's getting beyond the control of the average person how to manage it properly." C166 [never uses technology]* |
| Feeling excluded/missing out if not using technology | *"I do feel…a slight social outcast, but I seem to be very much in the minority." C166 [never uses technology]* |
| Worried about the consequences of technology misuse | *"… certain things are very useful [but] once it gets to the mass market…it of gets exploded and misused. … You've got to the point of small businesses being blackmailed by hackers." C166 [never uses technology]* |
| Worried about online security | *"And as for banking online, God, it's a laugh, I would not touch it…if something goes wrong you have the devil's own job to try and put it right because there's no proof." C166 [never uses technology]* |
| Worried about intrusion into their privacy | *"I wouldn't have a Smartphone for a start, you know cos Big Brother's up there already." C166 [never uses technology]* |
| Previous negative experience using technology that was unreliable | *"…eventually these programs got more and more complicated and the time it was taking for sending information down and getting back was absolutely ludicrous; the thing would crash say at eleven o'clock on a Monday morning and you'd have to start all over again." C166 [never uses technology]* |
| Technology is an unwanted replacement for human contact | *"They can't pick up a phone normally anymore because they're so busy faffing about with their smart phones." C166 [never uses technology]* |

For peer review only

# Appendix table 4 Facilitators for TiM use.

| Facilitators for TiM use | |
|---|---|
| **Improving confidence using the TiM** | |
| Face to face training | *"I would take it up if I was shown that the program was spot on" C166* |
| Clear instructions | *"…That the instructions were aimed at an idiot like me… and not on the assumption that I would know what to do, cos half the instructions are based on the person knowing what they're gonna do, so it seems to me." C166* |
| Participants realising it's easy to use | *"I thought I wouldn't be able to do it (laughter) but I can… I'm not really… I don't want computer or anything like that, you know, so it's only that only because of that." C228* |
| Using it weekly to develop skills | *"I was quite happy using it weekly, cos things can change so quickly…I'm a terrible, I'd forget when I'd done it the last time," C392* |
| Enabling others to help them use it | *"He's got used to it as long as I set it up."   P217* |
| Seeing a purpose to use technology | *"It's unbelievable that sort of technology, but equally it's open to anybody to get into it. So, I might be persuaded eventually, but [laughs] it's a slow process" C166* |
| **Facilitators to use by those with disabilities** | |
| Stylus pen helped patients with upper limb disabled | *"Q: You mention you've got some problems with your hands. How do you find using your iPad?<br>P: It's a lot better since you gave me this [stylus] So I don't hold it like a button.  Other than that it's great.<br>Q: Have you got any other gadgets like that?<br>P: No. [to C] You ordered some more because you liked it didn't you?" P172 [daily technology user]* |
| Touch screen is accessible | *"It's fine. I mean it's getting more awkward to me, for me cos obviously my hands are [weak], but, it's quite easy.   Yeah, the touch screen, it's better than if you had to press this keyboard sort of thing." P056 [daily technology user]* |
| Use patients' own device | *"I think it'll probably have more of a take-up with people using their own device, because … I'm comfortable with my phone." P134 [daily technology user]* |
| Family/carers enter information on patients' behalf | *"Q: … is it something that you'd ask your carers to do or your family…?<br>P: Yeah, yeah, definitely.<br>Q: … would you have any concerns in them seeing your answers and that sort of thing?<br>P: No, I'd rather have the family do it rather than the carer do it, but I wouldn't have any problem, the family seeing it at all." P056 [daily technology user]* |

For peer review only

## Appendix table 5 Participants attitudes towards the frequency of TiM sessions.

| **TiM session frequency** | |
|---|---|
| Weekly sessions acceptable | *"Maybe once or twice a week would be enough." C063 (Frequent user)* |
| Weekly sessions make it part of routine | *"I'll keep it to Tuesday and do it every Tuesday while I'm working at home and I'll have peace and quiet." P122 (Excellent user)*<br>*"I was quite happy using it weekly, cos things can change so quickly…I'm a terrible, I'd forget when I'd done it the last time and … doing it maybe on the thirtieth of every month, I'd forget…" C392 (excellent user)* |
| Varying frequency according to speed of progression | *"Q: How often do you think you'd need to do it to make it worthwhile…?*<br>*P: Probably every month.*<br>*Q: …and if things changed how would you then approach it, would you go back and do it again or would you wait for the month…?*<br>*P: If things were changing quicker I'd go back in and do it again…" P423 (Excellent user)* |
| Carers may do it less often | *"Maybe carers don't need to do it quite as often but … maybe you would ask people how often they wanted to do it and maybe they would start at once a month and then as things progressed they did it a bit more often" C378 (control)* |

For peer review only

## Appendix table 6 Reasons for frequent and infrequent adherence to TiM identified during the interviews.

| | |
|---|---|
| **Reasons for missing individual sessions** | |
| Forgetting to use the TiM | *"I can't stand on my own so C has to bring it in here because I don't like to do it when he's working. So then I forget." P172 (Infrequent user)* |
| Patient and carer not doing it together | *"I'll do it and just leave it on the arm of the chair or something and say, "You need to do it". I don't know if she has on not." P122 (High user)* |
| Holidays | *"Oh dead easy; I know we do miss some weeks, but we've been, you know, if we, if we're away and we don't take it with us, we leave it here." P317 (High user)* |
| Acute illness | *"We just didn't do it, he weren't fit enough to do it." C228 (Frequent user)*<br>*"...when he was not very well he just couldn't be bothered." C366 (High user)* |
| Technical difficulties | See Table 6.1 & 6.2 |
| **Reasons for infrequent adherence** | |
| Poor health state at the start of the study | Reported in field notes made during calls with relatives. |
| Being too busy / having other priorities | *"It's just, with, with all the renovations and stuff it's sort of not become a priority at the moment." P134 (Infrequent user)* |
| Not receiving feedback and becoming demoralized with the TiM system | *"I have not received any feedback / contact from the STH MND team while using the TiM"* P047 |
| No current need for MND services so not seeing a benefit of TiM to their care | *"Well there'd be no point, would there?" P317 (Infrequent user)* |
| Unable to access the TiM tablet out without assistance | *"I can't stand on my own so C has to bring it in here because I don't like to do it when he's working. So then I forget." P172 (Infrequent user)* |
| **Facilitators of frequent adherence** | |
| Using TiM on the same day each week | *"We always, always remember that it's on a Thursday." P392 (Excellent user)* |
| Phone alarm reminders | *"Q: If it sends you an alarm reminder, would that be an annoyance...?*<br>*P: No, it would probably be more of a trigger for me to do it" P134 (Infrequent user)* |
| Family members reminding them | *"Our grand daughter rings us at about 7 o'clock to remind us. Then we finish up doing it on a Thursday morning." P217 (Moderate user)* |
| Being able to use TiM on their own device | *"If it worked on my phone there would be no issue whatsoever, cos I do everything on there; I shop… I can control lights, heating, everything on the phone. I think if TiM worked on there then you would get your responses every week." P134 (Infrequent user)* |

For peer review only

## Appendix table 7 Participants' expectations of the potential communication and monitoring benefits of the TiM service.

| **Potential benefits of the TiM identified by participants** | |
|---|---|
| **Improved communication with MND team** | |
| Increase speed of communication | *"If I put it on the tablet and I send it to you, you get it there and then. So if anything … happens to me in that period of time you know straightaway … So the quicker you can pick up on so, something it's better for you as a doctor as well as me as a patient." P091* |
| Increase frequency of communication | *"And you know somebody within the team is going to look at that information. So you are connected, once a week. So it's not every three months." C184* |
| Provides a direct connection with specialists | *"Well I'd read up about the telemed and I thought, well it gives you a direct connection with Sheffield and the team. I know it's an IT link but it's a definite link because once a week you are communicating with a team." C184* |
| Improve communication and liaison with other members of the MDT | *"Well I can't see there's a different way to what we're doing now; I'm quite surprised that you're all in communication really….I didn't know you spoke to [community MND team], and I didn't that [GP] and you and the hospital were quite so connected, so that seemed to be quite a positive thing." P354* |
| **Increased monitoring** | |
| To identify problems quickly | *"It just seemed a way of being able to communicate with, with my care team and letting them know on a regular basis how you're doing; and no doubt at the end of it (…) it flags up that I need help or I don't need help." P134* |
| To identify problems between clinic appointments | *"I thought it'd be a good idea for both of us, but mainly for P and, because it is a long time between the hospital appointments, and I thought well it's a good idea to monitor symptoms if they crop up out of the blue and we're not sure whether it's anything..." C062* |
| Enables a better understanding of the disease | *"When, when P started off with the home journey we, it was obviously dizzy spells…. So I said well we'll just keep a diary of what's happening so you yourself know what's happening, so that's what he's doing, and in effect, this is what, your telehealth could do eventually is sort of monitor people…" C091* |
| Enable self monitoring | *"So we can monitor ourselves as well at the same time. So it's helping you in the long run as well…I think you've gotta monitor yourself so you know how, what you can do and what you can't do. It's, you can't just rely on everybody on the end of a phone, pick up and say, you know, I've got this, that and the other. You've gotta look at health and safety for yourself as well, for whether you can walk or whether you can run or whether you can open a door or open a lid or carry a glass or whatever, you know, at the end of the day that's monitoring yourself, so you've gotta do it." P091* |



For peer review only - http://bmjopen.bmj.com/site/about/guidelines.xhtml

## Appendix table 8 Participants' expectations of the TiM service.

| Potential benefits of the TiM identified by participants | |
|---|---|
| **Psychological benefits** | |
| Clinicians take an interest | *"Somebody's taking an interest, somebody's wanting to know" C184* |
| Reducing isolation | *"You're not going to feel isolated." C184* |
| Reassurance | *"Sometimes it's just the fact that, "should somebody know about this?" or "should you be telling somebody that." Wouldn't it be nice if somebody knew this. Just small details that you sometimes think, "Does it make a difference if somebody knew about it?" And that telemed makes that difference." C184* |
| **Improve accessibility of the MND service** | |
| Reduce clinic appointments | *"If you can glean enough information of what I send through so I don't have to come to the hospital, I think that's a positive" P354* |
| Provide access to MDT when unable to travel to clinic | *" We just thought it would be quite good cos not everybody can get into clinic and thinking about the later stages, but it's still important to monitor how things are going" P480* |
| Reduce travel time | *"that would then cut down my travel time to clinic, you know, clinic doesn't really fulfil a great deal in my life other than ticking some boxes and sort of knowing where the progression's going but, you know, I think most of that now can be done through this server, through TiM." P134* |


For peer review only - http://bmjopen.bmj.com/site/about/guidelines.xhtml

# Appendix table 9 The acceptability of the TiM questions.

| **Acceptability of the TiM questions** | |
|---|---|
| Patients wanted the TiM to include questions about all aspects of MND | *"I've found the questions, I think they're all relevant. And, … it might be in the future when things do, if things do get worse it might be I might need those questions to highlight things and that's quite, quite comforting." P423*<br>*"There's one question about you use a feeding PEG. I don't at the minute so that's not relevant to me at the moment. But obviously as time goes by it, it will be. I think I just put, "no", I don't use one... But I think it should be on there because, … in time I probably will be using one." P145* |
| Emotional wellbeing questions were welcomed | *"I think it's important to find out, not just physically, how a disease affects somebody but how, emotionally, mentally it affects them. As a say, I'm quite a positive person, but, I have some off days." P122* |
| Carers happy sharing their sensitive information | *"Q: The sort of questions that they're asking you, quite, they're quite personal questions, aren't they...? How you feel about sharing those?*<br>*C: No problem." C392* |
| Questions made patients think about the consequences of their disease, but this was not a problem | *"Q: How does it feel having to sort of be faced with that question [about gastrostomy tubes]?*<br>*P: … when I first did that it was dead easy cos I just put "no" … and just moved on. But since then, since we've been to this Advance Care [planning] thing. …And that, that sort of brought it to the fore …. it's a thought that I wouldn't really wanna think about, but you've gotta think about, so I think on that one, is that something I shall maybe ask more to get more advice on." P248* |
| Reporting a deterioration on the TiM was not distressing | *"I think: am I getting worse? But, yes, am I getting worse…. I don't think any of the questions are really frightening. You know it might happen." P172*<br>*"in a sense it's good that you can change, you know, the, keep every, everybody up-to-date. Cos you don't know when things are going to change anyway, do we, we don't, you know, it's just, it's like a bit of a, a grey area isn't it? … as long as people who need to know see that information then it's … not waiting till your next visit at the clinic is it...?" P122* |
| Wanting questions that assessed how they were coping | *"Maybe if they say, "No, I've not been out for a fortnight", that it makes them think: well, actually why haven't I? And it would highlight that person's not getting out of the house and not doing something themselves and they are continually there. Do they need a bit more support?" C122* |
| Questions were repetitive | *" It's repetitive. … I pick it up and I think oh God, here we go again, same old, same old. [I] put a couple …more different ones in,… but no, the questions are right..." C145* |
| Questions become irrelevant if disability severe | *"As I am unable to use my arms and legs at all, many of the questions have become irrelevant to me beyond the first time of using it."* P047 |

For peer review only

## Appendix table 10 The validity / accuracy of the TiM questions.

| The validity/accuracy of the TiM questions | |
|---|---|
| Patients want answers to be accurate | *"If I've got a form I like to fill it in as accurately as possible." P248* |
| Patients were concerned that their answers would be misleading | *"I have lost weight [a few weeks ago] but I haven't [lost weight in the last week]. The way I'm reading it now… sounds like I'm still losing weight, well I'm not…I wouldn't like 'em to think looking at it, or you looking at it in Sheffield and thinking "God, he's still losing weight" P248* |
| The answers are insufficient to reflect small changes | *"Sometimes they answers to chose are too far apart so if the answer is one day a week, I answer no, or several, it's nearer several. So it's accurate but it's wide." P172*<br>*"The questions are quite in a narrow band, and because it's a slow-burner not much changes unless there's a step change, such as …the chest infection the other week." P354* |
| The condition varied from day to day making weekly assessments difficult | *"I thought: if I was doing the questionnaire on that day, "what sort of assistance do you get from your family? The answer would be off the scale somewhere. I thought: some of the questions don't quite fit the answer. So I try to put the most representative" C172*<br>*"I think some of the questions are a bit too general and wide rather than, for instance… what can I not do with my hand that I used to do, and how am I dealing with it." P166* |
| Informal care requirement questions were difficult to answer | *"The margins are too wide. One of the questions is, how much time do you spend in the day looking after her? And, there's a sort of, there's 3-4 hours. But I don't even look after myself for 3-4 hours, I just potter around. That's always gonna be the same answer." C172* |
| Some questions contradicted each other | *There are some questions on there that are bit ambiguous for me [for example] being able to use the stairs. Well I can't use the stairs but I still have a bathroom upstairs so I still walk from the top of the stairs to our bedroom." P134* |
| Patients wanted to provide more information to clarify their answers | *"… if you have any more questions to ask you've got that… availability to ask [the MND team] if you have any problems…" P056*<br>*"If you want to ask anything maybe you could type it in instead of it just being yes/no" CaT Patient 317*<br>*"I think that, that would be a good addition. Because you, you could answer a question and at the end of the section just put in, and just expand on what, why you've answered that, if you see what I mean? I think a comments box would be a good idea." P122* |
| Same problems identified every week | *"It's a probably the same answer I give every week… It says; "Do you stumble or feel that you fall, or have you fallen?" And I do, every single week, I guarantee that" P122* |
| Answer affected by other health problems | *"So at the minute I'm slightly doctoring the answer; so I'm saying I can dress myself but I can't, but it's not the MND." P381* |

For peer review only

# Appendix table 11 Participants understanding of the TiM service.

| | |
|---|---|
| **Aims of the TiM service:** | |
| Monitoring | *"For you to be aware of it, and monitor it..." P402* |
| Relay information to the MND team | *"It shows how you're feeling which gets then relays to the nursing staff. If you've any questions you've got the item on it where you can ... ask that as well... I would think... it's read, isn't it, and then probably recorded somewhere." P056* |
| Look for trends | *"I would say they are probably going on to some sort of graph that detects a trend. I mean with me, you wont get a trend because sometimes you are up and sometimes I'm down. It'll look like a [gestured up and down]. But I would imagine the ideal situation is when you can detect a trend but that does depend on what's happening." C172* |
| Provides trends and alerts to problems | *"I'm guessing that it comes to someone's desk and that they're able to see a chart... from week to week, and I would imagine that there would be a series of red flags for you or some traffic light system... that red's intervention required, amber's a warning, green's get on with it.." P134* |
| Prioritise patients in need | *"I could see the reason why you were doing it; I realised that all the data was going to be collated and you can see then at a glance... you can see the statistics and everything, and it would highlight to you... if I had a dramatic change." P317* |
| Data would be used for research | *"I didn't realise that that's what was going to happen, I thought it was just being used for research." C392*<br>*"I would assume it goes to some databank somewhere and they try and correlate the answers I've given with, perhaps somebody else ... to see if there is any common ground."P122*<br>*"I imagine it goes onto a database ... and then if yourself and [Professor] are ever doing any research they may be able to...or if there's any trials you might be able to select from that who would be suitable for that trial." P 317* |
| Both clinical care and research | *"I imagine it goes on a survey of all the people you're doing to compare us with each other, and also to follow my own progress." P166* |
| **The role of the Telehealth Nurse** | |
| TiM Nurse looked at data | *"It shows how you're feeling which gets then relays to the nursing staff, doesn't it." P056* |
| EH was looking at data | *"I didn't realise that [Telehealth Nurse] was involved and she would ring us if our answers drastically changed, cos obviously they've stayed very much the same. Then I'm quite encouraged by that, I just presume that you do it yourself...and if you'd got a problem you'd ring [Telehealth Nurse] up for a bit of help." C392* |
| Unsure who looked at data | *"I'm guessing that it comes to someone's desk and that they're able to see a chart" P134*<br>*"The ones that have spoke about it to me I don't think they realise that I'm seeing their responses." Telehealth Nurse* |



For peer review only - http://bmjopen.bmj.com/site/about/guidelines.xhtml

## Appendix table 12 Nurses' experience of using the TiM clinical portal. All quotes are from the Telehealth Nurse.

| **Using the clinical portal** | |
|---|---|
| Easy to use | *"The training that I've had from seeing it this side was brief from Esther and it's, it's never, it's not difficult so it, I find I, right from the beginning I found logging on has been the most difficult thing, so; but once you're on finding your way around it is very, very easy, very easy,"* |
| Low burden | *"It only takes minutes"* |
| Easy to understand the flag system | *"If everything's OK it's green, … [if] there's maybe some elements that might need to be looked at, it's an orange or yellow, and then if there's an alert it's a red one."* |
| IT support for passwords required | *"My main problem was me accessing it to begin with, and that was very problematic. It's just asked me to change me password."* |
| Nurse didn't know how to use the patient TiM app or resolve technical problems | *" N: I have had a couple of phone calls, cos [the participants have] got my contact number through this, and they've rung with a problem, usually a logging on problem and…*<br>*Q: Forgotten their password?*<br>*N: Yeah…I've asked Esther, to be honest, because I'm not familiar with their device."* |

For peer review only

## Appendix table 13 The accuracy and sensitive of the TiM information. Quotes are from the Telehealth Nurse (N) unless otherwise stated.

| **Accuracy of the TiM information** | |
|---|---|
| Information accurate to help make some decisions | *"N: [EH] has led me to believe that it's quite easy to red flag on the carer. So this chap is triggering ... [reads the PHQ4 questions] So I mean ... the four that are triggering, they're all about...<br>Q: Worry, stress?<br>N: Yeah, thoughts: yeah. I know how poorly this lady is, I know how disabled she is, and I do know him, and when you speak to him he's very blasé about it all, which actually is more of a worry."* |
| Information in TiM wasn't sufficiently detailed enough to be sensitive | *"N: ...I think if they're routinely sending us an update every week I think you're more likely to pick up on problems, but I don't... I thought that would be so.<br>Q: Is that, do you think that's happened?<br>N: No not really. I don't know that the questions are sensitive enough, and I suppose the thing is that it might, if it triggers a contact phone call then you may well pick up on things sooner." (early interview)* |
| Needed more information | *"N: It's almost like you need a two-way thing (laughs) you need to ask them a question, [for example] how long have you been coughing... are you bringing anything up when you're coughing... you know, that type of thing... have you had a temperature... it's almost like [laughs] you need a two-way communication, cos this is just a snapshot, isn't it..."* |
| Discussions are required to fully understand a problem | *"I think some of the problems with patients using NIV, very specific problems, you only pick up from a conversation with them. I don't think you pick it up on the Tele, cos it's [the way] it's structured, it might show a problem but not a specific problem."* |
| An additional comment box would help gain more detailed information | *"Q: Yeah. Do you have, would you have time to read the comment boxes?<br>TW: I would hope so. You'd have to make time. If it was a problem that was coming up all the time, yes, I would hope that that; in some ways that might save you time."* |

For peer review only

## Appendix table 14 Participants' experiences of how the TiM impacted on their care.

| **Impact on if TiM on patient care** | |
|---|---|
| Improves knowledge | *"Because since I've done it I'm taking more notice of my weight but I didn't before so I know it very carefully. It made me aware of that." P172* |
| Close monitoring | *"I'm certainly not left alone for more than a month, ever…Sheffield is there if I really need them, but on the other hand [local team] is here." P166* |
| Reassurance to know being monitored | *"I think the benefit to P is real. Because... somebody is there on hand looking at things... Because it's slow with P and he doesn't need as much attention and care, it's easy to feel detached from any positive interaction." C122* |
| Keeping in touch with specialist | *"…it's knowing that somebody else is in your corner." C423<br>Q: Do you think there might have been a point in your disease where those questions …were useful?<br>P: Nearer the beginning, definitely. I think, and I can only guess, that it must be the same for lots of long-term conditions; you know, I've lived with this for over five years now… you get to understand your own body and you know it's, it's normal for me now and for the first year no day was normal, no day looked like any other day in my life before that. So… if I could have camped in [MND consultant]'s house for the first six months I would have done, just so she was there, so I could say, but what about this, what about that; and you imagine symptoms, or I did, you know, you think God, this is happening and that must be related to the MND... So in the first year I would have filled that in every day, just to have that touch point," P047* |
| Nurse giving advice | *"When I came back on Tuesday last week and I did the second questionnaire, and within a day [Telehealth Nurse] saying "I've got a red flag on one of your answers." And it's the fact that I'd fallen twice while I was away on holiday and I'd put on it, you know: it said "have you fallen recently, how many times?" and I'd fallen twice while I was on holiday. So she phoned me, and said, "Are you ok? Is there a reason why you fell?" No, just my usual clumsiness....<br>Q: Were you expecting her to call?<br>P: No, I wasn't actually. It was just a bolt out of the view...I find that quite positive. It shows that the whole idea of it works.<br>Q: Has it changed your behaviour at all?<br>P: No. Not really." P122* |
| Nurse identifying problems | *"Q: [Telehealth Nurse] called, I think she spoke to you about when you fell...<br>P: Mm.<br>Q: ...what did you think about that when that happened?<br>P: It was useful wasn't it?<br>C: Well you weren't in so she spoke to me.<br>P: Yes, because I'd tripped over the bedroom chair...<br>C: that's right and, yes, that was the main thing, that's right, yeah. No, it was interesting that that, that had been picked up because we weren't, we don't know how it was picked up." P&C166* |
| Identifying problems between clinic visits | *"When I filled it in last week, and within a day [Telehealth Nurse] was phoning me. How much better could you have that? Instead of, two months down the line and I attend the clinic and they say "how have you been, have you fallen?" and I say "oh yeah I did: two months ago". See where I'm coming from? You've got that instantaneous contact with this technology that perhaps you don't have without it. So I'm a great believer in that, I'm a great believer in technology." P122* |
| Supporting important decision making | *"Q: The other question I had was that we kept an eye on your weight and I wonder whether you think that may have influenced your decision to have a feeding tube or not; do you think it, do you think it had?<br>P: [writing] I was frightened by the speed of loss of weight but was convinced how much muscle I lost." P409* |
| Help accept the disease | *" The questions nudged me to facing what I could do and not what I can't." P409* |

For peer review only

# Appendix table 15 The Telehealth Nurse's attitudes towards the value of the TiM.

Quotes are all from the Telehealth Nurse

| **The Telehealth Nurses' attitudes towards the value of the TiM** | |
|---|---|
| TiM could be valuable | *"As a tool to aid the patient and then aid the nurse"* |
| To identify problems between clinic visits | *"A number of patients with MND and their carers will wait till a clinic, instead of contacting us with, if they're, they're worried about something, or there's a change in their, you know, their condition, and I think if they're routinely sending us an update every week I think you're more likely to pick up on problems."* |
| Alerting to problems earlier | *"Q: So it could have a use maybe in alerting you to earlier need for intervention?*<br>*N: Yeah, yeah."* |
| Help monitoring trends | *"I mean the weights have been quite interesting, cos if they can use the weighing scales … that's been quite interesting, so we can monitor their weight…. cos otherwise you wouldn't necessarily see that variation."* |
| TiM wouldn't have a negative impact on the service | *"Q: do you think a patient will be concerned this is trying to take away a part of the service, or do you think the clinical team might feel it's taking away part of the service?*<br>*N: I don't think either side would. From a clinical side I think that anybody would be willing to make it as easy for the patient as possible."* |
| Clinics are a burden to patient | *"I think from the patient's point of view I think it becomes very burdensome, the travel into clinic, very much so…But certainly the, the travel and the amount of time and effort for them to come to clinic to sit in clinic to then go home again, it's very difficult for them."* |
| Patients do not see value in attending clinic | *"… [patients] sometimes say;* "nothing, I don't get anything out of coming to clinic because you're reiterating the same things, I know I'm getting worse…" *So there are some patients that don't see the value of coming to clinic anyway. Now whether they would use a system like this and see the value of that I don't know."* |
| TiM could allow patients to be managed remotely | *" I think it could. I think it's one of those difficult things that at the moment, because it's not something we systematically do. And they are attending clinic or I have connections with their community care team, so I am in touch with what's happening and things are getting monitored. I don't know."* |
| Some problems need face to face assessment | *"Q: How reliable this kind of technology would be in deciding when or, when to cancel an appointment for a patient?*<br>*A: Oh I don't know, I don't know.*<br>*Q: Would you feel secure looking at that saying that patient doesn't need to come in?*<br>*A: I think, I think it depends, so when I look at, if it's a breathing problem and if I didn't know them and they were having problems with their breathing …that would alert me and I would [think]: right we need… to contact, we need to be looking at this and monitoring it…I think it depends."* |



For peer review only - http://bmjopen.bmj.com/site/about/guidelines.xhtml

## Appendix table 16 Participants experiences of clinician feedback.

| To act upon problems and provide feedback | |
|---|---|
| Feedback reinforces the benefit of the TiM | *"I find that quite positive. It shows that the whole idea of it works." P122* |
| Not receiving feedback is demoralising | *"The emotional psychological depths that I've been to, I was putting the stuff in Telehealth and thinking but nobody's acknowledged this or contacted me about it, and I thought: well they're not going to because that isn't what the clinic's about, and that made me stop using it." P047*<br>*"…it's like all forms and all surveys… they say "your opinion is important please fill in the following…" and you say something that you think is absolutely dramatic and mind blowing and nobody comes back to you on it. And you think: well how important is that survey?" C172* |
| To provide feedback even if nothing had changed | *"Q: And would you expect perhaps someone to tell you what's going on with the tablet or the answers?*<br>*P: Yes. Because if I hadn't changed much, I would have thought I would have had some feedback."P172* |
| To acknowledge problems even if nothing can be done | *"[falls] … knocks your confidence …I probably were putting too much onus on Sheffield Hallam because (laughs) we've got this and there's not jack shit they can really do about this and we know that...*<br>*Q: But that kind of acknowledgement's quite important, do you think, of the, what happens …?*<br>*C: I do, yeah, it's a bit of support, in't it, it's knowing that somebody else is in your corner." C423* |
| MND team couldn't solve their problem | *"Q: If your answers changed what do you expect of the MND service?*<br>*P: I don't, to be honest. Let's say if I thought I'd got a problem I wouldn't necessarily come to you because I don't think, I think, you know, I, the impression that it's medical to do with P, and if I have got a problem really; because when you flash up it does say if you, is to contact the, there is, so I would probably, I mean that's not part of your remit, is it, me really if I've got a problem?" C381*<br>*"[emotional support] That's not what that clinic's about, that clinic's about physical wellbeing and physical health… truthfully I thought I don't know why these questions are in here, because that clinic isn't equipped to deal with that." P047* |
| Feedback thought to be pointless if nothing can be done | *"But then again, what's the point of coming back if you can't say anything?" C172* |



For peer review only - http://bmjopen.bmj.com/site/about/guidelines.xhtml

## Appendix table 17 The mismatch between the participants' and nurses' expectations of the TiM service.

| | |
|---|---|
| **Mismatch between patient and nurse expectations** | |
| Patients expected contact if they experienced important problems: | |
| Falls<br>Chest infections<br>Dysphagia | *"Q: ...do you, do you think they're important things that she needs to know about?*<br>*P: I would say yeah… cos one of the questions is have you fallen or tripped? Obviously if I am falling more often, (well I am, I fell a few weeks ago, didn't I? but that, that's first time in months...). If I was falling … over every week I would say that, yeah, she needs to know something like that, or if I'm starting to not eat me food properly, if I'm starting to cough, I think obviously she needs to know that" P145* |
| Emotional difficulties | *"Most of my problems at the time are emotional, and the TiM does not appear to trigger any intervention." P047* |
| Problems may not be seen as important to nurse | *"She red flagged that she'd fallen, which is quite a common occurrence on a lot of patients, and I don't particularly worry unless they've been very, very well and then suddenly." Telehealth Nurse* |
| Problems may not be seen as important to patients | *"Q: When you put that you had fallen, what were you expecting to happen?*<br>*P : No. No.*<br>*Q: And when you heard that [your OT] had heard about it, what did you think about that?*<br>*P: Nothing really.*<br>*Q: And, do you think in the future, if something were to happen to you, and you put on here, what would you expect to happen, what would you like to happen?*<br>*P: I would think [Telehealth nurse] would ring. But the only thing is the over balancing, so far, isn't it." C217* |
| Problem may not be seen to be under the MND team's remit | *"[emotional support is] not what that clinic's about, that clinic's about physical wellbeing and physical health. To me, and in my own head, I thought it's never been about psychological wellbeing and your mental wellness and therefore; and truthfully I thought I don't know why these questions are in here, because that clinic isn't equipped to deal with that." P047* |
| Participants expecting contact when stable | *" It would be nice for someone to phone occasionally … particularly if anything changes.: C172* |
| Not expecting a particular action: expect nurse to decide | *"Q: And what would you expect to happen if you did change your answers like that week?*<br>*P: Not really, no, cos you know more than I do." P056*<br>*"I would tend to leave it to them to solve anything that needed solving." C217* |


For peer review only - http://bmjopen.bmj.com/site/about/guidelines.xhtml

## Appendix table 18 The problems associated with excessive TiM alerts. All quotes are from the Telehealth Nurse unless indicated.

| **Excessive TiM system alerts** | |
|---|---|
| The same alerts appear every week | *"We have some of our patients that do this every time… They ... cough or choke at least once a week."* |
| Alerts increase the time required to use the system and can cause frustration | *"Then when it was coming up every week… I knew I'd spoken to them about that [problem] and that was their choice. And I'd kept putting [a comment in the TiM notes] but you're sorta thinking; why do I have to keep putting it on every time?"* |
| Nurse can't control the alerts | *"If you know somebody's got a problem and they're not really trying to do anything about it, then you know that [alert is] gonna keep coming back every week; and I haven't been putting comments on all the time."* |
| Nurses may appear to not be acting on potentially dangerous problems | [1]*"[reads]* "Do you ever cough or choke on food?"*And then he's put:* "Occasionally", *and that's fine."* |
| Problems flagged even though the patient has chosen not to medical advice to avoid the problem | *"cos I know that patient and I know that they've chosen to eat and that it is problematic. But they've got a feeding tube and they should really be using their feeding tube, but they're [also eating]. Then when it was coming up every week, I knew I'd spoken to them about that and that was that choice?"* |
| Reporting the same problems without solutions could be demoralising to patients | *"Maybe to them the fact that it's the same thing week in week out, they've got an insight into that problem, it's not changing and, so they're not looking for something to help with it really."* |
| Repeated problems on which no action could be taken should be paused to avoid excessive alerts | *"Q: Are there any other, other things that you'd change at the moment to make it, to improve the system?*<br>*TW: Apart from the, the same red flags coming up every week with the same problem, having some way of either taking them off or say, or putting a comment in that, that would take it off by saying you've addressed this problem, it's, it's still gonna be there, it's not gonna change."* |



For peer review only - http://bmjopen.bmj.com/site/about/guidelines.xhtml

## Appendix table 19 The problems associated with excessive TiM alerts.

All quotes are from the Telehealth Nurse.

| Nurse reaction to alerts | |
|---|---|
| Calls patient for more information | *"[I] ring the patient about it and ask them"* |
| Liaises with the community team to get more information | *"I try and get a bit more information, cos the, the community team may have been out and seen them and seen them face-to-face and have got something a little bit more useful back; so I have used it for that circumstance. And I'm in touch with the community team quite often, so sometimes if I'm ringing about something else I'll ask about one of the patients that's on here."* |
| Prefers to wait until clinic | *"I've seen somebody that was red flagged, cos they're coughing more..I've put a [TiM note] that we'll review in clinic next week."* |
| Will chose not respond to problems she thought were common in MND | *"She red flagged that she'd fallen, which is quite a common occurrence on a lot of patients. I don't particularly worry unless they've been very, very well and then suddenly. So if it happens over a few weeks and I've spoken to them and I know the situation…I'm not always alerted by that red flag."* |
| Using all the TiM information on the to make a decision | *"They were having problems with their breathing but I would look: are they on a breathing machine? No. That would alert me. So I think it depends."* |
| Patients and nurses prioritise different problems | *"I did the second questionnaire, and within a day the [Telehealth Nurse] calls saying "I've got a red flag on one of your answers." And it's the fact that I'd fallen twice while I was away on holiday… she phoned me, and said, "Are you ok? Is there a reason why you fell?" No, just my usual clumsiness….*<br>*Q: Were you expecting her to call?*<br>*P: No, I wasn't...It was just a bolt out of the view… I find that quite positive. It shows that the whole idea of it works." P122* |
| Having a relationship with the patient made it easier for the nurse to call the patient | *"I think it helps me that I know the patients. So I know this chap very, very well, I have a relationship with him and his wife; ringing up's quite easy to do, they wouldn't, they wouldn't be fazed by that. My response would be;* "oh there's been a red flag on the Telemedicine that you sent through, a message that you've sent through, and that's why I'm ringing"*. And that was quite an easy one."* |
| Harder to ring those who she didn't know | *"There are a small number of patients that I don't know, it just so happens that they've been very well through, so there hasn't really been any major alerts."* |
| Ringing carers may be difficult | *"If we've had a carer's response that's red flagging I don't always find that an easy phone call to make to a carer who's struggling."* |
| Patients not expecting her call | *"I rang a lady who I didn't know and she red flagged that she'd fallen… It was the lady's husband … he was quite shocked that I'd rung cos I didn't know them and I just explained about it, and he just said "No, it was just a little trip, she's absolutely fine, no problem."* |
| Nurse doesn't feel her calls benefit patient | *"I've said "Oh you've had some problem with this?...I know because you sent a Telemedicine and I'm the one that looks at the problems and sees what's what." And they said "Oh right, oh yes, it's nothing, it's fine." I don't know…"* |


For peer review only - http://bmjopen.bmj.com/site/about/guidelines.xhtml

# Appendix table 20 The future of the TiM.

| Preferred way to communicate with MND team | |
|---|---|
| Modes of communication depends on the individual | *Q: How do you think is the best way for people contact you?<br>P: I think for me it'd be by phone, but again that will be depending on the carer and my speech. I mean I'm currently OK with the phone.<br>Q: And how do you feel about email contact or contact through the Telehealth?<br>P: Again that's fine by me, I use email, computer all the time, but again you'd have to judge that on the individual." P381* |
| Contact needs to be convenient | *"And phoning you up, I don't know about you but I hate phone calls: you are just settling down to have a nice cup of tea or a chocolate biscuit or whatever and the phone rings, you have someone trying to persuade you to change your heating systems" C172* |
| Alternative methods of communication add flexibility | *"I'm really happy …communicating by email, because for me I can do it then in my own time, because I haven't got use of my hands and arms; so if I know that there's an email that I can read and then reply to in my own time, that's far more relaxed for me actually than the telephone… I can speak fine but the telephone, somebody has to hold it for me…if I'm not in exactly the right position it's not comfortable to take a r." P047* |
| Telephone may be uncomfortable for some | *"I feel a bit awkward on the phone, and you can't get across how you're really feeling on a phone anyway..I tend to get nervous when I'm on the phone and stuff and I forget what I'm saying to people." P423* |
| Dysarthria makes telephone hard | *C: [to P] Well telephone calls aren't that practical are they for you. Because if you are tired, I mean it's difficult to convey."C172* |
| Email may be impersonal | *"I think that's a bit impersonal email, you know, that, that's what I thought" C423* |
| Telephone useful for problems needing an immediate answer | *"If it's something that needs doing, dealing with here and now: the telephone, email is, is my favourite way of communicating, because it suits my condition.." P047* |
| Some subjects are better discussed face to face | *"I think it's not personal… I'd rather see somebody or talk to somebody than, than read about it on something on a screen." C366* |
| Happy to receive feedback in clinic | *" I realise that you are probably busy people, so I'm quite happy for someone to say, when we're down there, "oh by the way, your survey has altered and, you know, do you want to talk to you about it." C172* |
| Happy to wait for a reply as long as their message had been acknowledged | *Q: And if you put in a question… How long would you expect before you got an answer?<br>P: Well normally [Telehealth Nurse] gets back to me within a day or so. So I would expect two days, almost maximum.<br>Q: OK. So, reasonably quickly.<br>P: Even if, it was: "we've got your question :we're thinking about it"<br>Q: So, just an acknowledgement that you've email?<br>P: Yeah. It may be [Telehealth Nurse] can't answer me first time. Like the citalopram. What she said was: "I'll discuss it with, (I think) you" and then came back to me." P381* |
| Importance of respiratory monitoring | *"Q: What do you think, how do you think this would fit in around your clinic visits and the support you would get from [Telehealth Nurse]? If this was to become…rather than a research project, part of standard care, how do you think you would suggest using it?<br>P: Well I think in my case I would be happy to use that and lengthen the time between visits. I mean, the only difference to me is the breathing test." P381* |
| Happy to share the information with other members of the care team | *"Q: And is there, is there someone locally that you think would be, so that would be able to see your answers …?<br>P: Well a good one would be the GP, wouldn't they? Or even the hospice, or, even the district nurses, probably more the district nurses, cos obviously they've got a regular visit now, haven't they?" P056* |



For peer review only - http://bmjopen.bmj.com/site/about/guidelines.xhtml

## Appendix table 21 The Telehealth Nurse's attitudes towards how the TiM might be used in the future.

Quotes are all from the Telehealth Nurse

| **How would the TiM be used?** | |
|---|---|
| Delegating TiM duties may not be possible | *"I agreed to do it originally cos I said the thing is when a red flag comes if it's somebody else doing it and looking at it they're gonna come to me."* |
| The nurse wants to use her judgement, not follow a strict protocol | *"The way this study is run at the moment I can respond in, in the way I think is appropriate" Telehealth Nurse*<br>*"if it was written down, [that] I had to ring and I had to ring straightaway, ... I don't know whether, I would have found that quite difficult not being able to use my initiative and how I'm familiar with the patients and, don't know I might not have, I might have found that a bit more difficult."* |
| Some nurse may be more willing to follow a protocol and call more often | *"N: It's difficult, I think you would probably get more useful information out of the research nurses, but you would have to have a system where they'd be able to go to somebody to act upon what was [needed]" Telehealth Nurse*<br>*"I think if it was a bigger study and.. it was a very ... carefully monitored study, I think maybe the person doing this, looking at this ... looking at what the replies have been, if they had to contact them that might work better."* |



For peer review only - http://bmjopen.bmj.com/site/about/guidelines.xhtml

## Appendix table 22 How the TiM might be used by other teams

| | |
|---|---|
| **Use by community teams** | |
| Community teams would need capacity | *"I think if they have the capacity it would be useful," Telehealth Nurse* |
| It would increase information available for community teams | *"Q: Could [you] see [the TiM] fitting into the role you play or whether you think it's not very helpful?<br>N: I think it probably could because, like you've just said.. that [patient has] reported on here that she's … coughing/choking so many times but she never said that to me." Community nurse* |
| Community staff may not have access to computers | *"I'm not very good at computers. I have to say, that is the thing I find most stressful about working here. You I'm home based. I go on NHS website...so I'm not on, well I can get onto it but I do not cos I don't spend a lot of time [in the hospital]."Community nurse* |
| Community staff happy to receive information from Telehealth Nurse | *"Q: If someone else was looking at this, telling you there's a problem?<br>N: Yeah, no, that would be fine, that would be fine, yeah, yeah."Community nurse* |
| **Use by other MND centres** | |
| Other services would respond positively | *"I think their gut reaction is that that sounds something potentially helpful to us; I haven't really had any negativity" Telehealth Nurse* |
| Other services would want TiM if it saved them time | *"I think that if it saves them time I think that they would definitely embrace it." Telehealth Nurse* |
| Other services would want TiM if it benefited the patient | *"From a clinical side I think that anybody would be willing to make it as easy for the patient as possible, as long as it was useful." Telehealth Nurse* |
| Nurses would use it if it were part of their usual role | *"Q: But then if you were thinking about implementing this into the NHS who would you get the most useful information from?<br>TW: I think if it was implemented and it was part of somebody's role every day they would just do it, they would do it automatically; and, yes, I could envision that it would be very useful. So I suppose I'm a bit, it's half and half." Telehealth Nurse* |


For peer review only - http://bmjopen.bmj.com/site/about/guidelines.xhtml

BMJ Open

# A process evaluation and exploration of telehealth in motor neurone disease in a UK specialist centre



| | |
|---|---|
| Journal: | *BMJ Open* |
| Manuscript ID | bmjopen-2018-028526.R2 |
| Article Type: | Original research |
| Date Submitted by the Author: | 10-Aug-2019 |



| | |
|---|---|
| Complete List of Authors: | Hobson, Esther; University of Sheffield, Sheffield Institute for Translational Neuroscience; Sheffield Teaching Hospitals NHS Trust, Department of neurology<br>Baird, Wendy; University of Sheffield, School of Health and Related Research<br>Bradburn, Mike; University of Sheffield, Clinical Trials Research Unit, School of Health and Related Research<br>Cooper, Cindy; University of Sheffield, Clinical Trials Research Unit, School of Health and Related Research<br>Mawson, Susan; University of Sheffield, School of Health and Related Research<br>Quinn, Ann; Sheffield Motor Neurone Disease Association Research Advisory Group, SITraN<br>Shaw, Pamela; University of Sheffield, Sheffield Institute for Translational Neuroscience; Sheffield Teaching Hospitals NHS Trust, Department of neurology<br>Walsh, Theresa; University of Sheffield, Sheffield Institute for Translational Neuroscience; Sheffield Teaching Hospitals NHS Trust, Department of neurology<br>McDermott, Christopher; University of Sheffield, Sheffield Institute for Translational Neuroscience; Sheffield Teaching Hospitals NHS Trust, Department of neurology |



| | |
|---|---|
| <b>Primary Subject Heading</b>: | Neurology |
| Secondary Subject Heading: | Health services research, Palliative care, Patient-centred medicine |
| Keywords: | Telemedicine < BIOTECHNOLOGY & BIOINFORMATICS, Motor neurone disease < NEUROLOGY, Organisation of health services < HEALTH SERVICES ADMINISTRATION & MANAGEMENT, Adult palliative care < PALLIATIVE CARE |
| | |


SCHOLARONE™
Manuscripts

# A process evaluation and exploration of telehealth in motor neurone disease in a UK specialist centre

*Dr Esther V Hobson[1,2], Professor Wendy O Baird[2], Mr M Bradburn[2,3], Professor Cindy L Cooper[2,3], Professor Susan Mawson[2, 4], Mrs Ann Quinn[5], Professor Pamela J Shaw[1], Sister Theresa Walsh[1], Professor Christopher J McDermott[1]

*corresponding author

1 Sheffield Institute for Translational Neuroscience, University of Sheffield (SITraN), Sheffield, UK, 2 School of Health and Related Research, University of Sheffield (ScHARR), Sheffield, UK, 3 Clinical Trials Research Unit, School of Health and Related Research, University of Sheffield, Sheffield, UK, 4 NIHR CLAHRC Yorkshire and Humber, UK, 5 Sheffield Motor Neuron Disease Research Advisory Group, Sheffield, UK.

**Author email addresses**
e.hobson@sheffield.ac.uk
w.o.baird@sheffield.ac.uk
m.bradburn@sheffield.ac.uk
c.l.cooper@sheffield.ac.uk
s.mawson@sheffield.ac.uk
Ann Quinn: c/o e.hobson@sheffield.ac.uk
pamela.shaw@sheffield.ac.uk
theresa.walsh@sheffield.ac.uk
c.j.mcdermott@sheffield.ac.uk

**Keywords**
Telehealth, telemedicine, motor neuron disease, amyotrophic lateral sclerosis, qualitative, process evaluation

**Word count**
6894

For peer review only

## ABSTRACT

### Objectives
To evaluate the processes involved in using a novel digitally enabled healthcare system (TiM: Telehealth in Motor neuron disease) in people living with motor neuron disease (MND) and their informal carers. We examined TiM implementation, potential mechanisms of impact and contextual factors that might influence TiM implementation or impact.

### Design
An 18-month, single-centre process evaluation within a randomised, pilot and feasibility study.

### Intervention
TiM telehealth plus usual care vs. usual care.

### Setting
A specialist UK MND care centre.

### Participants
40 patients with MND and 37 primary informal carers

### Primary and secondary outcome measures
Patient, carer and staff outcomes and experiences using semi-structured interviews. Descriptive data on implementation and use of TiM telehealth.

### Results
The TiM was acceptable and accessible to patients, carers and staff. Intervention uptake and adherence were good: 14 (70%) patients completed a TiM session at least fortnightly. Barriers to TiM use (such as technology experience and disability) were overcome with well-designed technology and face-to-face training. Reported potential benefits of TiM included improved communication and care coordination, reassurance, identification of complications and the potential for TiM to be an alternative or addition to clinic. Benefits depended on patients' current level of needs or disability.

The main challenges were the large number of alerts that were generated by TiM, how the clinicians responded to these alerts and the mismatch between patient/carer expectations and nurses actions. This could be improved by better communication systems and adjusting the alerts algorithm.

### Conclusions
TiM has the potential to facilitate access to specialist care but further iterative developments to the intervention and process evaluations of the TiM in different services are required.

For peer review only

## ARTICLE SUMMARY

### Strengths and limitations of the study

- This is a detailed evaluation of a digitally-enabled care system that aims to provide accessible, specialist holistic care to patients with motor neuron disease and their carers.
- The use of mixed-methods provided an in-depth understanding of the processes involved in the new care service, the potential value of TiM and factors which are likely to influence its use, implementation and success.
- This was a small, single centre study using only one nurse to deliver the intervention.

## BACKGROUND

Motor neuron disease (MND) causes progressive muscle paralysis, severe disability and eventually death within on average, two to four years (1). Attendance at a specialist multidisciplinary clinics (MDC) is associated with improved survival and use of assistive aids, devices and proven therapies (2-7). Patients face many barriers accessing specialist services meaning many patients fail to gain the full benefit from these services (8). There is some evidence that digital-enabled technologies can improve access to specialist care (9). Video-consultation (telemedicine) is used in some centres and appears to be feasible and acceptable (10-14). However, between consultations changes in patients' condition may not be recognised. There are several technologies in development that can frequently record aspects of MND progress (telehealth) such as speech or hand function. They have predominately been developed for research but telehealth could be used for clinical care purposes (15-18). No digital system has yet been evaluated that can monitor the full range of problems faced by patients with MND in order to detect and act upon issues that arise in a timely manner.

There also remains a need to develop interventions to support carers of those with MND. Medical interventions such as non-invasive ventilation and gastrostomy feeding require carers to conduct complex tasks with little training (19). Carers report that these interventions are associated with increased physical strain and carer time (20-22). Provision of disease specific information and signposting to external support services have been identified as helpful (23,24) and whilst one small study of intensive case management reported positive feedback (25), another showed no improvement in carer strain (26).

We employed a user-centred design process to develop the TiM telehealth system (Telehealth in MND) (27). This allows patients and carers to send information about their condition to the specialist care team (27). Early testing has already been conducted with a small number of potential users but further testing in the clinical environment was required (27). Telehealth services are complex interventions, consisting of different component parts and whose success relies on the context and individual behaviours of those using and delivering the intervention (28). Clinical trials of complex interventions such as telehealth face problems with patient and staff engagement and often lack of



For peer review only - http://bmjopen.bmj.com/site/about/guidelines.xhtml

understanding of how the intervention is used, what works, and for whom (29-31). People living with MND and their carers face additional barriers to using digitally-enabled care due to their disability and age. The complexity of the disease and the holistic aims of care in this terminal disease mean telehealth systems will need to differ from more simplistic, target-driven care systems (such as blood pressure management). With these challenges in mind, the Medical Research Council recommend that development of a telehealth service is iterative with learning from development, piloting, evaluation and implementation informing each step (32).

**Aim of the study**

We conducted a pilot and feasibility randomised controlled trial (RCT) in patients and carers of usual care plus TiM, versus usual care. The results of the pilot and feasibility trial are described in a parallel publication (33). In this paper we describe the process evaluation (34) which aimed to gain a deeper understanding of the use of the TiM system.

We aimed to observe the processes that occurred when the TiM was used to deliver specialist multidisciplinary care in MND exploring:

- TiM technology set-up and delivery
- Participants' adherence to TiM
- Participants' attitudes towards TiM
- Clinicians' attitudes towards TiM
- Potential impacts of the TiM on participants and staff (both intended and unintended)
- The mechanisms and contextual factors that may affect the impact and implementation on a larger scale.

We also aimed to:

- Identify problems with TiM
- Begin to implement and evaluate improvements
- Identify further improvements and uses of the TiM.

## METHODS

**Study design**

The methods of the pilot and feasibility RCT are described in a parallel publication (33). A supplementary file [Online supplementary file Methods TiM protocol and statistics analysis plan Process evaluation 2019] contains the TiM protocol, topic guides and statistics analysis plan. In brief, patients with MND (amyotrophic lateral sclerosis, progressive muscular atrophy and primary lateral sclerosis) and their primary informal carer currently receiving care from the Sheffield MDC were recruited and randomised to receive either the intervention (TiM plus usual care) or control (usual care alone). Participants were followed up for between six and 18 months. Usual care involved invitations to the MDC two to six monthly plus access between visits to the multidisciplinary team via a MND specialist nurse by telephone, email or through liaison with other healthcare professionals.



For peer review only - http://bmjopen.bmj.com/site/about/guidelines.xhtml

## Ethics and consent

Approval was gained from Leeds Bradford Research Ethics Committee (REC reference 14/YH/1068) and the sponsor (Sheffield Teaching Hospitals NHS Foundation Trust Clinical Research Office). Trial identifier ISRCTN26675465. Written or witnessed verbal consent was gained from patients, carers and the telehealth nurse prior to randomisation.

## Intervention

A detailed description of TiM has been published (27). The TiM Patient/Carer App contains a set of patient and carer questions assessing the disease progression, complications of MND, use of medical interventions and patient and carer wellbeing. The app was loaded onto a 7-inch Samsung Galaxy tablet with 3G internet. We asked participants to complete the questions weekly or more frequently if they desired. The results were sent using the internet to a clinical portal used by the MDC specialist nurse (the telehealth nurse) who had over 15 years experience of MND care. The portal assigned a red, amber or green flag to all the answers depending on a predetermined algorithm created by the TiM developers. The telehealth nurse was expected to review the answers and could liaise with specialists in the wider multidisciplinary team (MDT) and provide telephone advice. All patients and carers continued to receive usual care, Sheffield MDC appointments and telephone/email access to the telehealth nurse. Clinic appointments could not be postponed but could be expedited. The nurse was required to have a discussion with the patient, carer or clinician to confirm the accuracy of the information prior to making any clinical decisions. Also loaded onto the tablet were educational materials (known as the Knowledge Centre) and within the app was a section where patients could make a list of issues the participant wished to discuss at clinic (the Problem List) and educational messages, which appeared between questions.

EH recruited participants at home. Participants were given the tablet and wifi-enabled standing weighing scales. They were given face-to-face training, written information, telephone and email support. EH contacted patients after two weeks if they did not submit a TiM session and visited at one and six months for interviews which provided an opportunity to resolve technical issues. EH trained clinicians to use the clinical portal in face-to-face training, which took approximately ten minutes. During the trial we gained feedback from users in order to improve the system. The trial started in October 2014 and one major change to the patient app and the clinical portal was implemented in October 2015.

## Data collection

A detailed description of all data collection is described in the accompanying feasibility paper (33) and in the protocol [Online supplementary file Methods TiM protocol and statistics analysis plan Process evaluation 2019]. Patient/carer reported outcome measures were collected at 0, 3, 6,12 and 18 months using postal questionnaires. These assessed patient disability, depression, anxiety, quality of life and health utility. To gain a deeper understanding of the processes involved we evaluated patient, carer and staff experiences of the TiM. Patients


For peer review only - http://bmjopen.bmj.com/site/about/guidelines.xhtml

and carers completed satisfaction questionnaires using postal questionnaires at the same intervals. We also collected clinician reported adverse events and health resource use using postal questionnaires.

We conducted semi-structured interviews. Interviews were conducted by EH at baseline (control participants), one and six months (intervention participants). Interviews with the telehealth nurse were conducted at 14 and 18 months. The 18 month interview was conducted by an experienced qualitative research WB. Additionally an interview with an MND specialist community nurse who cared for a number of participants in both treatment arms during the study was conducted at 18 months. Topic guides [Online supplementary file Methods TiM protocol and statistics analysis plan Process evaluation 2019 page 41) included participants' experience of specialist MDC and community care, attitudes towards technology and digitally enabled care, expectations and experiences using TiM, reasons for good and poor TiM usage, potential impacts TiM could have on MND care, and improvements and future applications of TiM. Early results and observations from the trial influenced the topic guides for later interviews. Interviews were audio-recorded, transcribed verbatim, checked by the interviewer and organised using NVivo (35). Thematic analysis was used (36). A triangulation process compared the quantitative and qualitative data to further understand and explain important, incongruent and unexpected observed phenomenon (37). EH conducted the analysis with supervision from WB. Results were presented to the trial management group and steering committee

MDC physicians completed a satisfaction questionnaire ("Shadow monitoring form", see [Online supplementary file Methods TiM protocol and statistics analysis plan Process evaluation 2019] page 71) when they saw a patient in clinic using TiM capturing opinions on the accuracy, feasibility and acceptability of TiM. Field notes were also taken to describe any problems with TiM, solutions that were used and their outcomes. At the end of the trial we downloaded all data collected by TiM into Excel. This contained the time and date of every session, all answers provided by patients and carers, all alerts generated by the system and all clinical notes made by the telehealth nurse. We used descriptive statistics to report the TiM use. We attempted to record the telehealth nurse's time spent using TiM using diaries but these were incomplete. Instead, this was explored using interviews.

**Patient and public involvement**
During development of TiM (27) and the protocol we consulted patients, carers and the Sheffield MND Research Advisory group (a patient and public involvement group). They reviewed the intervention, principles of the trial, trial design, outcome measures and participant information leaflets and provided comments on their feasibility and accessibility. They were not involved in recruitment. Results of the study have been communicated at various public meetings, through the Sheffield MND Research Advisory group and local branch of the MND Association and a lay summary will be circulated. Members of this group attended the trial steering and trial management groups. AQ was a


For peer review only - http://bmjopen.bmj.com/site/about/guidelines.xhtml

member of the trial management group, provided advice on the research methods, interpretation of the data and dissemination and is a co-author on this paper.

## RESULTS

### Participants

40 patients and 37 informal carers (three patients had no carer) were recruited. Two TiM patients withdrew due to severe illness and one died before six months, the rest used TiM for between six and 18 months. Participant characteristics are reported in full in the parallel paper (33). The patients broadly represented those attending an MDC including patients at all stages of disease (Table 1). Whilst patients' use of digital technologies was common, some participants had little or no experience using technology. 38 (95%) patients had home broadband and 33 (83%) had mobile internet reception.

For peer review only


For peer review only - http://bmjopen.bmj.com/site/about/guidelines.xhtml

Table 1 Participant characteristics

| | **Telehealth (n=20)** | **Control (n=20)** |
|---|---|---|
| Patient gender, male | 14 (70%) | 14 (70%) |
| Age (years) Mean (SD), range | 60.4 (11.7), 30-78 | 60.0 (10.0), 39-73 |
| Disease duration (months) Mean (SD), range | 53 (48), 12-197 | 46 (35), 7-123 |
| King's ALS clinical stage[a] | | |
| 1 | 3 (15%) | 2 (10%) |
| 2 | 4 (20%) | 5 (25%) |
| 3 | 5 (25%) | 8 (40%) |
| 4 | 8 (40%) | 5 (25%) |
| Use of the TIM App | | |
| Independently | 17 (85%) | 17 (85%) |
| Help from carer | 1 (5%) | 1 (5%) |
| Patient instructs carer | 2 (10%) | 2 (15%) |
| Patient technology use[b] | | |
| Daily | 14 (70%) | 18 (90%) |
| A few times per week | 3 (15%) | 1 (5%) |
| Once a week | 1 (5%) | 1 (5%) |
| Every few weeks | 0 (0%) | 0 (0%) |
| Never | 2 (10%) | 0 (0%) |
| | **Telehealth (n=19)** | **Control (n=18)** |
| Carer gender, male | 4 (21%) | 5 (28%) |
| Carer age (years) Mean (SD), range | 59 (12, 42-84) | 60.8 (11,38-73) |
| Relationship to patient | | |
| Partner | 18 (95%) | 16 (89%) |
| Child | 0 (0%) | 1 (6%) |
| Parent | 1 (5%) | 1 (6%) |
| Carer technology use[b] | | |
| Daily | 12 (67%) | 16 (84%) |
| A few times per week | 1 (6%) | 0 (0%) |
| Once a week | 1 (6%) | 2 (11%) |
| Every few weeks | 0 (0%) | 1 (5%) |
| Never | 4 (22%) | 0 (0%) |

[a]King's stage 1 refers to patients with functional deficit in 1 domain, stage 2 refers to disability in 2 domains, stage 3 refers to disability in 3 domains and stage 4 refers to patients requiring NIV and/or gastrostomy. King's stage was calculated using the ALS-FRS-R scale at baseline. [b] Technology use: computer, tablet, smart phone. SD: standard deviation

For peer review only

**TiM technology set-up and delivery**

*[Online Supplementary data file Results Quotes and Figures Process evaluation 2019 tables 1-4]*

All participants allocated the intervention received the TiM system and could use it using mobile or home broadband. Technical problems and solutions were all resolved and had little impact on the trial except the wifi-enabled scales which were not found to be practical due to a lack of reliability connecting to the internet (Table 2). At six months all the patients and 14 (93%) of the carers felt TiM was easy to use (Figure 1). 14 (93%) patients and carers did not think the system was tiring and 13 (87%) did not think the questions were distressing, nor the use of the system intrusive.

*"It's so easy to do; it literally takes five minutes from home." Patient 317*

27 patients (68%) reported some difficulty using technology due to upper limb disability but 34 (85%) could use the patient app independently (although they might need a carer to help charge the tablet). One patient could use the app but during training it was noticed that his answers were not accurate (Patient 073). He was unable to communicate verbally and exhibited behaviours suggestive of mild cognitive deficits. In this case, he was happy for carers to help him use it.

Participants found face-to-face training important. Many were pleasantly surprised by their achievements.

*"I thought I wouldn't be able to do it (laughter) but I can…." Carer 228*

The main perceived barrier was participants' experience using technology.

*"…we're quite happy to deal with it, as long as it wasn't too techy" Carer 062*

Many participants (including those using technology daily) perceived themselves lacking an intrinsic ability to use technology, saying that they were "*bad*" at it, thinking others were more "*wired*" to using technology. They shared common attitudes towards their abilities, experience and approach to technology. They reported struggling to learn to use new technology finding it stressful and were fearful of making a mistake or breaking the device. They did not feel in control of technology, expressing frustration when technology did not "*obey*" them. This meant they were not confident adapting to unfamiliar technology. They tended to use technology for a limited number of basic tasks, lacking confidence to problem solve when technology *"went wrong"*, relying instead on others. Some low users explained they never learnt, or did not see the need to use technology, particularly if their partner used it.

One couple (Patient 166) had no experience of technology and were highly critical of the perceived intrusion of technology in their lives. They described themselves as *"technophobes"*. However, they trusted their care team would provide them with a secure and safe system. Only one carer in his 80s (Carer 217) struggled to *"catch on"*, needing help from his partner. He had little prior experience with technology and it was later noted that he had difficulties with



For peer review only - http://bmjopen.bmj.com/site/about/guidelines.xhtml

language due to another medical condition.

For peer review only

Table 2. Technology problems encountered during the trial

| Problem | Solution adopted | Impact on the use of the TiM | Recommendations for future TiM use |
|---|---|---|---|
| **TiM Patient App software** | | | |
| Poor finger dexterity | Handheld stylus provided. | 85% used TiM independently. | Provide stylus to all patients. |
| | Carer help to use device | Interview data suggested that help from carers was acceptable. | Encourage carer support. |
| Difficulties entering login password | Telephone support | Problem resolved with second TiM app release | Provide face-to-face training.<br>Provide telephone technical support. |
| | Login page redesigned | No further problems reported. | Make local staff familiar with the software. |
| Lack of confidence using the app or other features on the TiM | Face to face training<br>Partner/family helped | None: all participants could use the app. | Provide face-to-face training plus an additional contact after a few weeks to reinforce learning. Identify low confidence/experience users & provide extra training. |
| Patients not giving correct answers | Patient completing TiM with their family | Uncertain impact . | Capacity assessment at recruitment. Check TiM answers in training and in clinic. |
| **Tablet** | | | |
| Tablet stored in place not accessible to patient | No solution available. | Interviews suggested that adherence was reduced for two patients. | Use patients' own equipment where possible. |
| Tablet battery drained, unable to switch on | Telephone advice. | No impact. | Use patients' own equipment. Familiarise local staff with hardware. |
| Unexpected screens/ software updates | Telephone advice. | Reduced user confidence in tablet but no impact on use. | Use patients' own equipment. Use a basic tablet that only displays the TiM app. |
| User fear of "breaking" the tablet | Face to face training to improve user confidence | Patients/carers reluctant to use the additional features on the tablet | Use patients' own equipment.<br>Face to face training. |
| **Internet connection** | | | |
| Poor 3G phone signal | Used patients' own broadband. | None: solution found for all patients (3G or patients' own broadband) | Use patients' own broadband. Check internet availability prior to TiM enrolment. |
| Patients switched wifi off | Home visit required. | Several TiM sessions failed to download. | Monitor adherence. Alert team if adherence is low. |
| Unreliable connection between scales and tablet/broadband | A separate 3G Wi-Fi route provided ("Mifi"). No solution if using broadband. | Additional home visits required and loss of weight data for several weeks. | Manual weight recording Avoid using peripheral devices. |
| **Clinician portal** | | | |
| Password/login problems | Support by external IT team. | Delayed access during MDC visit. | Local systems access support. |


For peer review only - http://bmjopen.bmj.com/site/about/guidelines.xhtml

**Participant adherence to TiM.**

*[Online Supplementary data file Results Quotes and Figures Process evaluation 2019 tables 4-6]*

Participants preferred to complete the TiM weekly (13, 87% of patients, 8, 53% of carers). They reported that weekly sessions helped them remember to use the TiM. Some suggested they would complete TiM less frequently if nothing had changed. The telehealth nurse suggested that fortnightly information was sufficient to enable her to assess a patient's progress, and even less frequently for carers. Adherence was calculated by dividing the total number of sessions completed by the number of weeks participants were in the trial (Figure 2 and Figure 3). 14 (70%) patients and 10 (55%) carers completed TiM sessions on average, at least fortnightly. 13 (70%) carers completed sessions, on average, at least every three weeks. Some participants who completed fewer sessions still continued to use TiM regularly. Adherence dropped during the study, but at 12 months 80% of patients were still using it weekly. The main reasons for low adherence were forgetting, holidays and deterioration in their illness. A lack of feedback about the answers participants gave was also a disincentive to use TiM, particularly when if they were expecting feedback.

*"It's like all surveys... they say "your opinion is important..." You say something absolutely and nobody comes back to you. And you think: well how important is that survey?" Carer 228*

The use of the additional features of the tablet (the educational messages, Knowledge Centre or Problem list) was not monitored during the trial. Unlike the main TiM app, participants reported that they were less likely to use these. Most were aware of these resources, particularly the short messages that appear whilst completing the TiM questions. Some participants reported reading some of the additional material but no one felt strongly that these had had a major positive impact with only half agreeing that they were useful (Figure 1).

**Participants' attitudes towards TiM**

*[Online Supplementary data file Results Quotes and Figures Process evaluation 2019 tables 7-11]*

14 (87%) patients and carers agreed that TiM questions were relevant to them and 12 (73%) patients and carers felt able to report all the problems they experienced using TiM (Figure 1). 13 (80%) patients and 12 (73%) carers would use it again. 14 (87%) patients and 12 (73%) carers would recommend it to another patient.

*"I think it's probably one of the best ideas to come out of the NHS for years." Patient 122*

Participants did not find using TiM intrusive or upsetting. They explained they were reminded about their disease everyday already and knew themselves if they had deteriorated without using the TiM.

*"It doesn't bother me. When I was first diagnosed I was not keen on talking about it. I've since got better and I think that helps. I'm quite happy to be reminded. I think you are reminded every day. That tablet makes no difference to that." Patient 381*


For peer review only - http://bmjopen.bmj.com/site/about/guidelines.xhtml

Some participants thought the questions were repetitive, particularly carers. Some noticed they reported the same problems every week. Some felt the questions were insufficiently sensitive to reflect the day-to-day fluctuation in their condition. One carer suggested that the questions should use other ways to assess carers lives more critically, which could be used to encourage carers to consider their own wellbeing and adopt coping strategies.

**Clinicians experiences using TiM**
*[Online Supplementary data file Results Quotes and Figures Process evaluation 2019 tables 12-13]*
The telehealth nurse said that using the clinical portal was "*very, very easy*". taking "*minutes*" per use. She explained that initially she looked at the system every day but by the end of the trial she looked weekly, and sometimes a little less often. She would look at each patient but focused on new and changing alerts. She explained that if she saw a patient/carer's answer was associated with a green flag it indicated everything was "*ok*", orange meant "*there's maybe some elements that might need to be looked at*" whilst a red flag indicated a problem.

*Safety and accuracy of TiM*
No adverse events related to TiM were reported and no participant reported delaying seeking medical attention as a result of TiM. 38 clinician satisfaction forms were completed when patients using TiM attended clinic. All agreed that TiM gave a useful and accurate picture of the patient and carer's condition and was a positive influence on the consultation. However, 20 (54%) were completed by the investigator (EH) who saw the patient in clinic, 18 (46%) were completed by other physicians.

**The reported potential impacts of TiM**
*[Online Supplementary data file Results Quotes and Figures Process evaluation 2019 tables 14,15]*
Reported potential impacts included improved monitoring and communication with the clinical team and improved effectiveness of MDC visits and care coordination. Other potential impacts included improved patient awareness of their disease and the ability to identify carer distress.

*Improving links with the clinical team*
Participants thought TiM could provide a "*direct link*" with the care team, increasing monitoring, providing better connection with specialists and enabling earlier identification of problems. Participants (particularly carers) also felt reassured that they were being thoroughly monitored. Reassurance was particularly beneficial for those who had infrequent contact with clinicians, either because they were early in the disease or were progressing slowly.

*"P… for the first year no day was normal… You imagine symptoms, … you think...that must be related to the MND...?*
*Q: Do you think there might have been a point in your disease where those questions ...were useful?*


For peer review only - http://bmjopen.bmj.com/site/about/guidelines.xhtml

*P: Nearer the beginning, definitely…If I could have camped in [neurologist]'s house for the first six months I would have done, just so she was there, so I could say; "but what about this, what about that?"… in the first year I would have filled that in every day, just to have that touch point" Patient 047*

*Improved awareness of the disease*

Many participants thought that TiM improved their awareness of their condition. They felt that they did not need to track their progress using TiM as they knew themselves how they were changing, but some had become more aware of their weight and nutrition. Rapid progression was something all patients feared so those with slowly progressive disease noticed that their answers did not change rapidly. This made them feel more positive.

*Early identification of problems*

The telehealth nurse described TiM as "*a tool to aid the patient and then aid the nurse*". She could use TiM to gain more information and alert her earlier to problems. In one example, the TiM detected that one patient's condition was declining quickly and the carer reported high levels of strain. The couple was struggling to accept his decline and the patient tried to minimise his symptoms. TiM alerted the telehealth nurse to this patient's rapidly declining weight and bulbar function (Figure 4) She contacted him, discussed this information and encouraged him to consider gastrostomy insertion, which he accepted. He was asked about whether TiM affected this decision and explained that the information had been helpful:

*"The questions nudged me to facing what I could do and not what I can't...*
*I was frightened by the speed of loss of weight but was convinced how much muscle I lost. [sic]" Written quote Patient 409*

Other patients had reported problems on the app that they had not disclosed to their care team. The telehealth and community nurse speculated that this was because the patients were struggling to accept the change in their disease or need for treatment and might have felt that they can be more honest using technology. They found this information useful to gain a more complete picture of the situation.

*Improving the multidisciplinary clinic*

The telehealth nurse and physicians suggested that TiM could improve the MDC clinic. The telehealth nurse said if TiM could reduce the burden faced by patients attending clinic it would be welcomed by nurses. Potential benefits included facilitating sharing of information, collecting information and identifying problems before the visit. This could help plan the MDC visit to address the pertinent issues, shorten the time spent in hospital and make telephone appointments easier and a better alternative to face-to-face.

After seeing patients in their clinic using TiM, clinicians completed a feedback form (Shadow monitoring protocol). 23 forms were completed. On 19 (79%) occasions clinicians agreed that the information gathered by TiM could be used to make appropriate decisions without the patient attending in person. After 4



For peer review only - http://bmjopen.bmj.com/site/about/guidelines.xhtml

(17%) appointments they answered "*neutral*". 14 (93%) patients said they would use TiM if they were unable to travel to clinic and 11 (73%) felt that they would be happy to have their appointment delayed if the doctor felt they were stable. Three (20%) were unsure and one (7%) disagreed. When interviewed, participants thought that the individual should choose how they used TiM depending on their needs, preferences and speed of progression. Some patients who were progressing slowly did not feel frequent MDCs were valuable. They were happy to use TiM and reduce the frequency of appointments as long as they could access the team quickly if required. Others wanted to attend the MDC to receive psychological support or address their problems and did not want to reduce visits. Some were not keen on telephone appointments, particularly when discussing sensitive matters or when they had speech difficulties. Similarly, the telehealth nurse explained face-to-face meetings helped her develop her relationship and gain information about the patient and carer. She felt some aspects of their care would need a combination of TiM system and communication with the patients/carers and other members of the MDT. Both clinicians and patients identified the need for face-to-face consultation to fully evaluate respiratory symptoms.

**Impact on carers**

Carers felt that their wellbeing was directly linked to the patients' wellbeing: interventions that improved the patient's life or carer's ability to provide care could improve a carer's wellbeing. Carers felt reassured that their loved one was being monitored and that problems could be identified and solved more rapidly.

*"I think the benefit to P is real. Because ... somebody is there on hand looking at things...Because it's slow with P and he doesn't need as much attention and care, it's easy to feel detached from any positive interaction. Whereas with [TiM], you know somebody's there and if there was something you'd pick up quite quickly as apposed to, waiting until your next twelve week appointment" Carer 122*

Carers and the telehealth nurse reported that the information highlighted by TiM system could help couples accept the diagnosis and the problems they were facing, and enable them to look positively towards receiving additional medical and social care. Carers reported TiM prompted them to consider their own wellbeing and consider help if they were experiencing difficulties. They explained that usually the patient was present in meetings and healthcare professionals tended to focus on the patient's needs. This meant it was difficult for them to discuss their own concerns because of the potential impact this may have on the patient. Carers thought that TiM provided an opportunity for them to express their feelings in private, separately from the patient but still in the convenience of the home environment, without having to leave the patient. The impersonal nature of TiM was also an advantage as it enabled carers to be more honest about their difficulties.

*"I think it's a really good way of doing it. Because whereas I probably try and flower things up a lot of the time ...I found that, because it was just me and the tablet and I was able just to be totally honest about how I was… feeling at that particular time… The impersonal*



For peer review only - http://bmjopen.bmj.com/site/about/guidelines.xhtml

*format… of the way it was actually done... for me, has been a real help just to be able… to do that." Carer 409*

**Problems with TiM**

*[Online Supplementary data file Results Quotes and Figures Process evaluation 2019 tables 16-18]*

Whilst the participants did react positively to TiM, many felt it had not had a significant impact on their care. At six months, only seven (47%) patients and five (33%) of carers agreed, *"The MND team contacted me quickly if my condition changed or I had a problem*". In some cases participants felt their condition had not changed during the trial so TiM had not highlighted any problems. In other cases, problems were reported and help was offered but declined either because the participant did not want it or because they did not think the MDT could help. However, the main explanation for this perceived lack of impact related to the large number of alerts generated by TiM, the way in which the telehealth nurse acted upon information and lack of interaction between the participants and the telehealth nurse when problems occurred.

*TiM system alerts*

Of the total 585 TiM sessions completed, only 19 (3%) of all the patient/carer sessions return answers all of which were flagged as green. 322 (55%) of sessions contained answers which generated at least one red flag and 244 (42%) generated amber alerts (Figure 5a). We analysed individual answers and clinical notes for ten randomly selected patients (334 sessions). Bulbar questions caused most red alerts (usually due to swallowing difficulties or excessive saliva), followed by limb function (usually due to falls) and respiratory symptoms. In the 334 sessions analysed we identified 99 notes made by clinicians. 32 (32%) notes reported the telehealth nurse taking action (Figure 5b): commonly providing telephone advice or liaising with the MDT to share information and coordinate care. 17 (17%) times she planned to review the problem when the patient next came to clinic. In over half of occasions the telehealth nurse documented that she took no action and in only 18 (18%) occasions did she contact the patient or carer. She explained that she usually took no action because she already was aware of the problem, the participant was awaiting treatment or had declined to accept the recommended treatment/advice. She felt that she was seeing the same problems repeatedly. This was particularly frustrating when she had already taken action or when she felt there was no action required. Whilst she did not feel TiM took up a lot of her time, these multiple problems for which she saw no solution caused an element of psychological burden. She could not control how the alerts were generated and would prefer to be able to "*pause*" these alerts.

*Patient-nurse interaction and feedback*

Some participants recalled the telehealth nurse contacting them after reporting a problem on the TiM. The nurse would usually offer reassurance or new advice. Whilst the telehealth nurse felt that these interactions did not have an impact, in the face of a relentlessly progressing disease, these interactions were welcomed


For peer review only - http://bmjopen.bmj.com/site/about/guidelines.xhtml

by patients and carers and it reinforced their perception that specialists were monitoring them closely.

*"...I did the second questionnaire, and within a day [Telehealth nurse] called.*
*Q: Were you expecting her to call?*
*P: No, I wasn't actually. It was just a bolt out of the blue...I find that quite positive. It shows that the whole idea of it works." Patient 122*

The telehealth nurse prioritised problems that were unexpected and placed less priority on problems she expected to occur in MND. Contacting the patient depended on whether the problem needed or had a solution.

*"She red flagged that she'd fallen, which is quite a common occurrence on a lot of patients, and I don't particularly worry unless they've been very, very well and then suddenly." telehealth nurse*

Problems the nurse perceived not to be significant were often felt to be important to patients and carers. For them, acknowledgement was helpful, even if no solution was needed.

*"[falls] ... knocks your confidence ...I probably were putting too much onus on [the hospital MDC] because (laughs) we've got this and there's not [much] they can really do about this and we know that...*
*Q: But that kind of acknowledgement's quite important?*
*C: I do, yeah, it's a bit of support, in't it, it's knowing that somebody else is in your corner." Carer 423*

The telehealth nurse found it easier to manage patients and carers whom she already knew well. She developed relationships through hospital visits and a telephone helpline and through liaison with the community MDT. When the telehealth nurse had less knowledge of the patient it could be more difficult to interpret the information from TiM and liaison with the MDT was less easy. She also explained that she often did not know carers well and sometimes felt there were less obvious solutions to carer distress making her reluctant to telephone. In contrast, most carers felt extremely positive that the nurse provided vital counseling and advice.

The telehealth nurse felt that a more formalised protocol, outlining how she should respond to alerts, would likely increase the number of telephone calls made to patients. However, she felt it was important that she could use her knowledge and experience to decide how to respond to the information presented and would find it difficult working to an overly prescriptive protocol.

**Improvements made to TiM during trial**

Additional questions were added to the patient app: these recorded bladder and bowel symptoms and recorded disease progression in those with more severe disability (using the ALSFRS-EX (38)). Participants felt it was very important that the information provided was accurate and sometimes wanted to give more information in their answers. A free text facility was added but this was used



For peer review only - http://bmjopen.bmj.com/site/about/guidelines.xhtml

very infrequently. We added a heatmap (Figure 4) to the clinical portal which displayed a visual representation of patients' answers over the previous six months. We added the ability for the telehealth nurse to temporarily pause alerts. The telehealth nurse found the heatmap helpful to enable her to get an overview of things she thought were important, such as weight and to see which category had changed. She also found the ability to pause alerts helpful.

**Recommended improvements to TiM**

*[Online Supplementary data file Results Quotes and Figures Process evaluation 2019 tables 20-22)*

The most important improvement we identified was to facilitate communication between the nurse and participants. Most participants did not want automatic feedback on their progress from TiM: they could judge their progress themselves. They were also worried how they would react to seeing objective measures of decline without any interpretation from their clinical team. However, many expected some feedback. The minimum expected was to know that their information had been received and reviewed and for their problems to be acknowledged in some way, even if no action was taken. Participants were asked about their preferences for communication. Telephone and face-to-face conversations were felt to be important for sensitive or complex discussions.

*"I think it's not personal... I'd rather see somebody or talk to somebody than, than read about it on a screen." Carer 366*

Patients found email helped them communicate at their own pace. This was particularly helpful for those with speech problems where a phone call would be difficult. Participants were happy waiting for a definitive answer if they received an acknowledgement. Participants also thought TiM could be used to arrange appointments and other administrative matters and they were happy for their answers to be shared with other clinicians. The Community MND nurse felt TiM information would be useful but the clinical portal would be difficult to access in the community without a computer. She also explained she was *"not good with computers."* but would be happy receiving alerts and information from someone else.

**DISCUSSION**

This detailed study of a digitally-enabled MND service confirmed TiM was acceptable and feasible for patients, carers and staff. Differences in outcomes between the two groups were not formally assessed, however, our findings suggest that the TiM was accurate and safe. The study also gained a deeper understanding of the potential value of TiM and the context in which benefits might be seen (39). We identified some key barriers and enablers to acceptance and use of telehealth which are applicable to other digitally enabled care systems. A key initial barrier for participants was their perceived ability to master the technology. However, uptake was good with most participants (even those with severe disabilities) finding that they were able to use the system and sustain a good level of TiM adherence. The interviews suggested that this was


For peer review only - http://bmjopen.bmj.com/site/about/guidelines.xhtml

because of the accessible software and face-to-face training provided. This is in contrast to other telehealth projects which found acceptance and adherence were major barriers (40). This study aligns with other evidence that suggests that patients have a positive attitude to digital products and, in the right setting, respond positively to digitally-enabled care, as long as the service is accessible and continues to offer personal contact with healthcare professionals (41,42) (41). We recommend new interventions should adopt our user-centred design process and offer additional support to those without confidence in technology to make the intervention accessible.

The potential impacts of digitally-enabled care may differ depending on a patient's needs. TiM offered those with early or slowly progressive disease reassurance, information and the ability to remain in contact with the care team when they needed it as well as offering an alternative to clinic visits. Early identification and coordinated management of complications of the disease appears to be most valuable to those patients at a later stage of the disease or those with more rapidly changing disease. These factors may explain why MDC care improves survival. The study also highlighted the potential value of TiM to carers who could receive support separately from the patient, whilst still allowing them to fulfill their role as a carer. Patients and carers already use digital technology to seek reliable and relevant information in order to self-manage (41) and the TiM can be used to provide information and promote self-management and self-efficacy (something which has a positive influence of patient quality of life (43)). This type of interaction also promotes good adherence to telehealth (44). It was therefore interesting to find that participants in this study reported that they did not use the additional education services on TiM that frequently. This may be because many patients wish to deal with problems as they occur, rather than learning extensively about their condition (41). Telehealth could address this by providing a more personalised information resource, signposting to topics at pace appropriate for the user according to their circumstances and the answers they provide on TiM.

The main weakness of TiM was the mismatch between staff actions and the expectations of participants. This was due to lack of communication and high numbers of unnecessary alerts generated by TiM. MND progression is experienced as a series of losses (45,46) and it is important for staff to acknowledge these problems and recognise the value that interactions with clinicians offer to patients and carers. Remote technology risks disrupting the traditional way in which the nurse would gather information, develop relationships and respond to patient difficulties (47). There is a risk of care becoming less patient centre and patients becoming demoralised as the aims of patients/carers and the clinical team no longer align. We recommend care is taken to develop these interpersonal relationships and for staff to be aware of the potential pitfalls of remote management. Key improvements include improving the algorithms to reduce alerts, facilitating feedback and better communication between patients, carers and staff using technology (such as chat facilities, email or telemedicine) and better training of staff who are required to work in ways that differ from the traditional face-to-face model.



For peer review only - http://bmjopen.bmj.com/site/about/guidelines.xhtml

The key strength of this study was the depth in which we examined the processes occurring in order to explain not just what happened, but why events occurred and how TiM could be improved and implemented. However, this was a small study involving only one centre. As some patients died or withdrew early in the study, of the 20 assigned the TiM a maximum of 17 patients were using the TiM at any one time. Only a small number of clinicians, many of whom had been helped with the TiM development were involved. In particular, only one telehealth nurse used TiM and this study showed that clinicians' behaviour clearly influences the way in which the intervention is delivered. In this case, a nurse who engaged more with the system may have resulted in better outcomes. Larger studies are needed to gain a true understanding of what would occur when the service is offered to all patients as "*business as usual",* in different centres using different staff members. Other staff may behave differently and our results are a reminder that they way they use the TiM warrants monitoring to ensure intervention fidelity and safety. The other limitations of evaluating complex interventions such as telehealth in traditional trials are discussed in detail in the parallel paper (33). We recommend that future evaluations should retain the methods employed in this trial to understand processes in detail, and examine implementation in different contexts, continuing to examine factors such as acceptability, adoption, fidelity, safety, costs and sustainability.

**Conclusion**

The TiM system was acceptable to patients and carers with good uptake and adherence. Key to its success will be continued user and staff engagement and ensuring the system complements the existing services. Improvements to TiM should be based on the findings of this evaluation. Future process evaluations should also examine the implementation of TiM at other sites to determine whether the findings from this study can be generalised.

**Funding and acknowledgements**

The TiM was developed within a collaboration between the University of Sheffield, Sheffield Teaching Hospitals NHS Trust, Mylan Ltd and Abbott Healthcare. This trial was funded by a National Institute for Health Research (NIHR) Doctoral Research Fellowship grant to EH (DRF-2013-06-076) and the Motor Neuron Disease Association. The trial was supported by the Sheffield Teaching Hospitals NHS Trust NIHR Clinical Research Facility and the University of Sheffield Clinical Trials Unit. Mylan Ltd supplied the software and hardware required for the trial. EH is also funded by an NIHR Clinical Lecturer award. PJS is supported by NIHR Senior Investigator award NF-SI-0512-10082 and PJS and CMD by the NIHR Sheffield Biomedical Research Centre (Translational Neuroscience) IS-BRC-1215-20017.

This paper presents independent research funded by the National Institute for Health Research. The views expressed are those of the author(s) and not necessarily those of the NHS, the NIHR or the Department of Health.



For peer review only - http://bmjopen.bmj.com/site/about/guidelines.xhtml

**Conflict of interest**
The TiM intellectual property is owned by Mylan and the University of Sheffield. There are no individual conflicts of interest.

**Author contributions**
The following were involved: intervention development (CMD, EH, TW, PJS), trial design and management (EH, MB, WB, CC, AQ, CMD), clinical oversight (CMD and PJS), recruitment and intervention delivery (EH and TW) data collection (EH, WB), data analysis (EH, MB, WB, CMD, CC, SM, AQ) and manuscript preparation (All). All authors reviewed and commented on the manuscript. The authors are grateful to Sheffield MND Research Advisory Group for their guidance on the research methods.

**Data sharing**
All data requests should be submitted to the corresponding author for consideration. Access to anonymised data may be granted following review.

For peer review only

**Figure legends**

Figure 1
Patient and carer satisfaction with TiM telehealth at 6 months.

Figure 2
A visual representation of individual patient adherence over the duration of the trial. Each black dot indicates a completed TiM session. A coloured dot indicates the end of follow-up.

Figure 3
A visual representation of individual carer adherence over the duration of the trial. Each black dot indicates a completed TiM session. A coloured dot indicates the end of follow-up.

Figure 4
The "heat map" for Patient 409. This is a screenshot from the clinical portal. The heatmap reports the highest level flag for each section of the questionnaire (mobility, bulbar, breathing, wellbeing, nutrition) at each week. This indicates the patient has red and amber flags for both bulbar and later mobility. Below is the weight compared to the patients' weight at baseline demonstrating a greater than 15% weight loss.

Figure 5
Figure 5a (left) The frequency of flags generated by the TiM sessions. The total sessions reports the frequency of "top level" flag (green, amber, red) for all 585 sessions completed. Below are the frequencies of "section flags" generated by 10 patients who completed a total of 334 sessions. 5b (left) Actions described in 99 clinical notes. P: patient, C: carer, TN: telehealth nurse

For peer review only

1. McDermott CJ, Shaw PJ. Diagnosis and management of motor neurone disease. *BMJ*. 2008;336:658–62.

2. Traynor BJ, Alexander M, Corr B *et al.* Effect of a multidisciplinary amyotrophic lateral sclerosis (ALS) clinic on ALS survival: a population based study, 1996-2000. *J Neurol Neurosurg & Psychiatry*. 2003;74:1258–61.

3. Ng L, Khan F. Multidisciplinary care for adults with amyotrophic lateral sclerosis (ALS)/motor neuron disease (MND). *Cochrane Database Syst Rev.* 2009;13:105.

4. Aridegbe T, Kandler R, Walters SJ *et al.* The natural history of motor neuron disease: assessing the impact of specialist care. *Amyotroph Lateral Scler Frontotemporal Degener*. 2013;14:13–9.

5. Rooney J, Byrne S, Heverin M *et al*. A multidisciplinary clinic approach improves survival in ALS: a comparative study of ALS in Ireland and Northern Ireland. *J Neurol Neurosurg & Psychiatry*. 2015;86:496–501.

6. Martin S, Trevor-Jones, A, Khan S *et al*. The benefit of evolving multidisciplinary care in ALS: a diagnostic cohort survival comparison. *Amyotroph Lateral Scler Frontotemporal Degener*; 2017;7-8:569-575.

7. Moura MC, Casulari LA, Garbi Novaes MRC. Multidisciplinary Care Improves Survival of Patients with Amyotrophic Lateral Sclerosis in the Unique Health System (SUS) in Brazil. *J Neurol Disord*. 2017;05:1–7.

8. Motor Neurone Disease Association UK. Personal communication. 2016.

9. Hobson EV, Baird WO, Cooper CL *et al*. Using technology to improve access to specialist care in amyotrophic lateral sclerosis: A systematic review. *Amyotroph Lateral Scler Frontotemporal Degener.* 2016;5-6:313–24.

10. Andrew G, Courtney W, Anne M, Susan W, Bethany S, Zachary S. Incorporation of telehealth into a multidisciplinary ALS Clinic: feasibility and acceptability. Amyotroph Lateral Scler Frontotemporal Degener. Informa UK Limited, trading as Taylor & Francis Group; 2017 Nov 8;0(0):555–61.

11. Selkirk SM, Washington MO, McClellan F *et al*. Delivering tertiary


For peer review only

centre specialty care to ALS patients via telemedicine: a retrospective cohort analysis. *Amyotroph Lateral Scler Frontotemporal Degener.* 2017;(5-6):324–32.

12. Van De Rijn Marc, Sabrina P, Mark L-W, Katharine C, Amy SE, Juan E, et al. Experience with telemedicine in a multi- disciplinary ALS clinic. *Amyotroph Lateral Scler Frontotemporal Degener*; 2018;0(0):1–6.

13. Henderson RD, Hutchinson N, Douglas JA, Douglas C. Telehealth for motor neurone disease. *Med J Aust*. 2014;201(1):31.

14. Pulley MT, Brittain R, Hodges W, *et al.* Multidisciplinary amyotrophic lateral sclerosis telemedicine care: The store and forward method. *Muscle Nerve* 2019 :59(1):34-39.

15. Care augmentation by location-link messaging. Call-Me. www.isrctn.com/ISRCTN71271888. [Website. Date accessed April 2019]

16. ALS at home. als-at-home.org. [Website. Date accessed April 2019]

17. Answer ALS. www.answerals.org [Website. Date accessed April 2019]

18. ALS Analyzer. www.alsanalyzer.com. [Website. Date accessed April 2019]

19. O'Brien M, Whitehead B. Optimisation of service for people with Motor Neurone Disease (MND) in Lancashire and South Cumbria. 2010 Jul.

20. Baxter SK, Baird WO, Thompson S *et al*. The impact on the family carer of motor neurone disease and intervention with noninvasive ventilation. *J Palliat Med.* 2013;16(12):1602–9.

21. ProGas Study Group. Gastrostomy in patients with amyotrophic lateral sclerosis (ProGas): a prospective cohort study. *Lancet Neurol.* 2015;14(7):702–700.

22. Stavroulakis T, Baird WO, Baxter SK *et al*. The impact of gastrostomy in motor neurone disease: challenges and benefits from a patient and carer perspective. *BMJ Support Palliat Care.* British Medical Journal Publishing Group; 2016;6(1):52–9.

23. Bergin S, Mockford C. Recommendations to support informal carers of people living with motor neurone disease. *British Journal of Community Nursing*. 2016;21(10):518–24.

24. Galvin M, Carney S, Corr B, *et al*. Needs of informal caregivers across the caregiving course in amyotrophic lateral sclerosis: a qualitative


For peer review only - http://bmjopen.bmj.com/site/about/guidelines.xhtml

analysis. *BMJ Open.* 2018;8(1):e018721.

25. Vitacca M, Comini L, Tentorio M, *et al*. A pilot trial of telemedicine-assisted, integrated care for patients with advanced amyotrophic lateral sclerosis and their caregivers. *J Telemed Telecare.* 2010;16(2):83–8.

26. Creemers H, Veldink JH, Grupstra H, *et al* Cluster RCT of case management on patients' quality of life and caregiver strain in ALS. *Neurology*. 2014;82(1):23–31.

27. Hobson EV, Baird WO, Partridge R, *et al*. The TiM system: developing a novel telehealth service to improve access to specialist care in motor neurone disease using user-centered design. *Amyotroph Lateral Scler Frontotemporal Degener.* 2018;19:351-361

28. Medical Research Council. A framework for development and evaluation of RCTs for complex interventions to improve health. *MRC*. 2000.

29. Steventon A, Bardsley M, Billings J *et al*. Effect of telehealth on use of secondary care and mortality: findings from the Whole System Demonstrator cluster randomised trial. *BMJ*. 2012;344:e3874–4.

30. Fan VS, Gaziano JM, Lew R *et al*. A comprehensive care management program to prevent chronic obstructive pulmonary disease hospitalizations: a randomized, controlled trial. *Ann Intern Med*; 2012;156:673–83.

31. Takahashi PY, Pecina JL, Upatising B *et al*. A randomized controlled trial of telemonitoring in older adults with multiple health issues to prevent hospitalizations and emergency department visits. *Arch Intern Med*. 2012;172:773–9.

32. Craig P, Dieppe P, Macintyre S, *et al*. Developing and evaluating complex interventions: the new Medical Research Council guidance. *BMJ*; 2008;337:a1655–5.

33. Hobson EV, Baird WO, Bradburn MJ *et al*. Using telehealth in motor neuron disease to increase access to specialist multidisciplinary care: a UK-based pilot, feasibility study. *BMJ open*. 2019.

34. Moore GF, Audrey S, Barker M, *et al*. Process evaluation of complex interventions: Medical Research Council guidance. *BMJ*. 2015 Mar 19;350:h1258.

35. QSR International Pty Ltd. NVivo qualitative data analysis software. 2014.

36. Braun V, Clarke V. Using thematic analysis in psychology. *Qual Res*

For peer review only

*Psychol*. 2006;3(2):77–101.

37. Patton MQ. Enhancing the quality and credibility of qualitative analysis. *Health Serv Res*. 1999;34(5 Pt 2):1189–208.

38. Wicks P, Massagli MP, Wolf C, *et al*. Measuring function in advanced ALS: validation of ALSFRS-EX extension items. *Eur J Neurol*. 2009;16(3):353–9.

39. Lamont T, Barber N, de Pury J *et al*. New approaches to evaluating complex health and care systems. *BMJ*. 2016;352:i154–5.

40. Sanders C, Rogers A, Bowen R *et al*. Exploring barriers to participation and adoption of telehealth and telecare within the Whole System Demonstrator trial: a qualitative study. *BMC Health Serv Res*. 2012;12:1–12.

41. Hobson EV, Fazal S, Shaw PJ *et al.* "Anything that makes life's journey better." Exploring the use of digital technology by people living with motor neurone disease. *Amyotroph Lateral Scler Frontotemporal Degener*. 2017;34:1–10.

42. James N, Power E, Hogden A, Vucic S. Patients' perspectives of multidisciplinary home-based e-Health service delivery for motor neurone disease. *Disabil Rehabil Assist Technol*. 2018;14:1–7.

43. Galin S, Heruti I, Barak N, *et al*. Hope and self-efficacy are associated with better satisfaction with life in people with ALS. *Amyotroph Lateral Scler Frontotemporal Degener*. 2018;19(7-8):611–8.

44. Maeder A, Poultney N, Morgan G, Lippiatt R. Patient Compliance in Home-Based Self-Care Telehealth Projects. *J Telemed Telecare*. 2015;21(8):439–42.

45. Foley G, Timonen V, Hardiman O. Exerting control and adapting to loss in amyotrophic lateral sclerosis. *Soc Sci Med*. 2014;101:113–9.

46 Mistry K, Simpson J. Exploring the transitional process from receiving a diagnosis to living with motor neurone disease. *Psychol Health*. 2013;8:939–53.

47. Greenhalgh T, A'Court C, Shaw S. Understanding heart failure; explaining telehealth – a hermeneutic systematic review. *BMC Cardiovasc Disord*. 2017;17,1-16


For peer review only - http://bmjopen.bmj.com/site/about/guidelines.xhtml

For peer review only



For peer review only - http://bmjopen.bmj.com/site/about/guidelines.xhtml

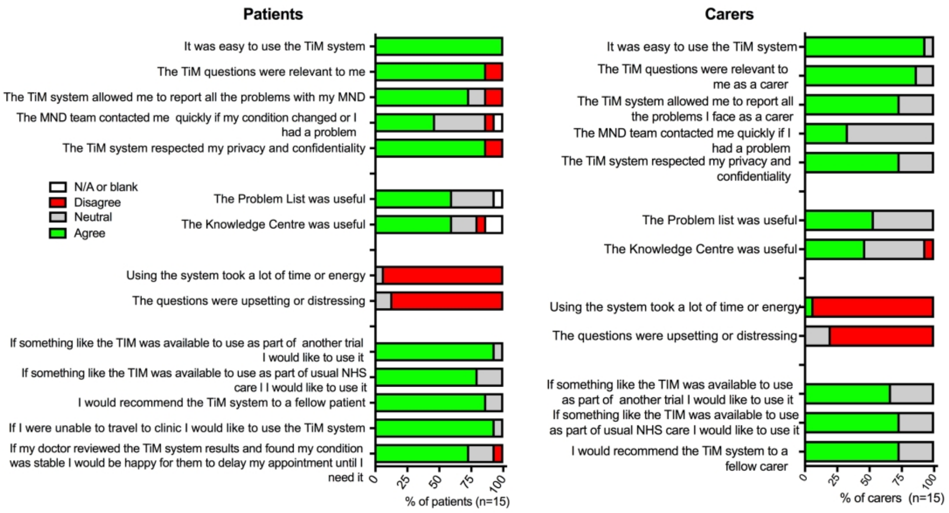


Patient and carer satisfaction with TiM telehealth at 6 months.

264x138mm (300 x 300 DPI)

| TiM ID | Age | Adherence |
|---|---|---|
| 423 | 30s | 104% |
| 166 | 70s | 99% |
| 381 | 60s | 97% |
| 392 | 50s | 93% |
| 228 | 70s | 91% |
| 122 | 60s | 85% |
| 145 | 40s | 83% |
| 366 | 70s | 77% |
| 248 | 60s | 73% |
| 217 | 70s | 73% |
| 56 | 50s | 69% |
| 409 | 60s | 65% |
| 63 | 60s | 60% |
| 354 | 50s | 53% |
| 317 | 50s | 47% |
| 19 | 50s | 41% |
| 172 | 60s | 35% |
| 134 | 40s | 22% |
| 73 | 60s | 22% |
| 47 | 50s | 13% |

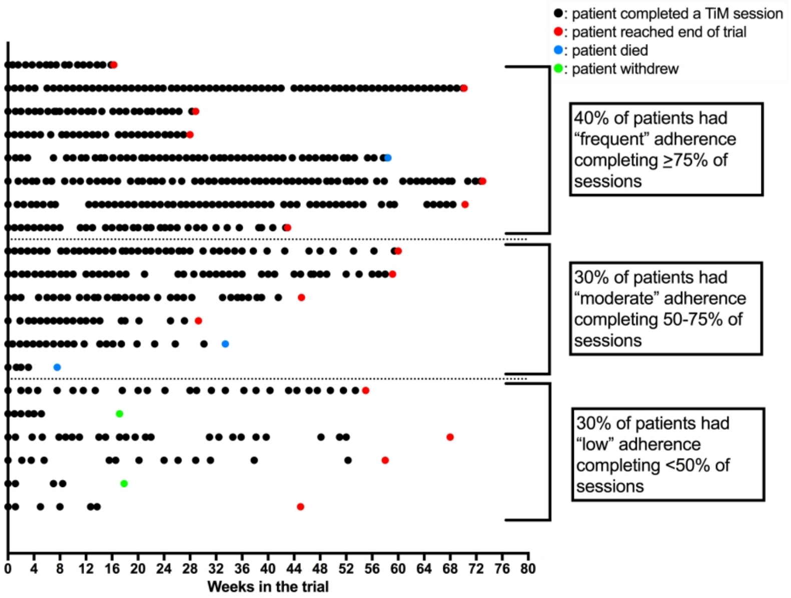


A visual representation of individual patient adherence over the duration of the trial. Each black dot indicates a completed TiM session. A coloured dot indicates the end of follow-up.

275x166mm (300 x 300 DPI)

| TiM ID | Age | Adherence |
|---|---|---|
| 166 | 70s | 95% |
| 392 | 50s | 90% |
| 217 | 80s | 86% |
| 381 | 60s | 83% |
| 423 | 50s | 78% |
| 145 | 50s | 77% |
| 366 | 70s | 74% |
| 248 | 60s | 68% |
| 228 | 60s | 62% |
| 63 | 60s | 52% |
| 354 | 40s | 47% |
| 409 | 50s | 44% |
| 19 | 50s | 44% |
| 317 | 50s | 25% |
| 172 | 60s | 24% |
| 122 | 50s | 19% |
| 73 | 40s | 17% |
| 134 | 40s | 12% |

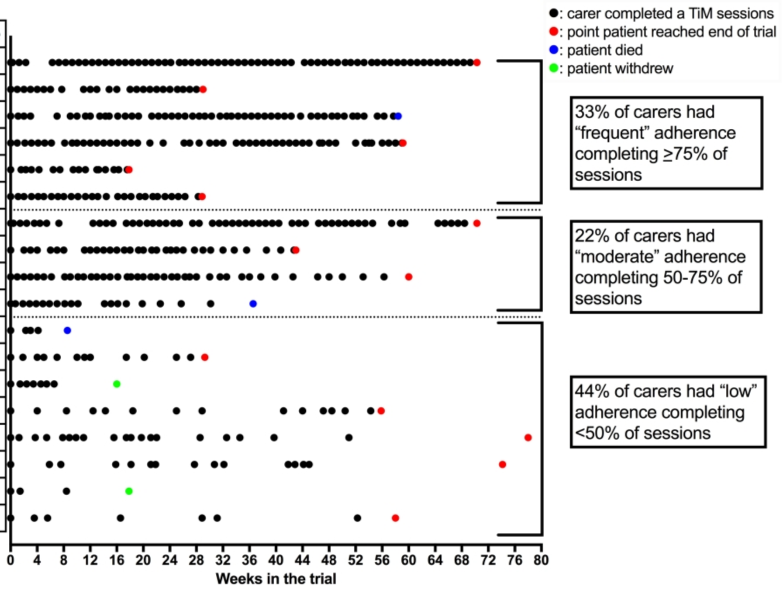


A visual representation of individual carer adherence over the duration of the trial. Each black dot indicates a completed TiM session. A coloured dot indicates the end of follow-up.

277x165mm (300 x 300 DPI)

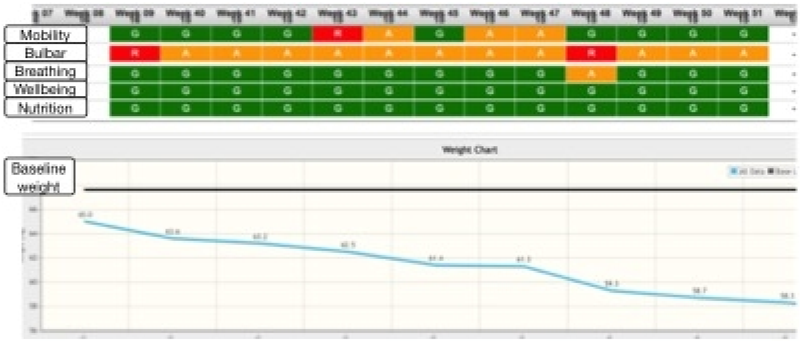


The “heat map” for Patient 409. This is a screenshot from the clinical portal. The heatmap reports the highest level flag for each section of the questionnaire (mobility, bulbar, breathing, wellbeing, nutrition) at each week. This indicates the patient has red and amber flags for both bulbar and later mobility. Below is the weight compared to the patients’ weight at baseline demonstrating a greater than 15% weight loss.

140x59mm (72 x 72 DPI)

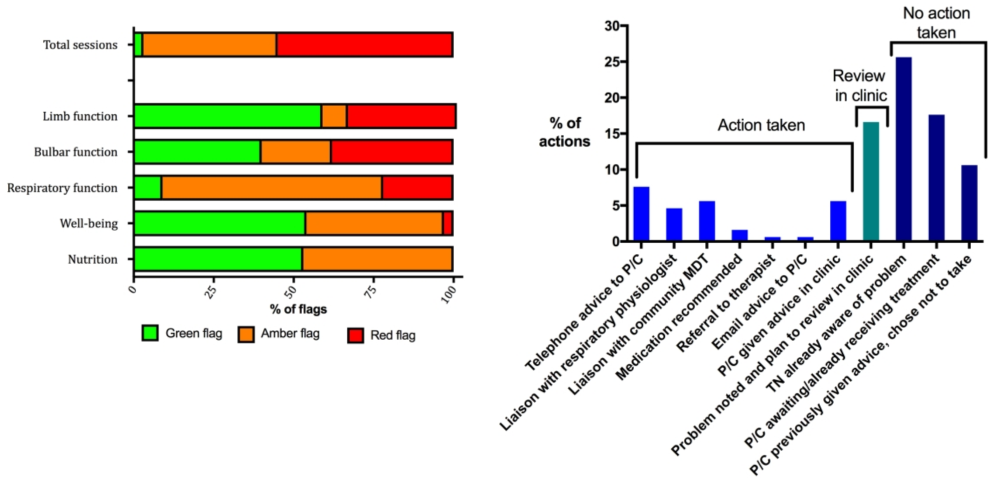


Figure 5a (left) The frequency of flags generated by the TiM sessions. The total sessions reports the frequency of "top level" flag (green, amber, red) for all 585 sessions completed. Below are the frequencies of "section flags" generated by 10 patients who completed a total of 334 sessions. 5b (right) Actions described in 99 clinical notes. P: patient, C: carer, TN: telehealth nurse

193x97mm (300 x 300 DPI)

# Telehealth in motor neurone disease to increase access to specialist multidisciplinary care: a pilot, feasibility study.

Hobson EV., Bradburn M., Baird WO, Cooper CL, Mawson S, Quinn A, Shaw PJ, Walsh T, McDermott CJ

## Supplementary data files

**TiM protocol V1.5.................................................Page 2**
**TiM statistics analysis plan V1.3......................Page 45**
**Shadow monitoring form ..................................Page 71**

For peer review only


For peer review only - http://bmjopen.bmj.com/site/about/guidelines.xhtml

TiM™

# Telehealth in Motor Neurone Disease

## Telehealth in Motor Neurone Disease: A single centre, randomised controlled feasibility and pilot study of the use of the TiM telehealth system to deliver highly specialised care in Motor Neurone Disease at a distance

The University Of Sheffield.

Sheffield Teaching Hospitals NHS Foundation Trust

D4D Devices for Dignity

SITraN Sheffield Institute for Translational Neuroscience

CLAHRC for South Yorkshire



For peer review only - http://bmjopen.bmj.com/site/about/guidelines.xhtml

## General Information

**Sponsor identifier**: 17165
**National Institute for Health Research Portfolio ID**: 17022
**Trial Identifier:** ISRCTN26675465

**Sponsor**

Sheffield Teaching Hospitals NHS Foundation Trust
Tanya.turgoose@sth.nhs.uk
Sheffield Teaching Hospitals NHS Foundation Trust Research Department
11 Broomfield Road Sheffield S10 2SE
Tel: (0114) 2265942
Fax: (0114) 2265937
Email: Tanya.Turgoose@sth.nhs.uk

**Principal investigator & Trial Manager**

Dr Esther Hobson
National Institute for Health Research Doctoral Research Fellow
Neurology Specialty Registrar
Sheffield Institute of Translational Neuroscience University of Sheffield
385a Glossop Road
Sheffield
S10 2HQ
Tel: (0114) 2222261
Fax: (0114) 2222290
Email: esther.hobson@gmail.com

**Chief investigator**

Dr. Christopher McDermott
Reader and Honorary Consultant Neurologist
Sheffield Institute of Translational Neuroscience University of Sheffield
385a Glossop Road
Sheffield
S10 2HQ
Tel: (0114) 2222261
Fax: (0114) 2222290
Email: c.j.mcdermott@sheffield.ac.uk

This study is a non-commercial, portfolio study supported by the DeNDRoN (Dementia and Neurodegenerative Diseases Clinical Research Network). It is funded through a National Institute for Health Research Doctoral Research Fellowship grant.


For peer review only - http://bmjopen.bmj.com/site/about/guidelines.xhtml

Abbreviations

| | |
|---|---|
| AE | Adverse event |
| ALS | Amyotrophic lateral sclerosis |
| ALSAQ-40 | Amyotrophic Lateral Sclerosis Assessment Questionnaire – long form |
| ALS-FRS-R | Amyotrophic lateral sclerosis rating scale-revised |
| BiPAP | Bi-level positive pressure non-invasive ventilation |
| CBI | Carer burden inventory |
| CI | Chief Investigator |
| CLRN | Comprehensive Local Research Network |
| CONSORT | Consolidated standards of reporting trials |
| CRF | Clinical Research Facility, Sheffield Teaching Hospitals |
| CSS-MND | Clinical Saliva Scale for Motor Neurone disease |
| CTRU | Clinical trials research unit, University of Sheffield |
| EQ-5D-3L | EuroQol Group Health Questionnaire, |
| GCP | Good clinical practice |
| GP | General practitioner |
| HADS | Hospital Anxiety and Depression Scale |
| HTA | Health Technology Assessment |
| MCSI | Modified Caregiver Strain Index |
| MDT | Multidisciplinary team |
| MND | Motor neurone disease |
| NICE | National Institute for Health and Clinical Excellence |
| NHS | National Health Service |
| NIHR | National Institute for Heath Research |
| NIV | Non-invasive ventilation |
| PHQ | Patient Health Questionnaire |
| PI | Principal Investigator |
| SAE | Serious adverse event |
| ScHARR | School of Health and Related Research, University of Sheffield |
| SF-36 RAND | 36-Item Short Form Survey from the RAND Medical Outcomes Study |
| SITraN | Sheffield Institute of Translational Neuroscience |
| STH | Sheffield Teaching Hospitals |
| SU | Sheffield University |
| Telecare | A system of sensors, alarms or communication in the home used to support safe living |
| Telehealth | Remote monitoring of patients physiology or patient reported measures, forwarded to a central service with the aim to diagnoses or monitor a medical condition |
| Telemedicine | Videoconferencing consultation |
| TMG | Trial management Group |
| TSC | Trial Steering Committee |
| TiM | Telehealth in Motor neurone disease |
| TM | Trial manager |


For peer review only - http://bmjopen.bmj.com/site/about/guidelines.xhtml

**Contents**


1. Title and abstract
2. Lay summary
3. Background
4. Summary and hypothesis
5. Research objectives
    a. Objective of the pilot study
    b. Objectives of the full scale study
    c. Justification of the pilot study
6. Study Methodology
    a. Participant recruitment and selection
    b. Consent
    c. Randomisation
    d. Inclusion and exclusion criteria
    e. Withdrawal
    f. Compliance
    g. Sample size
    h. Blinding
7. Study treatment
8. Data collection
    a. Quantitative data collection
    b. Qualitative sub-study
    c. Shadow monitoring protocol
    d. Process evaluation
9. Analysis
    a. Feasibility and quantitative analysis
    b. Qualitative analysis
10. Data entry, security and confidentiality
11. Safety and safety assessments
12. Ethical considerations
13. Finance and indemnity
14. Dissemination and publication
15. References

Appendix 1 Drugs produced by Abbott Healthcare Ltd.
Appendix 2 CONSORT flow diagram
Appendix 3 Qualitative interview topic guides


This document describes a clinical study. The protocol is not intended for use as a guide to the treatment of other patients. Amendments may be necessary; these will be circulated to investigators in the study.



For peer review only - http://bmjopen.bmj.com/site/about/guidelines.xhtml

## 1. Telehealth in Motor Neurone Disease: The TiM ™ Study

Telehealth in Motor Neurone Disease: A single centre, randomised controlled pilot study of the use of the TiM ™ telehealth system to deliver highly specialised care in Motor Neurone Disease at a distance.


### Abstract

### Objectives

People with motor neurone disease benefit from the specialist care provided by multidisciplinary teams. As their disease progresses patients struggle to attend hospital and find it difficult to access the care they need. The aim of the TiM system is to improve access to this specialist care by using technology to monitor, educate and communicate with our patients and their carers.

This is a pilot study of the TiM ™ telehealth system. The pilot study is designed to assess the feasibility, acceptability and safety of the telehealth system in clinical practice and of conducting a full study of the system. It will also allow a process evaluation of the system to determine how the telehealth system could be effectively utilised within an NHS service.

### Methods

This is a single-centre, randomized controlled mixed methods pilot study of the TiM ™ telehealth system. It will recruit 40 patients along with their primary informal carer. 20 will be assigned to use the TiM system for a minimum of 6 months (intervention) and 20 will be assigned usual care (control). Quantitative outcome data will be collected at baseline, three and six months, six monthly thereafter and at the end of the trial. Qualitative interviews with participants and staff and analysis of the system in use will enable a process evaluation of the system and the trial methodology. It will also assess the safety of the system in a clinical setting.

### Results

Results of this pilot will determine whether a large, multi-centre full trial is appropriate and enable further development of the TiM system. It is proposed that the TiM system could be adopted into the care of patients with MND throughout the UK.




For peer review only - http://bmjopen.bmj.com/site/about/guidelines.xhtml

## 2. Lay summary

Motor neurone disease is a condition affecting approximately 5 000 people in the UK. It results in progressive weakness in muscles causing paralysis, disability, and eventually death after an average of only three to five years. To receive the expert care provided by motor neurone disease multidisciplinary teams most patients have to travel to regional centres, whilst community-based care is usually provided by non-specialist teams. Between clinic appointments and towards the end of their lives when patients are unable to travel to clinic and they may be unable to access the specialist assessment and care provided in these centres.

Telehealth has been shown to increase access to specialist care in patients with chronic disease, regardless of geography or the ability to travel. The overall purpose of this pilot study is to test the feasibility and acceptability of the TiM telehealth system. The TiM system is web-based system that enables weekly monitoring of patients and carers' health and wellbeing. It has been developed by the Sheffield Motor Neurone Disease Care Centre team in partnership with industry (Abbott Healthcare Products Ltd and Carematix) and other experts within the NHS and the University of Sheffield. The study will also determine whether it would be feasible to conduct a larger study of the system to examine the effectiveness of the TiM system.

Patients with motor neurone disease who are cared for by the Sheffield motor neurone disease clinic will be invited to take part in the trial. 40 patients will be recruited. Their primary informal carer (usually their spouse or close relative) will also be invited to participate in carer monitoring. All patients will continue their usual care but half will also be randomised to use the TiM telehealth system for a minimum of six months.

Information about the participants will be collected at the start, three and six months, and then every six months until the end of the trial by postal questionnaires and during routine appointments. Up to 20 participants in the control group will be interviewed at the start at the trial to explore their experiences on completing the postal questionnaires. Up to 20 participants who are using the TiM system will be interviewed at one and six months to understand the effect the trial and the system has on their lives in more depth. The clinical staff will also be interviewed at the end of the trial.

A pilot study is a small-scale study that is carried out to determine whether a larger study is practical. It will also enable the identification and resolution of any problems with either the telehealth system or the trial procedures.



For peer review only - http://bmjopen.bmj.com/site/about/guidelines.xhtml

## 3. Background

There are approximately 5 000 people in the UK suffering from motor neurone disease (MND) at any one time (1). MND is an incurable disease causing progressive weakness of muscles involving the limbs, speech and swallowing leading to progressive disability and eventual respiratory failure. The average life expectancy following diagnosis is two to three years but the course of MND can vary from only a few months to over 10 years. The distress and burden of the disease affects patients, their family and carers and the relenting progression of disability causes social, emotional and financial strain (2, 3).

There are 22 specialist multidisciplinary MND care centres in the UK. Expert clinicians and therapists offer interventions such as riluzole (which can improve survival by approximately two to three months) and gastrostomy feeding to promote good nutrition (4). Treatment of respiratory failure with non-invasive ventilation (NIV) improves both quality of life and life-expectancy by, on average, 11 months (5). Attendance at specialist MND clinics has also been reported to improve survival independent of these other interventions (6, 7).

The traditional model of care is to review patients at the MND centre at fixed regular intervals. This model is not responsive to patient or carer needs (which can change rapidly) and requires the patient and their family to undertake progressively more difficult journeys to clinic at a time predicted by the clinician at their last meeting. Given the burden associated with travelling to clinic, it is important that visits occur when they are most needed. Some patients whose needs have not changed may not benefit from a clinic appointment at the previously predicted interval whereas others may need more timely intervention.

The highly specialist services provided by the MND clinic are contrasted by the services most patients receive in their community (8). These community teams, who have limited experience in caring for patients with MND, are usually the first point of contact for patients between clinic visits. Lack of expertise in MND amongst community teams and limited access to specialist staff and equipment (particularly at the end stages of the disease) causes patients and their carers to experience significant difficulties (2, 3, 9-12). Research conducted in SITraN and by others, highlights the major impact that caring for someone with MND has on the physical and emotional well being of carers, as well as patients (3, 11, 13-16). Where access to specialist services and community support is limited, this impact is even more notable (3, 11, 13-16). This is particularly a problem in the later stages, when it is usually impossible to attend clinic, when arguably the most care is needed. It is therefore essential that the input from the specialist centre is still possible both between visits and when patients become unable to travel.

### Telehealth to provide specialist care in MND

In the last few years, technology has developed sufficiently to allow high quality communication between patient and their care team at a potentially reasonable cost.



For peer review only - http://bmjopen.bmj.com/site/about/guidelines.xhtml

Trials have shown that telehealth is an acceptable way to improve access to specialist expertise and facilitate self-management patients with long-term health conditions (17-21). In some cases this approach has been associated with a reduction in hospital admissions (17, 18, 20). In 2012, in response to research evidence and the need to provide cost-effective care to an expanding population of patients with chronic disease, the UK government created the "3millionlives" campaign (22). This project in aims foster NHS, academic and industry collaboration in order to provide telehealth services to up to three million people with chronic health and social care needs in the UK.

The problems faced in MND are unlike many common chronic diseases in which telehealth has been previously trialed. Care for MND requires holistic and multi-disciplinary expertise and the use of uncommon interventions such as non-invasive ventilation and gastrostomy feeding. To date, use of telehealth in MND is limited, although small studies do show promise in certain niche areas. Telehealth systems using telephone consultation have been developed with some success in Italy and Portugal, to remotely manage patients who require home ventilation. These systems were associated with a reduction in emergency healthcare usage; more efficient use of staff time and potential cost savings (23-28) In Holland and rural Scotland, MND services have used video-conferencing (29, 30). Both approaches have potential benefits but telehealth used in this way is labour intensive and costly and care is driven by the priorities identified by the clinician rather than those of the patient. No telehealth system has been developed to provide frequent, holistic and highly specialist care to patients with MND at all stages of their illness.

We propose that telehealth could enable people with MND to have better access to the specialist monitoring and care that they require. Patients with MND are able to accurately report their level of disability and appropriate questions can identify new symptoms or early signs of respiratory failure (31-33). These features would suggest that a system of remote, question-based monitoring could provide regular, accurate clinical information to enable the clinician to detect and better manage problems without the patient needing to attend hospital. Telehealth provides the opportunity to provide education and reassurance and support to enable patients to better manage their own care (a core requirement of the National Service Framework for Long-term Conditions (34)).

There are estimated to be 10 million people in the UK living with a neurological condition (1). Both the common diseases such as Parkinson's disease and epilepsy and rarer conditions such as muscular dystrophy and MND require specialist, multidisciplinary support from specialist services. A successful telehealth system may therefore be able to improve the services provided to many patients in the UK and their families.

**4. Summary and hypothesis**

**Summary**



For peer review only - http://bmjopen.bmj.com/site/about/guidelines.xhtml

We will undertake a pilot study of the use of the TiM telehealth system to improve the care of patients and their carers living with motor neurone disease. Whilst telehealth services have been used successfully in other long-term conditions, no service of this kind exists for patients with motor neurone disease. A pilot randomised controlled trial will employ a mixed methods approach to explore the feasibility and acceptability of using the TiM system to improve access to specialist care in MND. The pilot study will also explore the feasibility of a full-scale trial.

## Hypothesis

The TiM telehealth system will:

- Improve the quality of life of patients with MND
- Improved clinical outcomes for patients with MND
- Improve quality of life and other measures of well being for the primary informal carers of patients living with MND.
- Be acceptable to patients, carers and staff
- Lead to more cost effective utilisation of heath care resources


For peer review only - http://bmjopen.bmj.com/site/about/guidelines.xhtml

## 5 Research objectives

### 5a. Objectives of the pilot study

- Determine the requirements of a full-scale study of the TiM system
    - Determine recruitment, retention and withdrawal rates.
    - Determine the most acceptable and appropriate outcome measure(s) that reflect the impact of the TiM system on patients and carers and health resources.
    - Provide an estimate of the resources required to conduct a full-scale study.
- Study the use of the TiM system in clinical practice
    - Assess the relationship between the benefits of the TiM system perceived by staff and participants with those captured by the outcome measures
    - Assessing participants' use and compliance with the TiM system
    - Health-care staff qualitative interviews and focus group
- Assess the safety of the TiM system using:
    - A shadow monitoring protocol
    - Health-care staff qualitative interviews and focus group
    - Analysis of technical and clinical adverse events

### 5b. Objectives of the full-scale study

**Proposed primary end-point**

- Patient quality of life (outcome measure(s) to be determined in the pilot trial)

**Proposed secondary end-points**

Patient outcomes

- Severity of pain
- Severity of oropharygeal secretions
- Incidence of depression and anxiety
- Time from diagnosis to death

Carer outcomes

- Quality of life
- Carer Burden
- Incidence of depression and anxiety

Health economic outcomes involving a cost utility analysis using costs of the system, costs of associated care requirements, EQ5D and patient survival

Safety of the TiM system

- Frequency of adverse events

### 5c. Justification of the pilot study



For peer review only - http://bmjopen.bmj.com/site/about/guidelines.xhtml

Since this type of telehealth has never been evaluated in those with MND a pilot study is necessary to determine how a full-scale evaluation of its clinical and cost-effectiveness could be conducted and to gain a better understanding of how the TiM system would work. This includes evaluating recruitment and retention, as well and resource requirements. By evaluating compliance and safety monitoring and using qualitative the study will also enable a better understanding of how the telehealth system is used by patients, carers and staff.

A number of the proposed benefits of telehealth such as improving quality of life, providing reassurance and support, prompting self-care and a more efficient use of resources (18, 35-40) may be difficult to quantify. The validated measures of quality of life most commonly used in research (EQ5D and SF-36) were not specifically designed for patients with MND or their carers. The ALSAQ-40 tool better encompasses dimensions of life that are particularly affected by MND such as social and emotional function but it is unclear whether the ALSAQ-40 would fully reflect the impact of telehealth (41).

Data from quantitative elements of a randomised controlled trial will not, in isolation determine which outcome measures best reflect the impact of the intervention. It would also not fully explain how the TiM system would be used in the real world and what factors would influence its adoption and success. Utilising mixed methods will allow the combination of quantitative data with more in-depth results from qualitative interviews that will explore participants' experiences in more depth. It will also allow explanation of outcomes that occurred (particularly those that were unexpected) and understand why (and in what context) aspects of the system were successful or unsuccessful which could lead to improvements in the TiM system.


For peer review only - http://bmjopen.bmj.com/site/about/guidelines.xhtml

## 6. Study Methodology

We will conduct a randomised controlled pilot trial comparing the TiM telehealth service and standard care with standard care alone. The intervention and follow-up period will be a minimum of 6 months. Quantitative data will be collected at 0, 3 and 6 months then every six months until the patient finishes the trial. Qualitative data will be collected at baseline in the control arm and at 1 and 6 months for a selection of patients in the intervention arm.

### 6a. Participant recruitment and selection

Pre-screening will identify a list of potential patients who cared for by the Sheffield Teaching Hospitals MND care centre clinic as part of usual care using the MND care centre "ARC" clinical database. Each patient will be assigned a number. The Clinical Research Facility at the Royal Hallamshire Hospital, Sheffield Teaching Hospitals NHS trust will generate random numbers to identify the patients to invite. These patients will be sent a letter of invitation to participate. This will be accompanied by patient and carer information leaflets and a return slip to indicate their interest. Those who do not return the slip will be followed up by telephone or at clinic, if appropriate a minimum of once and a maximum of twice. A log will be kept in order to complete the CONSORT diagram (Appendix 2) (42).

Patients and their primary carer who express an interest will be invited to discuss the trial in a face-to-face meeting with the PI and also via telephone with their consultant neurologist (Dr. Christopher McDermott or Professor Dame Pamela Shaw, Sheffield Teaching Hospitals MND Care Centre). The Sheffield MND Care Centre sees approximately 120 new patients with MND per year. At any time there are approximately 300 patients attending clinic. We expect to be able to recruit a minimum of four patients per month.

Participants in the intervention arm will be invited to participate in qualitative interviews, conducted at month one and month six. Purposive sampling will be used to reflect the variation and predefined patient prognostic factors thereby capturing a range of experiences. Interviews will continue until data saturation is reached or 20 interviews have been conducted. All participants assigned to the control arm will be invited to be interviewed after completion of the baseline questionnaires to determine the feasibility and acceptability of these measures and their views on participating in the trial. The qualitative component will provide information not easily obtained from questionnaires that will facilitate understanding of the intervention from the perspective of all stakeholder groups.



For peer review only - http://bmjopen.bmj.com/site/about/guidelines.xhtml

## 6b. Consent

Following indication of their interest to participant potential participants will be met at a mutually agreeable location, preferably the patients' home. They will have further opportunity to discuss the trial with the PI and decide whether they wish to participate. Willing participants will be asked to give informed written consent or use an appropriate witnessed alternative (which may include verbal consent or via a communication device) for screening and involvement in the trial. Carer consent will be obtained by full written consent.

In versions prior to V1.5 of the protocol both patient and carer consent were required. V1.5. has amended the inclusion criteria to allow a patient to participate with carer participation.

If one or both consent to the study a member of the study team will initiate the screening process. Participants will be screened and recruited by the PI according to the CONSORT principles and Good Clinical Practice (42, 43).

Those who decline participation will be invited to give their reasons in order to identify common factors; this may help recruitment strategies and identify potential problems for compliance. Basic anonymised details of these patients (age, gender, reason for exclusion) will be collected on all eligible patients in order to fulfill the CONSORT flow chart (Appendix 2) (42, 43).

## 6c. Randomisation

Once recruited, randomisation will be performed using the independent web-based system http://www.sealedenvelope.com using block randomisation. All patients and carers will be assigned an anonymous individual study code, and a recruitment log held by the research team in SITraN.

For peer review only

**6b. Inclusion and exclusion criteria**

Inclusion criteria:

- Patients aged 18 years or over who have attended the MND clinic at the Royal Hallamshire Hospital, Sheffield.
- Patients with amyotrophic lateral sclerosis diagnosed by a consultant neurologist with symptom onset within the last three years.
    - Or
- Patients with amyotrophic lateral sclerosis, primary muscular atrophy or progressive lateral sclerosis diagnosed by a consultant neurologist with a deterioration in their condition as evidenced by a deterioration in the ALS functional rating score (ALSFRS-R) by at least two points during the previous 18 months.
- Live within 120 minute drive from Sheffield

Exclusion criteria:
The main circumstances where patients or carers will be excluded are those in which individuals would be unable to use the telehealth system or give informed consent.

- Patients attend another MND care centre in the UK.
- Significant impairment in decision making capacity preventing informed consent by the subject due to a major mental disorder including fronto-temporal dementia.
- Patient unable to use the TiM system due to physical, intellectual or language difficulties and unwilling to permit carer to operate it on their behalf. Patients will be asked to complete two questions used within the TiM system, with, or without the help to their carer to verify their ability to use the system.
- ~~The patient has no eligible informal carer willing to participate in the trial~~ (V1.5)
- Insufficient mobile telephone reception in the patients' home to use the TiM system.
- Any other major impairment that may affect their ability to participate in the study

Carer inclusion criteria

- Age 18 years or older
- Person identified by the patient as the major provider of informal care (emotional and/or practical support) to the patient and provides more than one hour per week of unpaid care
- Carer willing to allow data they provide during the trial to be shared by the research team with their own doctor in the event of serious clinical need.

Carer exclusion criteria

- Significant decision making capacity preventing informed consent due to a major mental disorder.
- Carer unable to use the TiM system due to physical, intellectual or language difficulties. Carers will be asked to complete two questions used within the TiM system to verify their ability to use the system.


For peer review only - http://bmjopen.bmj.com/site/about/guidelines.xhtml

- Inability to participate in the study due to other major physical or mental illness or language difficulties.
- Professional carers receiving direct payment for their services.

## 6e. Withdrawal

Participants will be followed up until the end of the study, death, or withdrawal. Those wishing to withdraw will be given the opportunity to speak to a member of the study team. Participants are free to withdraw from the intervention or study at any time. As a pilot study, importance of understanding reasons for withdrawal is recognised. This will be explained to the participants in the information leaflets. The importance of understanding reasons for withdrawal and the characteristics of these participants is recognised given the nature of the study. This will be explained to the participants in the information leaflets.

**Withdrawal criteria**

1. Patient request
2. Carer request
3. Patient loses capacity to continue to provide consent

If a Patient withdraws from the study arrangements will be made for the equipment to be collected or returned. Where appropriate participants will be invited to give the reasons for withdrawal.

Patients will also be given the option of:

1. Withdrawal from the intervention but remain within the study. Study data will only be collected at clinic visits at 3 and 6 months and six monthly until the end of the study.
2. Withdrawal from the study. Unless the participant objects, any data collected up to this point would be retained and used in study analysis. Participant agrees to allow contact to give safety and survival data.
3. Withdrawal from the study entirely. Unless the participant objects, any data collected up to this point would be retained and used in study analysis. If the participant does not wish to be contacted with regard to safety or survival data, no further contact with regard to this study will be made.

In the event that the patient dies or loses the capacity to provide consent they will be withdrawn from the trial but any data collected up to that point would be retained and used in study analysis. Carers would also be withdrawn at this point.

If the carer participant withdraws the patient can opt to continue to use the system or withdraw. If appropriate, carers will be invited to give reasons for withdrawal.

## 6f. Compliance

Steps have been taken to encourage compliance with the weekly schedule. Patients will receive regular messages on the system to invite them to complete a scheduled telehealth session. They will receive feedback on their compliance record and an


For peer review only - http://bmjopen.bmj.com/site/about/guidelines.xhtml
For peer review only

encouraging message at the start of each visit. Those who fail to complete the session within one day will be reminded by text and via the telehealth system. If after two weeks they do not enter data they will be contacted by the PI and offered more support and training. Compliance data will be analysed as part of the process evaluation.

Compliance with the patient reported outcome measures would be monitored by the CRF nurse. She will contact the participants to support data collection, identify and chase missing data and feedback to the PI. In the event of missing data the CRF nurse will telephone the patients/carer after two weeks. She will contact them a minimum of twice and a maximum of three times to chase the data. A contact log will be kept.

**6g. Sample size**

The study aims to recruit a total of 40 patients and their carers. 20 patients and their primary carer will be randomised to the intervention arm (a minimum of 6 months use of the TiM telehealth plus usual care) and 20 patents and their carer in the control arm (usual care).

Since the proposed trial is primarily an assessment of the acceptability of the intervention and the feasibility of a full trial, the proposed sample size is not based on standard statistical parameters such as a clinically relevant difference between groups. Instead, the sample size is justified on the grounds of quantifying patient variance (i.e. the standard deviation) in the proposed outcome measures (in particular quality of life measures) and on feasibility of the full trial, as follows:

- A sample size of 40 patients allows a standard deviation to be estimated to within a precision of ±20% of its true underlying value with 90% confidence. This estimate will be synthesised with standard deviations observed in other published studies (e.g. (41, 44-47) and on-going trials within SITraN (48, 49), to provide a robust estimate for use in the sample size calculation for the full trial.

- Given the rarity of MND, any definitive study will be infeasible if the required sample size is substantial. Assuming the upper limit for feasible UK study is around 200-300 patients in total, it follows that the full study would need powering to detect a standardised effect size of at least 0.4 SDs. This pilot trial will provide a preliminary assessment of whether the intervention might feasibly achieve this, and inform the choice of outcome measures for the proposed full study.

This sample size is also in keeping with the proposal of 12 evaluable patients per arm in a pilot study (after withdrawal or drop-out) (50).

**6h. Blinding**

The PI will not be blinded to the randomisation as they are responsible for training the participants to use the TiM system and any on-going technical or training requirements. The treating clinicians will not be blinded to the arm of the intervention as they are responsible for the clinical care of the patient and reviewing the data and the Shadow Monitoring System.



For peer review only - http://bmjopen.bmj.com/site/about/guidelines.xhtml

The PI will enter screening baseline data. Participant reported outcome measures after this will be collected by an independent research nurse from the Sheffield Teaching Hospitals Clinical Research Facility who will facilitate collection of these surveys by post or, if preferred by the patient, in person. They will enter the details into the study database.

Following the end of the trial and database lockdown the PI will analyse the two groups of data whilst remaining blinded to the allocation of the two groups. The STH CRF will hold the database code to identify the allocated groups.

The PI will conduct the qualitative interviews and collect the system use data and will not be blinded to these measures.

## 7. Study treatment

### Standard clinical care – Intervention arm and Standard care arm

Usual clinical care will continue throughout for participants in both arms of the study. All participants will continue to be invited to the Sheffield MND Care Centre Clinic for routine review. They will be seen by their consultant neurologist and the MND multidisciplinary team, according to their routine two- to three-monthly schedule. All patients will have access to the MND telephone helpline provided by the Sheffield MND care team.

### The TiM system - Intervention arm

Those in the intervention arm will use the TiM system, in addition to standard clinical care. All necessary hardware, software, data transfer and support costs for the TiM system will be met by Abbott Healthcare Products Ltd in collaboration with Carematix.

Patients will be provided with a TiM patient hub: a handheld, touch screen Samsung Galaxy tablet computer that communicates with the MND specialist nurse's TiM clinician system at the Sheffield MND Care Centre. Patients will be asked to use the system at least weekly. Each week the telehealth hub asks the patient a series of questions to detect common problems found in MND, such as worsening mobility, or swallow, symptoms of depression, anxiety, pain, saliva and spasms. Some of the questions closely match validated scoring scales (e.g. the ALS Revised Functional Rating Score (51) and the depression and anxiety short screen: PHQ-4) but others have been specifically designed by the clinical team to be used in the telehealth system. The TiM system also enquires about symptoms of respiratory insufficiency and infection, nutrition and social care. Patients using specialist equipment, such as non-invasive ventilation or gastrostomy tubes will additionally be asked to report problems related to the intervention. The TiM system can also weigh patients weekly and monitor patients' overnight oximetry using established telehealth monitors.



For peer review only - http://bmjopen.bmj.com/site/about/guidelines.xhtml

Carers will also be asked to complete a weekly telehealth session in order to monitor their well being. The TiM system includes a carer strain screen and the PHQ-4 depression and anxiety screen. There is also the opportunity with the telehealth system for patients or carers to trigger an adhoc session if issues arise during the week about which they wish to inform the centre. Interspersed through the questions are educational messages and users have access to a bank of educational resources within the hub.

The patient and carer responses are transmitted (via an encrypted 3G mobile signal) to the Carematix server. The responses undergo immediate computational analysis, using pre-determined clinical algorithms, which assigns an alert level to each response. This limits the amount of nurse's time required to use the system. An automated acknowledgement is sent back to the user indicating whether to expect contact from the MND centre based on the results and the timescale for the response.

Each day the MND nurse will log into the TiM system and will be presented with the responses from all patients using the TiM system. They will be automatically alerted to any important changes. Urgent alerts include any new and severe symptom or any new problem that poses a major risk to the patient or carer (e.g. choking, falling, respiratory insufficiency). Routine alerts include any other deterioration in the patient's ALSFRS-R or any new symptom. An appropriately timely response will be made to each alert level. Patients will be reminded to seek urgent medical attention in an emergency.

The information on patient status may facilitate rescheduling of appointments according to patient need rather than the fixed intervals used at present (e.g. the appointment could be delayed if the patient is well, and the TiM system has activated no new alerts). As the feasibility and safety of the TiM system has not been previously evaluated, during the TiM trial patients will continue to attend routine clinic appointments and no patient will have their clinic delayed. The feasibility and safety of rescheduling appointments will be examined using a shadow monitoring protocol (detailed later).

Patients and carers will undergo a training session and a follow-up telephone call after two weeks. The TiM system has been designed to be user friendly and to encourage compliance with the weekly sessions. Face-to-face training with the hub system will be offered at the start of the intervention. Support will be available throughout the trial in the hub. Compliance will be monitored and should patients not complete the TiM system for three weeks in a row, contact will be made to offer more training or support.

The TiM system has been designed by the applicant in collaboration with her supervisors, the Sheffield MND team, Abbott Healthcare Products Ltd. and Carematix. Carematix have experience in delivering similar home telemonitoring systems in other diseases. In developing the system, expertise has also been sought from those developing telehealth services in other diseases in the University of Sheffield and NIHR CLARHC for South Yorkshire. These included the School of Health and Related Research (ScHARR) SMART consortium (Self Management supported by Assistive, Rehabilitation and Telecare technologies), NIHR CLAHRC SY Telehealth & Care Technologies (TaCT) for Long Term Conditions theme, and Devices for Dignity (52).



For peer review only - http://bmjopen.bmj.com/site/about/guidelines.xhtml

For peer review only



For peer review only - http://bmjopen.bmj.com/site/about/guidelines.xhtml

## 8. Data collection

### 8a. Quantitative data collection

Data collection will occur at baseline, three months and six months at the end of the study. Participant data will be completed using postal and telephone questionnaires to minimize patient burden and cost. The study will continue for a minimum of six months. Follow-up will continue until the last participant has used the system for six months. The maximum proposed follow-up will be 18 months.

**Patient measures**
Baseline measures:

- Age
- Gender
- Experience with technology (frequency of use of a computer, tablet or smart phone)
- Major health condition that could impact on the use of telehealth (including mood disorder, other symptomatic chronic disease)
- Medication

Outcome measures will be collected at 0, 3 and 6 months, then every six months until the end of the study and finally at the end of the study:

- Quality of life measures
  - ALSAQ-40 (an MND disease specific quality of life score (41))
  - SF-36-RAND
  - EQ-5D+D (EQ-5Q-3L with a dignity bolt-on)
- Clinical outcomes
  - ALSFRS-R (an MND disease specific functional rating score (51)
  - Pain score (modified Likert scale)
  - CSS-MND Saliva Severity Scale (designed for use with MND patients) plus global change scale
  - Hospital Anxiety and Depression Scale
- Health resource usage questionnaire
- Patient experience questionnaire



For peer review only - http://bmjopen.bmj.com/site/about/guidelines.xhtml

**Carer measures**
Baseline measures:
- Age
- Gender
- Frequency of use of a computer, tablet or smart phone
- Major health that could impact on the use of telehealth
- Relationship to patient
- Number of hours spent per week providing care for patient

Outcome measures will be collected at 0, 3 and 6 months and at the end of the study:
- SF-36 RAND
- 12 item Zarit Burden Inventory (53)
- Hospital Anxiety and Depression Scale (54)
- Carer satisfaction questionnaire

Data will be collected to evaluate the conduct of the trial including:
- Participant compliance with the weekly telehealth session
- Rate of completion of outcome measures
- Rates of recruitment and withdrawal
- Participant actual and perceived time burden associated with the system
- Time spent by the MND nurse using TiM system and responding to alerts or queries generated by the system.


For peer review only - http://bmjopen.bmj.com/site/about/guidelines.xhtml

## 8b. Qualitative sub-study

**Intervention patient and carer interviews**

Qualitative semi-structured interviews will be conducted with patients and carers in the intervention arm. Participants randomized into the intervention arm will be invited to take part in interviews. Baseline interviews will occur at one month after the intervention is started. A further interview will be conducted at 6 months. Six months is considered an appropriate timeframe for patients to become familiar with the intervention and its impact on quality of life.

Interviews will be conducted until data saturation is reached. The interviews will draw directly upon peoples' own experience and views, within the context of everyday lives to explore topics including

- Participants experience and expectations of technology
- Participants' expectations of telehealth services
- Barriers and aids to recruitment
- Compliance with the TiM system
- How the TiM system is used at home by patients and their carers
- The impact of using the TiM system on their lives and well-being
- The impact of education on their day-to-day lives
- The experiences of carers monitoring
- Whether the outcome measures used capture the changes in participants well being associated with using the TiM system.
- How the system would be used outside a trial

The early phase interview will explore participants' expectations of technology and the TiM system, the views on the system, their experiences of training and using the equipment. The later phase will explore further how the TiM system influenced their care and quality of life, mental well being as their condition changed. It will also identify barriers and facilitators to adoption of the TiM system.

The applicant will agree pre-defined topic schedules (see Appendix B) developed from the literature, expert consensus and discussion with the trial management group with supervision from Dr. Wendy Baird, an experienced qualitative researcher (School of Health and Related Research, Sheffield University). The PI will conduct interviews in the participants' home. The PI will conduct qualitative interviews until data saturation is reached (55). Interviews will be audio- recorded, transcribed verbatim and analysed with coding and retrieval of data supported by NVivo software.

Due to the nature of MND consideration will be given to participants' needs. The research team has experience in conducting qualitative interviews with patients and carers and these interviews will be conducted in a similar fashion. Often patients with MND prefer to be interviewed with their carer. This also aids communication where patients have speech difficulties and allows participants to support each other whilst discussing sensitive issues. Patients can use communication devices and all participants will be provided with a brief topic guide prior to the interview to facilitate participation for those with communication difficulties. Interviews will be limited to approximately one hour to reduce burden and fatigue. If participants prefer to be interviewed together carers will also be offered separate interviews where possible.



For peer review only - http://bmjopen.bmj.com/site/about/guidelines.xhtml

Field notes will also be collected by the PI during the face-to-face training using the TiM system to determine participants' early reactions to using the system and their needs for training.

**Control group interviews**

Following randomization, those patients and carers who are assigned the control arm will complete the baseline questionnaires. They will then have a short (15-20 minute) semi-structured interview with the PI. This will focus on their experiences and opinions of the baseline questionnaires. It will examine whether they were easy or difficult to complete, whether they were acceptable or caused distress to complete and which questions most reflected their condition and current quality of life.

The interviews, topic guides and analysis will be conducted in the same way as described in the previous section. Topic guides will not be provided before the interview but participants invited to submit any further comments to the research team either in writing or telephone following the interview. It is expected that patients and carers will be interviewed together. Interviews will continue until data saturation is reached or a maximum of 10 interviews conducted.

**Staff interviews**

At least five staff that care for the participants will undergo one-to-one semi structured interview by the PI during and at the end of the intervention. This will include the two responsible consultant clinicians (Dr Chris McDermott and Professor Pamela Shaw), at least one MND specialist nurse who has used the telehealth system and two members of the MND community team who have cared for participants. They will allow them to draw on their experiences of the TiM system in more depth. A staff information leaflet will be provided and written consent will be required prior to any interview.

Topics will include

- The day-to-day use of the TiM system
- The impact of the TiM system on clinical care of patients and carers
- The safety and accuracy of the system
- Barriers and aids to adoption of the TiM system.
- Views on amending the appointment schedule

These will be planned and conducted in the same manner as the participant interviews under the supervision of Dr Wendy Baird. An interview with the MND nurse and clinicians using the system will be scheduled early in the trial to capture any problems with training and set up of the system. At the end of the trial further interviews will be held with the MND team as described above.

Following the interviews a focus group with the clinical team will be held to draw together all the information gathered from the patient and staff interviews. It will be chaired by Dr Wendy Baird, independent qualitative researcher, transcribed and analysed by the PI under her supervision.


For peer review only - http://bmjopen.bmj.com/site/about/guidelines.xhtml

The qualitative findings will facilitate the exploration of any issues and challenges, which may arise from using TiM from the perspective of all stakeholder groups. The findings will enhance understanding of the feasibility of using TiM and assist with the interpretation of the clinical data from the perspective of patients and clinicians.

## 8c. Shadow monitoring protocol

In order to determine the safety of a remote monitoring system that may enable clinicians to make decisions regarding a patient's management the trial will also collect data on clinicians' opinion on the accuracy of the data displayed by the TiM system. This is referred to as the shadow monitoring protocol.

Prior to each patient's face-to-face visit (depending on their appointment schedule) the treating MND doctor will be asked to conduct a remote assessment of the patient by reviewing the TiM system clinical information. They will be asked to indicate, given the information provided by the TiM system, whether they would change their patient's appointment. The patient would attend the appointment as scheduled and after the appointment the clinician would be asked whether the appointment schedule time was correct. They would also be asked to indicate whether they felt that the information displayed on the TiM system was a safe and accurate reflection of the patient's condition and whether it influenced their clinic visit. Clinicians will also indicate whether the TiM system had affected the consultation. They will also report any adverse events identified. Should patients be unable to travel to clinic they will be offered a telephone consultation at the usual scheduled time. The same Shadow Monitoring questions and need to report adverse events will apply.

The results of this shadow monitoring will be triangulated with the qualitative sub-study and will influence the later interview topic guide.

## 8d Process evaluation

Data regarding the TiM system use by patients, carers and staff will be will be collected in order to understand how the system could be used in the NHS MND care process. It will also collect data regarding the extra time and resources required to manage the problems generated by the TiM system. It will be triangulated with data gained from the qualitative sub-study, adverse event log and shadow monitoring protocol.



For peer review only - http://bmjopen.bmj.com/site/about/guidelines.xhtml

## 9. Analysis

The PI will conduct analysis with regular supervision from the TMG.

### 9a. Feasibility and quantitative analysis

The feasibility of a full trial will be determined by analysis of

- Recruitment rates
- Retention rates
- Compliance rates
- Sample size calculations as detailed above

The safety, acceptability and feasibility of use of the TiM system

- Incidence of adverse events (clinical and related to the TiM system functionality)
- Information collected using the Shadow Monitoring process
- Qualitative data analysis

The PI and the CRF study nurse will be responsible for chasing missing data. The CRF nurse is responsible for chasing the questionnaire data and will telephone the patients a minimum of once and maximum of twice to chase unreturned questionnaires or clarify missing data within the questionnaire packs. They will report monthly to the PI. For the main outcome measures, (SF-36 and ALSAQ-40) protocols are provided for managing missing data if necessary. Participants who withdraw will be encouraged to continue to be followed up and reasons for withdrawal ascertained where possible. In the proposed larger, efficacy trial intention to treat analysis will be adopted.

Quantitative analysis will be undertaken in a similar manner for all endpoints. The change from baseline at each time point will be analysed using analysis of covariance in which the covariates are treatment group and the baseline value. For instance, the change in ALSFRS-r at six months will be analysed with treatment group and baseline ALSFRS-r as covariates. The mean (standard deviation) change in each group, the difference between groups and its associated 95% confidence interval will be reported. No formal hypothesis testing will be undertaken for this pilot study.

For peer review only

## 9b. Qualitative analysis

Data from the interviews will be recorded, transcribed and undergo Framework analysis (56). Although Framework analysis was developed for applied policy it has proved useful in applied health research. Analysis will be ongoing and iterative involving concurrent data collection and analysis, with systematics efforts to check and refine developing categories of data. Themes and hypothesis identified in the early phases of data collection will inform the areas of investigation in later interviews. Regular meetings with supervisors will review the data analysis, explore respondents' underlying reasoning, discuss deviant cases and reach agreement on recurrent themes and findings. The PI's field notes and reflexive diary will also be reviewed and used to inform the analysis of qualitative data. Dr Wendy Baird, an independent, experienced qualitative research, will supervise this stage of the work.

Results from the qualitative analysis will be triangulated, for example, to explore the reasons why problems with the trial methodology or TiM system have occurred. Both themes and anonymous verbatim comments will be published to demonstrate the findings.

For peer review only



For peer review only - http://bmjopen.bmj.com/site/about/guidelines.xhtml

**10. Data entry, security and confidentiality**
Clinical quantitative data input will be the responsibility of the PI (baseline) and CRF study nurses (months 3 and 6, and at the end of the study). Data quality will be the responsibility of CRF nurses and PI who will report back to the TMC and TSC. The qualitative data and system usage data will be the responsibility of the PI. Data (including audio-recordings) will be collected and retained in accordance with the Data Protection Act 1998 and Caldicott Principles. Anonymised study data will be entered onto a validated database system designed to an agreed specification between the PI and Sheffield CTRU and securely stored on the SU intranet. The PI and the CRF research nurses will have access to data on the database through the use of usernames and encrypted passwords. Study documents will be retained in a secure location during and after the study has finished.

All source documents will be retained for a period of at least 5 years following the end of the study, as per the CTRU SOP. Where study related information is documented in medical records those records will be retained for at least 5 years after the last patient last visit

The data provided through the TiM system will be collected using a secure web-app accessed by the participants by a unique username and password. It will be stored on a secure server that will be available through a web-portal hosted by Carematix to the clinical team using secure usernames and password.

For the purposes of the trial each participant will be given a unique TiM system code. This will allow all data to be relayed through the web-app without any associated patient identifiable features. This code will be held separately and stored securely on the STH intranet to allow individual identification by the MND care team. The clinician will display only the anonymous code. This will be accessed through a secure portal with usernames and passwords. No identifiable information will be stored on the patient hub or on the TiM server. The technology providers will have no access to patient identifiable information. Any technology problems will be dealt with by the research team and participants will have no contact with the technology providers.

The system has a full electronic audit trail and will be regularly backed up and will be held in a way that conforms to STH information governance procedures.

**Access to source data**
Monitoring and audit by the relevant health authorities will be permitted by the sponsor. These include the Research Ethics Committee and local R&D departments. The sponsor will be allowed to monitor and audit the study at each site and be allowed access to source data and documents for these purposes.



For peer review only - http://bmjopen.bmj.com/site/about/guidelines.xhtml

## 11. Safety and safety assessments

We do not envisage any serious safety or adverse events associated with the intervention. The system does not give individual advice to a patient or recommend change in management without input from a clinician. The trial protocol requires patients to continue with their usual care including planned outpatient appointments and the Shadow Monitoring Protocol will evaluate whether the data provided by the TiM system is felt to accurately reflect the patients' clinical condition. The responsible clinician who is a consultant neurologist with specialist experience in MND and research will continue to review the patient on a regular basis (unless the patient is unable to attend clinic) and will have overall responsibility for their care throughout the trial. The specialist MND nurses using the TiM system have extensive experience in managing patients via the existing MND helpline.

The database will automatically alert the trial manager to any carer scoring 11 or more of the Hospital Anxiety and Depression score collected as part of the outcome measures. This will allow the trial manger to identify those carers who may require further support.

**Adverse Event Reporting**

All adverse events will be reported in accordance with the Sheffield CTRU Adverse Event and Serious Adverse Events SOP.

Participants will be monitored for adverse clinical events and efforts will be made to ascertain whether the TiM system influenced the event or could have predicted the event. These include unplanned admissions and deaths. Non-clinical events relating to the use of the telehealth hub will recorded e.g. failure to record or deliver information to and from the clinical interface.

In research other than CTIMPs an adverse event is defined as: is any unfavorable and unintended sign (including an abnormal laboratory finding), symptom, or disease having been absent at baseline, or, if present at baseline, appears to worsen AND is temporally associated with medical treatment or procedure, **REGARDLESS** of the attribution (i.e., relationship of event to medical treatment or procedure).

**Serious Adverse Event (SAE)**

In research other than CTIMPs, the National Research Ethics Service defines a Serious Adverse Event (SAE) is defined as an untoward occurrence that:
(a) results in death;
(b) is life-threatening*;
(c) requires hospitalization** or prolongation of existing hospitalization**;
(d) results in persistent or significant disability or incapacity;
(e) consists of a congenital anomaly or birth defect; or
(f) is otherwise considered medically significant by the investigator.

*"life-threatening" in the definition of "serious" refers to an event in which the patient was at risk of death at the time of the event; it does not refer to an event which hypothetically might have caused death if it were more severe. **Hospitalisation is defined as an inpatient admission, regardless of length of stay, even if the



For peer review only - http://bmjopen.bmj.com/site/about/guidelines.xhtml

hospitalisation is a precautionary measure for continued observation. Hospitalisations for a pre-existing condition, including elective procedures that have not worsened, do not constitute an SAE.

**Adverse event exclusions**
The only adverse event that will be excluded is:
1. Standard or expected disease progression.

**Adverse event inclusions**

All serious adverse events will be reported. These include deaths of participants and emergency admissions. We will attempt to determine whether the use of the TiM system contributed to the event, in particular whether there was any delay in seeking help due to the use of the system.

**Assessment of Adverse Events**
The following criteria will be used when assessing adverse events: Intensity (severity):
Mild - does not interfere with routine activities
Moderate - interferes with routine activities
Severe - impossible to perform routine activities

Relationship to the study treatment:
Unrelated - There is no evidence of any causal relationship. N.B. An alternative cause for the AE should be given
Unlikely - There is little evidence to suggest there is a causal relationship. There is another reasonable explanation for the event (e.g. the participant's clinical condition, other concomitant treatment).
Possible - There is some evidence to suggest a causal relationship. However, the influence of other factors may have contributed to the event (e.g. the participant's clinical condition, other concomitant treatments).
Probable - There is evidence to suggest a causal relationship and the influence of other factors is unlikely.
Definite - There is clear evidence to suggest a causal relationship and other possible contributing factors can be ruled out.
Not assessable - There is insufficient or contradictory information which cannot be supplemented or verified

**Reporting procedures**

All study participants will be encouraged to contact and inform their site research team if they experience any new medical problem or are admitted to hospital. Those that are not picked up through general contact will be identified at their routine 2-3 monthly outpatient appointments either in person or by telephone as part of the Shadow Monitoring Protocol. The patients' consultant neurologist (Dr. Chris McDermott or Professor Dame Pamela Shaw) will enquire about any adverse events since the previous visit and record these on the adverse event paper CRF and database. For any Serious Adverse Events an SAE paper CRF and database entry will be completed. The PI and consultant neurologist will assess the event and the CRF will be kept in the site file. Serious adverse events will be reported to the TSC, TMG and the sponsor if deemed by


For peer review only - http://bmjopen.bmj.com/site/about/guidelines.xhtml
For peer review only

either to be related to the trial. Reports of related and unexpected SAEs will be submitted to the ethics committee within 15 days of the chief investigator becoming aware of the event. This will use the National Research Ethics Service Report of Serious Adverse Event form. Information will also be included in the routine progress reports to the sponsor and ethics committee. Routine safety data and all SAEs that the TMG or TSC deems to be related to the trial will also be reported to the technology provider in the same manner.

Any suspected adverse drug reaction would be assessed and reported to the MHRA as part of clinicians' routine pharmacovigilance responsibilities using the Yellow Card Scheme. The technology provider, Abbott Healthcare Products Ltd. manufactures a number of drugs (listed in Appendix A). Any suspected adverse drug reaction involving an Abbott Healthcare Products Ltd. drug would also be reported to the manufacturer in the same manner, within 24 hours of receipt by the CI or the next working day for reports received out of hours. Such reports should be sent to ukpharmacovigilance@abbott.com. On a monthly basis the PI will send a reconciliation list of all the reports sent to the Abbott Pharmacovigilance Department within that month to ensure that all the appropriate information has been exchanged. Should any discrepancies arise, both parties will immediately seek to resolve them.

For peer review only

## 12. Ethical considerations

The study will be conducted in accordance to Good Clinical Practice Guidelines and subject to Research Ethics Committee favourable opinion. The study received a favorable approval from an independent panel representing the NIHR, which funds Dr. Esther Hobson's NIHR Doctoral Fellowship Award.

The study has approval from the Sheffield Teaching Hospitals NHS Foundation Trust's Research and Development department. It has also received favourable review from Dr. Mike Bradburn, study statistician at ScHARR, Dr. Cindy Cooper, director of CTRU and ScHARR, and Dr. Wendy Baird of the Yorkshire and Humber Research and Design service, Professor Alicia O'Cathain, Professor of Health Services Research, ScHARR and Professor Dame Pamela Shaw, SITraN. The application will be submitted through the IRAS central allocation system. The approval letter from the ethics committee and copy of approved patient information leaflet, consent forms, CRF's and questionnaires will be present in the site files before initiation of the study and patient recruitment.

It is recognized that patients with MND may be frail and nearing at the end stages of their lives. The research team has extensive experience in conducting clinical trials in this population. The study design has attempted to limit the burden imposed by the study by avoiding unnecessary study visits (by combining them with scheduled visits), collecting data in the participants' homes at their convenience and limiting the study procedures to the minimum necessary. The intervention has been designed in collaboration with patients and carers to maximize ease of use and minimize impact on participants' lives. It is appreciated that there are a number of questionnaires that require completion. Given one aim of the study is to determine the most appropriate outcome measures to evaluate efficacy of the TiM system there are more questions than would be used in a large scale trial. These have been reviewed by the Sheffield MND Research Advisory Group (the local PPI group) and the lay members of the TSC (David Stelmach) and TMG (Anne Quinn) to ensure acceptability. Participants will be supported by the CRF nurse to complete these at their convenience in a manner selected by the participant (either by post, telephone or in person).

There are other clinical studies ongoing in the Sheffield MND care centre. Involvement in other studies would not preclude patients from entering this study. Consideration of the burden involved in the study, potential impact on the outcome of the study and the patients' expressed priorities will be considered before patients are approached to be involved. If involvement in this study excludes patients from entering another clinical trial patients will be given the option to withdraw from this study.

The potential conflict of interest between the role of the clinical team in caring for patients and their role as researchers is recognized. The study design has considered the impact of this conflict on the participants choices and also any potential bias. Whilst PI is a doctor working within the MND team she is a specialty training registrar and overall responsibility for the patients' clinical care will remain with the consultant neurologist rather than the PI. Whilst she may have already cared for potential participants, following an invite to participate in the trial will no longer see these patients in their routine clinical appointments and her role will be as a researcher.


For peer review only - http://bmjopen.bmj.com/site/about/guidelines.xhtml

The dual role of the PI as a doctor and researcher has been previously evaluated. The professional background of a doctor may actually aid the building of a research relationship, allow patients to be more open and comfortable with discussing their health with someone who they already trust (56). In order successfully identify any potential bias the purpose of the research and nature of the PI's role will be emphasized throughout the study, the PI will keep a reflexive diary and field notes and identify any potential bias. Where bias is most likely, i.e. in the collection of outcome measures steps have been taken to limit this: the quantitative outcome measures will be collected by an independent study nurse and the qualitative interview structure and topic guides have been planned with supervision from an independent researcher Dr. Wendy Baird. The PI will be supervised, as part of her PhD by independent academics: Dr. Cindy Cooper and Dr. Mike Bradburn (focusing mainly on the trial methodology and conduct, and quantative data analysis), Dr. Wendy Baird and Professor Sue Mawson (qualitative work and service evaluation). If, during the research, participants identify any medical problems, the PI has a duty of care and will make arrangements to deal with these problems. This might involve signposting them to appropriate services or liaising with the clinical team. A log of these activities will be kept and reviewed by the TMG.

When the participants have prior knowledge of the researcher they may feel a sense of duty and feel pressurized to participate (57). Ground rules, informed consent, confidentiality, freedom to stop and what to expect will be discussed with all participants. Participants will be approached by letter and they will be required to contact the study team allowing them to consider the trial in detail first. It will be explained to the patient (both verbally and in the information leaflets and consent forms) that participation is voluntary and will not affect their ongoing care. The information leaflet differentiates the research process and their usual care. It will be made clear, particularly in the interview phase that the PI's role is as a researcher and the aim of the study is to critically analyse service provision and that whilst comments, particular negative comments, will be passed back to the care team they will treated with confidence and respect.

Whilst the carer participant is not a patient of the Sheffield MND team the research team have a duty of care to the carer. There may be circumstances where the carer may disclose information that requires medical care, for example disclosing symptoms of depression or anxiety. At the start of the trial the carer participants' GP will be informed of the trial. In the event of a serious risk being identified the research team will discuss this in confidence with the carer and make arrangements to resolve the problem. This might include referral to his or her own GP or other health professional. Carer participants will be informed of these procedures in the Carer information leaflet and consent form. Confidentiality will be maintained in accordance with the General Medical Council's guidance on Confidentiality (58).

Upon publication of the qualitative interviews it may be possible to identify participants' comments although this will be avoided if possible. This is explained to participants in the Interview Information Leaflets and on the consent form.


For peer review only - http://bmjopen.bmj.com/site/about/guidelines.xhtml

## 13. Finance and indemnity

The trial has been financed through an NIHR doctoral fellowship grant and details have been drawn up in a separate agreement.

This is an NHS sponsored study. If there is negligent harm during the clinical trial when the NHS body owes a duty of care to the person harmed, NHS indemnity will cover NHS staff, medical academic staff with honorary contracts and those conducting the trial.

The University of Sheffield has in place insurance against liabilities for which it may be legally liable and this cover includes any such liabilities arising out of this clinical trial.

## 14. Reporting and dissemination

Results of the study will be disseminated in peer reviewed scientific journals and clinical and academic conferences.  Details of the study will also be made available on the SITraN and ScHARR websites, blogs and social media and through local MND groups. Summaries of the research will be updated periodically on the SITraN website to inform readers of the ongoing progress.  Following publication contact with other UK MND care centres will be made to disseminate the findings and assess buy-in potential for a full study if this is appropriate.

For peer review only

## 15. References

1. The neurological alliance. Neuro numbers. A brief review of the numbers of people in the UK with a neurological condition.
2. Whitehead B, O'Brien MR, Jack BA, Mitchell D. Experiences of dying, death and bereavement in motor neurone disease: A qualitative study. Palliat Med. 2012;26(4):368-78.
3. O'Brien MR, Whitehead B, Jack BA, Mitchell D. The need for support services for family carers of people with motor neurone disease (MND): views of curent and former family caregivers a qualitative sutdy. Disabil Rehabil. 2012;34(3).
4. Miller RG, Mitchell JD, Moore DH. Riluzole for amyotrophic lateral sclerosis (ALS)/motor neuron disease (MND). Cochrane Database Syst Rev. 2012;3:CD001447.
5. Bourke SC, Tomlinson M, Williams TL, Bullock RE, Shaw PJ, Gibson GJ. Effects of non-invasive ventilation on survival and quality of life in patients with amyotrophic lateral sclerosis: a randomised controlled trial. The Lancet Neurology. 2006;5(2):140-7.
6. Aridegbe T, Kandler R, Walters SJ, Walsh T, Shaw PJ, McDermott CJ. The natural history of motor neuron disease: Assessing the impact of specialist care. Amyotroph Lateral Scler. 2012.
7. Chio A, Buffa C, Mutani R, Bora G, and the PARALS. Positive effects of tertiary centres for amyotrophic lateral sclerosis on outcome and use of hospital facilities. J Neurol Neurosurg Psychiatry. 2006;77(8):948-50.
8. Johnston B, Kidd L, Wengstrom Y, Kearney N. An evaluation of the use of Telehealth within palliative care settings across Scotland. Palliat Med. 2012;26(2):152-61.
9. Foley G, Timonen V, Hardiman O. Experience of Services as a Key Outcome in Amyotrophic Lateral Sclerosis (ALS) Care: The Case for a Better Understanding of Patient Experiences. Am J Hosp Palliat Care. 2012;29(5):362-7.
10. Motor Neurone Disease Association UK. MND statistics.
11. Baxter SK, Baird WO, Thompson S, Bianchi SM, Walters SJ, Lee E, et al. The initiation of non-invasive ventilation for patients with motor neuron disease: Patient and carer perceptions of obstacles and outcomes. Amyotroph Lateral Scler. 2012.
12. Foley G, Timonen V, Hardiman O. Patients' perceptions of services and preferences for care in amyotrophic lateral sclerosis: a review. Amyotroph Lateral Scler. 2012;13(1):11-24.
13. The use of non-invasive ventilation at end of life in patients with motor neurone disease. Submitted for publication. 2012.
14. Hecht MJ, Graesel E, Tigges S, Hillemacher T, Winterholler M, Hilz MJ, et al. Burden of care in amyotrophic lateral sclerosis. Palliat Med. 2003;17(4):327-33.
15. Peters M, Fitzpatrick R, Doll H, Playford ED, Jenkinson C. The impact of perceived lack of support provided by health and social care services to caregivers of people with motor neuron disease. Amyotroph Lateral Scler. 2012;13(2):223-8.
16. van Teijlingen ER, Friend E, Kamal AD. Service use and needs of people with motor neurone disease and their carers in Scotland. Health Soc Care Community. 2001;9(6):397-403.
17. McLean S, Chandler D, Nurmatov U, Liu J, Pagliari C, Car J, et al. Telehealthcare for asthma: a Cochrane review. CMAJ : Canadian Medical Association journal. 2011;183(11):E733-42.



For peer review only - http://bmjopen.bmj.com/site/about/guidelines.xhtml

18. McLean S, Nurmatov U, Liu JL, Pagliari C, Car J, Sheikh A. Telehealthcare for chronic obstructive pulmonary disease. Cochrane Database of Systematic Reviews (Online). 2011(7):CD007718.
19. Chua R, Craig J, Wootton R, Patterson V. Randomised controlled trial of telemedicine for new neurological outpatient referrals. 2001;71(1):63-6.
20. Steventon A, Bardsley M, Billings J, Dixon J, Doll H, Hirani S, et al. Effect of telehealth on use of secondary care and mortality: findings from the Whole System Demonstrator cluster randomised trial. Br Med J. 2012;344.
21. Corben S, Rosen R. Self-management for Long-term Conditions. Patients' perpective on the ways ahead. In: Conditions TKsFrML-t, editor. 2005.
22. 3millionlives Campaign. Available from: http://3millionlives.co.uk.
23. Vitacca M, Bazza A, Bianchi L, Gilè S, Assoni G, Porta R, et al. Tele-assistance in chronic respiratory failure: patients characterization and staff workload of 5-year activity. Telemedicine Journal and e-health : the official journal of the American Telemedicine Association. 2010;16(3):299-305.
24. Vitacca M, Comini L, Tentorio M, Assoni G, Trainini D, Fiorenza D, et al. A pilot trial of telemedicine-assisted, integrated care for patients with advanced amyotrophic lateral sclerosis and their caregivers. J Telemed Telecare. 2010;16(2):83-8.
25. Vitacca M, Bianchi L, Guerra A, Fracchia C, Spanevello A, Balbi B, et al. Tele-assistance in chronic respiratory failure patients: a randomised clinical trial. The European Respiratory Journal. 2009;33(2):411-8.
26. Vitacca M, Comini L, Assoni G, Fiorenza D, Gilè S, Bernocchi P, et al. Tele-assistance in patients with amyotrophic lateral sclerosis: long term activity and costs. Disability and rehabilitation Assistive technology. 2012.
27. Pinto A, Almeida JP, Pinto S, Pereira J, Oliveira AG, de Carvalho M. Home telemonitoring of non-invasive ventilation decreases healthcare utilisation in a prospective controlled trial of patients with amyotrophic lateral sclerosis. J Neurol Neurosurg Psychiatry. 2010;81(11):1238-42.
28. de Almeida JPL, Pinto AC, Pinto S, Ohana B, de Carvalho M. Economic cost of home-telemonitoring care for BiPAP-assisted ALS individuals. Amyotrophic lateral sclerosis : official publication of the World Federation of Neurology Research Group on Motor Neuron Diseases. 2012;13:533-7.
29. Sweeney V. Pilot scheme allowed Alan to be nursed at home. The Inverness Courier. 2009 24 Feb, 2009.
30. Nijeweme-d'Hollosy WO, Janssen EPF, Huis in 't Veld RMHA, Spoelstra J, Vollenbroek-Hutten MMR, Hermens HJ. Tele-treatment of patients with amyotrophic lateral sclerosis (ALS). J Telemed Telecare. 2006;12 Suppl 1:31-4.
31. Montes J, Levy G, Albert S, Kaufmann P, Buchsbaum R, Gordon PH, et al. Development and evaluation of a self-administered version of the ALSFRS-R. Neurology. 2006;67(7):1294-6.
32. Maier A, Holm T, Wicks P, Steinfurth L, Linke P, Munch C, et al. Online assessment of ALS functional rating scale compares well to in-clinic evaluation: a prospective trial. Amyotroph Lateral Scler. 2012;13(2):210-6.
33. Rafiq MK, Proctor AR, McDermott CJ, Shaw PJ. Respiratory management of motor neurone disease: a review of current practice and new developments. Pract Neurol. 2012;12(3):166-76.
34. Department of Health U. Supporting People with Long Term Conditions: An NHS and Social Care Model to supprt local innovation and integration. 2005.


For peer review only - http://bmjopen.bmj.com/site/about/guidelines.xhtml

35. McCall K, Keen J, Farrer K, Maguire R, McCann L, Johnston B, et al. Perceptions of the use of a remote monitoring system in patients receiving palliative care at home. Int J Palliat Nurs. 2008;14(9):426-31.
36. McCann L, Maguire R, Miller M, Kearney N. Patients's perceptions and experiences of using a mobile phone-based advanced symptom management system (ASyMS ©) to monitor and manage chemotherapy related toxicity. Eur J Cancer Care (Engl). 2009;18(2):156-64.
37. Kofoed S, Breen J, Gough K, Aranda S. Benefits of remote real-time side-effect monitoring systems for patients receiving cancer treatment. Oncology Reviews. 2012;6(7):1-13.
38. Bower P, Kennedy A, Reeves D, Rogers A, Blakeman T, Chew-Graham C, et al. A cluster randomised controlled trial of the clinical and cost-effectiveness of a 'whole systems' model of self-management support for the management of long- term conditions in primary care: trial protocol. Implement Sci. 2012;7:7.
39. Palmer R, Enderby P, Cooper C, Latimer N, Julious S, Paterson G, et al. Computer therapy compared with usual care for people with long-standing aphasia poststroke: a pilot randomized controlled trial. Stroke. 2012;43(7):1904-11.
40. Domingo M, Lupon J, Gonzalez B, Crespo E, Lopez R, Ramos A, et al. [Noninvasive remote telemonitoring for ambulatory patients with heart failure: effect on number of hospitalizations, days in hospital, and quality of life. CARME (CAtalan Remote Management Evaluation) study]. Rev Esp Cardiol. 2011;64(4):277-85.
41. Jenkinson C, Fitzpatrick R, Brennan C, Swash M. Evidence for the validity and reliability of the ALS assessment questionnaire: the ALSAQ-40. Amyotrophic lateral sclerosis and other motor neuron disorders : official publication of the World Federation of Neurology, Research Group on Motor Neuron Diseases. 1999;1(1):33-40.
42. Moher D, Hopewell S, Schulz KF, Montori V, Gotzsche PC, Devereaux PJ, et al. CONSORT 2010 explanation and elaboration: updated guidelines for reporting parallel group randomised trials. BMJ. 2010;340:c869.
43. Schulz KF, Altman DG, Moher D. CONSORT 2010 statement: updated guidelines for reporting parallel group randomised trials. BMJ. 2010;340:c332.
44. Jenkinson C, Peto V, Jones G, Fitzpatrick R. Interpreting change scores on the Amyotrophic Lateral Sclerosis Assessment Questionnaire (ALSAQ-40). Clin Rehabil. 2003;17(4):380-5.
45. Jenkinson C, Fitzpatrick R, Swash M, Jones G. Comparison of the 40-item Amyotrophic Lateral Sclerosis Assessment Questionnaire (ALSAQ-40) with a short-form five-item version (ALSAQ-5) in a longitudinal survey. Clin Rehabil. 2007;21(3):266-72.
46. Epton J, Harris R, Jenkinson C. Quality of life in amyotrophic lateral sclerosis/motor neuron disease: a structured review. Amyotroph Lateral Scler. 2009;10(1):15-26.
47. Jenkinson C, Hobart J, Chandola T, Fitzpatrick R, Peto V, Swash M. Use of the short form health survey (SF-36) in patients with amyotrophic lateral sclerosis: tests of data quality, score reliability, response rate and scaling assumptions. J Neurol. 2002;249(2):178-83.
48. McDermott CJ, Maguire C, Cooper CL, Ackroyd R, Baird WO, Baudouin S, et al. Protocol for diaphragm pacing in patients with respiratory muscle weakness due to motor neurone disease (DiPALS): a randomised controlled trial. BMC Neurol. 2012;12:74.



For peer review only - http://bmjopen.bmj.com/site/about/guidelines.xhtml

49. Evaluation of the impact of a CoughAssist® mechanical in-exsufflator (MI-E) device on morbidity, quality of life and survival in patients with motor neurone disease (MND) using non-invasive ventilation (NIV) [Internet].
50. Julious S. Sample size of 12 per group rule of thumb for a pilot study. Pharm Stat. 2005;4(4):287-91.
51. Cedarbaum JM, Stambler N, Malta E, Fuller C, Hilt D, Thurmond B, et al. The ALSFRS-R: a revised ALS functional rating scale that incorporates assessments of respiratory function. BDNF ALS Study Group (Phase III). J Neurol Sci. 1999;169(1-2):13-21.
52. NIHR CLAHRC South Yorkshire. Telehealth & Care Technologies (TaCT) for Long Term Conditions 2012. Available from: http://clahrc-sy.nihr.ac.uk/theme-tact-introduction.html.
53. O'Rourke N, Tuokko HA. Psychometric properties of an abridged version of The Zarit Burden Interview within a representative Canadian caregiver sample. Gerontologist. 2003;43(1):121-7.
54. Zigmond AS, Snaith RP. The hospital anxiety and depression scale. Acta Psychiatr Scand. 1983;67(6):361-70.
55. Francis JJ, Johnston M, Robertson C, Glidewell L, Entwistle V, Eccles MP, et al. What is an adequate sample size? Operationalising data saturation for theory-based interview studies. Psychology & health. 2010;25(10):1229-45.
56. Hoddinott P, Pill R. Qualitative research interviewing by general practitioners. A personal view of the opportunities and pitfalls. Fam Pract. 1997;14(4):307-12.
57. Richards HM, Schwartz LJ. Ethics of qualitative research: are there special issues for health services research? Fam Pract. 2002;19(2):135-9.
58. (UK) GMC. Confidentiality. 2009.

For peer review only

**Appendix 1: Drugs produced by Abbott Healthcare Ltd.**

Fenofibrate
Pancreatin
Moxonidine
Estradiol/dydrogesterone
Mebeverine
Betahistine
Fluvoxamine maleate
Lactulose
Estradiol, oral applications
Influenza virus vaccine
Eprosartan mesylate
Ibuprofen
Flurbiprofen
Propafenone
Clarithromycin
Verapamil

For peer review only

# Appendix 2: CONSORT flow diagram

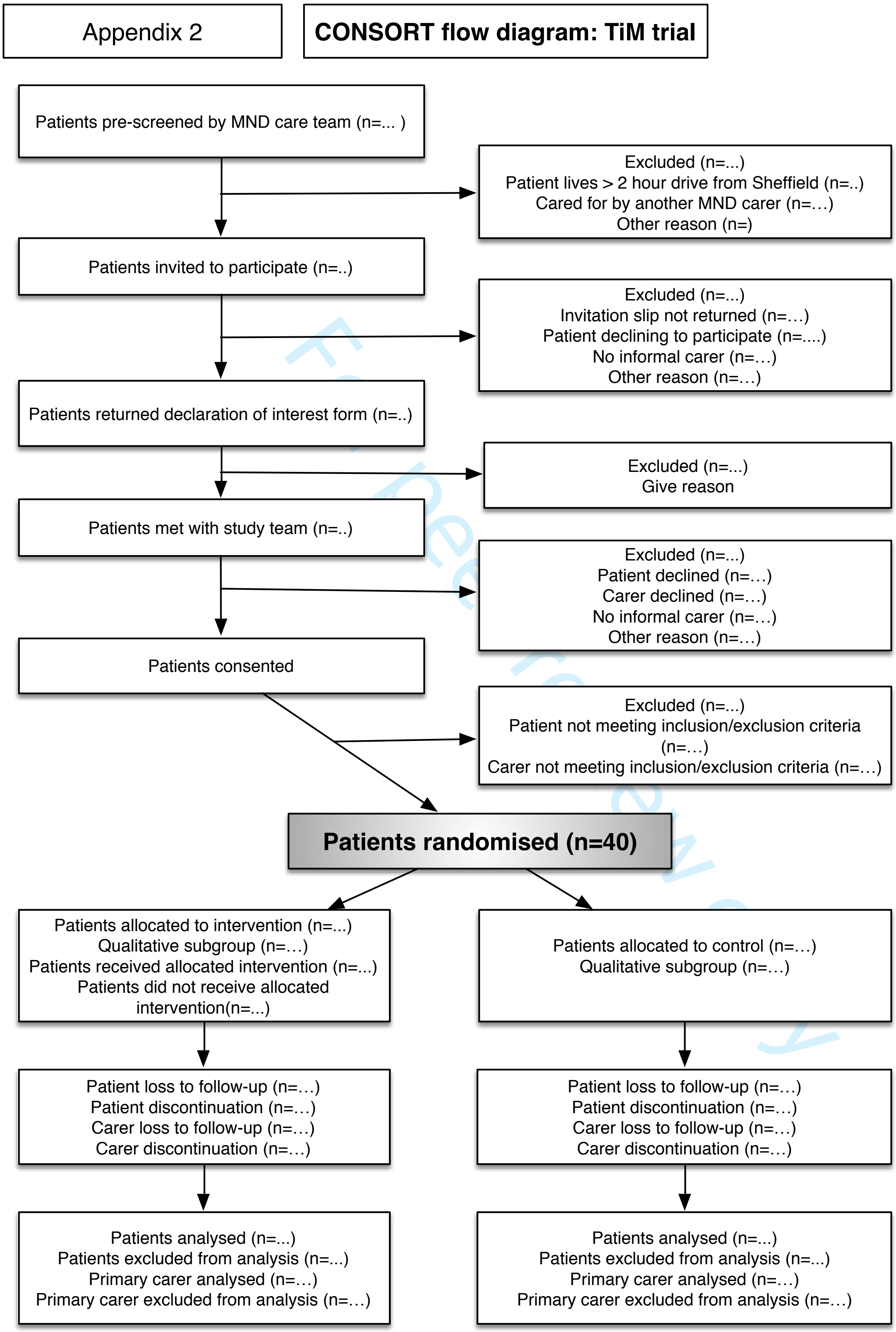




For peer review only - http://bmjopen.bmj.com/site/about/guidelines.xhtml

# Appendix 3 Qualitative interview topic guides

## Baseline interview (control group)

"I'd just like to reiterate that everything you say in the interview is confidential to me and the research team. If anything you say is used in a publication then it will be anonymous. I'll be recording the interview to make sure I've don't miss anything important. It will take about 15 minutes but we can stop if you wish. If you have any questions I can answer them now or at the end of the interview. There aren't any right or wrong answers - I'm simply interested in your experience and your views. I'd like to know how you found filling in the questionnaire booklet. The questions are designed to understand more about your condition and experiences of MND."

For patients:

- Some are asking quite personal questions. How do you feel about that?
- Do you think the questionnaires asked questions about your life with MND?
- Did you find any of the questionnaires confusing?
- Did you find any of the questionnaires upsetting?
- What would you think about filling in these questionnaires again?
- If so, how would you fill them in?
- If you needed help, how would you fill in your questionnaire?

For carers

- Did you find any of the questionnaires confusing?
- Did you find any of the questionnaires upsetting?
- Some are asking quite personal questions. How do you feel about that?
- What would you think about filling in these questionnaires again?

For peer review only

**Intervention group**

Interviews will be conducted at 1 and 6 months. Early interview results will guide later interviews. Participants will be provided with the topic guide questions prior to the interview in order for them to communicate their answers easily. The main focus of the interviews is on:

- Participants experience and expectations of technology
- Participants' expectations of telehealth services
- Barriers and aids to recruitment and compliance with the TiM system
- How the TiM system is used at home by patients and their carers
- The impact of using the TiM system on their lives and well-being
- The impact of education on their day-to-day lives
- The experiences of carers monitoring
- Whether the outcome measures used capture the changes in participants well being associated with using the TiM system.
- How the system would be used outside a trial

**1-month interview (intervention group)**

"I'd just like to reiterate that everything you say in the interview is confidential to me and the research team. If anything you say is used in a publication then it will be anonymous. I'll be recording the interview to make sure I've don't miss anything important. It will take about an hour but we can stop at any time if you wish. If you have any questions I can answer them now or at the end of the interview. There aren't any right or wrong answers. I'd like to here about your experiences of starting using the TiM system and of MND care. You remember you received some questions in the post, we'll be going over those subjects again today"

Previous experiences in MND care

- Can you tell me a little about how you came to get the diagnosis of MND?
- What have your experiences been since then?
- Can you tell me about your last MND hospital clinic visit?
- Have you used the MND helpline?
- How do you manage if you have a question or problem?
- What would you say you are most worried about?
- How do you think your MND team have helped you?
- How do you think your care could be better?
- What problems do you think have been most troublesome?
- How much do you know about MND?



For peer review only - http://bmjopen.bmj.com/site/about/guidelines.xhtml

For carers

- How do you get the support you need as a carer?
- How do you find the help the MND team gives?
- How do you think your care could be better?
- What problems do you think have been most troublesome?
- How much do you know about MND?

Expectations of the TiM system

- Before the start of the study what technology did you use?
- What did you expect the TiM system would be like?
- Is there anything you would have hoped it would have?
- Is there anything that worried you about it?

Experiences of training and starting to use the TiM system

- What did you think when you first saw it?
- How did you find the training?
- Do you remember what it was like using it for the first time?

Barriers and facilitators to using the TiM system

- Is there anything things you like about it?
- Is there anything you don't like?
- Has it worked every time as you expected?
- Do you think you will continue to use it regularly?  Why?
- What have you told your friends about it?

For the carer

- How have you found using the TiM system?
- What was it like using it for the first time?
- How have you found the questions?

For peer review only

**6-month interview (intervention group)**

"I'd just like to reiterate that everything you say in the interview is confidential to me and the research team.  If anything you say is used in a publication then it will be anonymous.  I'll be recording the interview to make sure I've don't miss anything important.  It will take about an hour but we can stop at any time if you wish.  If you have any questions I can answer them now or at the end of the interview.  There aren't any right or wrong answers.  I'd like to here about your experiences of using the TiM system and how it has affected your life and your MND care.  You remember you received some questions in the post, we'll be going over those subjects again today"

For patients:

- How have you found using the TiM system?
- How often do you use it?
- Is it easy to use?
- Have there been any problems with it?
- Has the MND nurse contacted you about your answers?
- Have you talked about your answers during your clinic visits?
- How has it changed your MND care?
- Have you used the education section or the problem list?
- Would you like to use it as part of your routine care?
- How would you improve it?

For carers

- How have you found answering the questions?
- Has the MND nurse contacted you about your own well being?
- Would you like to use it as part of your routine care?

For peer review only

# Telehealth in Motor Neurone Disease

Telehealth in Motor Neurone Disease (TiM): A mixed methods, randomised controlled, pilot study of the use of the TiM telehealth system to deliver highly specialised care in Motor Neurone Disease, at a distance

**Statistical Analysis Plan Version 1.3 17/8/16**

For peer review only

*Authored by*

______________________________/___/____

*Dr Esther Hobson* *Date*

*TiM trial manager & co-investigator*

*SITraN, University of Sheffield*

*Approved by*

______________________________/___/____

*Dr Christopher McDermott* *Date*

*TiM Chief Investigator*

*SITraN, University of Sheffield*

______________________________/___/____

*Mr Michael Bradburn* *Date*

*TiM Trial Statistician*

*CTRU, University of Sheffield*

Contents

1 Introduction, Study Design & Objectives 5

1.1 Study Background 5
1.2 Primary Objectives 5
1.2.1 Feasibility Outcomes 5
1.2.2 Clinical Outcomes 6
2 Sample Size Estimation 7
3 Randomisation & Blinding 8
4 Interim Analysis & Study Monitoring. 8
5 Data Sources, Evaluability & Study Populations 8
5.1 Data Sources 8
5.2 Data Collection 9
5.3 Protocol non compliances 9
5.4 Study Population 9
5.5 Analysis Populations 9
6 Statistical Analysis 10
6.1 General considerations 10
6.2 Recruitment and attrition rates 10
6.2.1 Eligibility 10
6.2.2 Participant Attrition 11
6.3 Status of participants and completion of outcome measures 11
6.4 Baseline Characteristics 11
6.5 TiM Treatment adherence 12
6.6 Clinical outcomes 13
6.6.1 Patient outcomes 13
6.6.2 Carer clinical outcomes 16
6.6.3 Health economic outcomes 16
6.6.4 Patient experiences 16
6.6.5 Safety 17
6.7 Estimation of primary outcome and sample size for a main trial 17
6.8 Economic Evaluation Analysis 18
6.9 TiM process evaluation 18
7 Detailed Statistical Methods & Calculations 19
7.1 Missing Spurious & Unused Data 19
8 Implementation of the Analysis Plan 19
9 Modifications to the Original Protocol Analysis Statement 19
10 Appendix 20
11 20
10.2 Example Tables and Figures 22
12 References 26


For peer review only - http://bmjopen.bmj.com/site/about/guidelines.xhtml

## List of Abbreviations

| | |
|---|---|
| AE | Adverse event |
| ALS | Amyotrophic lateral sclerosis |
| ALSAQ-40 | Amyotrophic Lateral Sclerosis Assessment Questionnaire – long form |
| ALS-FRS-R | Amyotrophic lateral sclerosis rating scale-revised |
| CI | Confidence Interval |
| CONSORT | Consolidated standards of reporting trials |
| CRF | Case Report Form |
| CSS-MND | Clinical Saliva Scale for Motor Neurone disease |
| CTRU | Clinical trials research unit, University of Sheffield |
| EQ-5D-3L | EuroQol Group Health Questionnaire |
| EQ-5D+D | EQ-5D questionnaire with dignity bolt-on |
| HADS | Hospital Anxiety and Depression Scale |
| ICH | International Conference on Harmonisation of Technical Requirements for Registration of Pharmaceuticals for human use |
| ITT | Intention To Treat |
| QoL | Quality of life |
| MND | Motor neurone disease |
| NIV | Non-invasive ventilation |
| SAE | Serious adverse event |
| SAP | Statistical analysis plan |
| SD | Standard deviation |
| SF-36 RAND | 36-Item Short Form Survey from the RAND Medical Outcomes Study |
| SITraN | Sheffield Institute of Translational Neuroscience |
| SOP | Standard operating procedure |
| Telehealth | Remote monitoring of patients physiology or patient reported measures, forwarded to a central service with the aim to diagnoses or monitor a medical condition |
| TMG | Trial management Group |
| TSC | Trial Steering Committee |
| TiM | Telehealth in Motor neurone disease |
| TM | Trial manager (EH) |
| ZBI | Zarit Burden Index |

For peer review only

1. Introduction, Study Design & Objectives

This Statistical Analysis Plan (SAP) is written in conjunction with the ICH E9 (International Conference on Harmonisation of Technical Requirements for Registration of Pharmaceuticals for human use; ICH Harmonised Tripartite Guideline: Statistical Principles for Clinical Trials E9), applicable standard operating procedures (SOPs) from the Sheffield Clinical Trials Research Unit (CTRU) and trial documents (Protocol, case report form (CRF) and Data Validation Specifications). This SAP will guide the trial manager (TM) and Trial Statistician during the statistical analysis of all quantitative outcomes in order to answer the objectives of the study.

## 1.1 Study Background

This is a single-centre, pilot, mixed methods, randomised controlled trial to explore the feasibility and acceptability of using the TiM (Telehealth in Motor Neurone Disease (MND)) system in clinical practice and explore the feasibility of a larger, multicentre trial. This plans refers to the TiM trial protocol V1.5 April 2015.

All analyses will be performed in a validated statistical software package such as GraphPad prism.

## 1.2 Primary Objectives

As this is a pilot study, no formal primary clinical outcome will be defined. Instead, the trial will assess the feasibility and requirements of a full-scale study of the TiM as defined by the as successful recruitment of 40 eligible patients and their primary carer; and the feasibility, acceptability, safety and use of the TiM system within a health service. The specific objectives and outcomes of this study are separated into two groups: feasibility and clinical outcomes.

### 1.2.1 Feasibility Outcomes

**Feasibility of a full-scale study**

- To make a decision on the primary outcome for the main trial. The mechanism for choosing this outcome will be informed by statistical considerations which are detailed in section 6.7.
- Number of potentially eligible patients among the pool of patients under the care of the Sheffield MND care centre
- Number/characteristics of eligible patients approached for the study:
- List of reasons for declining/refused consent;
- Participant attrition rate
- List of reasons for attrition
- Number of missing values/incomplete cases (see **Error! Reference source not found.**)



For peer review only - http://bmjopen.bmj.com/site/about/guidelines.xhtml

- Treatment receipt/adherence;
- Patient, carer and clinician views on intervention/research protocol (using qualitative methods).

Feasibility/safety of TiM system:

- Treatment receipt/participant and staff adherence
- Participant and clinician acceptability of the intervention (using qualitative methods and the TiM system experience questionnaire and Shadow monitoring protocol)
- Patient, carer and clinician views on intervention (using qualitative methods);
- Incidence of TiM system technical problems;
- Incidence of adverse events related to intervention.

Participant and clinician views will be investigated using qualitative interviews (described in the protocol). Participant and clinician acceptability will be reported based on TiM system experience questionnaire and Shadow monitoring questionnaire.

### 1.2.2 Clinical Outcomes

The following clinical outcomes will be reported using self-completed questionnaires baseline, 3, 6, 12 and 18 months.

Patient outcomes

- Quality of life (QoL) measures
    - ALSAQ-40 (an MND disease specific quality of life score)
    - SF-36-RAND
    - EQ-5D+D (EQ-5Q-3L with a dignity bolt-on)
- Clinical outcomes
    - ALS-FRS-R (an MND disease specific functional rating score
    - Pain score (modified Likert scale)
    - CSS-MND Saliva Severity Scale (designed for use with MND patients) plus global change scale
    - Hospital Anxiety and Depression Scale (HADS)
- Health resource usage questionnaire
- Patient experience questionnaire
- TiM experience questionnaire

Carer outcomes

- SF-36 RAND
- 12 item Zarit Burden Inventory (ZBI) (53)
- Hospital Anxiety and Depression Scale (54)
- Carer satisfaction questionnaire



For peer review only - http://bmjopen.bmj.com/site/about/guidelines.xhtml

The following safety outcomes will be assessed at every clinical visit:

- Incidence of adverse events (AEs)
- Clinician satisfaction

## 2 Sample Size Estimation

The study aims to recruit a total of 40 patients and their carers. 20 patients and their primary carer will be randomised to the intervention arm (a minimum of 6 months use of the TiM telehealth plus usual care) and 20 patents and their carer in the control arm (usual care).

Since the proposed trial is primarily an assessment of the acceptability of the intervention and the feasibility of a full trial, the proposed sample size is not based on standard statistical parameters such as a clinically relevant difference between groups. Instead, the sample size is justified on the grounds of quantifying patient variance (i.e. the standard deviation) in the proposed outcome measures (in particular quality of life measures) and on feasibility of the full trial, as follows:

- A sample size of 40 patients allows a standard deviation to be estimated to within a precision of ±20% of its true underlying value with 90% confidence. This estimate will be synthesised by combining baselines measurements of quality of life measurement standard deviations with those observed in other published studies and on-going trials within SITraN, to provide a robust estimate for use in the sample size calculation for the full trial.

- Given the rarity of MND, any definitive study will be infeasible if the required sample size is substantial. Assuming the upper limit for feasible UK study is around 200-300 patients in total, it follows that the full study would need powering to detect a standardised effect size of at least 0.4 SDs. This pilot trial will provide a preliminary assessment of whether the intervention might feasibly achieve this, and inform the choice of outcome measures for the proposed full study.

This sample size is also in keeping with the proposal of 12 evaluable patients per arm in a pilot study (after withdrawal or drop-out) (1).



For peer review only - http://bmjopen.bmj.com/site/about/guidelines.xhtml

# 3 Randomisation & Blinding

Randomisation is conducted according to the protocol.

The patient, clinicians, TM and trial team are not blinded to the outcomes. Data entry for follow-up clinical outcomes was performed by an independent research nurse, not involved in the study. Blinding of this nurse was impractical given additional measures were collected for those in the intervention group. The TM will undertake the analysis under the supervision of the independent trial statistician. Blinding during analysis was impractical given the small number of participants who had with unique and characteristics which are likely to be identifiable to the TM. This will be reported as a limitation

# 4 Interim Analysis & Study Monitoring.

This is a pilot study with no planned interim analysis or early stopping. Two committees have been set up to govern the conduct of the study:

- Trial Steering Committee (TSC)
- Trial Management Group (TMG)

Decisions to stop the trial early on grounds of safety will be made by the Trial Steering Committee or funding body. There will not be a Data Monitoring and Ethics Committee for this study as it is considered low risk. No interim analysis is planned.

The TM will receive notifications of all carers whose Hospital Anxiety and Depression subscores exceed 11. These events will be recorded as AEs, reported to the TMG and TSG during the study and reported in the analysis.

# 5 Data Sources, Evaluability & Study Populations

## 5.1 Data Sources

Data used in this study will come from data entered onto CRFs and questionnaires and from data entered directly on the CTRU database (PROSPECT). The data will be stored on the database with the exception of the randomisation list which is held on www.sealedenvelope.com and allocation verified by the data management team. Electronic data will be extracted from the system during the trial for the purpose of checking (validating) and trial progress reports. Access to PROSPECT is controlled by usernames and encrypted passwords, and a privilege management feature will be used to ensure that users have access to only the minimum amount of data required to complete their tasks. This will be used to restrict access to personal identifiable data.



For peer review only - http://bmjopen.bmj.com/site/about/guidelines.xhtml

For peer review only

## 5.2 Data Collection

Data will be collected from the participants and their carers at:

- Consent and Screening, eligibility and baseline
- Month 3, 6, 12 and 18
- Each clinic visit (Shadow monitoring protocol)
- End of study (participant status alive/dead and date of death).

**Due to the pilot nature of the study there are no predefined protocol non compliances other than misrandomisation or randomisation in error. Intervention adherence will be assess as an outcome (see section 6.5).**

## 5.3 Protocol non compliances

**Due to the pilot nature of the study there are no predefined protocol non compliances other than misrandomisation or randomisation in error. Intervention adherence will be assess as an outcome (see section 6.5).**

## 5.4 Study Population

Described in the protocol.

## 5.5 Analysis Populations

The intention to treat population (ITT) includes all patients for whom consent is obtained and who are randomised to treatment. This is the primary analysis set and endpoints will be summarised for the intention to treat population unless stated otherwise.



For peer review only - http://bmjopen.bmj.com/site/about/guidelines.xhtml

# 6 Statistical Analysis

## 6.1 General considerations

As the trial is a pilot parallel group randomised controlled trial, data will be reported and presented according to the proposed modifications for reporting pilot trials as well as the Consolidated standards of reporting trials (CONSORT) statement (2,3). The analysis will be performed on an ITT basis. The final analysis will be performed after data lock by the TM under the supervision of the study statistician who will also be responsible for quality checking the results.

**Each planned follow-up timepoint will use a time window to ensure that responses have been collected within a reasonable time frame. The time windows allow a slippage of four weeks at 3 months and six weeks thereafter, as outlined below:**

**3 months:** **within 61-91 days following randomisation**

**6 months:** **within 140-224 days following randomisation**

**12 months:** **within 323-407days following randomisation**

**18 months:** **within 506-590 days following randomisation**

## 6.2 Recruitment and attrition rates

Relevant summaries related to recruitment, consent and patient throughput will be reported and presented in a CONSORT flow diagram (see appendix, Figure 1).

The following will be reported:
The number of (potential) participants;

- Potentially eligible as identified by the study team at participating centres,
- Approached for the study,
- Not randomised (with reasons),
- Randomised,
  - allocated to treatment
  - allocated to control
- Withdrawn and lost to follow up (with reasons),
- Discontinuing TiM intervention,
  - reasons for discontinuation
- Included and excluded from analysis,
  - Reasons for exclusion.

### 6.2.1 Eligibility

Described in the study protocol



For peer review only - http://bmjopen.bmj.com/site/about/guidelines.xhtml

### 6.2.2 Participant Attrition

The rate of attrition will be reported (defined as the proportion of the consented and randomised participants who withdrew or were lost to follow up). The reasons for attrition, where provided, will be reported as number and percentage in each category.

## 6.3 Status of participants and completion of outcome measures

We will report the status of patients and carers at each time point.

At each time-point we will report the number of patients and carers:

- Returning the postal questionnaire booklet
- Completing each questionnaire

We will report these by treatment group and overall.

For the patient and carer questionnaires the response rate at each time point (measured as the total number of questionnaires completed as a fraction of total number of patients alive) will be reported. An example table is given in section 0 (Table 1).

## 6.4 Baseline Characteristics

The baseline demographics and clinical characteristics of the participants will be reported. For the continuous variables, (e.g. age) either mean and standard deviation will be presented or median and inter quartile range (IQR) depending on the distribution of the data. The number of observations used in each calculation will be presented alongside the summaries. For the categorical variables, the number and percentage of participants in each of the categories and the total number of observations will be presented.

All baseline summaries will be presented and reported for each treatment group and in total. An example of the table of baseline summaries is given in section 0 (



For peer review only - http://bmjopen.bmj.com/site/about/guidelines.xhtml

Table 2). No statistical significance testing will be done to test baseline imbalances between the intervention arms but any noteworthy differences will be descriptively reported.

For peer review only



For peer review only - http://bmjopen.bmj.com/site/about/guidelines.xhtml

The following summaries will be presented:

| | |
|---|---|
| Demographics | Age, gender, technology use |
| MND Characteristics | Age of onset, disease duration, classification of MND (e.g. ALS, PMA, PLS), clinical stage of MND, use of non-invasive ventilation (NIV)/gastrostomy, riluzole use |
| Carer demographics | Age, gender, relationship to patient, technology use |
| Patient reported outcomes | ALS-FRS-R (including upper limb function), ALSAQ40, RAND36 and subscores, CSS-MND, HADS, pain score, EQ-5D+D, patient experience, health resource use (number and type of clinical encounters and hospital admissions in last 3 months, carer requirements) |
| Carer reported outcomes | RAND36, ZBI, HADS, carer experience |

## 6.5 TiM Treatment adherence

**Intervention adherence will be reported as the number of TiM sessions attended within between recruitment and the end of March 2016 and the mean and SD of percent adherence. We will also report adherence at 1, 3, 6, 9, 12, 15 and 18 months.**

**We will also report**

- **The number and percentage of participants that completed 50% and 75% of expected sessions.**
- **A description of the adherence of each patient and carer using the TiM over the course of the trial.**

**Any reasons for poor adherence will be reported where available although it was not possible to identify reasons for all missed sessions.**

**Cumulative session attendance will be displayed for each participant using a spaghetti plot to illustrate intervention adherence.**

**The number and percentage of participants that withdrew from the TiM intervention will be reported, alongside listings of:**



For peer review only - http://bmjopen.bmj.com/site/about/guidelines.xhtml

- **Reasons for withdrawing from intervention, where provided**
- **Number of TiM sessions (and %) before withdrawing from intervention**

## 6.6 Clinical outcomes

Descriptive statistics will be presented for the clinical outcomes; significance testing will not be undertaken. Continuous outcome measures will be presented as mean differences between groups and their associated 95% confidence intervals (CI). For categorical outcomes, the number and percentages falling into different categories and potential differences between groups in terms of the percentages in each category will be presented, together with their confidence intervals. Clinical outcomes will be presented for the ITT set with available 6 month and 12 month outcome data.

### 6.6.1 Patient outcomes

The following outcomes measured at 3, 6, 12, 18 months will be presented by group and overall.

The following patient –reported quality of life outcomes will be reported.

| | |
|---|---|
| ALSAQ-40 | Individual scores of five sub-scales and a summary aggregate score:<br>▪ physical mobility<br>▪ activities of daily living and independence<br>▪ eating and drinking<br>▪ communication<br>▪ emotional reactions |
| RAND-36 | A summary of the eight sub-scales and two aggregated scales:<br>▪ Physical Functioning<br>▪ Role Limitations due to Physical Problems<br>▪ General Health Perceptions<br>▪ Vitality<br>▪ Social Functioning<br>▪ Role Limitations due to Emotional Problems<br>▪ General Mental Health<br>▪ Health Transition<br>▪ Aggregate physical health<br>▪ Aggregate mental health |
| EQ-5D+D | Health utility (as derived from the five questions)<br>Thermometer health scale<br>Health utility plus dignity (as derived five questions plus |



For peer review only - http://bmjopen.bmj.com/site/about/guidelines.xhtml

| | |
|---|---|
| | dignity bolt-on) |

In each case the within-group results will be summarised as mean (SD), and the difference between the two as the mean difference together with its CI. Forest plots of confidence intervals of different widths (e.g. 95%, 90%, 80%) with respect to the treatment difference in the overall ALSAQ40 score and RAND36 (mental and physical domain) will be used to illustrate the strength of preliminary evidence (see Figure 2) (Lee, 2014).

In each case, the summaries will be presented by treatment group and time point (see Table 3 and

For peer review only



For peer review only - http://bmjopen.bmj.com/site/about/guidelines.xhtml

ll be presented for the clinical outcomes; significance testing will not be undertaken.
significance testing will not be undertaken. Continuous outcome measures will be
ignificance testing will not be undertaken. Continuous outcome measures will be
presented as mean differences between groups and their associated 95% confidence
erences between groups and their associated 95% confidence intervals (CI). For
5% confidence intervals (CI). For categorical outcomes, the number and percentages
% confidence intervals (CI). For categorical outcomes, the number and percentages
ntages falling into different categories and potential differences between groups in
potential differences between groups in terms of the percentages in each category will
potential differences between groups in terms of the percentages in each category will
ome data.
ll be presented by group and overall.
tcomes will be reported.
ary aggregate score:
obility
g
-36
le 3 and
e number of patients alive and the number of patients completing the outcome
score mean and SD
ure will be scored as described below and compared, where possible and relevant to

ompared, where possible and relevant to population values.
be scored as described in Ware et al (4). In the case of partially completed
9D4-BDB0-
C034</uuid><priority>0</priority><publications></publications></citation>(4). In
publications></citation>(4). In the case of partially completed questionnaires, scores
questionnaires, scores will be calculated for domains in which at least 50% of the
ons have been answered. Taking physical functioning as example, if at least five of the
ns have been answered. Taking physical functioning as example, if at least five of the
lity (Q1-10), activities of daily living/independence (Q11-20), eating and drinking,
ity (Q1-10), activities of daily living/independence (Q11-20), eating and drinking, (Q21-
(Q21-23) communication (Q24-30), emotional functioning (Q31-40). Each question is
unctioning (Q31-40). Each question is scored 0 (never) to 4 (always/cannot do at all).
nctioning (Q31-40). Each question is scored 0 (never) to 4 (always/cannot do at all).

5 +Q6 +Q7 +Q8 + Q9 + Q10)/40) x 100.
I).
urology, Neurosurgery & Psychiatry</title><type>-100</type><subtype>-
0</subtype><uuid>0303282F-D994-4EC9-B986-
bundle><authors><author><firstName>C</firstName><lastName>Jenkinson</lastNa
or><firstName>C</firstName><lastName>Jenkinson</lastName></author><author><f
stName>Jenkinson</lastName></author><author><firstName>J</firstName><middle
tName>Jenkinson</lastName></author><author><firstName>J</firstName><middleN


For peer review only - http://bmjopen.bmj.com/site/about/guidelines.xhtml

The following other clinical outcomes will be presented:

| | |
|---|---|
| Hospital anxiety and depression score | The Anxiety and Depression subscores |
| Pain (Likert scale) | Current and average weekly score |
| ALS-FRS-R | Total score |
| CSS MND | Total score<br>% patients reporting a clinically significant improvement or worsening (according to Global change CSS-MND self-reported statement) |
| Clinical encounters | The number of clinical encounters in the 6 months following randomisation, by type and location and reason |
| Hospital admissions | The number and percentage of patients admitted to hospital, and the number of hospitalisations, by type, location and reason |

The HADS anxiety and depression inventories will be scored using the approach of Zigmond and Snaith (7). Each domain will be calculated as the sum of seven questions, each of which is scored 0-3, giving a total score which ranges from 0 and 21. In the case of partially completed questionnaires, the domain will be scored and upweighted provided at least four of the seven questions have been answered.

Self-completed revised ALS functional rating scale (ALS-FRS-R) consists of 12 questions scoring 0-4 (8). Sub-domains include upper limb, lower limb, bulbar and respiratory.

Pain score: the current level of pain (0-10 likely scale) and the average current weekly level of pain (0-10) will be represented as a mean and SD.

Modified CSS MND saliva score is awaiting validation. The total score is the total of all answers scoring 0-3 for each question A to J (9). The percentage of patients reporting a change on the saliva clinical change assessment will be reported.

### 6.6.2 Carer clinical outcomes

The following carer-reported QoL outcomes will be presented is the same manner as described for the patients.

- RAND 36
- HADS



For peer review only - http://bmjopen.bmj.com/site/about/guidelines.xhtml

- Zarit Burden Index

The each of the 12 items in the shortened Zarit burden inventory is scored 0 (Never) to 4 (nearly always) (10). A total score between 0 and 48 will be reported.

### 6.6.3 Health economic outcomes

A complete health economic analysis is beyond the scope of this plan. However, descriptions of the following clinical outcomes will be reported, by group and overall:

| | |
|---|---|
| Clinical encounters | The number of clinical encounters recorded at each encounter following randomisation, by type and location. |
| Hospital admissions | The number and percentage of patients admitted to hospital recorded at each encounter. the number of hospitalisations |
| Informal care requirements | The number of hours of informal care recorded by patients. |
| Formal care requirements | The number of hours of formal care recorded by patients. |

### 6.6.4 Patient experiences

The following will be reported by group and overall.

| | |
|---|---|
| Patient care experience | Percentage of patients agreeing and disagreeing with each satisfaction statement |
| Carer care experience | Percentage of carer agreeing and disagreeing with each satisfaction statement |
| Patient TiM experience (intervention only) | Percentage of patients agreeing and disagreeing with each satisfaction statement |
| Carer TiM experience (intervention only) | Percentage of carer agreeing and disagreeing with each satisfaction statement |

All free text responses will be reported.

### 6.6.5 Safety

Adverse events are recorded at every clinic appointment and patients will report health resource use and hospital admissions. Reported admissions will be followed up by the



For peer review only - http://bmjopen.bmj.com/site/about/guidelines.xhtml

TM and records as serious adverse events. HADS carer scores will be calculated and reported to the TM for action on an ongoing basis during the trial if either the depression or the anxiety subscore exceeds 11. These will be reported to the TSC during the trial and reported in the analysis and recorded as adverse events.

Advents Events (AEs) will be reported as number and percentage of patients overall and by treatment group but no formal statistical analysis is planned. The following summaries will be presented;

| | |
|---|---|
| *AEs* | The number and percentage* of patients reporting an AE and the number of AEs in total |
| *AEs by category* | The number and percentage* of patients reporting an AE and the number of AEs for each pre-defined category (pain, acute infection, fractures) |
| *Serious AEs (SAEs)* | The number and percentage* of patients reporting an SAE and the number of SAEs in total |
| *Treatment-related AEs* | The number and percentage* of patients reporting a treatment related AE and the number of treatment related AEs |
| *All AEs* | A listing of all AEs including<br>- Description / Site / Signs and Symptoms<br>- Severity<br>- Relationship<br>- Action taken<br>- Outcome<br>- Seriousness |

*defined as a percentage of all patients randomised.

## 6.7 Estimation of primary outcome and sample size for a main trial

The variability in clinical outcomes will be reported as standard deviation by treatment group and overall alongside their upper 80% confidence limits to get a robust estimate of SD (as recommended by Kieser, 2007), and observed treatment difference.

Descriptive assessment will be used to inform sample size calculations for the definitive study. These assessments will be calculated for candidate measures for the full trial (RAND-36 and ALSAQ40), and will be based on:

- Observed treatment difference at 6 and 12 months
- Standard Deviation;
- Correlation between baseline and 6 month measurements;
- The extent of missing data in each outcome;
- Participant feedback on the most appropriate assessment (analysed qualitatively).


For peer review only - http://bmjopen.bmj.com/site/about/guidelines.xhtml

The standard deviation used in the sample size calculation will be derived from the residual variance of the regression model for which the outcome is the 6-month response and the covariates are treatment group and baseline.
A table of sample size estimates for a definitive study stratified by outcome measure and power (80%, 90%) will be provided. E.g. Table 5

## 6.8 Economic Evaluation Analysis

No economic analysis will be conducted but patient health resource use will be reported.

## 6.9 TiM process evaluation

The following will be reported:

Patient and carer feasibility:

- The time taken to complete each TiM session by patient and carer (mean, range). TiM session time is automatically recorded by the application but total time between starting and completing and session is recorded. This includes any time delay because the patient pauses using the session and recommences it later e.g. the next day. Outliers will be identified and excluded with definition of outlier reported (e.g. > 600% of the average time);
- Adherence to weekly TiM sessions (see 6.5);

Clinical feasibility:

- Number, range and % of patient and carer sessions that trigger an overall red, amber and green flag;
- Number, range and % of patient and carer sub-sections that trigger an overall red, amber and green flag;
- Time taken for nurse to use the telehealth system per week, collected by nurse diary (mean, range, SD and time per patient enrolled in the system);
- Number of notes entered per patient.
- Shadow monitoring protocol (intervention)
    - Number of pre-clinic shadow monitoring forms completed
    - Number of clinic shadow monitoring forms completed
    - Clinician satisfaction: % agree/disagree with each statement
    - Free text comments will be reported.


For peer review only - http://bmjopen.bmj.com/site/about/guidelines.xhtml

# 7 Detailed Statistical Methods & Calculations

## 7.1 Missing Spurious & Unused Data

The extent of missing data will be reported. No sensitivity analyses involving imputation for missing data will be performed. Any spurious data will be queried and checked for consistency with data management before data lock.

Patient and carer questionnaires will be scored only if all relevant items that make up a domain are completed with the exception of RAND 36, HADS and ALSAQ40.

# 8 Implementation of the Analysis Plan

This SAP will be used as a work description for the statistician involved in the trial. All analyses will be performed by the TM (under the supervision of Trial Statistician MB).

Initially, blinded data will be delivered to the TM and MB by the data manager to define analysis sets and test statistical programs. Any queries will be communicated to the study and data manager prior to database lock. The database will be locked after agreement between the statistician, data manager and study manager. No changes will be made once the data has been locked. Database freeze and lock will be conducted in accordance with SOP DM012.

# 9 Modifications to the Original Protocol Analysis Statement

**None**

For peer review only

# 10 Appendix

*Figure 1: CONSORT flow diagram*

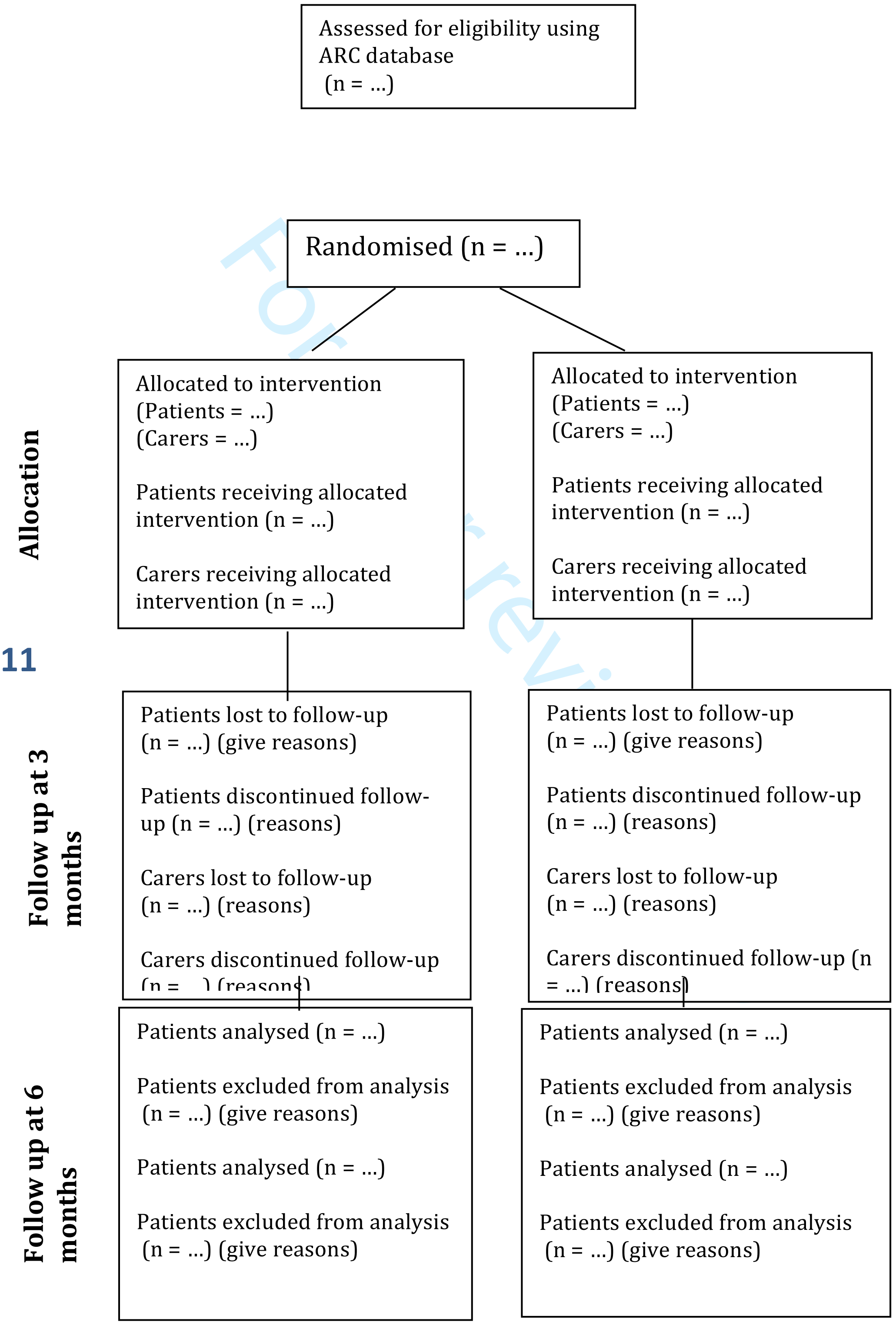


## 11



For peer review only - http://bmjopen.bmj.com/site/about/guidelines.xhtml

*Figure 2: Mean difference in ALSAQ40 with confidence intervals*

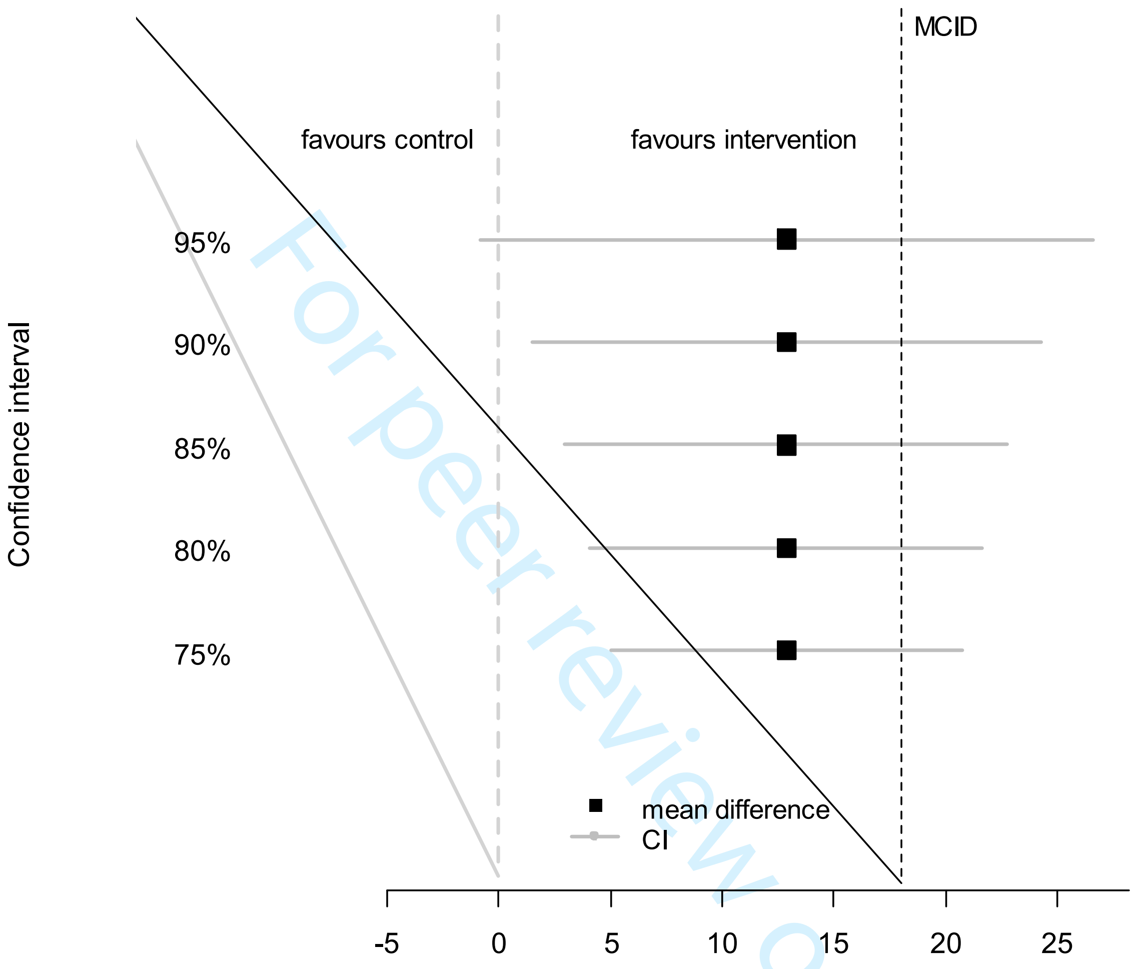


For peer review only

## 10.2 Example Tables and Figures

Note: The following tables are examples and do not include all outcome measures that will be included in the analysis.

*Table 1: Participant status*

| | 3 months | 6 months | 12 months | 18 months |
|---|---|---|---|---|
| **Telehealth** | | | | |
| Completed | N= | N= | | |
| Not completed | | | | |
| Died | | | | |
| Withdrew from study | | | | |
| Completed questionnaire but not within time window | | | | |
| Incomplete | | | | |
| **Control** | | | | |
| Completed | N= | N= | | |
| Not completed | | | | |
| Died | | | | |
| Withdrew from study | | | | |
| Completed questionnaire but not within time window | | | | |
| Incomplete | | | | |

*Completed includes questionnaires that were sufficiently complete to be used in the statistical analysis. Uncompleted refers to questionnaire booklets that were not returned. Incomplete refers to questionnaire booklets that were returned but insufficiently complete to be used in statistical analysis.

For peer review only

*Table 2: Participant baseline characteristics by treatment group*

| Characteristic | Scoring | Control (n=xx) | Intervention (n=xx) | All (n=xx) |
|---|---|---|---|---|
| Age (years) | Mean(SD, range) | x (xx) | x (xx) | x (xx) |
| | N | n | n | n |
| Gender | Male | n (%) | n (%) | n (%) |
| | Female | n (%) | n (%) | n (%) |
| | N | N | n | n |
| ALS-FRS-R | Mean(SD, range) | x (xx) | x (xx) | x (xx) |
| | N | n | n | n |
| King's clinical stage | Stage 1 | x (xx) | x (xx) | x (xx) |
| | Stage 2…etc. | | | |
| | N | n | n | n |

*This will be extended to include the other baseline variables measured.*

*Table 3: Display of outcome data by time, illustrated for pain*

| | Baseline | 3 months | | | |
|---|---|---|---|---|---|
| Outcome | | Mean (SD) | Change from baseline | *…repeat for other timepoints* | |
| Current pain: Mean (SD) | Mean (SD) | N= Mean (SD) | Mean (CI) | | |
| Control | Mean (SD) | N= Mean (SD) | Mean (CI) | | |
| Average pain: Mean (SD) | Mean (SD) | N= Mean (SD) | Mean (CI) | | |
| Control | Mean (SD) | N= Mean (SD) | Mean (CI) | | |



For peer review only - http://bmjopen.bmj.com/site/about/guidelines.xhtml

*Table 4: Clinical outcomes at six months: control vs intervention*

| | Change from baseline | | | | | | |
|---|---|---|---|---|---|---|---|
| | Intervention | | | Control | | | |
| Outcome | n | Mean | SD | n | Mean | SD | Mean difference (95% CI) |
| ALSAQ40 | x | xx | xx | x | xx | xx | xx (xx to xx) |
| RAND 36 (agg. physical) | x | xx | xx | x | xx | xx | xx (xx to xx) |
| RAND 36 (agg. mental) | x | xx | xx | x | xx | xx | xx (xx to xx) |
| HADS anxiety | x | xx | xx | x | xx | xx | xx (xx to xx) |
| HADS depression | x | xx | xx | x | xx | xx | xx (xx to xx) |
| Pain | x | xx | xx | x | xx | xx | xx (xx to xx) |
| … | | | | | | | |



For peer review only - http://bmjopen.bmj.com/site/about/guidelines.xhtml

For peer review only

Table 5: Sample size considerations for candidate primary outcome measures

| | **Effect size** | | **Standard deviation** | | **Power (%)** | **Number /group** | **Number /group + attrition** |
|---|---|---|---|---|---|---|---|
| **Outcome** | **MCID** | **Observed*** | | | | | |
| ALSAQ-40 total | xx | Xx | Observed | xx | 80 | NN | NN |
| | | | | | 90 | NN | NN |
| | | | Upper 80%CI | xx | 80 | NN | NN |
| | | | | | 90 | NN | NN |
| RAND-36 | | | | | | | |
| Agg physical | 5 | Xx | Observed | xx | 80 | NN | NN |
| | | | | | 90 | NN | NN |
| | | | Upper 80%CI | xx | 80 | NN | NN |
| | | | | | 90 | NN | NN |
| Agg mental | 5 | xx | … | | | | |
| *…repeat for other candidate measures* | | | | | | | |

**nb Observed effect size is for reference and is not used in sample size calculation*

For peer review only



For peer review only - http://bmjopen.bmj.com/site/about/guidelines.xhtml

# Shadow monitoring form

[Intervention]

T ☐☐☐

TiM Telehealth in Motor Neurone Disease

## Appointment summary

Date of clinic ☐☐ ☐☐ ☐☐☐☐ (d d m m y y y y)

Neurologist completing form ☐ CMD ☐ PJS ☐ Other → ________ *specify*

Mode of contact ☐ Face-to-face ☐ Telephone

Type of appointment ☐ Routine ☐ Unscheduled

Related to telehealth ☐ Yes ☐ No

Details

Next scheduled visit ☐☐ weeks

## Adverse events

Any adverse events (including hospital visits) since last visit? ☐ Yes ☐ No

*Please complete **Adverse event** or **Hospital attendance** form and contact CI immediately*

Any adverse drug reactions since last visit? ☐ Yes ☐ No

*Please complete **Adverse event** form and contact CI immediately*

## TiM

| | Completely disagree | Disagree | Neutral | Agree | Completely agree |
|---|---|---|---|---|---|
| The TiM data gave an accurate picture of the patient's current condition | ☐ | ☐ | ☐ | ☐ | ☐ |
| The TiM data could enable me to make appropriate decisions about the patient without seeing them in clinic | ☐ | ☐ | ☐ | ☐ | ☐ |
| The TiM data gave useful information about the carer's current condition | ☐ | ☐ | ☐ | ☐ | ☐ |
| The TiM was a positive influence on the consultation | ☐ | ☐ | ☐ | ☐ | ☐ |
| Prior to the patient attending you said you would arrange the patient to attend clinic in ______ weeks. Do you think this was an appropriate decision? | ☐ | ☐ | ☐ | ☐ | ☐ |

**Telehealth in motor neurone disease to increase access to specialist multidisciplinary care: a process evaluation.**

**Supplementary data file**

## Table of Contents


Appendix table 1 Participants' initial impressions of using the TiM app. .................. 2

Appendix table 2 The characteristics of those lacking confidence in technology user and their approach to using technology. ........................................................................ 3

Appendix table 3 Negative attitudes to technology expressed by participants....... 4

Appendix table 4 Facilitators for TiM use. ............................................................................ 5

Appendix table 5 Participants attitudes towards the frequency of TiM sessions... 6

Appendix table 6 Reasons for frequent and infrequent adherence to TiM identified during the interviews. ..................................................................................... 7

Appendix table 7 Participants' expectations of the potential communication and monitoring benefits of the TiM service. ........................................................................ 8

Appendix table 8 Participants' expectations of the TiM service................................... 9

Appendix table 9 The acceptability of the TiM questions. ........................................... 10

Appendix table 10 The validity / accuracy of the TiM questions................................ 11

Appendix table 11 Participants understanding of the TiM service. ........................... 12

Appendix table 12 Nurses' experience of using the TiM clinical portal. All quotes are from the Telehealth Nurse. ............................................................................................ 13

Appendix table 13 The accuracy and sensitive of the TiM information. Quotes are from the Telehealth Nurse (N) unless otherwise stated. ...................................... 14

Appendix table 14 Participants' experiences of how the TiM impacted on their care. ........................................................................................................................................ 15

Appendix table 15 The Telehealth Nurse's attitudes towards the value of the TiM. ................................................................................................................................................ 16

Appendix table 16 Participants experiences of clinician feedback. .......................... 17

Appendix table 17 The mismatch between the participants' and nurses' expectations of the TiM service. ................................................................................... 18

Appendix table 18 The problems associated with excessive TiM alerts. All quotes are from the Telehealth Nurse unless indicated. ............................................................ 19

Appendix table 19 The problems associated with excessive TiM alerts.................. 20

Appendix table 20 The future of the TiM. .......................................................................... 21

Appendix table 21 The Telehealth Nurse's attitudes towards how the TiM might be used in the future. ....................................................................................................... 22

Appendix table 22 How the TiM might be used by other teams ................................ 23




For peer review only - http://bmjopen.bmj.com/site/about/guidelines.xhtml

## Appendix table 1 Participants' initial impressions of using the TiM app.

| **Initial use of the app** | |
|---|---|
| Easy to use | *"It's so easy to do; it literally takes five minutes from home." P317 [daily technology user]* |
| Quick to use | *"It's not a problem is it? It takes minutes, it's not like you have to sit there for half a day and do it, it literally, it's, it's sort of minutes." P248 [daily technology user]* |
| TiM accessible and acceptable to the elderly | *"So this lady [P217], she's an elderly lady who you thought wouldn't have embraced anything like this.  But she has done and she, her and her husband both send it back; she's very disabled but able to use her hands quite well…they're both in their eighties; and she's a very good replier, sends it in." Telehealth nurse* |
| TiM accessible to those with significant disabilities | *"[There are] some are patients that, I'm surprised they took it up. I'm surprised; actually that, that, I never thought that he would use that… and he sends his back very, very well." Telehealth nurse* |
| Confidence the information would be held securely | *"I'm assuming that your department is one that's reasonably secure and has got reasonable standards… I can only make my judgement on the people I meet who are involved in it." C166* |

For peer review only

# Appendix table 2 The characteristics of those lacking confidence in technology user and their approach to using technology.

| **Characteristics of those with low technology confidence** | |
|---|---|
| Unsure if they would be able to use the TiM | *"…we're quite happy to deal with it, as long as it wasn't too techy" C062 [uses technology a few times a week]*<br>*"…as long as I can do it" P392 [uses technology daily]* |
| Lack of experience using technology | *"I don't use the internet for my job and it's all still paperwork orientated" C395 [uses technology daily]* |
| Difficulties with language | *"C: It's the system that's all. I don't catch on very well with it, that's ok. Like phones, I don't bother them really.*<br>*P: Couldn't read a text message.*<br>*C: couldn't do anything like that." C217 [never uses technology]* |
| Frightened they might make the TiM go wrong | *"Q: Have you used any of the other things that are on the tablet, so the education things or the website?*<br>*P: Oh I daren't touch all that stuff in case it goes wrong. (laughs)…I pressed that one day and I got a bit panicky (laughter) so I… left it…I just use it for the questionnaire now and that's it." P145 [uses technology daily]* |
| Frightened they might break it | *"…plus it doesn't belong to us and if we broke it we'd be devastated" C145 [uses technology daily]* |
| Frightened they will enter wrong information | *"My fingers are too heavy. … I'll end up phoning somebody." P378 [once a week technology user]* |
| Technology use is stressful | *"But I can… if I'm, if I'm taught …without aggravation." C380 [uses technology every few weeks]* |
| Problems using technology perceived as failure | *"I tried to order a book on it last week and I can't do it (laughs) it kept, I've lost passwords and all sorts…" C392 [uses technology daily]* |
| **Low user's approach to technology** | |
| Use technology for only a limited number of familiar purposes | *"I have used the computer for various things, and if I'm using it for something and I know what I'm doing, that's fine; I can get on for certain things now…" C380 [uses technology every few weeks]* |
| Avoids using unfamiliar technology | *"Q: And, and you say you were a little bit worried about the technical side?*<br>*C: Well I am, I was… I can manage things… I use Kindle and a laptop. Computers are a bit more of a thinker… (laughs) and…I don't do them all the time so I'm all right with basic stuff." C062 [uses technology a few times a week]*<br>*"I know basically how to navigate [his own device], whereas [the TiM] I don't." P145 [uses technology daily]* |
| Unable to problem solve: relies on others | *"Q: And what do you do if you get, get stuck on a problem?*<br>*C: Have to wait till me daughter-in-law comes up and get her to sort it out for me." C392 [uses technology daily]* |

For peer review only

## Appendix table 3 Negative attitudes to technology expressed by participants.

| Negative attitudes towards technology | |
|---|---|
| Lacking an interest in technology | *"It doesn't really interest me, if I'm being honest." C392 [uses technology daily]* |
| Not seeing a need for technology | *"I'm not against it but I don't want it and I can do without it and I don't need it." C166 [never uses technology]* |
| Feeling pressured to use technology | *"All the time we're being pressured [by people] that take all this marvellous technology up; and every day it is getting worse and worse, because it's getting beyond the control of the average person how to manage it properly." C166 [never uses technology]* |
| Feeling excluded/missing out if not using technology | *"I do feel…a slight social outcast, but I seem to be very much in the minority." C166 [never uses technology]* |
| Worried about the consequences of technology misuse | *"… certain things are very useful [but] once it gets to the mass market…it of gets exploded and misused. … You've got to the point of small businesses being blackmailed by hackers." C166 [never uses technology]* |
| Worried about online security | *"And as for banking online, God, it's a laugh, I would not touch it…if something goes wrong you have the devil's own job to try and put it right because there's no proof." C166 [never uses technology]* |
| Worried about intrusion into their privacy | *"I wouldn't have a Smartphone for a start, you know cos Big Brother's up there already." C166 [never uses technology]* |
| Previous negative experience using technology that was unreliable | *"…eventually these programs got more and more complicated and the time it was taking for sending information down and getting back was absolutely ludicrous; the thing would crash say at eleven o'clock on a Monday morning and you'd have to start all over again." C166 [never uses technology]* |
| Technology is an unwanted replacement for human contact | *"They can't pick up a phone normally anymore because they're so busy faffing about with their smart phones." C166 [never uses technology]* |

For peer review only

## Appendix table 4 Facilitators for TiM use.

| | |
|---|---|
| **Facilitators for TiM use** | |
| **Improving confidence using the TiM** | |
| Face to face training | *"I would take it up if I was shown that the program was spot on" C166* |
| Clear instructions | *"…That the instructions were aimed at an idiot like me… and not on the assumption that I would know what to do, cos half the instructions are based on the person knowing what they're gonna do, so it seems to me." C166* |
| Participants realising it's easy to use | *"I thought I wouldn't be able to do it (laughter) but I can… I'm not really… I don't want computer or anything like that, you know, so it's only that only because of that." C228* |
| Using it weekly to develop skills | *"I was quite happy using it weekly, cos things can change so quickly…I'm a terrible, I'd forget when I'd done it the last time," C392* |
| Enabling others to help them use it | *"He's got used to it as long as I set it up." P217* |
| Seeing a purpose to use technology | *"It's unbelievable that sort of technology, but equally it's open to anybody to get into it. So, I might be persuaded eventually, but [laughs] it's a slow process" C166* |
| **Facilitators to use by those with disabilities** | |
| Stylus pen helped patients with upper limb disabled | *"Q: You mention you've got some problems with your hands. How do you find using your iPad?<br>P: It's a lot better since you gave me this [stylus] So I don't hold it like a button. Other than that it's great.<br>Q: Have you got any other gadgets like that?<br>P: No. [to C] You ordered some more because you liked it didn't you?" P172 [daily technology user]* |
| Touch screen is accessible | *"It's fine. I mean it's getting more awkward to me, for me cos obviously my hands are [weak], but, it's quite easy. Yeah, the touch screen, it's better than if you had to press this keyboard sort of thing." P056 [daily technology user]* |
| Use patients' own device | *"I think it'll probably have more of a take-up with people using their own device, because … I'm comfortable with my phone." P134 [daily technology user]* |
| Family/carers enter information on patients' behalf | *"Q: … is it something that you'd ask your carers to do or your family…?<br>P: Yeah, yeah, definitely.<br>Q: … would you have any concerns in them seeing your answers and that sort of thing?<br>P: No, I'd rather have the family do it rather than the carer do it, but I wouldn't have any problem, the family seeing it at all." P056 [daily technology user]* |



For peer review only - http://bmjopen.bmj.com/site/about/guidelines.xhtml

## Appendix table 5 Participants attitudes towards the frequency of TiM sessions.

| **TiM session frequency** | |
|---|---|
| Weekly sessions acceptable | *"Maybe once or twice a week would be enough." C063 (Frequent user)* |
| Weekly sessions make it part of routine | *"I'll keep it to Tuesday and do it every Tuesday while I'm working at home and I'll have peace and quiet." P122 (Excellent user)*<br>*"I was quite happy using it weekly, cos things can change so quickly…I'm a terrible, I'd forget when I'd done it the last time and … doing it maybe on the thirtieth of every month, I'd forget…" C392 (excellent user)* |
| Varying frequency according to speed of progression | *"Q: How often do you think you'd need to do it to make it worthwhile…?*<br>*P: Probably every month.*<br>*Q: …and if things changed how would you then approach it, would you go back and do it again or would you wait for the month…?*<br>*P: If things were changing quicker I'd go back in and do it again…" P423 (Excellent user)* |
| Carers may do it less often | *"Maybe carers don't need to do it quite as often but … maybe you would ask people how often they wanted to do it and maybe they would start at once a month and then as things progressed they did it a bit more often" C378 (control)* |

For peer review only


For peer review only - http://bmjopen.bmj.com/site/about/guidelines.xhtml

## Appendix table 6 Reasons for frequent and infrequent adherence to TiM identified during the interviews.

| | |
|---|---|
| **Reasons for missing individual sessions** | |
| Forgetting to use the TiM | *"I can't stand on my own so C has to bring it in here because I don't like to do it when he's working. So then I forget." P172 (Infrequent user)* |
| Patient and carer not doing it together | *"I'll do it and just leave it on the arm of the chair or something and say, "You need to do it". I don't know if she has on not." P122 (High user)* |
| Holidays | *"Oh dead easy; I know we do miss some weeks, but we've been, you know, if we, if we're away and we don't take it with us, we leave it here." P317 (High user)* |
| Acute illness | *"We just didn't do it, he weren't fit enough to do it." C228 (Frequent user)*<br>*"...when he was not very well he just couldn't be bothered." C366 (High user)* |
| Technical difficulties | See Table 6.1 & 6.2 |
| **Reasons for infrequent adherence** | |
| Poor health state at the start of the study | Reported in field notes made during calls with relatives. |
| Being too busy / having other priorities | *"It's just, with, with all the renovations and stuff it's sort of not become a priority at the moment." P134 (Infrequent user)* |
| Not receiving feedback and becoming demoralized with the TiM system | *"I have not received any feedback / contact from the STH MND team while using the TiM"* P047 |
| No current need for MND services so not seeing a benefit of TiM to their care | *"Well there'd be no point, would there?" P317 (Infrequent user)* |
| Unable to access the TiM tablet out without assistance | *"I can't stand on my own so C has to bring it in here because I don't like to do it when he's working. So then I forget." P172 (Infrequent user)* |
| **Facilitators of frequent adherence** | |
| Using TiM on the same day each week | *"We always, always remember that it's on a Thursday." P392 (Excellent user)* |
| Phone alarm reminders | *"Q: If it sends you an alarm reminder, would that be an annoyance...?*<br>*P: No, it would probably be more of a trigger for me to do it" P134 (Infrequent user)* |
| Family members reminding them | *"Our grand daughter rings us at about 7 o'clock to remind us. Then we finish up doing it on a Thursday morning." P217 (Moderate user)* |
| Being able to use TiM on their own device | *"If it worked on my phone there would be no issue whatsoever, cos I do everything on there; I shop... I can control lights, heating, everything on the phone. I think if TiM worked on there then you would get your responses every week." P134 (Infrequent user)* |



For peer review only - http://bmjopen.bmj.com/site/about/guidelines.xhtml

## Appendix table 7 Participants' expectations of the potential communication and monitoring benefits of the TiM service.

| **Potential benefits of the TiM identified by participants** | |
|---|---|
| **Improved communication with MND team** | |
| Increase speed of communication | *"If I put it on the tablet and I send it to you, you get it there and then. So if anything … happens to me in that period of time you know straightaway … So the quicker you can pick up on so, something it's better for you as a doctor as well as me as a patient." P091* |
| Increase frequency of communication | *"And you know somebody within the team is going to look at that information. So you are connected, once a week. So it's not every three months." C184* |
| Provides a direct connection with specialists | *"Well I'd read up about the telemed and I thought, well it gives you a direct connection with Sheffield and the team. I know it's an IT link but it's a definite link because once a week you are communicating with a team." C184* |
| Improve communication and liaison with other members of the MDT | *"Well I can't see there's a different way to what we're doing now; I'm quite surprised that you're all in communication really….I didn't know you spoke to [community MND team], and I didn't that [GP] and you and the hospital were quite so connected, so that seemed to be quite a positive thing." P354* |
| **Increased monitoring** | |
| To identify problems quickly | *"It just seemed a way of being able to communicate with, with my care team and letting them know on a regular basis how you're doing; and no doubt at the end of it (…) it flags up that I need help or I don't need help." P134* |
| To identify problems between clinic appointments | *"I thought it'd be a good idea for both of us, but mainly for P and, because it is a long time between the hospital appointments, and I thought well it's a good idea to monitor symptoms if they crop up out of the blue and we're not sure whether it's anything..." C062* |
| Enables a better understanding of the disease | *"When, when P started off with the home journey we, it was obviously dizzy spells…. So I said well we'll just keep a diary of what's happening so you yourself know what's happening, so that's what he's doing, and in effect, this is what, your telehealth could do eventually is sort of monitor people…" C091* |
| Enable self monitoring | *"So we can monitor ourselves as well at the same time. So it's helping you in the long run as well…I think you've gotta monitor yourself so you know how, what you can do and what you can't do. It's, you can't just rely on everybody on the end of a phone, pick up and say, you know, I've got this, that and the other. You've gotta look at health and safety for yourself as well, for whether you can walk or whether you can run or whether you can open a door or open a lid or carry a glass or whatever, you know, at the end of the day that's monitoring yourself, so you've gotta do it." P091* |



For peer review only - http://bmjopen.bmj.com/site/about/guidelines.xhtml

## Appendix table 8 Participants' expectations of the TiM service.

| Potential benefits of the TiM identified by participants | |
|---|---|
| **Psychological benefits** | |
| Clinicians take an interest | *"Somebody's taking an interest, somebody's wanting to know" C184* |
| Reducing isolation | *"You're not going to feel isolated." C184* |
| Reassurance | *"Sometimes it's just the fact that, "should somebody know about this?" or "should you be telling somebody that." Wouldn't it be nice if somebody knew this. Just small details that you sometimes think, "Does it make a difference if somebody knew about it?" And that telemed makes that difference." C184* |
| **Improve accessibility of the MND service** | |
| Reduce clinic appointments | *"If you can glean enough information of what I send through so I don't have to come to the hospital, I think that's a positive" P354* |
| Provide access to MDT when unable to travel to clinic | *" We just thought it would be quite good cos not everybody can get into clinic and thinking about the later stages, but it's still important to monitor how things are going" P480* |
| Reduce travel time | *"that would then cut down my travel time to clinic, you know, clinic doesn't really fulfil a great deal in my life other than ticking some boxes and sort of knowing where the progression's going but, you know, I think most of that now can be done through this server, through TiM." P134* |



For peer review only - http://bmjopen.bmj.com/site/about/guidelines.xhtml

## Appendix table 9 The acceptability of the TiM questions.

| Acceptability of the TiM questions | |
|---|---|
| Patients wanted the TiM to include questions about all aspects of MND | *"I've found the questions, I think they're all relevant. And, ... it might be in the future when things do, if things do get worse it might be I might need those questions to highlight things and that's quite, quite comforting." P423*<br>*"There's one question about you use a feeding PEG. I don't at the minute so that's not relevant to me at the moment. But obviously as time goes by it, it will be. I think I just put, "no", I don't use one... But I think it should be on there because, ... in time I probably will be using one." P145* |
| Emotional wellbeing questions were welcomed | *"I think it's important to find out, not just physically, how a disease affects somebody but how, emotionally, mentally it affects them. As a say, I'm quite a positive person, but, I have some off days." P122* |
| Carers happy sharing their sensitive information | *"Q: The sort of questions that they're asking you, quite, they're quite personal questions, aren't they...? How you feel about sharing those?*<br>*C: No problem." C392* |
| Questions made patients think about the consequences of their disease, but this was not a problem | *"Q: How does it feel having to sort of be faced with that question [about gastrostomy tubes]?*<br>*P: ... when I first did that it was dead easy cos I just put "no" ... and just moved on. But since then, since we've been to this Advance Care [planning] thing. ...And that, that sort of brought it to the fore .... it's a thought that I wouldn't really wanna think about, but you've gotta think about, so I think on that one, is that something I shall maybe ask more to get more advice on." P248* |
| Reporting a deterioration on the TiM was not distressing | *"I think: am I getting worse? But, yes, am I getting worse.... I don't think any of the questions are really frightening. You know it might happen." P172*<br>*"in a sense it's good that you can change, you know, the, keep every, everybody up-to-date. Cos you don't know when things are going to change anyway, do we, we don't, you know, it's just, it's like a bit of a, a grey area isn't it? ... as long as people who need to know see that information then it's ... not waiting till your next visit at the clinic is it...?" P122* |
| Wanting questions that assessed how they were coping | *"Maybe if they say, "No, I've not been out for a fortnight", that it makes them think: well, actually why haven't I? And it would highlight that person's not getting out of the house and not doing something themselves and they are continually there. Do they need a bit more support?" C122* |
| Questions were repetitive | *" It's repetitive. ... I pick it up and I think oh God, here we go again, same old, same old. [I] put a couple ...more different ones in,... but no, the questions are right..." C145* |
| Questions become irrelevant if disability severe | *"As I am unable to use my arms and legs at all, many of the questions have become irrelevant to me beyond the first time of using it."* P047 |



For peer review only - http://bmjopen.bmj.com/site/about/guidelines.xhtml

## Appendix table 10 The validity / accuracy of the TiM questions.

| **The validity/accuracy of the TiM questions** | |
|---|---|
| Patients want answers to be accurate | *"If I've got a form I like to fill it in as accurately as possible." P248* |
| Patients were concerned that their answers would be misleading | *"I have lost weight [a few weeks ago] but I haven't [lost weight in the last week]. The way I'm reading it now… sounds like I'm still losing weight, well I'm not…I wouldn't like 'em to think looking at it, or you looking at it in Sheffield and thinking "God, he's still losing weight" P248* |
| The answers are insufficient to reflect small changes | *"Sometimes they answers to chose are too far apart so if the answer is one day a week, I answer no, or several, it's nearer several. So it's accurate but it's wide." P172*<br>*"The questions are quite in a narrow band, and because it's a slow-burner not much changes unless there's a step change, such as …the chest infection the other week." P354* |
| The condition varied from day to day making weekly assessments difficult | *"I thought: if I was doing the questionnaire on that day, "what sort of assistance do you get from your family? The answer would be off the scale somewhere. I thought: some of the questions don't quite fit the answer. So I try to put the most representative" C172*<br>*"I think some of the questions are a bit too general and wide rather than, for instance… what can I not do with my hand that I used to do, and how am I dealing with it." P166* |
| Informal care requirement questions were difficult to answer | *"The margins are too wide. One of the questions is, how much time do you spend in the day looking after her? And, there's a sort of, there's 3-4 hours. But I don't even look after myself for 3-4 hours, I just potter around. That's always gonna be the same answer." C172* |
| Some questions contradicted each other | *There are some questions on there that are bit ambiguous for me [for example] being able to use the stairs. Well I can't use the stairs but I still have a bathroom upstairs so I still walk from the top of the stairs to our bedroom." P134* |
| Patients wanted to provide more information to clarify their answers | *"… if you have any more questions to ask you've got that… availability to ask [the MND team] if you have any problems…" P056*<br>*"If you want to ask anything maybe you could type it in instead of it just being yes/no" CaT Patient 317*<br>*"I think that, that would be a good addition. Because you, you could answer a question and at the end of the section just put in, and just expand on what, why you've answered that, if you see what I mean? I think a comments box would be a good idea." P122* |
| Same problems identified every week | *"It's a probably the same answer I give every week… It says; "Do you stumble or feel that you fall, or have you fallen?" And I do, every single week, I guarantee that" P122* |
| Answer affected by other health problems | *"So at the minute I'm slightly doctoring the answer; so I'm saying I can dress myself but I can't, but it's not the MND." P381* |



For peer review only - http://bmjopen.bmj.com/site/about/guidelines.xhtml

# Appendix table 11 Participants understanding of the TiM service.

| | |
|---|---|
| **Aims of the TiM service:** | |
| Monitoring | *"For you to be aware of it, and monitor it..." P402* |
| Relay information to the MND team | *"It shows how you're feeling which gets then relays to the nursing staff. If you've any questions you've got the item on it where you can … ask that as well… I would think… it's read, isn't it, and then probably recorded somewhere." P056* |
| Look for trends | *"I would say they are probably going on to some sort of graph that detects a trend. I mean with me, you wont get a trend because sometimes you are up and sometimes I'm down. It'll look like a [gestured up and down]. But I would imagine the ideal situation is when you can detect a trend but that does depend on what's happening." C172* |
| Provides trends and alerts to problems | *"I'm guessing that it comes to someone's desk and that they're able to see a chart… from week to week, and I would imagine that there would be a series of red flags for you or some traffic light system… that red's intervention required, amber's a warning, green's get on with it.." P134* |
| Prioritise patients in need | *"I could see the reason why you were doing it; I realised that all the data was going to be collated and you can see then at a glance… you can see the statistics and everything, and it would highlight to you… if I had a dramatic change." P317* |
| Data would be used for research | *"I didn't realise that that's what was going to happen, I thought it was just being used for research." C392*<br>*"I would assume it goes to some databank somewhere and they try and correlate the answers I've given with, perhaps somebody else … to see if there is any common ground."P122*<br>*"I imagine it goes onto a database … and then if yourself and [Professor] are ever doing any research they may be able to…or if there's any trials you might be able to select from that who would be suitable for that trial." P 317* |
| Both clinical care and research | *"I imagine it goes on a survey of all the people you're doing to compare us with each other, and also to follow my own progress." P166* |
| **The role of the Telehealth Nurse** | |
| TiM Nurse looked at data | *"It shows how you're feeling which gets then relays to the nursing staff, doesn't it." P056* |
| EH was looking at data | *"I didn't realise that [Telehealth Nurse] was involved and she would ring us if our answers drastically changed, cos obviously they've stayed very much the same. Then I'm quite encouraged by that, I just presume that you do it yourself…and if you'd got a problem you'd ring [Telehealth Nurse] up for a bit of help." C392* |
| Unsure who looked at data | *"I'm guessing that it comes to someone's desk and that they're able to see a chart" P134*<br>*"The ones that have spoke about it to me I don't think they realise that I'm seeing their responses." Telehealth Nurse* |



For peer review only - http://bmjopen.bmj.com/site/about/guidelines.xhtml

## Appendix table 12 Nurses' experience of using the TiM clinical portal. All quotes are from the Telehealth Nurse.

| **Using the clinical portal** | |
|---|---|
| Easy to use | *"The training that I've had from seeing it this side was brief from Esther and it's, it's never, it's not difficult so it, I find I, right from the beginning I found logging on has been the most difficult thing, so; but once you're on finding your way around it is very, very easy, very easy,"* |
| Low burden | *"It only takes minutes"* |
| Easy to understand the flag system | *"If everything's OK it's green, … [if] there's maybe some elements that might need to be looked at, it's an orange or yellow, and then if there's an alert it's a red one."* |
| IT support for passwords required | *"My main problem was me accessing it to begin with, and that was very problematic. It's just asked me to change me password."* |
| Nurse didn't know how to use the patient TiM app or resolve technical problems | *" N: I have had a couple of phone calls, cos [the participants have] got my contact number through this, and they've rung with a problem, usually a logging on problem and…*<br>*Q: Forgotten their password?*<br>*N: Yeah…I've asked Esther, to be honest, because I'm not familiar with their device."* |

For peer review only

## Appendix table 13 The accuracy and sensitive of the TiM information. Quotes are from the Telehealth Nurse (N) unless otherwise stated.

| **Accuracy of the TiM information** | |
|---|---|
| Information accurate to help make some decisions | *"N: [EH] has led me to believe that it's quite easy to red flag on the carer. So this chap is triggering … [reads the PHQ4 questions] So I mean … the four that are triggering, they're all about…<br>Q: Worry, stress?<br>N: Yeah, thoughts: yeah. I know how poorly this lady is, I know how disabled she is, and I do know him, and when you speak to him he's very blasé about it all, which actually is more of a worry."* |
| Information in TiM wasn't sufficiently detailed enough to be sensitive | *"N: …I think if they're routinely sending us an update every week I think you're more likely to pick up on problems, but I don't… I thought that would be so.<br>Q: Is that, do you think that's happened?<br>N: No not really. I don't know that the questions are sensitive enough, and I suppose the thing is that it might, if it triggers a contact phone call then you may well pick up on things sooner." (early interview)* |
| Needed more information | *"N: It's almost like you need a two-way thing (laughs) you need to ask them a question, [for example] how long have you been coughing… are you bringing anything up when you're coughing… you know, that type of thing… have you had a temperature… it's almost like [laughs] you need a two-way communication, cos this is just a snapshot, isn't it…"* |
| Discussions are required to fully understand a problem | *"I think some of the problems with patients using NIV, very specific problems, you only pick up from a conversation with them. I don't think you pick it up on the Tele, cos it's [the way] it's structured, it might show a problem but not a specific problem."* |
| An additional comment box would help gain more detailed information | *"Q: Yeah. Do you have, would you have time to read the comment boxes?<br>TW: I would hope so. You'd have to make time. If it was a problem that was coming up all the time, yes, I would hope that that; in some ways that might save you time."* |



For peer review only - http://bmjopen.bmj.com/site/about/guidelines.xhtml

## Appendix table 14 Participants' experiences of how the TiM impacted on their care.

| Impact on if TiM on patient care | |
|---|---|
| Improves knowledge | *"Because since I've done it I'm taking more notice of my weight but I didn't before so I know it very carefully. It made me aware of that." P172* |
| Close monitoring | *"I'm certainly not left alone for more than a month, ever...Sheffield is there if I really need them, but on the other hand [local team] is here." P166* |
| Reassurance to know being monitored | *"I think the benefit to P is real. Because... somebody is there on hand looking at things... Because it's slow with P and he doesn't need as much attention and care, it's easy to feel detached from any positive interaction." C122* |
| Keeping in touch with specialist | *"...it's knowing that somebody else is in your corner." C423<br>Q: Do you think there might have been a point in your disease where those questions ...were useful?<br>P: Nearer the beginning, definitely. I think, and I can only guess, that it must be the same for lots of long-term conditions; you know, I've lived with this for over five years now... you get to understand your own body and you know it's, it's normal for me now and for the first year no day was normal, no day looked like any other day in my life before that. So... if I could have camped in [MND consultant]'s house for the first six months I would have done, just so she was there, so I could say, but what about this, what about that; and you imagine symptoms, or I did, you know, you think God, this is happening and that must be related to the MND... So in the first year I would have filled that in every day, just to have that touch point," P047* |
| Nurse giving advice | *"When I came back on Tuesday last week and I did the second questionnaire, and within a day [Telehealth Nurse] saying "I've got a red flag on one of your answers." And it's the fact that I'd fallen twice while I was away on holiday and I'd put on it, you know: it said "have you fallen recently, how many times?" and I'd fallen twice while I was on holiday. So she phoned me, and said, "Are you ok? Is there a reason why you fell?" No, just my usual clumsiness....<br>Q: Were you expecting her to call?<br>P: No, I wasn't actually. It was just a bolt out of the view...I find that quite positive. It shows that the whole idea of it works.<br>Q: Has it changed your behaviour at all?<br>P: No. Not really." P122* |
| Nurse identifying problems | *"Q: [Telehealth Nurse] called, I think she spoke to you about when you fell...<br>P: Mm.<br>Q: ...what did you think about that when that happened?<br>P: It was useful wasn't it?<br>C: Well you weren't in so she spoke to me.<br>P: Yes, because I'd tripped over the bedroom chair...<br>C: that's right and, yes, that was the main thing, that's right, yeah. No, it was interesting that that, that had been picked up because we weren't, we don't know how it was picked up." P&C166* |
| Identifying problems between clinic visits | *"When I filled it in last week, and within a day [Telehealth Nurse] was phoning me. How much better could you have that? Instead of, two months down the line and I attend the clinic and they say "how have you been, have you fallen?" and I say "oh yeah I did: two months ago". See where I'm coming from? You've got that instantaneous contact with this technology that perhaps you don't have without it. So I'm a great believer in that, I'm a great believer in technology." P122* |
| Supporting important decision making | *"Q: The other question I had was that we kept an eye on your weight and I wonder whether you think that may have influenced your decision to have a feeding tube or not; do you think it, do you think it had?<br>P: [writing] I was frightened by the speed of loss of weight but was convinced how much muscle I lost." P409* |
| Help accept the disease | *" The questions nudged me to facing what I could do and not what I can't." P409* |



For peer review only - http://bmjopen.bmj.com/site/about/guidelines.xhtml

## Appendix table 15 The Telehealth Nurse's attitudes towards the value of the TiM.

Quotes are all from the Telehealth Nurse

| **The Telehealth Nurses' attitudes towards the value of the TiM** | |
|---|---|
| TiM could be valuable | *"As a tool to aid the patient and then aid the nurse"* |
| To identify problems between clinic visits | *"A number of patients with MND and their carers will wait till a clinic, instead of contacting us with, if they're, they're worried about something, or there's a change in their, you know, their condition, and I think if they're routinely sending us an update every week I think you're more likely to pick up on problems."* |
| Alerting to problems earlier | *"Q: So it could have a use maybe in alerting you to earlier need for intervention?*<br>*N: Yeah, yeah."* |
| Help monitoring trends | *"I mean the weights have been quite interesting, cos if they can use the weighing scales ... that's been quite interesting, so we can monitor their weight.... cos otherwise you wouldn't necessarily see that variation."* |
| TiM wouldn't have a negative impact on the service | *"Q: do you think a patient will be concerned this is trying to take away a part of the service, or do you think the clinical team might feel it's taking away part of the service?*<br>*N: I don't think either side would. From a clinical side I think that anybody would be willing to make it as easy for the patient as possible."* |
| Clinics are a burden to patient | *"I think from the patient's point of view I think it becomes very burdensome, the travel into clinic, very much so...But certainly the, the travel and the amount of time and effort for them to come to clinic to sit in clinic to then go home again, it's very difficult for them."* |
| Patients do not see value in attending clinic | *"... [patients] sometimes say;* "nothing, I don't get anything out of coming to clinic because you're reiterating the same things, I know I'm getting worse..." *So there are some patients that don't see the value of coming to clinic anyway. Now whether they would use a system like this and see the value of that I don't know."* |
| TiM could allow patients to be managed remotely | *" I think it could. I think it's one of those difficult things that at the moment, because it's not something we systematically do. And they are attending clinic or I have connections with their community care team, so I am in touch with what's happening and things are getting monitored. I don't know."* |
| Some problems need face to face assessment | *"Q: How reliable this kind of technology would be in deciding when or, when to cancel an appointment for a patient?*<br>*A: Oh I don't know, I don't know.*<br>*Q: Would you feel secure looking at that saying that patient doesn't need to come in?*<br>*A: I think, I think it depends, so when I look at, if it's a breathing problem and if I didn't know them and they were having problems with their breathing ...that would alert me and I would [think]: right we need... to contact, we need to be looking at this and monitoring it...I think it depends."* |

For peer review only

## Appendix table 16 Participants experiences of clinician feedback.

| To act upon problems and provide feedback | |
|---|---|
| Feedback reinforces the benefit of the TiM | *"I find that quite positive. It shows that the whole idea of it works." P122* |
| Not receiving feedback is demoralising | *"The emotional psychological depths that I've been to, I was putting the stuff in Telehealth and thinking but nobody's acknowledged this or contacted me about it, and I thought: well they're not going to because that isn't what the clinic's about, and that made me stop using it." P047*<br>*"...it's like all forms and all surveys... they say "your opinion is important please fill in the following..." and you say something that you think is absolutely dramatic and mind blowing and nobody comes back to you on it. And you think: well how important is that survey?" C172* |
| To provide feedback even if nothing had changed | *"Q: And would you expect perhaps someone to tell you what's going on with the tablet or the answers?*<br>*P: Yes. Because if I hadn't changed much, I would have thought I would have had some feedback."P172* |
| To acknowledge problems even if nothing can be done | *"[falls] ... knocks your confidence ...I probably were putting too much onus on Sheffield Hallam because (laughs) we've got this and there's not jack shit they can really do about this and we know that...*<br>*Q: But that kind of acknowledgement's quite important, do you think, of the, what happens ...?*<br>*C: I do, yeah, it's a bit of support, in't it, it's knowing that somebody else is in your corner." C423* |
| MND team couldn't solve their problem | *"Q: If your answers changed what do you expect of the MND service?*<br>*P: I don't, to be honest. Let's say if I thought I'd got a problem I wouldn't necessarily come to you because I don't think, I think, you know, I, the impression that it's medical to do with P, and if I have got a problem really; because when you flash up it does say if you, is to contact the, there is, so I would probably, I mean that's not part of your remit, is it, me really if I've got a problem?" C381*<br>*"[emotional support] That's not what that clinic's about, that clinic's about physical wellbeing and physical health... truthfully I thought I don't know why these questions are in here, because that clinic isn't equipped to deal with that." P047* |
| Feedback thought to be pointless if nothing can be done | *"But then again, what's the point of coming back if you can't say anything?" C172* |



For peer review only - http://bmjopen.bmj.com/site/about/guidelines.xhtml

## Appendix table 17 The mismatch between the participants' and nurses' expectations of the TiM service.

| **Mismatch between patient and nurse expectations** | |
|---|---|
| Patients expected contact if they experienced important problems: | |
| Falls<br>Chest infections<br>Dysphagia | *"Q: ...do you, do you think they're important things that she needs to know about?<br>P: I would say yeah… cos one of the questions is have you fallen or tripped? Obviously if I am falling more often, (well I am, I fell a few weeks ago, didn't I? but that, that's first time in months...). If I was falling … over every week I would say that, yeah, she needs to know something like that, or if I'm starting to not eat me food properly, if I'm starting to cough, I think obviously she needs to know that" P145* |
| Emotional difficulties | *"Most of my problems at the time are emotional, and the TiM does not appear to trigger any intervention." P047* |
| Problems may not be seen as important to nurse | *"She red flagged that she'd fallen, which is quite a common occurrence on a lot of patients, and I don't particularly worry unless they've been very, very well and then suddenly." Telehealth Nurse* |
| Problems may not be seen as important to patients | *"Q: When you put that you had fallen, what were you expecting to happen?<br>P : No. No.<br>Q: And when you heard that [your OT] had heard about it, what did you think about that?<br>P: Nothing really.<br>Q: And, do you think in the future, if something were to happen to you, and you put on here, what would you expect to happen, what would you like to happen?<br>P: I would think [Telehealth nurse] would ring. But the only thing is the over balancing, so far, isn't it." C217* |
| Problem may not be seen to be under the MND team's remit | *"[emotional support is] not what that clinic's about, that clinic's about physical wellbeing and physical health. To me, and in my own head, I thought it's never been about psychological wellbeing and your mental wellness and therefore; and truthfully I thought I don't know why these questions are in here, because that clinic isn't equipped to deal with that." P047* |
| Participants expecting contact when stable | *" It would be nice for someone to phone occasionally … particularly if anything changes.: C172* |
| Not expecting a particular action: expect nurse to decide | *"Q: And what would you expect to happen if you did change your answers like that week?<br>P: Not really, no, cos you know more than I do." P056<br>"I would tend to leave it to them to solve anything that needed solving." C217* |

For peer review only

## Appendix table 18 The problems associated with excessive TiM alerts. All quotes are from the Telehealth Nurse unless indicated.

| **Excessive TiM system alerts** | |
|---|---|
| The same alerts appear every week | *“We have some of our patients that do this every time… They … cough or choke at least once a week.”* |
| Alerts increase the time required to use the system and can cause frustration | *“Then when it was coming up every week… I knew I’d spoken to them about that [problem] and that was their choice. And I’d kept putting [a comment in the TiM notes] but you’re sorta thinking; why do I have to keep putting it on every time?”* |
| Nurse can’t control the alerts | *“If you know somebody’s got a problem and they’re not really trying to do anything about it, then you know that [alert is] gonna keep coming back every week; and I haven’t been putting comments on all the time.”* |
| Nurses may appear to not be acting on potentially dangerous problems | [1]*“[reads]* “Do you ever cough or choke on food?”*And then he’s put:* “Occasionally”, *and that’s fine.”* |
| Problems flagged even though the patient has chosen not to medical advice to avoid the problem | *“cos I know that patient and I know that they’ve chosen to eat and that it is problematic. But they’ve got a feeding tube and they should really be using their feeding tube, but they’re [also eating]. Then when it was coming up every week, I knew I’d spoken to them about that and that was that choice?”* |
| Reporting the same problems without solutions could be demoralising to patients | *“Maybe to them the fact that it’s the same thing week in week out, they’ve got an insight into that problem, it’s not changing and, so they’re not looking for something to help with it really.”* |
| Repeated problems on which no action could be taken should be paused to avoid excessive alerts | *“Q: Are there any other, other things that you’d change at the moment to make it, to improve the system?<br>TW: Apart from the, the same red flags coming up every week with the same problem, having some way of either taking them off or say, or putting a comment in that, that would take it off by saying you’ve addressed this problem, it’s, it’s still gonna be there, it’s not gonna change.”* |

For peer review only

## Appendix table 19 The problems associated with excessive TiM alerts.

All quotes are from the Telehealth Nurse.

| **Nurse reaction to alerts** | |
|---|---|
| Calls patient for more information | *“[I] ring the patient about it and ask them”* |
| Liaises with the community team to get more information | *“I try and get a bit more information, cos the, the community team may have been out and seen them and seen them face-to-face and have got something a little bit more useful back; so I have used it for that circumstance. And I’m in touch with the community team quite often, so sometimes if I’m ringing about something else I’ll ask about one of the patients that’s on here.”* |
| Prefers to wait until clinic | *“I’ve seen somebody that was red flagged, cos they’re coughing more..I’ve put a [TiM note] that we’ll review in clinic next week.”* |
| Will chose not respond to problems she thought were common in MND | *“She red flagged that she’d fallen, which is quite a common occurrence on a lot of patients. I don’t particularly worry unless they’ve been very, very well and then suddenly. So if it happens over a few weeks and I’ve spoken to them and I know the situation…I’m not always alerted by that red flag.”* |
| Using all the TiM information on the to make a decision | *“They were having problems with their breathing but I would look: are they on a breathing machine? No. That would alert me. So I think it depends.”* |
| Patients and nurses prioritise different problems | *“I did the second questionnaire, and within a day the [Telehealth Nurse] calls saying “I’ve got a red flag on one of your answers.” And it’s the fact that I’d fallen twice while I was away on holiday… she phoned me, and said, “Are you ok? Is there a reason why you fell?” No, just my usual clumsiness….*<br>*Q: Were you expecting her to call?*<br>*P: No, I wasn’t...It was just a bolt out of the view… I find that quite positive. It shows that the whole idea of it works.” P122* |
| Having a relationship with the patient made it easier for the nurse to call the patient | *“I think it helps me that I know the patients. So I know this chap very, very well, I have a relationship with him and his wife; ringing up’s quite easy to do, they wouldn’t, they wouldn’t be fazed by that. My response would be;* “oh there’s been a red flag on the Telemedicine that you sent through, a message that you’ve sent through, and that’s why I’m ringing”*. And that was quite an easy one.”* |
| Harder to ring those who she didn’t know | *“There are a small number of patients that I don’t know, it just so happens that they’ve been very well through, so there hasn’t really been any major alerts.”* |
| Ringing carers may be difficult | *“If we’ve had a carer’s response that’s red flagging I don’t always find that an easy phone call to make to a carer who’s struggling.”* |
| Patients not expecting her call | *“I rang a lady who I didn’t know and she red flagged that she’d fallen… It was the lady’s husband … he was quite shocked that I’d rung cos I didn’t know them and I just explained about it, and he just said “No, it was just a little trip, she’s absolutely fine, no problem.”* |
| Nurse doesn’t feel her calls benefit patient | *“I’ve said “Oh you’ve had some problem with this?...I know because you sent a Telemedicine and I’m the one that looks at the problems and sees what’s what.” And they said “Oh right, oh yes, it’s nothing, it’s fine.” I don’t know…”* |

# Appendix table 20 The future of the TiM.

| **Preferred way to communicate with MND team** | |
|---|---|
| Modes of communication depends on the individual | *Q: How do you think is the best way for people contact you?*<br>*P: I think for me it'd be by phone, but again that will be depending on the carer and my speech. I mean I'm currently OK with the phone.*<br>*Q: And how do you feel about email contact or contact through the Telehealth?*<br>*P: Again that's fine by me, I use email, computer all the time, but again you'd have to judge that on the individual." P381* |
| Contact needs to be convenient | *"And phoning you up, I don't know about you but I hate phone calls: you are just settling down to have a nice cup of tea or a chocolate biscuit or whatever and the phone rings, you have someone trying to persuade you to change your heating systems" C172* |
| Alternative methods of communication add flexibility | *"I'm really happy …communicating by email, because for me I can do it then in my own time, because I haven't got use of my hands and arms; so if I know that there's an email that I can read and then reply to in my own time, that's far more relaxed for me actually than the telephone… I can speak fine but the telephone, somebody has to hold it for me…if I'm not in exactly the right position it's not comfortable to take a r." P047* |
| Telephone may be uncomfortable for some | *"I feel a bit awkward on the phone, and you can't get across how you're really feeling on a phone anyway..I tend to get nervous when I'm on the phone and stuff and I forget what I'm saying to people." P423* |
| Dysarthria makes telephone hard | *C: [to P] Well telephone calls aren't that practical are they for you. Because if you are tired, I mean it's difficult to convey."C172* |
| Email may be impersonal | *"I think that's a bit impersonal email, you know, that, that's what I thought" C423* |
| Telephone useful for problems needing an immediate answer | *"If it's something that needs doing, dealing with here and now: the telephone, email is, is my favourite way of communicating, because it suits my condition.." P047* |
| Some subjects are better discussed face to face | *"I think it's not personal... I'd rather see somebody or talk to somebody than, than read about it on something on a screen." C366* |
| Happy to receive feedback in clinic | *" I realise that you are probably busy people, so I'm quite happy for someone to say, when we're down there, "oh by the way, your survey has altered and, you know, do you want to talk to you about it." C172* |
| Happy to wait for a reply as long as their message had been acknowledged | *Q: And if you put in a question... How long would you expect before you got an answer?*<br>*P: Well normally [Telehealth Nurse] gets back to me within a day or so. So I would expect two days, almost maximum.*<br>*Q: OK. So, reasonably quickly.*<br>*P: Even if, it was: "we've got your question :we're thinking about it"*<br>*Q: So, just an acknowledgement that you've email?*<br>*P: Yeah. It may be [Telehealth Nurse] can't answer me first time. Like the citalopram. What she said was: "I'll discuss it with, (I think) you" and then came back to me." P381* |
| Importance of respiratory monitoring | *"Q: What do you think, how do you think this would fit in around your clinic visits and the support you would get from [Telehealth Nurse]? If this was to become...rather than a research project, part of standard care, how do you think you would suggest using it?*<br>*P: Well I think in my case I would be happy to use that and lengthen the time between visits. I mean, the only difference to me is the breathing test." P381* |
| Happy to share the information with other members of the care team | *"Q: And is there, is there someone locally that you think would be, so that would be able to see your answers ...?*<br>*P: Well a good one would be the GP, wouldn't they? Or even the hospice, or, even the district nurses, probably more the district nurses, cos obviously they've got a regular visit now, haven't they?" P056* |



For peer review only - http://bmjopen.bmj.com/site/about/guidelines.xhtml

## Appendix table 21 The Telehealth Nurse's attitudes towards how the TiM might be used in the future.

Quotes are all from the Telehealth Nurse

| **How would the TiM be used?** | |
|---|---|
| Delegating TiM duties may not be possible | *"I agreed to do it originally cos I said the thing is when a red flag comes if it's somebody else doing it and looking at it they're gonna come to me."* |
| The nurse wants to use her judgement, not follow a strict protocol | *"The way this study is run at the moment I can respond in, in the way I think is appropriate" Telehealth Nurse*<br>*"if it was written down, [that] I had to ring and I had to ring straightaway, … I don't know whether, I would have found that quite difficult not being able to use my initiative and how I'm familiar with the patients and, don't know I might not have, I might have found that a bit more difficult."* |
| Some nurse may be more willing to follow a protocol and call more often | *"N: It's difficult, I think you would probably get more useful information out of the research nurses, but you would have to have a system where they'd be able to go to somebody to act upon what was [needed]" Telehealth Nurse*<br>*"I think if it was a bigger study and.. it was a very … carefully monitored study, I think maybe the person doing this, looking at this … looking at what the replies have been, if they had to contact them that might work better."* |

For peer review only

## Appendix table 22 How the TiM might be used by other teams

| | |
|---|---|
| **Use by community teams** | |
| Community teams would need capacity | *"I think if they have the capacity it would be useful," Telehealth Nurse* |
| It would increase information available for community teams | *"Q: Could [you] see [the TiM] fitting into the role you play or whether you think it's not very helpful?<br>N: I think it probably could because, like you've just said.. that [patient has] reported on here that she's … coughing/choking so many times but she never said that to me." Community nurse* |
| Community staff may not have access to computers | *"I'm not very good at computers. I have to say, that is the thing I find most stressful about working here. You I'm home based. I go on NHS website...so I'm not on, well I can get onto it but I do not cos I don't spend a lot of time [in the hospital]."Community nurse* |
| Community staff happy to receive information from Telehealth Nurse | *"Q: If someone else was looking at this, telling you there's a problem?<br>N: Yeah, no, that would be fine, that would be fine, yeah, yeah."Community nurse* |
| **Use by other MND centres** | |
| Other services would respond positively | *"I think their gut reaction is that that sounds something potentially helpful to us; I haven't really had any negativity" Telehealth Nurse* |
| Other services would want TiM if it saved them time | *"I think that if it saves them time I think that they would definitely embrace it." Telehealth Nurse* |
| Other services would want TiM if it benefited the patient | *"From a clinical side I think that anybody would be willing to make it as easy for the patient as possible, as long as it was useful." Telehealth Nurse* |
| Nurses would use it if it were part of their usual role | *"Q: But then if you were thinking about implementing this into the NHS who would you get the most useful information from?<br>TW: I think if it was implemented and it was part of somebody's role every day they would just do it, they would do it automatically; and, yes, I could envision that it would be very useful. So I suppose I'm a bit, it's half and half." Telehealth Nurse* |


For peer review only - http://bmjopen.bmj.com/site/about/guidelines.xhtml
